# Humoral Immune Response Profile of COVID-19 Reveals Severity and Variant-Specific Epitopes: Lessons from SARS-CoV-2 Peptide Microarray

**DOI:** 10.3390/v15010248

**Published:** 2023-01-15

**Authors:** Arup Acharjee, Arka Ray, Akanksha Salkar, Surbhi Bihani, Chaitanya Tuckley, Jayanthi Shastri, Sachee Agrawal, Siddhartha Duttagupta, Sanjeeva Srivastava

**Affiliations:** 1 Department of Biosciences and Bioengineering, Indian Institute of Technology Bombay, Mumbai 400076, India; 2 Centre for Research in Nanotechnology and Science, Indian Institute of Technology Bombay, Mumbai 400076, India; 3 Kasturba Hospital for Infectious Diseases, Mumbai 400011, India; 4 Department of Electrical Engineering, Indian Institute of Technology Bombay, Mumbai 400076, India

**Keywords:** COVID-19, SARS-CoV-2, peptide microarray, humoral immunity, IgA, IgG, epitope mapping, SARS-CoV-2 variant

## Abstract

The amaranthine scale of the COVID-19 pandemic and unpredictable disease severity is of grave concern. Serological diagnostic aids are an excellent choice for clinicians for rapid and easy prognosis of the disease. To this end, we studied the humoral immune response to SARS-CoV-2 infection to map immunogenic regions in the SARS-CoV-2 proteome at amino acid resolution using a high-density SARS-CoV-2 proteome peptide microarray. The microarray has 4932 overlapping peptides printed in duplicates spanning the entire SARS-CoV-2 proteome. We found 204 and 676 immunogenic peptides against IgA and IgG, corresponding to 137 and 412 IgA and IgG epitopes, respectively. Of these, 6 and 307 epitopes could discriminate between disease severity. The emergence of variants has added to the complexity of the disease. Using the mutation panel available, we could detect 5 and 10 immunogenic peptides against IgA and IgG with mutations belonging to SAR-CoV-2 variants. The study revealed severity-based epitopes that could be presented as potential prognostic serological markers. Further, the mutant epitope immunogenicity could indicate the putative use of these markers for diagnosing variants responsible for the infection.

## 1. Introduction

The COVID-19 pandemic has been an epoch-defining moment in human history. COVID-19 has left behind an inexplicable trail of death and disease [1]. Epidemiological studies indicate that the actual number of cases could be inordinately higher than those reported [2]. Dedicated instrumentation and the risk of handling live viruses add to the woes of using nucleic-acid-based technologies [3]. Serological techniques are more robust in this regard as they are less susceptible to false-positives [4,5]. Omics technologies and big data analytics often have led to a panel of biomarkers for the prediction of disease severity [6,7,8,9,10,11] from the early days of the pandemic. However, they are of little or no translational value, especially in the event of a colossal hospitalization burden, as is often the case in outbreaks. Though we are in the third year of the pandemic, we are yet to find a reasonably accessible mode of detecting disease severity. Increased susceptibility of a certain group, such as the elderly population, those with pre-existing comorbidities [12], and immunocompromised patients, remains the biggest challenge in controlling the pandemic. Moreover, the variant-driven resurgence in COVID-19 cases further complicates the scenario [13,14,15]. With NGS as the primary way of finding the causative strain of the virus [14,16,17], clinicians and frontline workers have little or no means of fighting this amaranthine scourge when fresh outbreaks occur due to the emergence of new variants. Serological tools to diagnose and prognose the disease development in patients can aid in this regard. Moreover, serological tools to detect variants can be a good alternative to NGS.

Before the development of peptide microarrays, antibody detection has been traditionally handled using tools, such as the Enzyme-Linked Immunosorbent Assay (ELISA), Western blot, radioimmunoassay (RIA), flow cytometry, and platforms such as Luminex. Advanced variants of ELISA, such as Chemiluminescent immunoassay (CLIA), have also come to the fore. A diagnostic ELISA technique was used to identify MERS-CoV viral antibodies in patients with Middle East Respiratory Syndrome [18]. Western blotting was developed to assess antibodies produced against SARS [19]. The amount of class-specific antibodies against the tick-borne encephalitis virus (TBEV) was evaluated using an RIA-based test [20]. The presence of dengue virus antibodies in human patients has been studied using flow cytometry [21]. To detect and quantify antibodies against multiple viruses such as bovine respiratory syncytial virus, parainfluenza 3 virus, bovine viral diarrhea virus, and bovine herpes virus, Anderson et al. designed and assessed a multiplex assay employing the Luminex platform [22]. Although these techniques have historically been employed in basic and clinical research, peptide microarrays have emerged as cutting-edge since they can identify novel epitopes at an amino acid resolution.

To this end, a thorough understanding of the host immune response to SARS-CoV-2 [23] and its variants will aid in developing diagnostic and prognostic tools as well as vaccines. A thorough characterization of humoral antibody responses to viral proteins requires tools that could unravel the epitopes available on the entire proteome of SARS-CoV-2. Protein microarray-based technologies have been used widely over the past two decades to investigate multiple pathogens [24,25]. It is a fantastic tool for researching the humoral immune response at the amino acid resolution. They have been frequently used to investigate the immunological response to infections and to help in pathogen identification and strain-typing [26]. Peptide microarray is a fast-expanding area that has the potential to be a robust diagnostic platform for a wide range of diseases [27]. Multiple studies in the recent past have used this technology to study the impact of COVID-19 [24,28,29,30,31,32]. While most of these studies gleaned new information on the immunological landscape of the infected populace and found discriminatory epitopes, none of these studies assessed the impact of mutations on immunological topography.

In the current pilot investigation, we studied a cohort of 14 patients using a high-density SARS-CoV-2 peptide microarray. We investigated the humoral immune response to SARS-CoV-2 infection to identify immunogenic areas in the SARS-CoV-2 proteome at a resolution of 2-amino acids. The SARS-CoV-2 proteome was reflected by 4932 peptides printed in duplicate on a chip. The microarray also housed the most relevant mutations from SARS-CoV-2 B.1.1.7 lineage and the Gamma SARS-CoV-2 variant (P.1) apart from other widely circulating SARS-CoV-2 mutations.

In addition to the humoral immune landscape of SARS-CoV-2, the study found severity-specific IgG and IgA epitopes. Additionally, the response to the mutations panel reflected on the effect of mutations on immunoreactivity and may point to their potential utility for identifying infection-causing variants.

## 2. Materials and Methods

### 2.1. Cohort Characteristics and Sample Details

This study was approved by the Institutional Review Board of the Kasturba Hospital for Infectious Diseases and IIT Bombay. Patient consent was waived as leftover samples from the routine analysis were used in the study. The pilot study included 14 patients who had a confirmed diagnosis of COVID-19 using a RT-PCR test. The patients with at least a seven-day interval between symptom onset and sample collection were included in the study. Stratification of patients based on the severity of the symptoms was done by the clinical team at Kasturba Hospital for Infectious Diseases. Parameters such as disease presentation, respiratory distress, SpO_2_ levels, and oxygen supplementation status were used for classifying the patients into severe and non-severe COVID-19 groups (Table S1).

The leftover blood samples anticoagulated with EDTA after blood tests were centrifuged at 2000 rpm for 10 min to separate the plasma and aliquoted in sterile cryovials. The aliquots were then heat-treated at 56 °C for 30 min to inactivate the virus [33]. The heat-treated samples were then transported on ice to IIT Bombay. At IIT Bombay, the samples were divided into sub-aliquots and stored at −80 °C until further use to ensure minimum freeze–thaw cycles.

### 2.2. Peptide Microarray

Epitope-level antibody responses from the patient plasma were deciphered using SARS-CoV-2 whole proteome peptide microarray. The PEPperCHIP^®^ SARS-CoV-2 Proteome Microarray (PEPperPRINT GmbH, Heidelberg, Germany) covers the entire proteome of SARS-CoV-2 isolate Wuhan-Hu-1 (GenBank ID: MN908947.3). The protein sequences of ORF1a/b, Spike, ORF3a, Envelope, Membrane glycoprotein, ORF6, ORF7a, ORF8, Nucleocapsid, and ORF10 were translated into 15 amino acid long peptides with an overlap of 13 amino acids. This resulted in 4883 individual peptides printed in duplicates. As internal controls, the PEPperCHIP^®^ SARS-CoV-2 Microarray contained influenza hemagglutinin (HA) and polio control peptides (108 spots for each control peptide).

Individual patient plasma was thawed on ice and then diluted at 1:100 using staining buffer (PBS with 0.05% Tween20 and 10% Rockland blocking buffer MB 070, pH 7.4). One PEPperCHIP^®^ Peptide Microarray slide was processed per patient plasma for the experiment. PEPperCHIP^®^ Peptide Microarray slides were brought to room temperature, assembled onto the PEPperCHIP^®^ incubation tray (PEPperPRINT GmbH, Germany), and equilibrated using the staining buffer for 15 min. The slides were then incubated with the diluted plasma samples overnight at 4 °C. All the incubations were done on an orbital shaker at 140 rpm unless otherwise stated. On the following day, slides were washed thrice for 1 min each using a washing buffer (PBS with 0.05% Tween20, pH 7.4) while incubating the slide at room temperature. The washing buffer was aspirated entirely after each wash using a micropipette. The slides were then incubated with a mixture of Cy5 conjugated rabbit anti-human IgA (antibodies.com, ABIN901561) and Cy3-conjugated donkey anti-human IgG (Rockland, 609-704-123) antibodies diluted 1:1000 in staining buffer for 45 min at room temperature in the dark. The slides were again washed, as explained earlier. The incubation tray was then disassembled, and the slides were dipped in dipping buffer (1 mM Tris buffer, pH 7.4) to remove PBS residues or dust. The slides were dried under the pressurized nitrogen stream from top to bottom. The slides were then scanned using a Molecular Devices, GenePix^®^ 4000 B scanner, with 33% of the laser intensity for Cy3 and 100% for Cy5 signals.

Next, the slides were processed similarly to acquire signals for the influenza HA control spots for a quality check. The slides were re-assembled onto the incubation tray and equilibrated in staining buffer for 15 min at RT. This was followed by incubation with a PEPperCHIP^®^ Cy5-conjugated anti-HA control antibody diluted 1:2000 in staining buffer for 45 min at RT in the dark. Further washing, drying, and scanning steps were performed, as mentioned earlier.

### 2.3. Data Acquisition

After scanning the chips, their images were acquired in GenePix^®^Pro 7 in .tiff format. The fluorescence or raw intensity and the background intensity of the individual spots were extracted using a PepSlide^®^Analyzer. An R-based script was used for statistical analysis to determine the immunogenic response against the peptides and analyze the severity-based discrimination of the SARS-CoV-2 patients. These steps were carried out on the datasets generated against IgG and IgA antibodies.

### 2.4. Data Analysis

**Figure 1:**
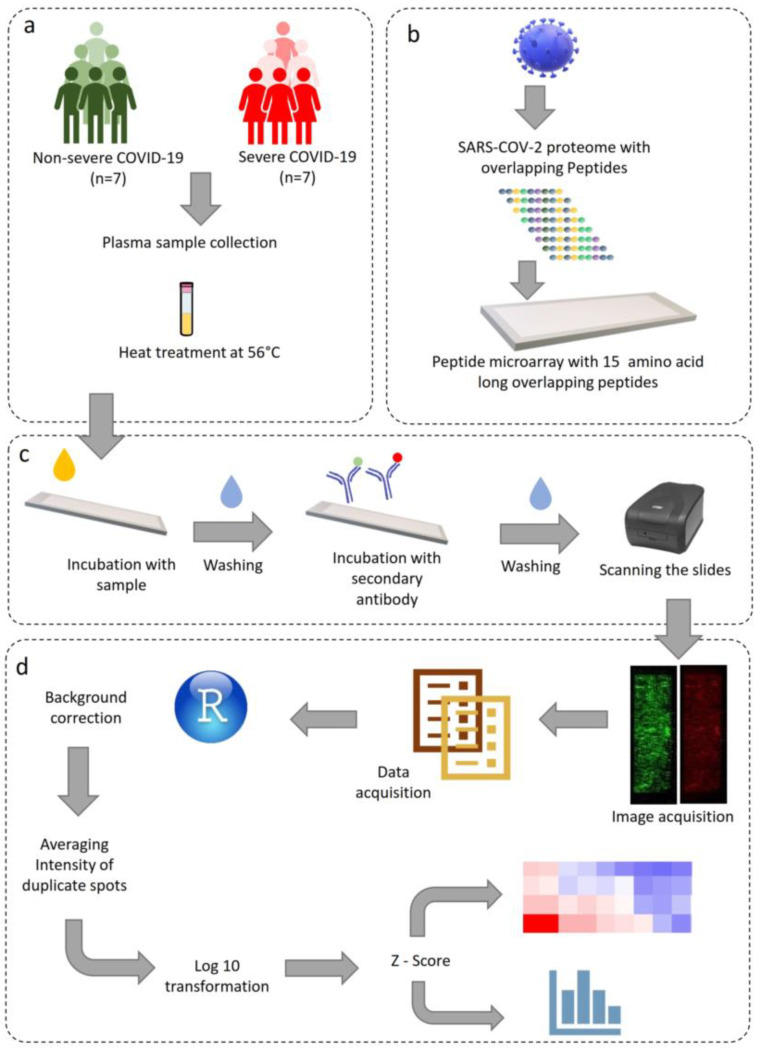
Schematic representation of the study design, microarray protocol, and data analysis. (**a**) Sample acquisition and heat inactivation of virus, (**b**) SARS-CoV-2 whole proteome microarray design, (**c**) microarray staining and image acquisition, (**d**) data analysis pipeline.

The raw intensities of the spots were background-corrected using the “norm-exp” technique to adjust the spot intensities individually with respect to the background. An offset value of 50 was also added so that the weak signals from the features do not get suppressed [34]. The “limma” package of R programming language was used to implement it [35]. Subsequently, the intensities of the duplicate peptides were averaged. Further, to reduce the skewness of the dataset, a logarithmic transformation with a base value of 10 was applied to these values. The mean and standard deviation of intensities of all the peptides across all the samples were used to compute the z-score [32,36,37]. The peptides for which the z-scores exceeded the value of 3 [32] in at least one COVID-19 patient sample were classified as immunogenic. The z-scores were then used to generate heatmaps for visualizing the IgG and IgA response against the SARS-CoV-2 proteome. For determining peptides with discriminatory potential, the response of patients with severe and non-severe COVID-19 was compared using the Mann–Whitney U-test [32]. Peptides with a *p*-value less than 0.05 were considered to be statistically significant. R programming language was used to generate heatmaps, box plots, and dot plots for visualizing different comparisons made during analysis. The study design and the overall workflow is depicted in Figure 1.

## 3. Results

We used the PEPperCHIP^®^ Peptide Microarray slides to study the landscape of B-cell epitopes of IgA and IgG antibodies in COVID-19 patients during acute infection. In total, 14 patients were included in the study; 7 had non-severe COVID-19, and 7 had severe COVID-19 (Table S1). The plasma samples were used to detect the humoral immune response against COVID-19. The PEPperCHIP^®^ Peptide Microarray had 15 amino acid-long 4932 peptides with 13 overlapping peptides, thus providing an epitope resolution as high as two amino acids. During data pre-processing, the data distribution of two samples, one severe (patient ID 69) and the other non-severe (patient ID 33), were skewed. Therefore, these two samples were removed from further data analysis.

### 3.1. Proteome-Wide Immunogenic Response for IgA and IgG

A total of 204 and 676 peptides were identified as immunogenic for IgA and IgG, respectively, based on z-scores. In addition, out of 49 peptides in the mutant panel, 5 and 10 peptides for IgA and IgG respectively, were found to be immunogenic. The peptides were considered immunogenic if the z-score was greater than 3 in any one of the patients.

**IgA Response**: Among the structural proteins, IgA immunoreactivity was found in 17, 5, and 1 peptides in spike, nucleocapsid, and membrane glycoprotein, respectively (Figure S1 and Table S2). No immunogenic response was observed in the envelope protein. Out of the 204 immunogenic peptides identified for IgA, 159 peptides were from the non-structural proteins encoded by ORF1a/b (Figure S1). 41 and 43 IgA-reactive peptides were identified in NSP3 (PLpro) and NSP12 (RdRp). In NSP3, VSELLTPLGIDLDEWSMATYYLFDE (aa81–aa105) epitope showed response for IgA. In NSP 12, we observed response from the N-terminal nidovirus RdRp-associated nucleotidyltransferase domain (RiRAN) and RdRp region. For accessory proteins, 9, 2, 3, and 8 immunogenic peptides were identified in ORF3a, ORF6, ORF7a, and ORF8, respectively (Figure S2a–k and Table S2). However, most of the consecutive regions exhibiting reactivity against IgA were identified in NSP 3, NSP 12, and spike protein (Table S4).

**IgG Response**: A total of 676 peptides were immunoreactive for IgG. There were 478 immunogenic peptides from the NSPs (Figure S1), of which 116 belonged to NSP 3 and 92 belonged to NSP 12. Similarly, 90, 31, 1, and 17 peptides were identified for spike, nucleocapsid, envelope, and membrane glycoprotein, respectively (Figure S2). Among accessory proteins, ORF3a, ORF6, ORF7a, and ORF8 had 27, 5, 12, and 14 immunoreactive peptides, respectively (Figure S3a–k and Table S3).

The top epitopes in the spike protein identified in at least one-fourth of the patients are “CEFQFCNDPFLGVYY” (aa131–aa145 located in the N-terminal domain), “VYYHKNNKSWMESEF” (aa143–aa157, N-terminal domain), “CLIGAEHVNNSYECD” (aa649–aa663, near furin cleavage site), “PSKPSKRSFIEDLLF” (aa809–aa823, near fusion peptide in S2 sub-unit), “ESLIDLQELGKYEQY” (aa1195–aa1209, HR2 region of the S2 sub-unit), “QELGKYEQYIKWPWY” (aa1201–aa1215, HR2 region of the S2 sub-unit) (Table S5). It is interesting to note that these highly immunogenic regions belong to regions other than the RBD. The immunoreactive peptides belonging to the RBD region were identified in a maximum of two patients only. Non-structural proteins 3 and 12 and accessory proteins ORF3a and ORF8 were also associated with strong immunoreactivity in at least one-fourth of the patients. Top immunogenic peptides identified in the nucleocapsid protein were located in the N-terminal domain. Strong immunoreactivity was not observed in envelope and membrane proteins in the majority of the patients. On the other hand we identified VSELLTPLGIDLDEWSMATYYLFDE (aa81–aa105), CSFYPPDEDEEEGDCEEEEFEPS (aa117–139), GDCEEEEFEPSTQYEYG (aa129–aa145), SAALQPEEEQEEDWLDDDS (aa161–aa179) and VLPNDDTLRVEAFEYYH (aa815–aa831) epitopes belonging to the NSP3 as highly reactive for IgG (Table S5).

**Figure 2:**
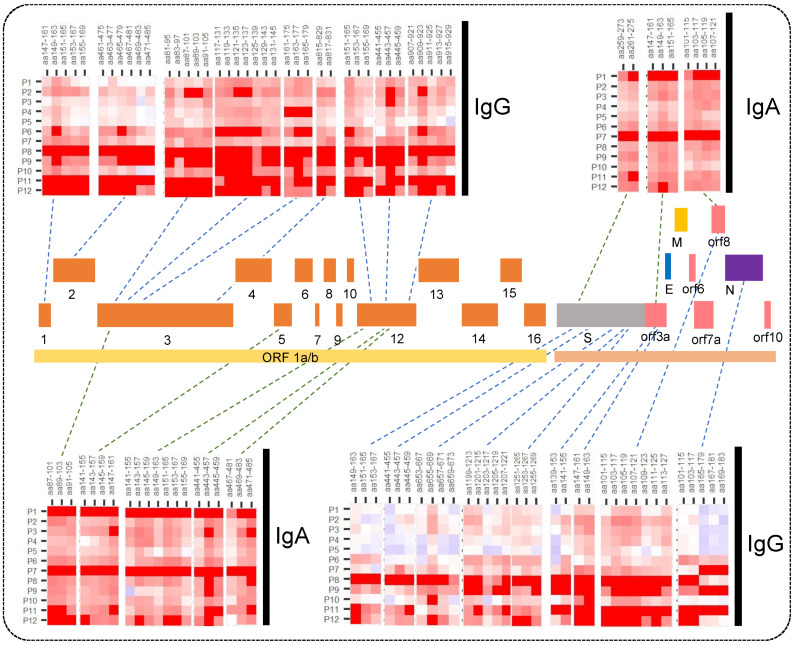
Heatmaps for IgA and IgG response showing major immunogenic regions identified in the SARS-CoV-2 whole proteome microarray. The printed proteome constitutes ORF1a/b polyprotein encoding 16 non-structural proteins (1–10 and 12–16), structural proteins (S, N, E, and M), and the accessory proteins (ORF3a, 6, 7a, 8, and 10).

The major regions in the SARS-CoV-2 proteome immunoreactive to IgA and IgG in at least one-fourth of the patients are shown in Figure 2.

### 3.2. Severity-Based Epitopes

**Figure 3:**
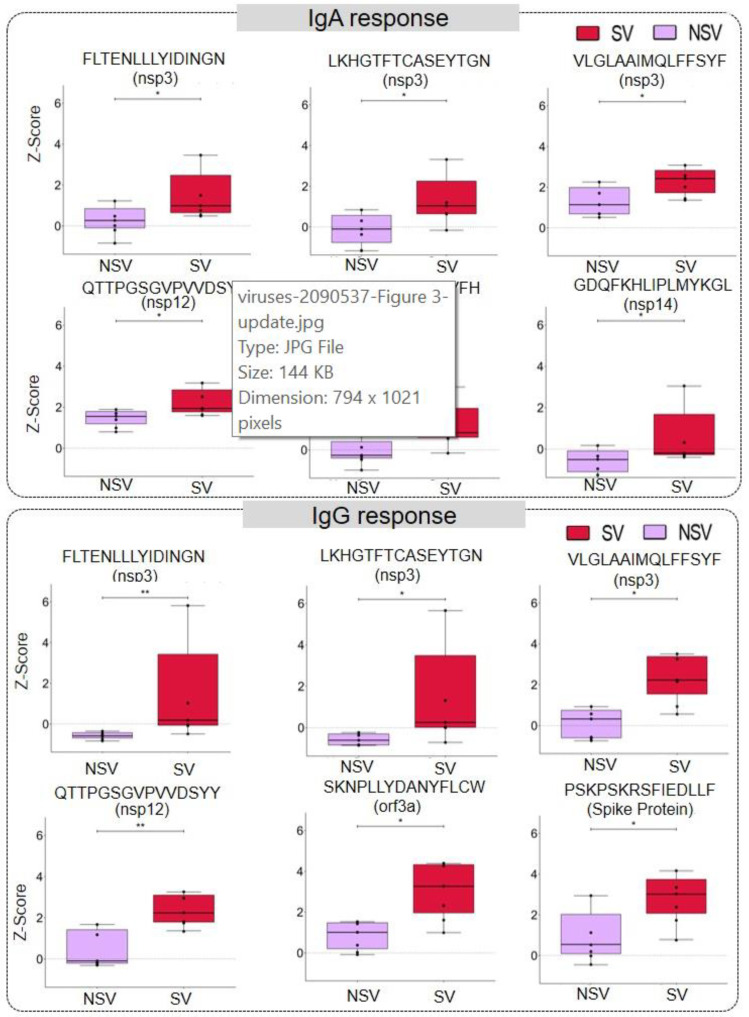
Severity-based discrimination in IgA and IgG response against SARS-CoV-2 peptides. The representative box plot showing IgA and IgG response against a particular peptide in severe (SV) and non-severe (NSV) COVID-19 patients. The significance was calculated using a Mann–Whitney U-Test with a *p*-value < 0.05 (the *p*-values between 0.05 and 0.01 are represented using ‘*’ and those between 0.01 and 0.001 by ‘**’).

We studied severity-based differences in the immune response. The significance of differential response was calculated using the Mann–Whitney U test. We observed that 6 and 319 peptides from ORF1a/b had a significant difference in IgA and IgG reactivity (Tables S6 and S7), respectively, in severe vs. non-severe patients. The response in severe and non-severe COVID-19 in IgA and IgG from some representative peptides has been illustrated in boxplots in Figure 3. Further, for the structural proteins such as spike, nucleocapsid, membrane, and envelope, we identified 57, 12, 8, and 1 peptide, respectively, with significantly differential responses. However, there was no significant difference in IgA response for these structural proteins. Further, we also identified 22, 2, 6, and 13 peptides eliciting severity-based significantly different IgG responses originating from accessory proteins like ORF3a, ORF6, ORF7a, and ORF8, respectively. Some of the discriminatory peptides showed responses both for IgG and IgA (Table 1). Of note, the discriminatory epitopes specific to the severe disease include “ANYFLCWHTNCYDYC” in ORF3a, “EILVTYNCCDDDYFN”, “EVVDKYFDCYDGGCI”, “VLTLDNQDLNGNWYD” and “YRNRDVDTDFVNEFY” in NSP12, “VSELLTPLGIDLDEWSMATYYLFDESGEF”, “VLPNDDTLRVEAFEY”, “CEEEEFEPSTQYEYG”, “FYPPDEDEEEGDCEE”, and “FKWDLTAFGLVAEWF” in NSP3, and “CEFQFCNDPFLGVYY” in spike protein (Tables S8 and S9).

### 3.3. Response to Mutant Peptides

**Figure 4:**
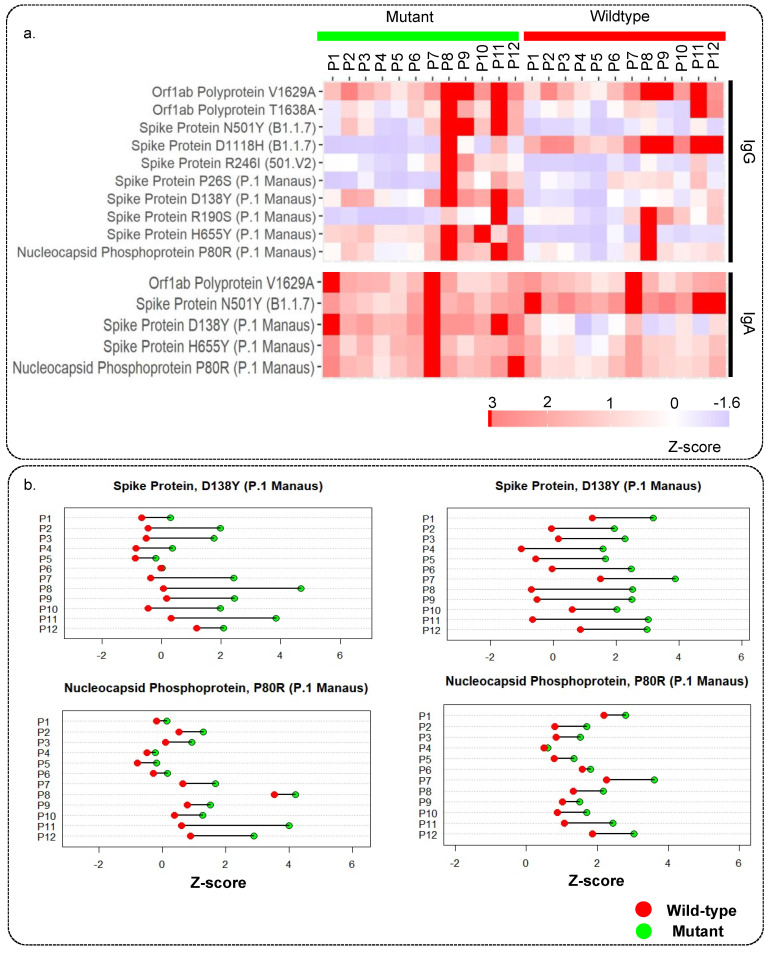
Immune response against mutant peptides for IgG and IgA: (**a**) Heatmap showing IgG and IgA response for peptides with mutations from different SARS-CoV-2 variants and their wildtype counterpart, (**b**) representative dot plots showing the patient-wise comparison of response against mutant and wildtype peptides.

The PEPperCHIP^®^ Peptide Microarray also had the peptides with the mutations from several SARS-CoV-2 variants printed on the slides (Table S10). In particular, the mutant peptides originated from the 501.V2, B1.1.7, and P.1 Manaus variants, as well as some other highly frequent mutations, were included. We observed strong IgG reactivity for 10 of the peptides harbouring mutation, of which 2 originated from ORF1a/b, 7 from the spike, and 1 from nucleocapsid protein. Seven mutant peptides from the spike proteins were associated with B1.1.7, 501.V2, and P.1. Manaus variants. In addition, 5 mutant peptides were reactive for IgA, of which 1 was from ORF1a/b, 3 from the spike, and 1 from nucleocapsid protein. The IgG and IgA response against mutant peptides has been illustrated using a heatmap (Figure 4a). We also looked at differences in the reactivity against the mutant peptide and the corresponding wildtype peptide to see the effect of mutations on the immune response. The patient-wise variation in immunoreactivity to the mutant peptides vs. the corresponding wildtype has been depicted in Figure 4b and Figure S3a–e and Figure S4a,b. We observed that the D138Y mutation in spike protein and the P80R mutation in the nucleocapsid protein increased the immunoreactivity of both IgA and IgG.

## 4. Discussion

SARS-CoV-2 epitope-level proteome-wide analysis was done using peptide microarrays to study the antibody responses in COVID-19 patients. The high peptide-to-peptide overlap of the SARS-CoV-2 proteome array allowed a high-resolution epitope analysis giving a detailed picture of antibody binding patterns, contributing to better characterization of SARS-CoV-2-specific humoral immune responses. For this study, we chose IgA and IgG responses. A previous study [38] using Spike-RBD specific antibodies on patient samples indicated that during the initial days post-infection, that is, 4–10 day, around 88% of the patients were seropositive for IgA, and this response was most robust among IgA, IgM, and IgG. While on the other hand, both IgA and IgG responses were found to be most stable, and 100% of patients showed seroconversion beyond 15 days post-infection. IgM responses have been reported to be erratic [39], indicating that for SARS-CoV-2 it is not a reliable immunological indicator.

**Figure 5:**
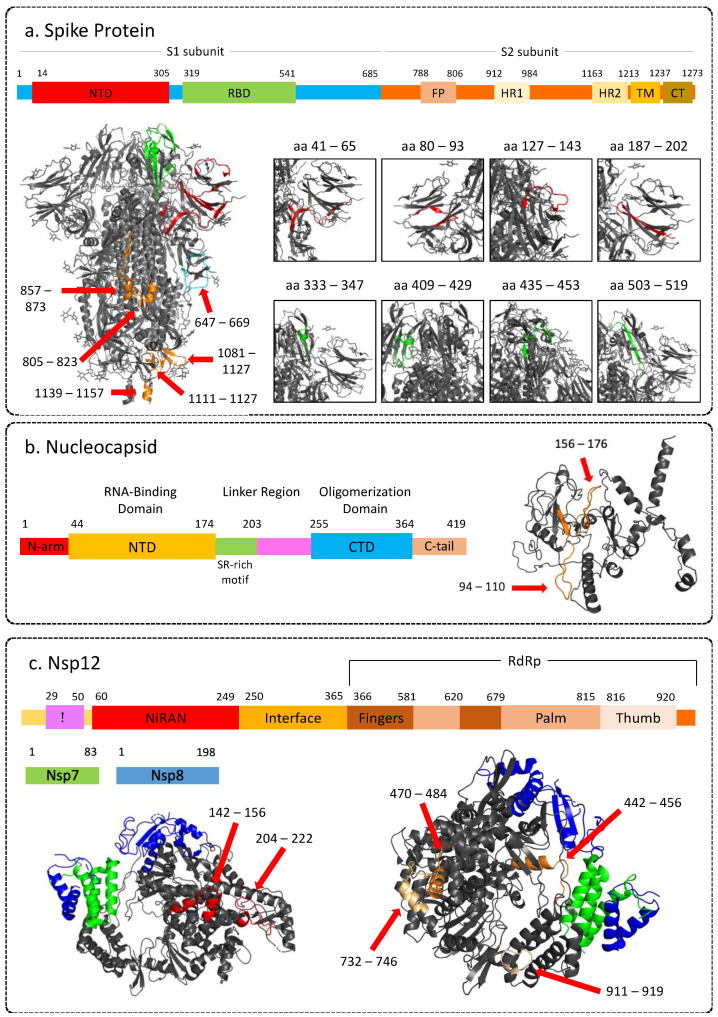
Representative images showing 3D structure of SARS-CoV-2 proteins highlighting immunogenic peptides, along with corresponding linear structure. (**a**) Spike protein (PDB: 6VXX); (**b**) Nucleocapsid (3D structure modeled by Zhang lab using I-Tasser). (**c**) Non-structural protein 12 (RNA-dependent RNA-polymerase) in complex with NSP7 and NSP8 (PDB: 6M71). NTD: N-terminal domain; RBD: Receptor binding domain; FP: Fusion; HR: Heptad repeat; TM: Transmembrane region; CT: Cytoplasmic tail; CTD: C-terminal domain. The 3-D structures of the proteins were visualised using PyMOL (version 2.5.2) [47].

The top immunoreactive regions identified in the spike protein were located in the NTD, HR2 region, and near the furin cleavage site (Figure 5a). Some of these epitopes have also been reported by other groups to be immunogenic [28,31,32]. The receptor binding domain (RBD) region of the spike protein enables viral entry into the cell, therefore, antibodies against spike RBD have been forerunners in neutralising the virus. However, intensities from this region were observed in at most two patients. This could be due to the presence of more conformational than linear epitopes in this region. In addition, antibodies against the NTD, furin cleavage site, and HR2 have also been reported to inhibit viral entry [40,41,42]. We identified the “ESLIDLQELGKYEQY” (aa1195–aa1209) belonging to the conserved HR2 region of the S2 subunit to be highly immunogenic. The S2 subunit is more conserved among the beta-coronaviruses and is less susceptible to non-synonymous mutations [43]. Studies have reported cross-reactive and neutralising antibodies against specific regions in the S2 subunit [44,45,46]. Thus, immunoreactivity of this region can be crucial for protection against other beta-coronaviruses and this can be used to develop effective vaccines.

Two regions in the N-terminal domain of the nucleocapsid phosphoprotein, that is, aa94–aa110 and aa156–aa176, were found to be highly immunoreactive (Figure 5b). Structure of the N-protein shows that part of both of these regions are on the surface and can be easily accessible to antibodies (Figure 5b). These regions have also been reported to be immunodominant by other studies [32,48] and thus can be considered as effective targets for vaccines and serological markers.

Apart from the structural proteins SARS-CoV-2 proteome also has 16 non-structural proteins, which regulate viral replication. Nsp 1 protein is crucial for hijacking the host translational machinery and is, therefore, a crucial virulence factor. Of the epitopes identified against IgG, LKSFDLGDELGTDPYEDFQENWN (aa147–aa169) epitope was identified from the C-terminal domain (CTD) of the Nsp1. A part of these epitopes was also found to be reactive for IgA. Even The region also has been reported to interact with host ribosomes to disarm the IFN-β or RIG-I governed immune responses [49]. This epitope was also reported to show differential responses in mild vs. severe patients in a previous study analyzing the epitope signatures of COVID-19 patients [31]. Therefore, targeting this epitope could be a potential approach for viral clearance.

Nsp2, the LDWLEEKFKEGVEFLRDGWEIVKFI (aa461–aa485) was identified to elicit an IgG response, whereas a part (aa465–aa481) of it was reactive for IgA. However, these epitopes originate from the residual region of the Nsp2 protein. The Nsp2 protein has an N-terminus and a residual domain. However, understanding the structure and function of disordered regions of NSP2 is in a nascent stage and remains elusive. Therefore, further studies targeting the disordered regions are required. It is the N-terminus domain that binds to the nucleic acids [50]. We also identified two immunogenic peptides from the N-terminus RGVYCCREHEHEIAW (aa59–aa73) and CCREHEHEIAWYTER (aa63–aa77) that elicited a response in at least three patients with significantly different severe vs. non-severe COVID-19. Schwarz et al. also observed a similar trend in comparing patients with severe symptoms to those with mild [31]. In another study by Heidepriem et al., the above-mentioned peptides showed a high IgA response in some of the patients during the early phase of infection. However, the IgG response remained low in comparison to IgA and IgM responses [51].

NSP 3, or papain-like protease, is critical for viral replication and suppression of host responses. It is responsible for hydrolyzing the polyprotein pp1a into Nsp 1, 2, and 3. It interferes with the immune response in the host, in particular, the interferon and NF-κB pathways [52]. NSP 3 is a multidomain protein and is divided into 10 domains. We observed reactivity against domains like ubiquitin-like domain 1, hypervariable region, macrodomain I, and macrodomain II. Interestingly, we found the highly reactive regions originating from ubiquitin-like domain 1, hypervariable region, and ubiquitin-like domain 2 regions. This hypervariable region is a Glu-rich region with probable interaction with other proteins like Nsp6, 8, and 9 and the NAB–βSM–TM1 of Nsp3 [53]. However, the exact role of this region remains unknown. Therefore, the potential role of HVR reactivity also remains elusive. Another highly reactive region was identified in ubiquitin-like 1 region of Nsp3; VSELLTPLGIDLDEWSMATYYLFDE (aa81–aa105). This region has been speculated to mimic the host ubiquitin enzymes and thus help in escaping the host degradation mechanism. Moreover, the regions have also been reported to interact with ssRNA, thereby indicating its role in viral RNA replication and processing [54]. This region also showed discriminatory potential based on disease severity. We also identified the reactive peptides from macrodomain I (mac1) and papain-like protease (PLpro) regions. Whereas the mac1 domain has the ADP-ribosyl hydrolyase activity by regulating the host-mediated antiviral adenosine diphosphate-ribosylation signaling. PLpro has proteolytic, deubiquitinating, and deISGylating activity. These immune reactive regions can be targeted for inhibiting the viral genome replication and transcription.

Nsp 12 protein consists of N-terminal nidovirus RdRp-associated nucleotidyltransferase domain (RiRAN), interface, and the RdRp domain. We identified epitopes originating from all three domains. LKEILVTYNCCDDDYFN (aa143–aa159) and NCCDDDYFNKKDWYDFVEN (aa151–169) were IgA and IgG-reactive epitopes belonging to the RiRAN domain. In addition, we also identified peptide DNQDLNGNWYDFGDF (aa209–aa223) to be reactive for IgG. This epitope was also reported as one of the top reactive epitopes in another study mapping the epitope response [30]. Further, we identified FFFAQDGNAAISDYDYYRY (aa441–aa459), QLLFVVEVVDKYFDCYDGGCI (aa469–aa489), LYYDSMSYEDQDALFAY (aa515–aa531) YRNRDVDTDFVNEFYAY (aa733–aa749) and TNDNTSRYWEPEFYEAMYTPH (aa909–929) epitopes originating from RdRp domain to be reactive against IgG with some peptides forming these epitopes also showing reactivity for IgA (Figure 5c). In addition, aa441–aa459, aa515–aa531, aa733–aa749, and aa911–aa929 were found to elicit a discriminant response based on severity. Since Nsp12 is the core of RTC, it has been reported to form complexes with other non-structural proteins during viral genome replication and transcription [52]. Therefore, understanding the immune response against Nsp12 becomes crucial for developing antiviral therapies.

Although many immunogenic peptides were identified in other non-structural proteins like Nsp4, 5, 6, 7, 8, 9, 10, 13, 14, 15, and 16, there were no immunodominant regions identified. Most of the reactivity was patient-specific and concentrated in patients 8 and 11 for IgG and patients 1 and 7 for IgA. Of particular interest was the peptides like SVGFNIDYDCVSFCY (aa147–aa161) from Nsp5 (main proteases) which elicited a response in both IgG and IgA and severity-based discrimination in terms of IgG response. Another noteworthy peptide was LGVYDYLVSTQEFRY (aa239–aa253) which showed reactivity for IgG. A previous study has also reported this peptide as one of the highly reactive peptides for IgG [30]. In addition, the discriminatory potential of this peptide has been observed [31]. However, the trend reported in the earlier study is not similar. Considering the current cohort size a definitive comparison is difficult. Nonetheless, despite the conserved sequences, these proteins can be explored further as potential therapeutic targets due to their role in the viral life cycle and host invasion or for developing prognostic assays. Further studies are required to study the protective role of antibodies against non-structural proteins.

ORF8 protein has been reported to be a secreted protein detectable in sera of COVID-19 patients and has been shown to elicit immune responses during the early stages of SARS-CoV-2 infection [32]. Hachim and colleagues have identified acute and convalescent antibody responses against ORF8 protein suggesting the possible use of ORF8 as a sensitive and specific method for detection of both early and late SARS-CoV-2 infection [55]. The protective role of anti-ORF8 antibodies, however, remains unclear. It has been reported that SARS-CoV-2 mediates the downregulation of MHC-I as a way of immune evasion [56]. Thus, it can be speculated that neutralization of ORF8 may salvage potential immune evasion by ORF8.

Immunoreactivity of IgGs and IgAs for several epitopes was higher in the severe cohort as compared to the non-severe cohort, as can be observed from Figure 3. Whereas, for some epitopes majorly from ORF1a/b, immunogenicity was higher in the non-severe group. The course of the disease and the outcomes thereof can be attributed to the intensity of immune response to various immunogenic regions in the SARS-CoV-2 proteome. A couple of studies have associated SARS-CoV-2 antibodies with disease severity and survivability [32,37,57]. However, the sample sizes were too small to comment on whether this difference in immunogenicity can be related to the severity and survivability of the patients.

The SARS-CoV-2 proteome microarray also had a panel of peptides with mutations associated with the common variants of concern. Immunoreactivity to several mutations, including those from the P.1 and B.1.1.7 lineages, was observed, indicating either infection with these variants or that these mutations do not affect the immunogenicity of the corresponding wildtype peptides. The information about which variant caused the infection in patients from whom the samples were collected is not known. However, the samples in the study were collected from March to April 2021 during which both P.1 (alpha) and B.1.1.7 (beta) variants were circulating and the B.1.615 (delta) variant was on the rise. The effect of mutations on the immunogenicity of epitopes can also be comprehended using the mutant panel. Both IgG and IgA response to the peptide “QSYGFQPTNGVGYQP” increased significantly with N501Y mutation of the B1.1.7 linage. The mutation N501Y affects the conformation of the receptor binding domain of the spike protein [58]. Thus, it can be concluded that this mutation increases the immunogenicity of the spike protein. Whereas some mutations decreased the immunogenicity of peptides for both IgG and IgA, such as K417T and D138Y, both of which are specific to P.1 lineage. K417T is another key mutation in the spike RBD and is involved in interaction with the ACE2 receptor, while D138Y lies in the highly immunogenic region of the spike NTD. Thus, it can be inferred that these mutations decrease the immunogenicity of the spike protein. Even so, despite mutations, by and large, most peptides remained immunogenic.

Finally, being a pilot study, we conducted the test on a small cohort of patients. Further work is currently being planned to study an extensive cohort of patients with a longitudinal follow-up. This may lead to a better understanding of disease progression and aggressiveness as well as the impact of variant-specific mutations on the immune signature of the patient.

## 5. Conclusions

In conclusion, the humoral response to SARS-CoV-2 proteins was studied, revealing several immunogenic regions and severity-based epitopes in the viral proteome at amino acid resolution. Immunogenic regions in the SARS-CoV-2 proteome can be associated with disease severity and survivability, and they have the potential to be used as serological markers for prognosis and disease stratification. In addition, the response to common mutations was also studied using the panel of mutant peptides. We found differences in immunoreactivity in response to some mutant peptides as compared to the corresponding wildtype peptides. This highlights the changes in the immunoreactivity due to mutations and mechanisms of immune escape by the variants.

## Figures and Tables

**Table 1:** Viral protein-wise list of epitopes that seroconverted from IgA to IgG in patients. The IgA/IgG epitopes that could discriminate between severe and non-severe forms of the disease are indicated.

Protein Name	Seroconverted Epitope	Discrimination Status
NSP1	GEIPVAYRKVLLRKNGN	Non-significant ^α^
NSP1	LKSFDLGDELGTDPYEDFQENWN	IgG ^β^
NSP2	GAYTRYVDNNFCGPDGYPLEC; NIVGDFKLNEEIAII; LDWLEEKFKEGVEFLRDGWEIVKFI	IgG
NSP3	QPVSELLTPLGIDLDEWSMATYYLFDESGEFKL; SAALQPEEEQEEDWLDDDSQQ	Non-significant
NSP3	MYCSFYPPDEDEEEGDCEEEEFEPSTQYEYGTEDDYQ; RTNVYLAVFDKNLYD; GIKIQEGVVDYGARFYFYT; FYVLPNDDTLRVEAFEYYH; TLRVEAFEYYHTTDPSFLGRY; IELKFNPPALQDAYY; AGEAANFCALILAYC; GVVCTEIDPKLDNYY; TFFPDLNGDVVAIDY; ITEEVGHTDLMAAYV; SYFAVHFISNSWLMWLI	IgG
NSP3	TLEETKFLTENLLLYIDINGN; LKHGTFTCASEYTGN; VLGLAAIMQLFFSYF	IgG/IgA ^λ^
NSP4	DTCFANKHADFDTWF; FATSACVLAAECTIF; EGSVRVVTTFDSEYCRH; VSFSTFEEAALCTFLLN	IgG
NSP5	QVTCGTTTLNGLWLDDVVYCPRH; LNGSCGSVGFNIDYDCVSFCYMHHMEL; LAWLYAAVINGDRWF; NGRTILGSALLEDEF; ILGSALLEDEFTPFDVVRQ	IgG
NSP6	LVQSTQWSLFFFLYE; MFLARGIVFMCVEYC; RGIVFMCVEYCPIFF; CLLNRYFRLTLGVYDYL; LTLGVYDYLVSTQEFRY	IgG
NSP8	NTCDGTTFTYASALWEI	IgG
NSP9	TTQTACTDDNALAYY	IgG
NSP9	VLGSLAATVRLQAGN	Non-significant
NSP12	TGTSTDVVYRAFDIYND; FQEKDEDDNLIDSYFVV;TKYTMADLVYALRHFDEGNCD; QTVKPGNFNKDFYDF; FFFAQDGNAAISDYDYYRYNL; ARLYYDSMSYEDQDALFAY; RLYECLYRNRDVDTDFVNEFYAY; HFSMMILSDDAVVCF	IgG
NSP12	NCDTLKEILVTYNCCDDDYFNKKDWYDFVEN; ADKYVRNLQHRLYECLY; FCSQHTMLVKQGDDYVYLPYP; MLTNDNTSRYWEPEFYEAMYTPHTVLQ	Non-significant
NSP12	QTTPGSGVPVVDSYY; PLTKHPNQEYADVFHLYLQYI	IgG/IgA
NSP13	ACIRRPFLCCKCCYD; DVTDVTQLYLGGMSYYC; FNAIATCDWTNAGDYIL; TQTVDSSQGSEYDYVIF	IgG
NSP13	VNALPETTADIVVFDEISM; CPAEIVDTVSALVYD	Non-significant
NSP14	SDTYACWHHSIGFDYVYNPFMIDVQQWGF; WGFTGNLQSNHDLYC; HECFVKRVDWTIEYPII	IgG
NSP14	EYPIIGDELKINAAC; AQPCSDKAYKIEELFYS; IEELFYSYATHSDKFTD; ATHSDKFTDGVCLFWNC	Non-significant
NSP15	NLGVDIAANTVIWDY; TVFFDGRVDGQVDLFRN; QMEIDFLELAMDEFIERYK; LAMDEFIERYKLEGYAFEH; KLEGYAFEHIVYGDFSH; LAKRFKESPFELEDF; GSSKCVCSVIDLLLDDFVEIIKS; VKVTIDYTEISFMLW	IgG
NSP16	PTGTLLVDSDLNDFV; TEHSWNADLYKLMGHFAWW	Non-significant
NSP16	PIQLSSYSLFDMSKF	IgG
Spike	TQDLFLPFFSNVTWF; CEFQFCNDPFLGVYY; FRVYSSANNCTFEYV; KNLREFVFKNIDGYFKI; AGCLIGAEHVNNSYECDIP; DPLQPELDSFKEELDKYFK; LNESLIDLQELGKYEQY;DLQELGKYEQYIKWPWYIW; KGCCSCGSCCKFDEDDSEP	IgG
Spike	DTTDAVRDPQTLEILDI	Non-significant
Membrane Glycoprotein	LEQWNLVIGFLFLTW	IgG
Nucleocapsid	WFTALTQHGKEDLKF; IRGGDGKMKDLSPRWYFYY; GSSRGTSPARMAGNGGDAALALLLLDR	IgG
Orf3a	YSHLLLVAAGLEAPFLYLY; WKCRSKNPLLYDANYFLCW; YDANYFLCWHTNCYDYCIPYN; VKDCVVLHSYFTSDYYQLY	IgG
Orf6	IMRTFKVSIWNLDYI	IgG
Orf7a	ILFLALITLATCELYHYQECVRG	IgG
Orf8	KLGSLVVRCSFYEDFLEYHDVRVVLDF	IgG

α indicates seroconverted epitopes that do not discriminate severe and non-severe forms of the disease. β indicates IgG isotype of seroconverted epitopes that could discriminate severe and non-severe forms of the disease. λ indicates both IgG and IgA isotypes of seroconverted epitopes could discriminate severe and non-severe forms of the disease.

## Data Availability

The microarray raw and processed data will be available upon request.

## Supplementary Materials

The following supporting information can be downloaded at: https://www.mdpi.com/article/10.3390/v15010248/s1, Figure S1: Heatmap showing IgG response for peptide against ORF1a/b non-structural protein NSP1, NSP2 (a), NSP3 (b); NSP4, NSP5 (c), NSP6, NSP7, NSP8, NSP9 (d), NSP10, NSP12 (e), NSP13 (f), NSP14, NSP15 (g), NSP14, and NSP15 (h), accessory proteins like ORF3a, ORF7a, ORF6, and ORF10 (i), and structural proteins envelope (h), membrane (h), spike (j) and nucleocapsid phosphoprotein (k). Figure S2: Heatmap showing IgA response for peptide against ORF1a/b non-structural protein NSP1, NSP2 (a), NSP3 (b); NSP4, NSP5 (c), NSP6, NSP7, NSP8, NSP9 (d), NSP10, NSP12 (e), NSP13 (f), NSP14, NSP15 (g), NSP14, and NSP15 (h), accessory proteins like ORF3a, ORF7a, ORF6, and ORF10 (i), and structural proteins envelope (h), membrane (h), spike (j) and nucleocapsid phosphoprotein (k). Figure S3: Representative dot plots showing patient-wise comparison of IgG response against mutant and wildtype peptides (a) i) Orf1a/b Polyprotein, T1638A ii) Spike Protein, D138Y (P.1 Manaus); (b) i) Spike Protein, D111H (B1.1.7) ii) Spike Protein, H655Y (P.1 Manaus); (c) i) Nucleocapsid Phophoprotein, P80R (P.1 Manaus) ii) Orf1a/b Polyprotein, V1629A; (d) i) Spike Protein, R190S (P.1 Manaus) ii) Spike Protein, R246I (501.V2); (e) i) Spike Protein, N501Y(B1.1.7) ii) Spike Protein, P26S (P.1 Manaus); Figure S4: Representative dot plots showing patient-wise comparison of IgA response against mutant and wildtype (a) i) Spike Protein, D138Y (P.1 Manaus), ii) Spike Protein, H655Y (P.1 Manaus); (b) i) Nucleocapsid Phophoprotein, P80R(P.1 Manaus), ii) Orf1a/b Polyprotein, V1629A, iii) Spike Protein, N501Y(B1.1.7). Table S1: Clinical Characteristics of patients. Table S2: IgA reactive peptides from the SARS-CoV-2 proteome. Table S3: IgG reactive peptides from SARS-CoV-2 proteome. Table S4: Epitopes identified based on IgA response against SARS-CoV-2 proteins. Table S5: Epitopes identified based on IgG response against SARS-CoV-2 proteins. Table S6: Severity-based discrimination in IgA response against SARS-CoV-2 peptides. Table S7: Severity-based discrimination in IgG response against SARS-CoV-2 peptides. Table S8: Discriminatory epitope for IgA. Table S9: Discriminatory epitopes for IgG. Table S10: List of peptides with mutations.
