# Supplementary material for: Humoral Immune Response Profile of COVID-19 Reveals Severity and Variant-Specific Epitopes: Lessons from SARS-CoV-2 Peptide Microarray
Source: Viruses. 2023 Jan 15;15(1):248. doi: 10.3390/v15010248 (PMC9866125; doi:10.3390/v15010248)

Supplementary Figure S1a: Heatmap showing IgA response for peptide against orf1ab non-structural protein (nsp1, nsp2). The amino acid sequence of the corresponding protein is denoted as "aa1-15" and the COVID-19 patients included in the study are presented as p1-p12. Immunogenic response against the peptide was visualised using Z-score of fluorescence intensity. Peptides with a Z-score greater than 3 in at least one COVID-19 patients was defined as immunogenic.

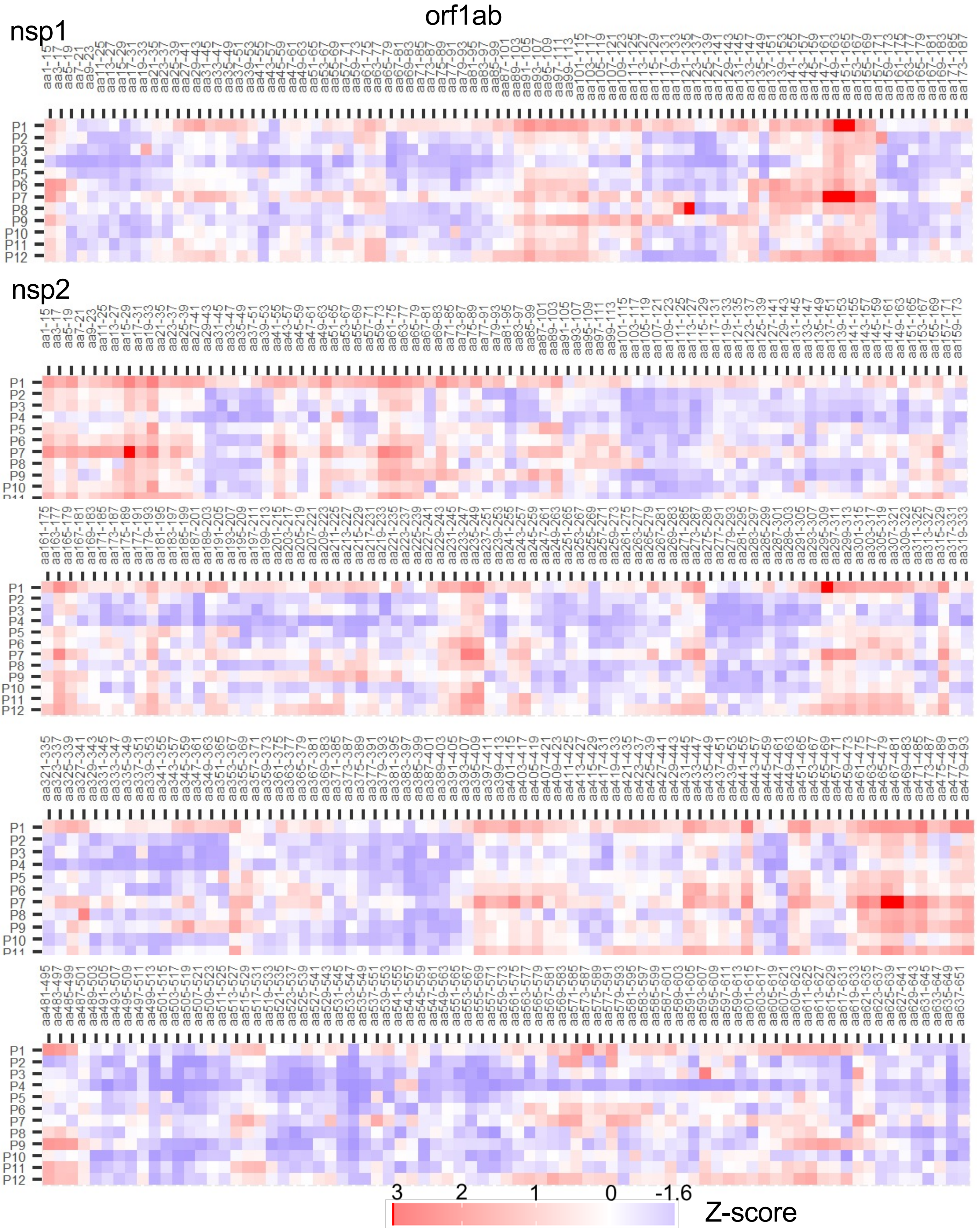

Supplementary Figure S1b: Heatmap showing IgA response for peptide against orf1ab non-structural protein (nsp3). The amino acid sequence of the corresponding protein is denoted as “aa1-15” and the COVID-19 patients included in the study are presented as p1-p12. Immunogenic response against the peptide was visualised using Z-score of fluorescence intensity. Peptides with a Z-score greater than 3 in at least one COVID-19 patients was defined as immunogenic.

nsp3

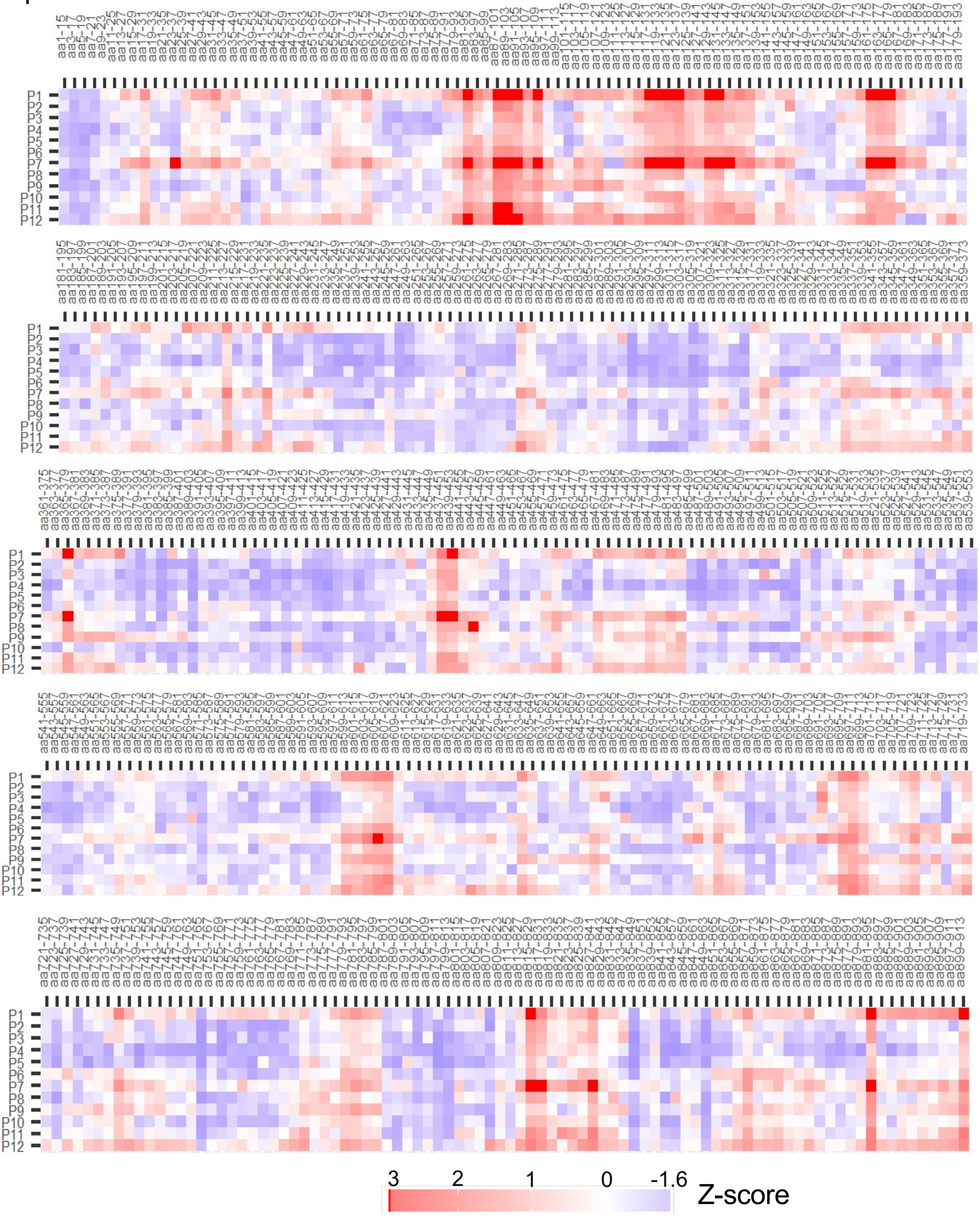

Supplementary Figure S1b contd…nsp3

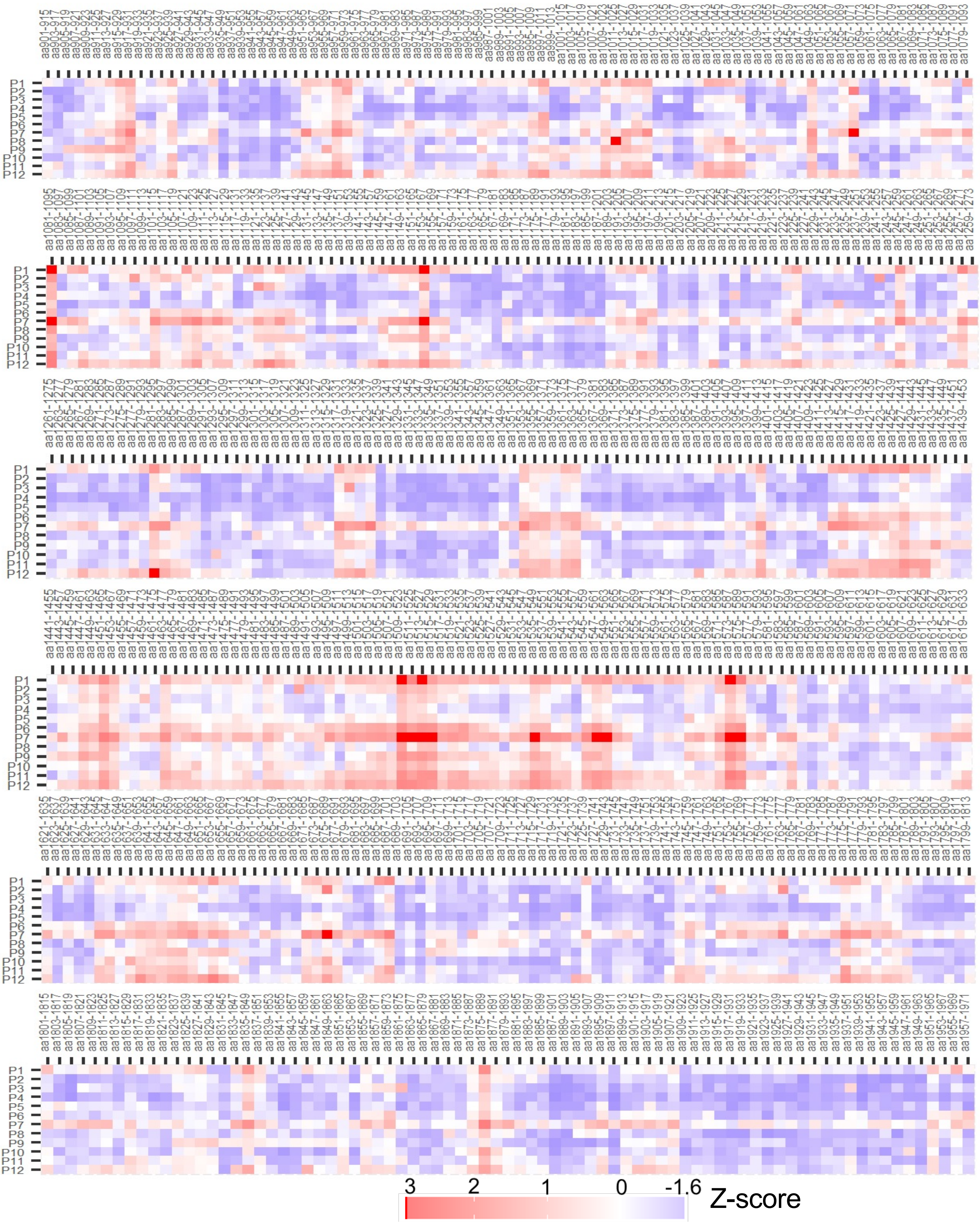

Supplementary Figure S1c: Heatmap showing IgA response for peptide against orf1ab non-structural protein (nsp4, nsp5). The amino acid sequence of the corresponding protein is denoted as "aa1-15" and the COVID-19 patients included in the study are presented as p1-p12. Immunogenic response against the peptide was visualised using Z-score of fluorescence intensity. Peptides with a Z-score greater than 3 in at least one COVID-19 patients was defined as immunogenic.

nsp4

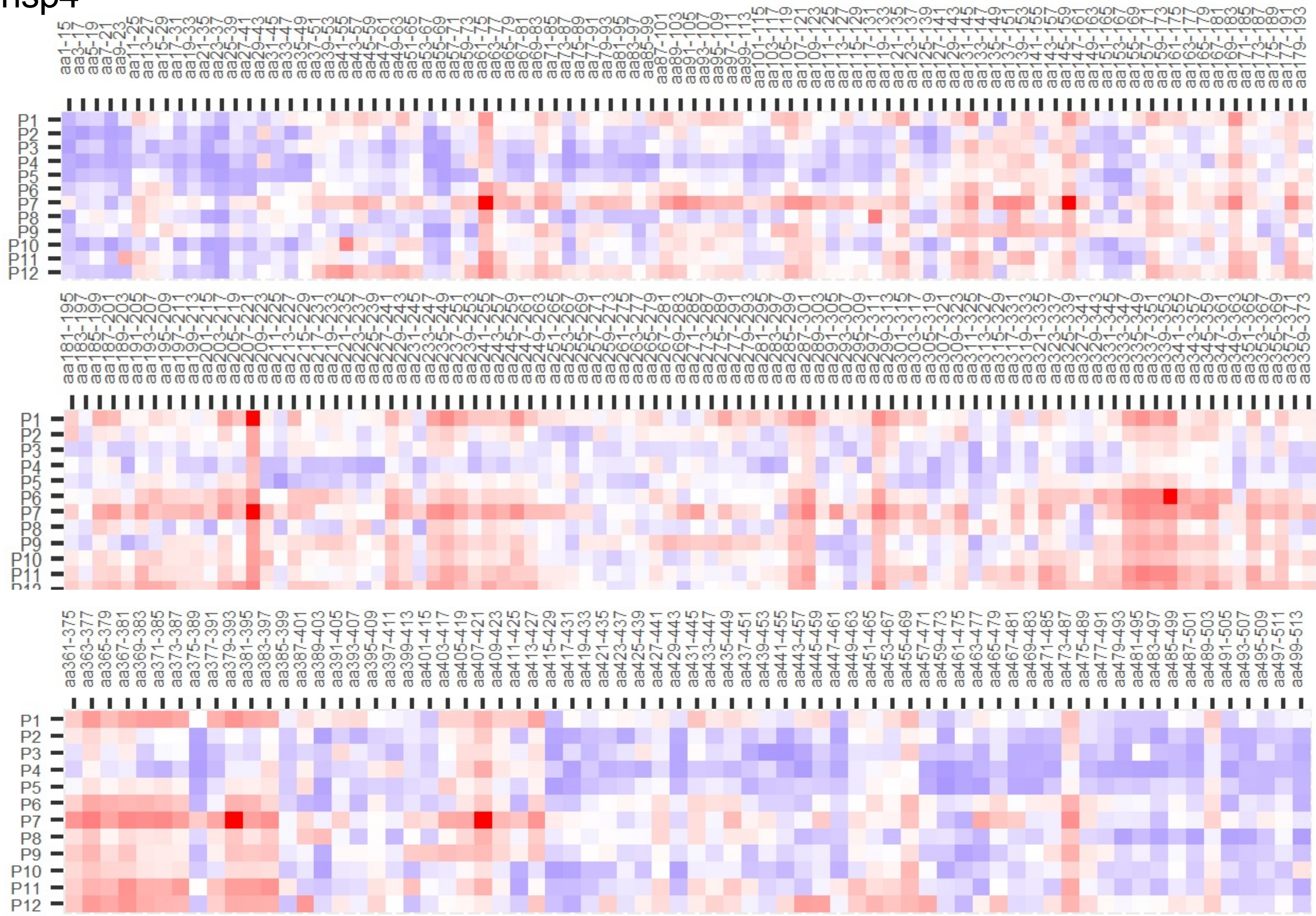

nsp5

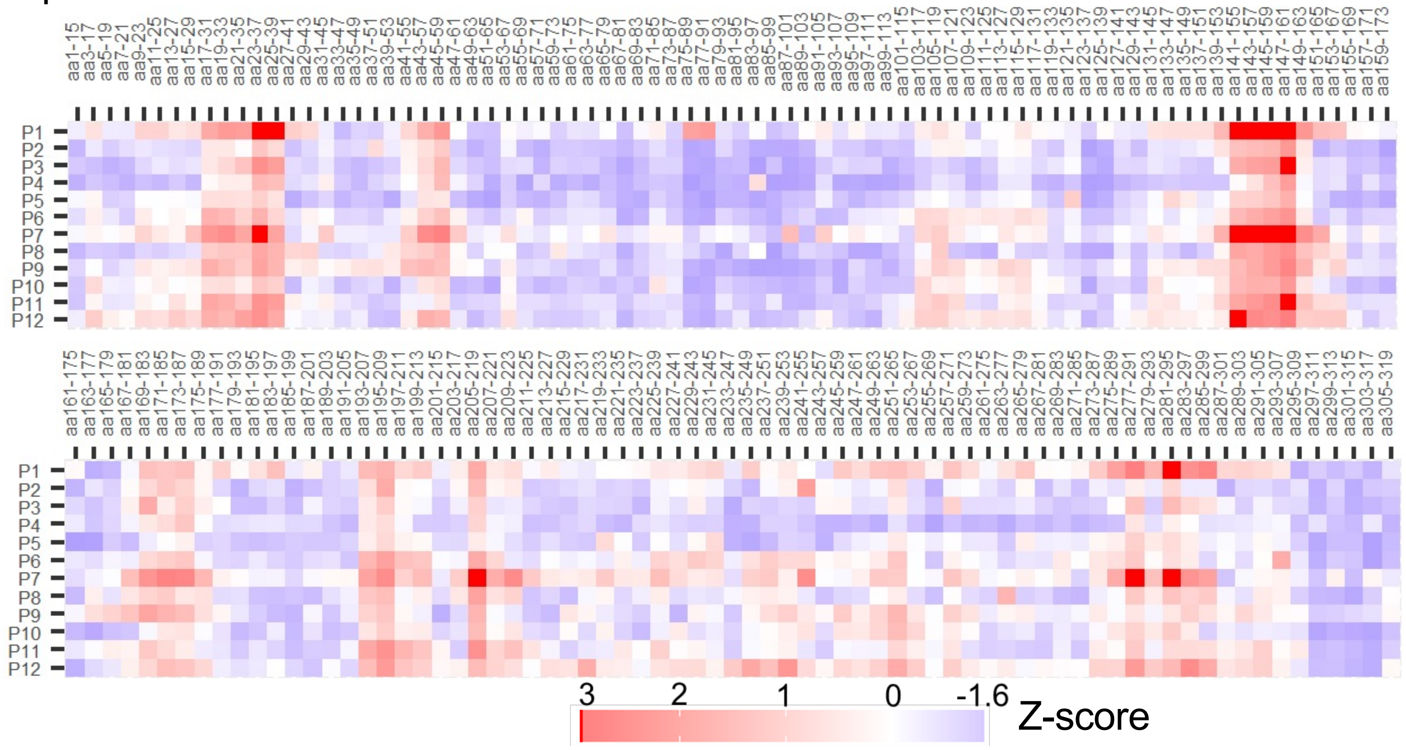

Supplementary Figure S1d: Heatmap showing IgA response for peptide against orf1ab non-structural protein (nsp6, nsp7, nsp8, nsp9). The amino acid sequence of the corresponding protein is denoted as "aa1-15" and the COVID-19 patients included in the study are presented as p1-p12. Immunogenic response against the peptide was visualised using Z-score of fluorescence intensity. Peptides with a Z-score greater than 3 in at least one COVID-19 patients was defined as immunogenic.

nsp6

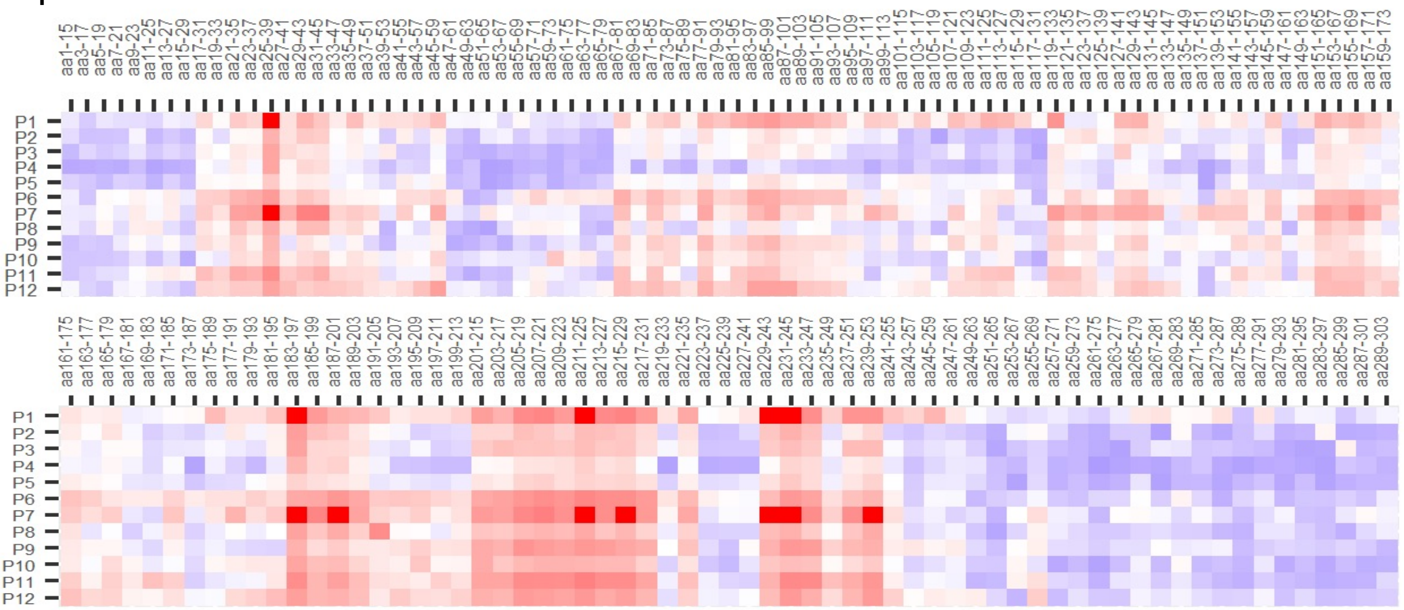

nsp7

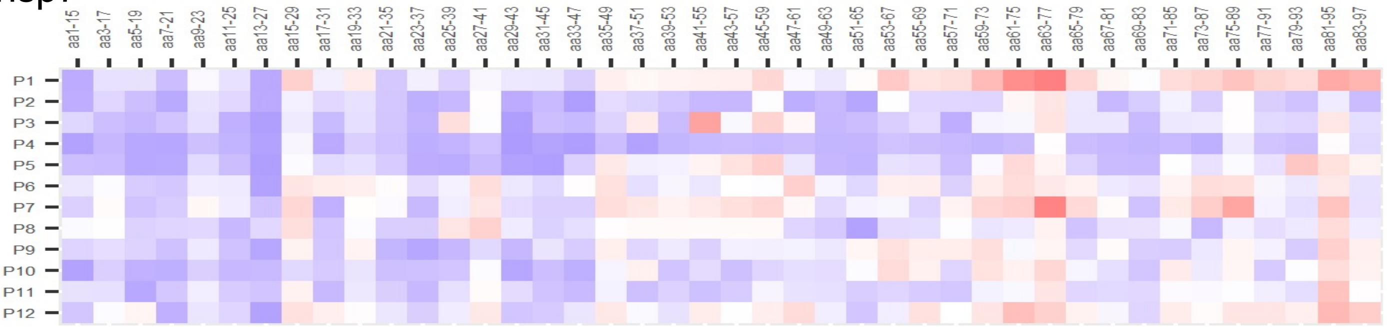

nsp8

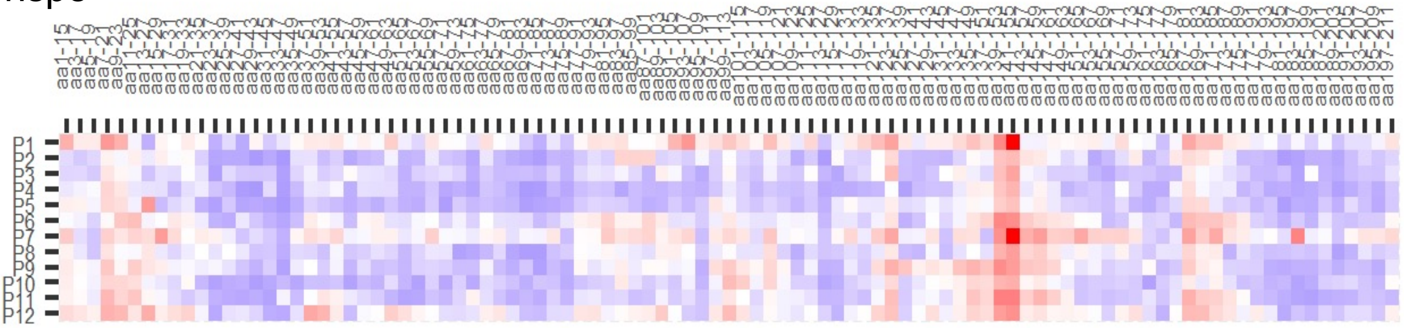

nsp9

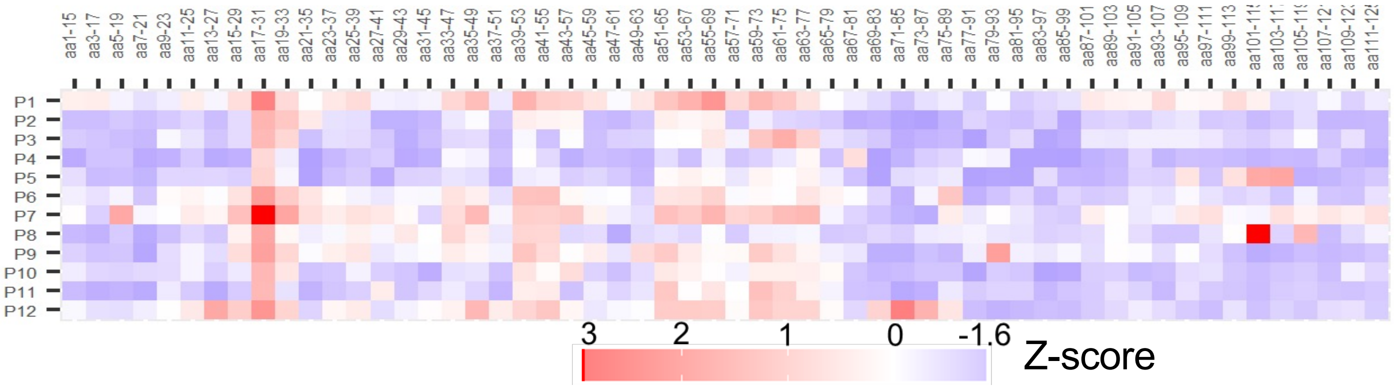

Supplementary Figure S1e: Heatmap showing IgA response for peptide against orf1ab non-structural protein (nsp10, nsp12). The amino acid sequence of the corresponding protein is denoted as "aa1-15" and the COVID-19 patients included in the study are presented as p1-p12. Immunogenic response against the peptide was visualised using Z-score of fluorescence intensity. Peptides with a Z-score greater than 3 in at least one COVID-19 patients was defined as immunogenic.

nsp10

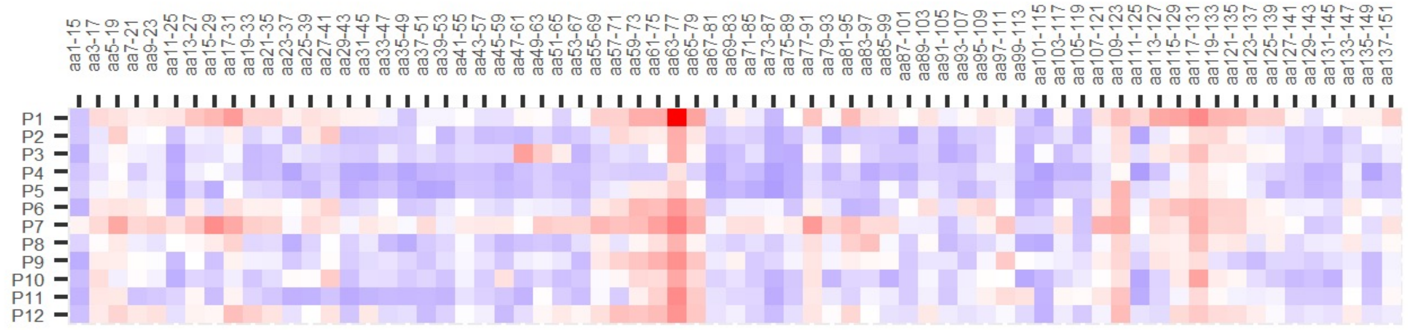

nsp12

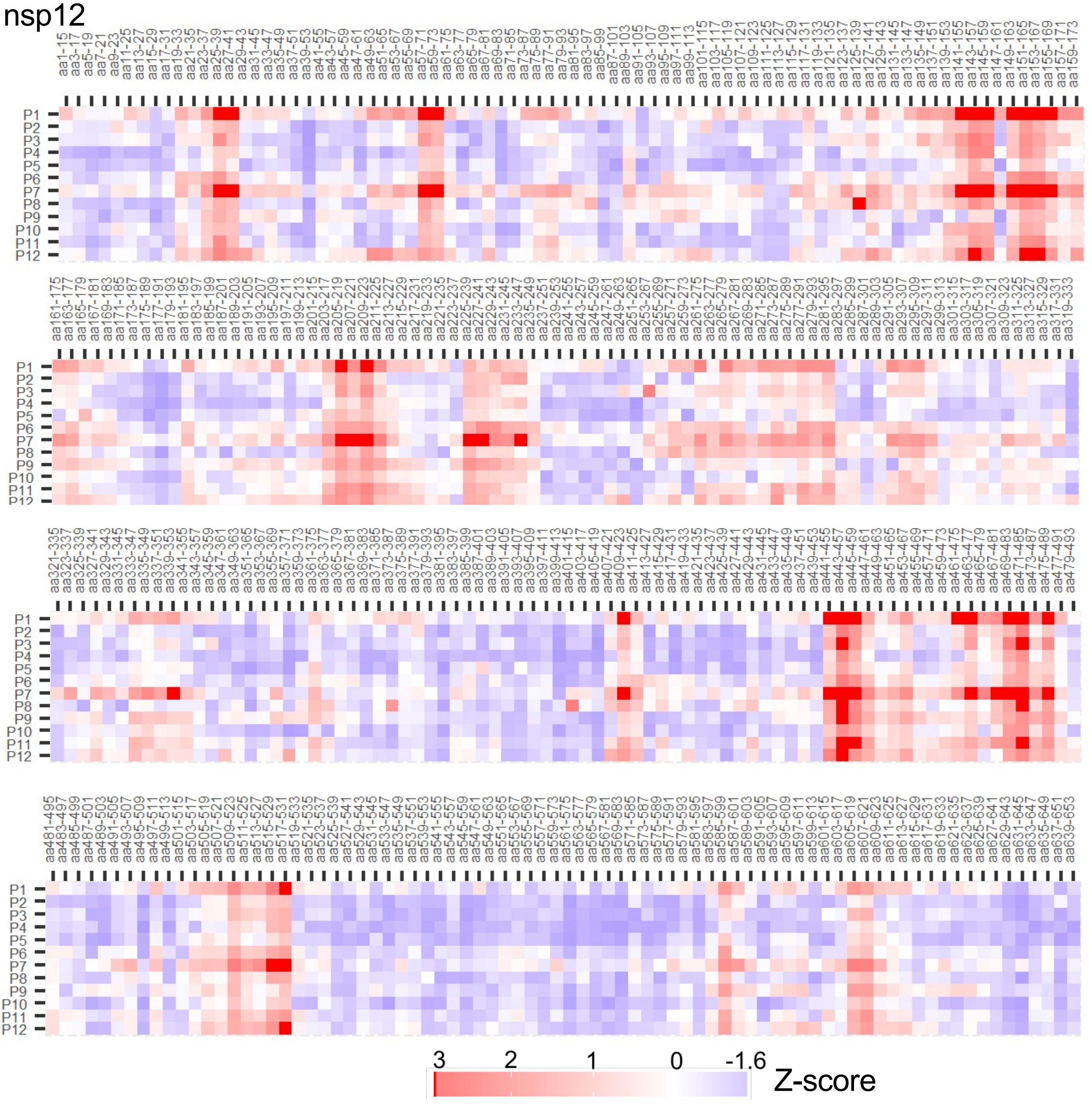

Supplementary Figure S1e contd…

nsp12

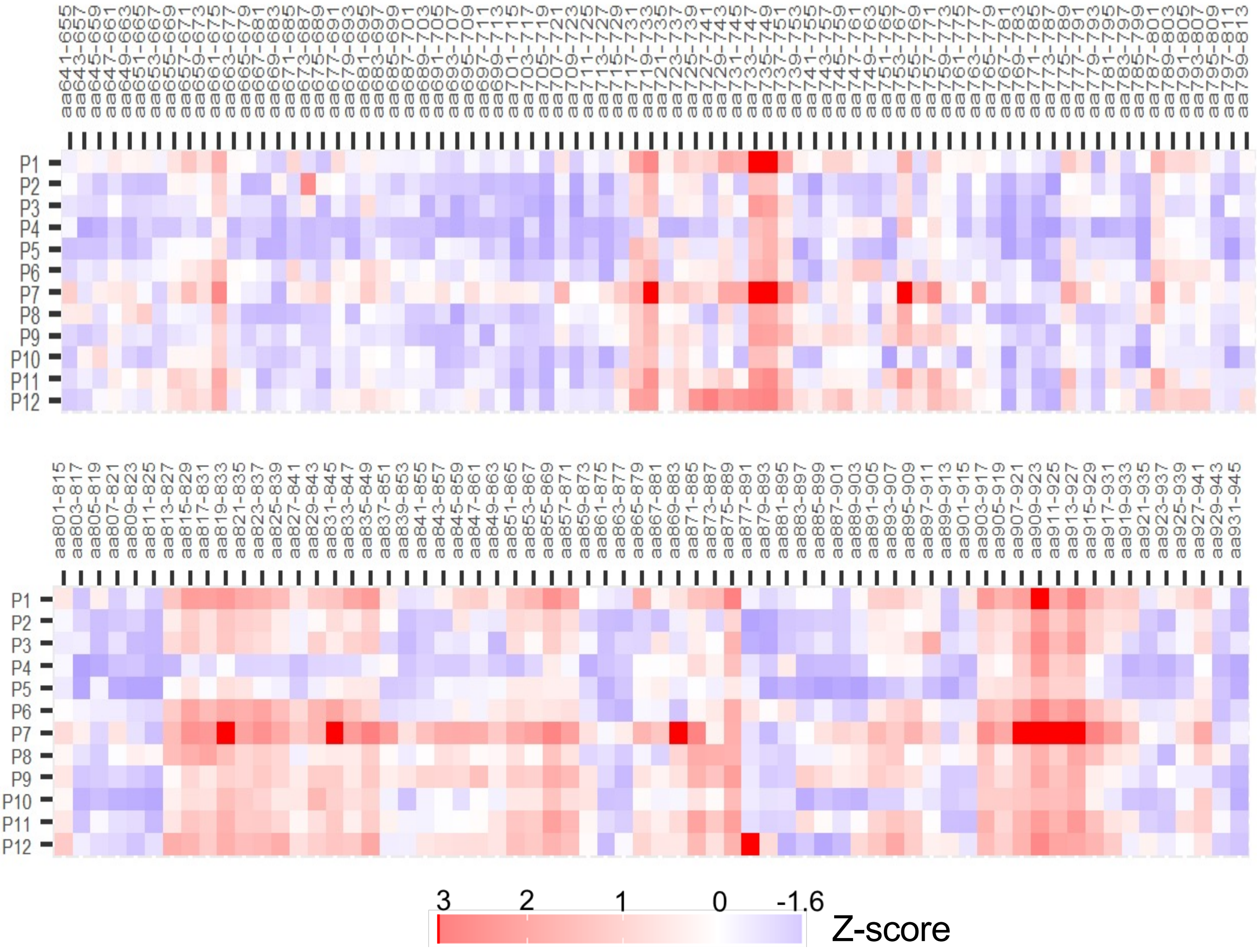

Supplementary Figure S1f: Heatmap showing IgA response for peptide against orf1ab non-structural protein (nsp13). The amino acid sequence of the corresponding protein is denoted as “aa1-15” and the COVID-19 patients included in the study are presented as p1-p12. Immunogenic response against the peptide was visualised using Z-score of fluorescence intensity. Peptides with a Z-score greater than 3 in at least one COVID-19 patients was defined as immunogenic.

nsp13

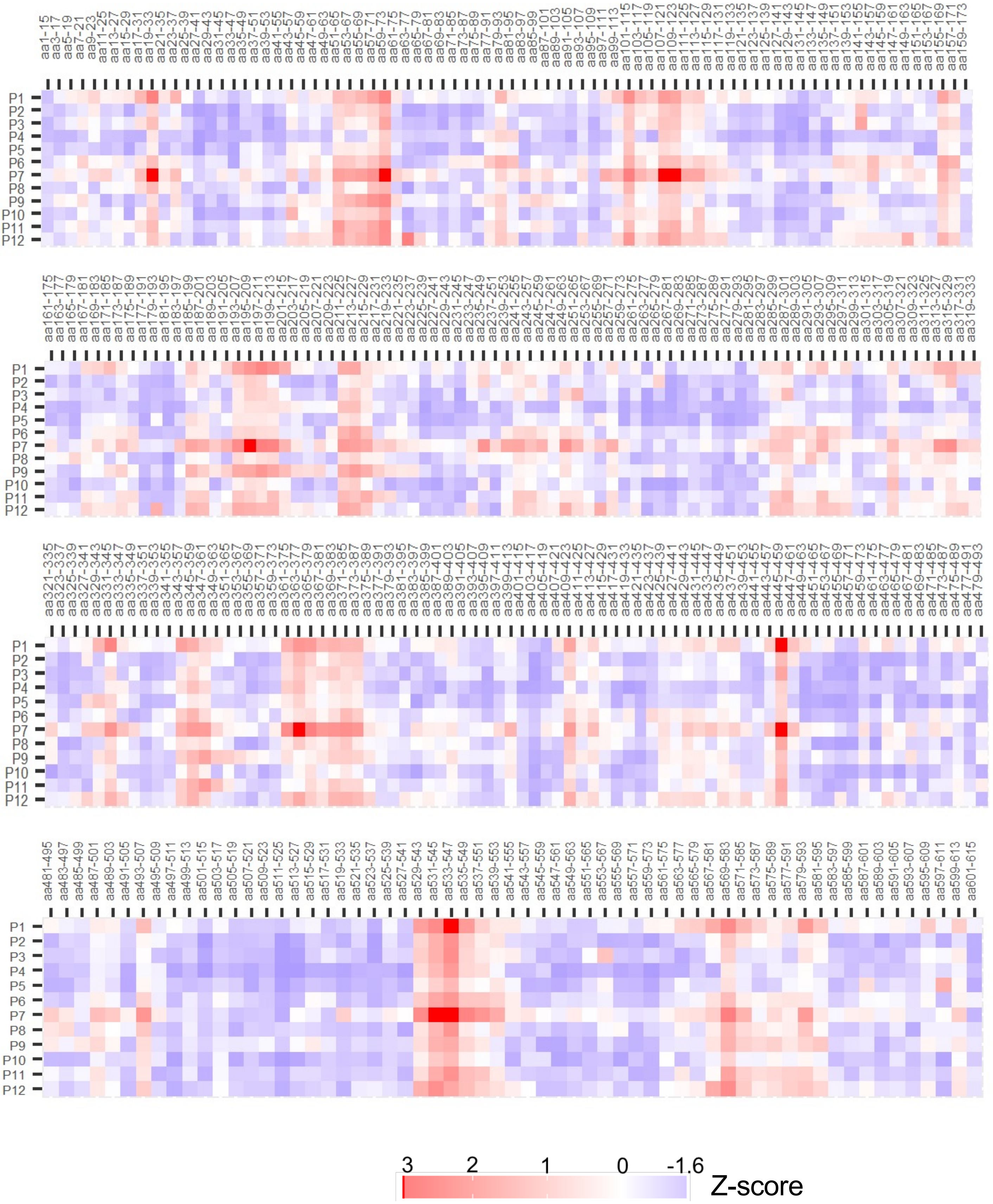

Supplementary Figure S1g: Heatmap showing IgA response for peptide against orf1ab non-structural protein (nsp14, nsp15). The amino acid sequence of the corresponding protein is denoted as "aa1-15" and the COVID-19 patients included in the study are presented as p1-p12. Immunogenic response against the peptide was visualised using Z-score of fluorescence intensity. Peptides with a Z-score greater than 3 in at least one COVID-19 patients was defined as immunogenic.

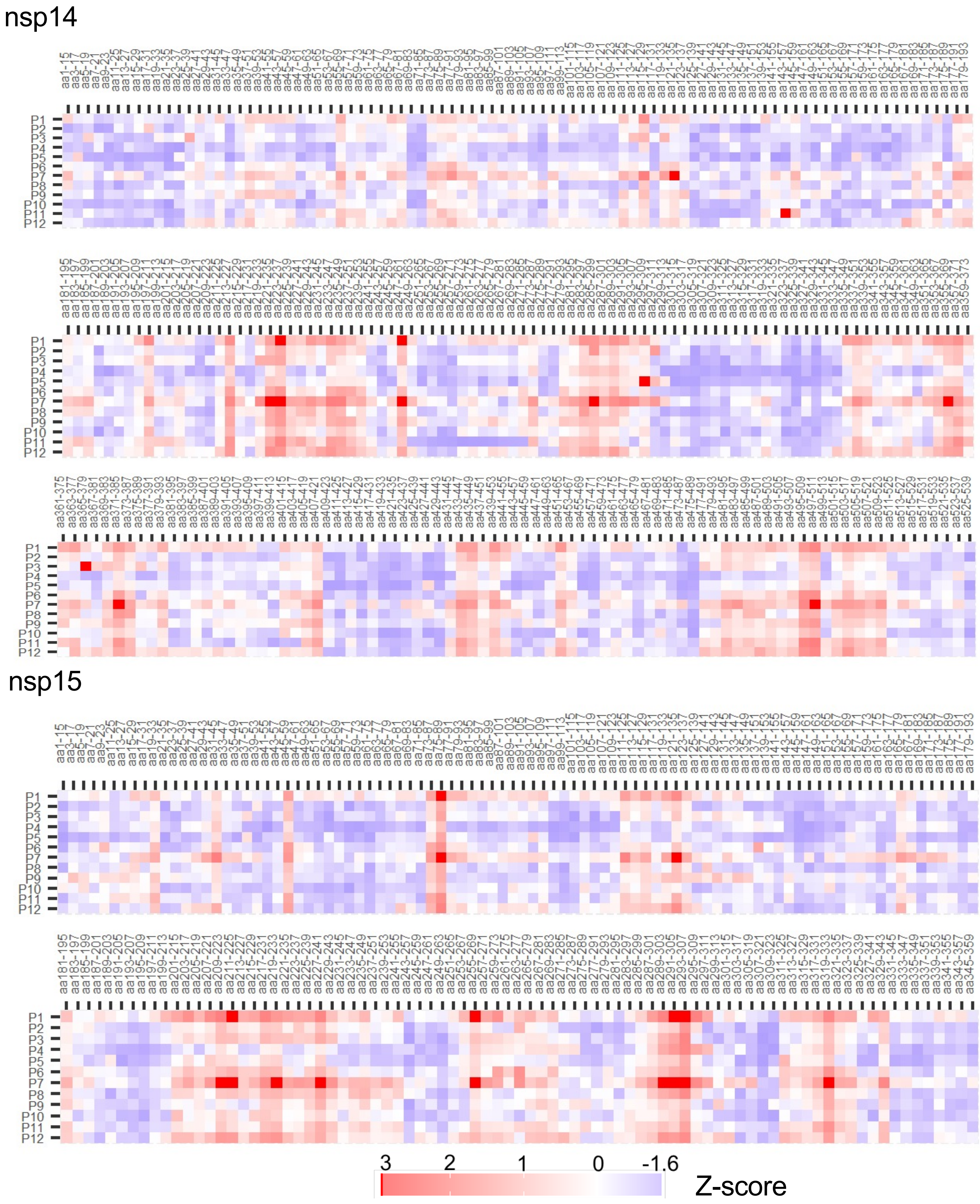

Supplementary Figure S1h: Heatmap showing IgA response for peptide against orf1ab non-structural, structural and accessory proteins (nsp16, membrane, envelop and orf8). The amino acid sequence of the corresponding protein is denoted as "aa1-15" and the COVID-19 patients included in the study are presented as p1-p12. Immunogenic response against the peptide was visualised using Z-score of fluorescence intensity. Peptides with a Z-score greater than 3 in at least one COVID-19 patients was defined as immunogenic.

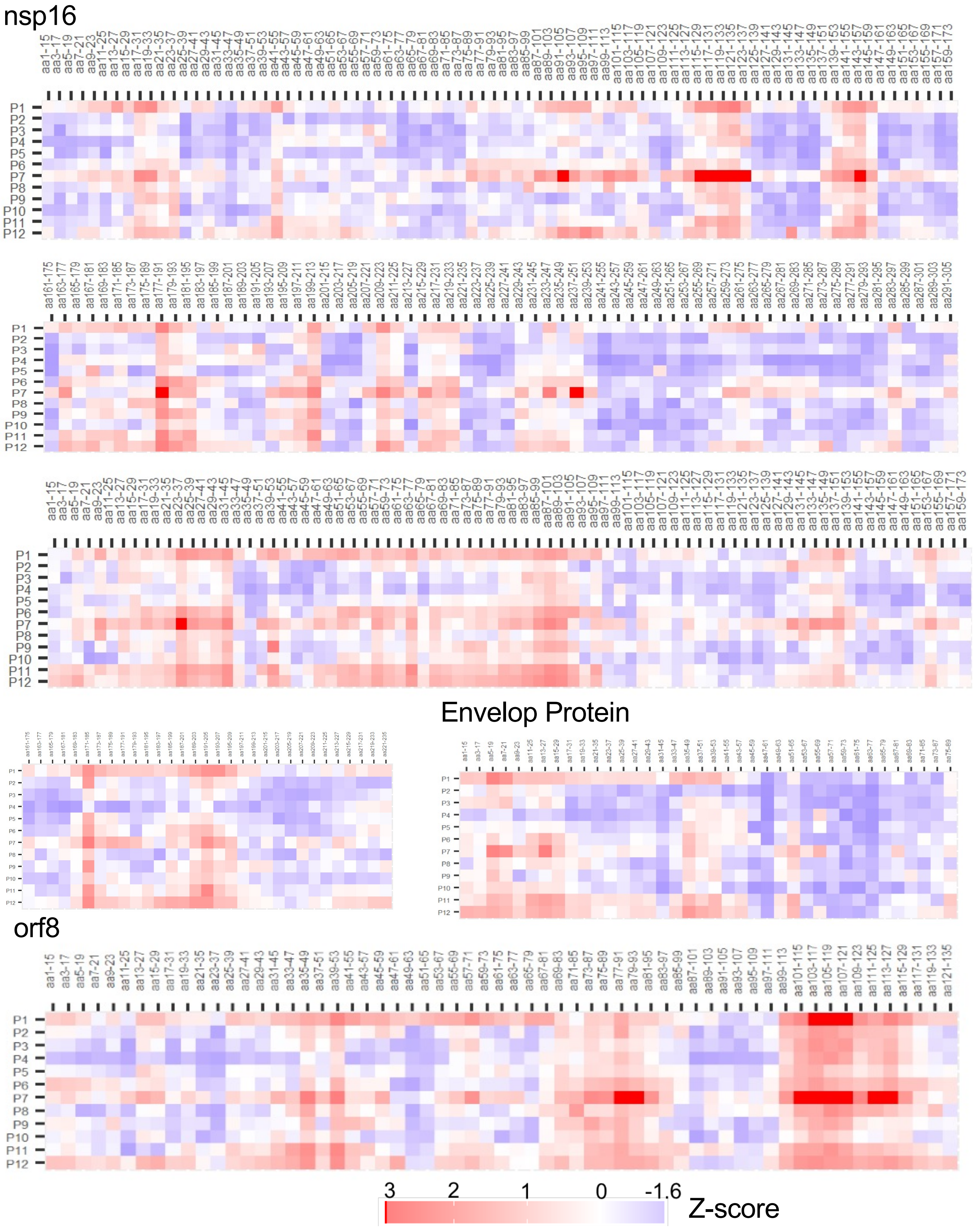

Supplementary Figure S1i: Heatmap showing IgA response for peptide against accessory proteins (orf3a, orf7a, orf6, orf10). The amino acid sequence of the corresponding protein is denoted as “aa1-15” and the COVID-19 patients included in the study are presented as p1-p12. Immunogenic response against the peptide was visualised using Z-score of fluorescence intensity. Peptides with a Z-score greater than 3 in at least one COVID-19 patients was defined as immunogenic.

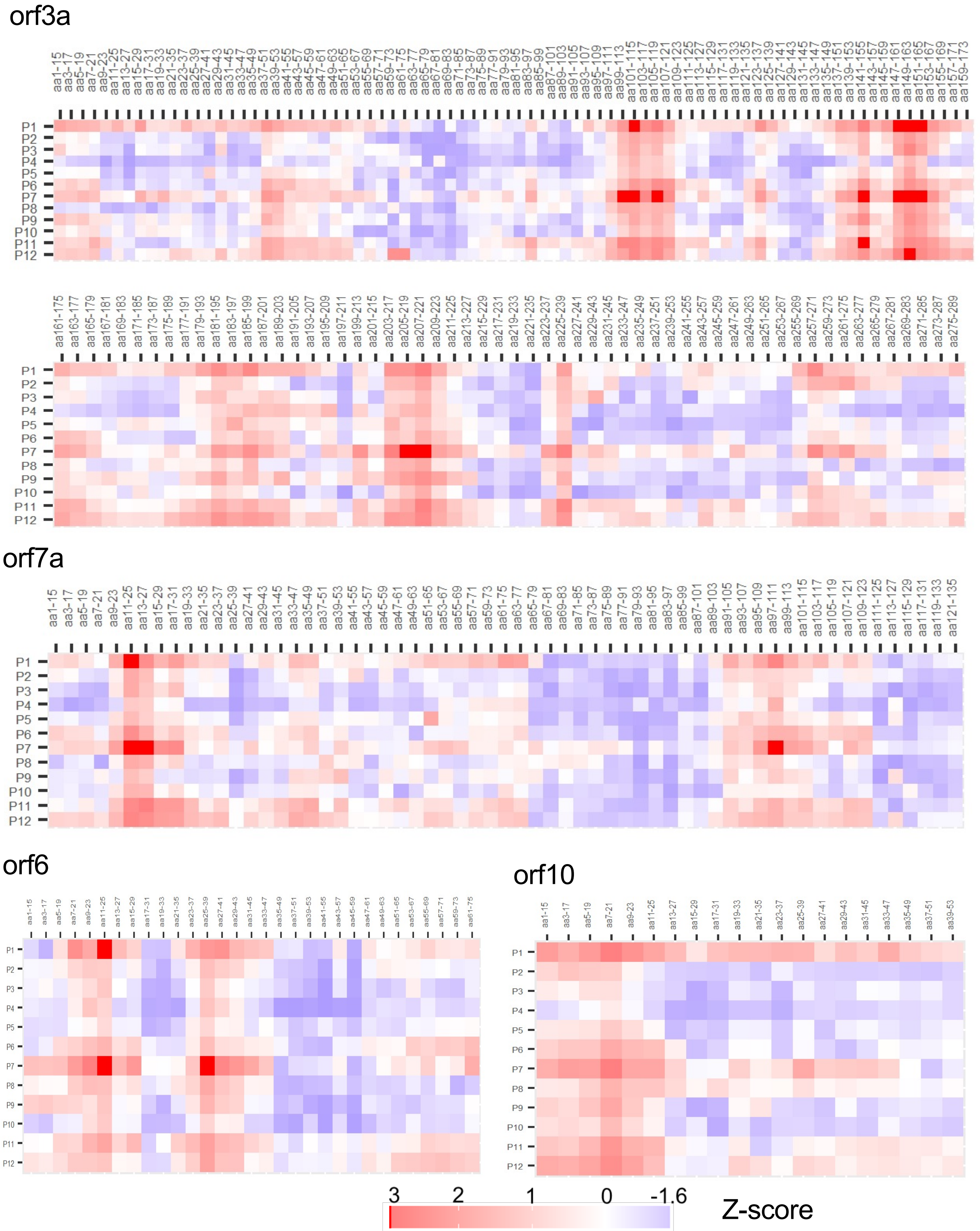

Supplementary Figure S1j: Heatmap showing IgA response for peptide against structural proteins (Spike). The amino acid sequence of the corresponding protein is denoted as “aa1-15” and the COVID-19 patients included in the study are presented as p1-p12. Immunogenic response against the peptide was visualised using Z-score of fluorescence intensity. Peptides with a Z-score greater than 3 in at least one COVID-19 patients was defined as immunogenic.

Spike Protein

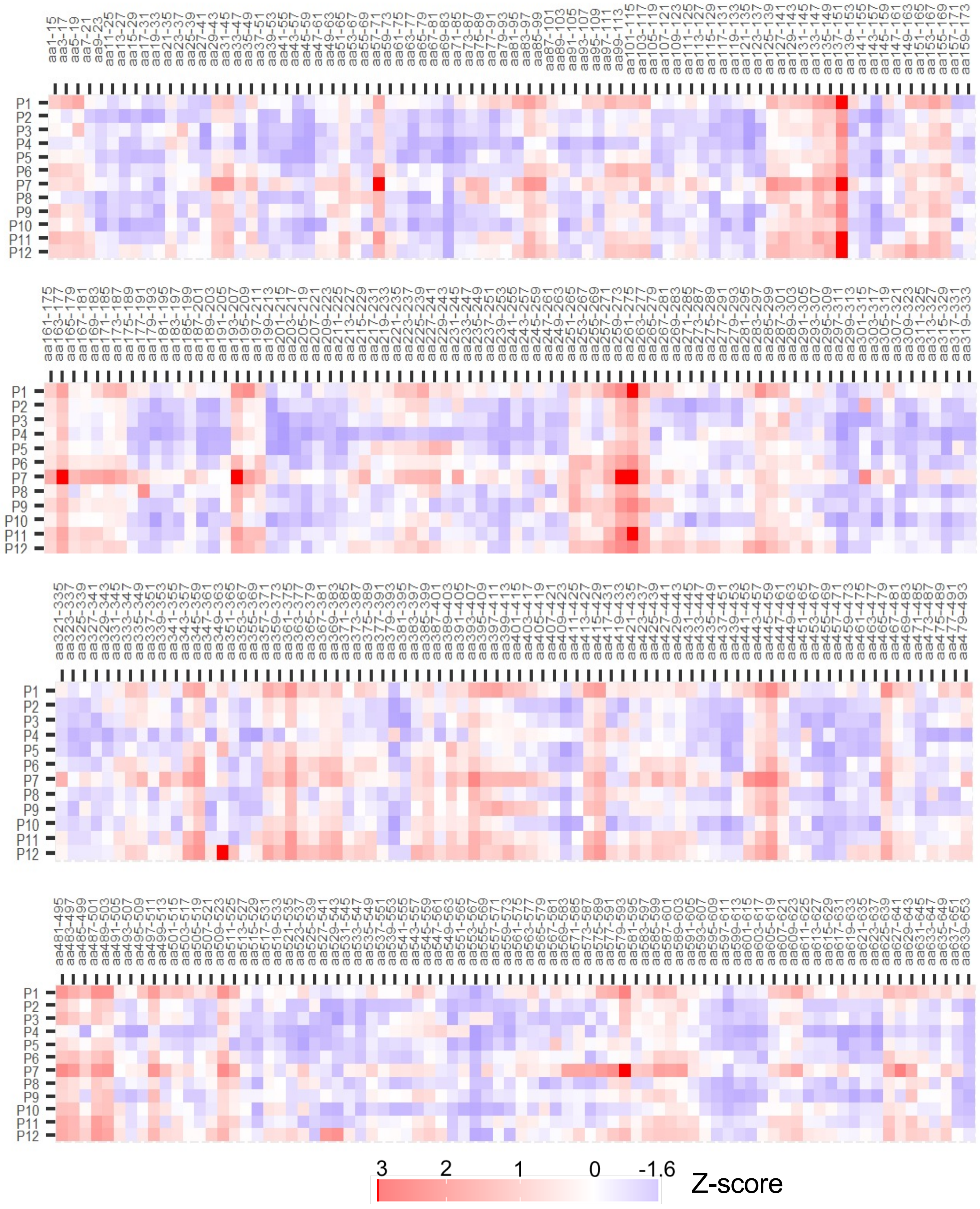

Supplementary Figure S1j contd…

Spike Protein

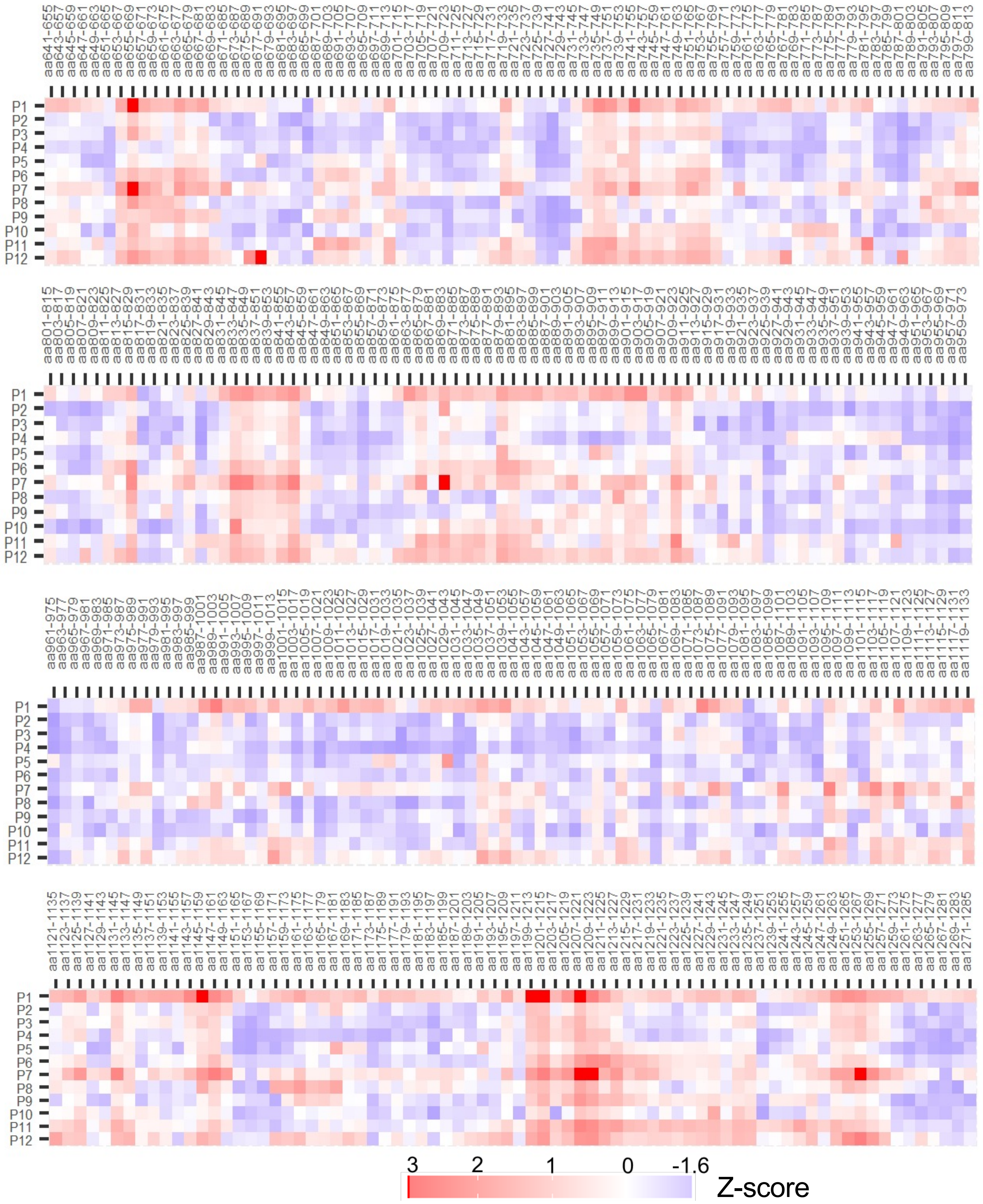

Supplementary Figure S1k: Heatmap showing IgA response for peptide against structural proteins (Nucleocapsid Phosphoprotein). The amino acid sequence of the corresponding protein is denoted as “aa1-15” and the COVID-19 patients included in the study are presented as p1-p12. Immunogenic response against the peptide was visualised using Z-score of fluorescence intensity. Peptides with a Z-score greater than 3 in at least one COVID-19 patients was defined as immunogenic.

Nucleocapsid Phosphoprotein

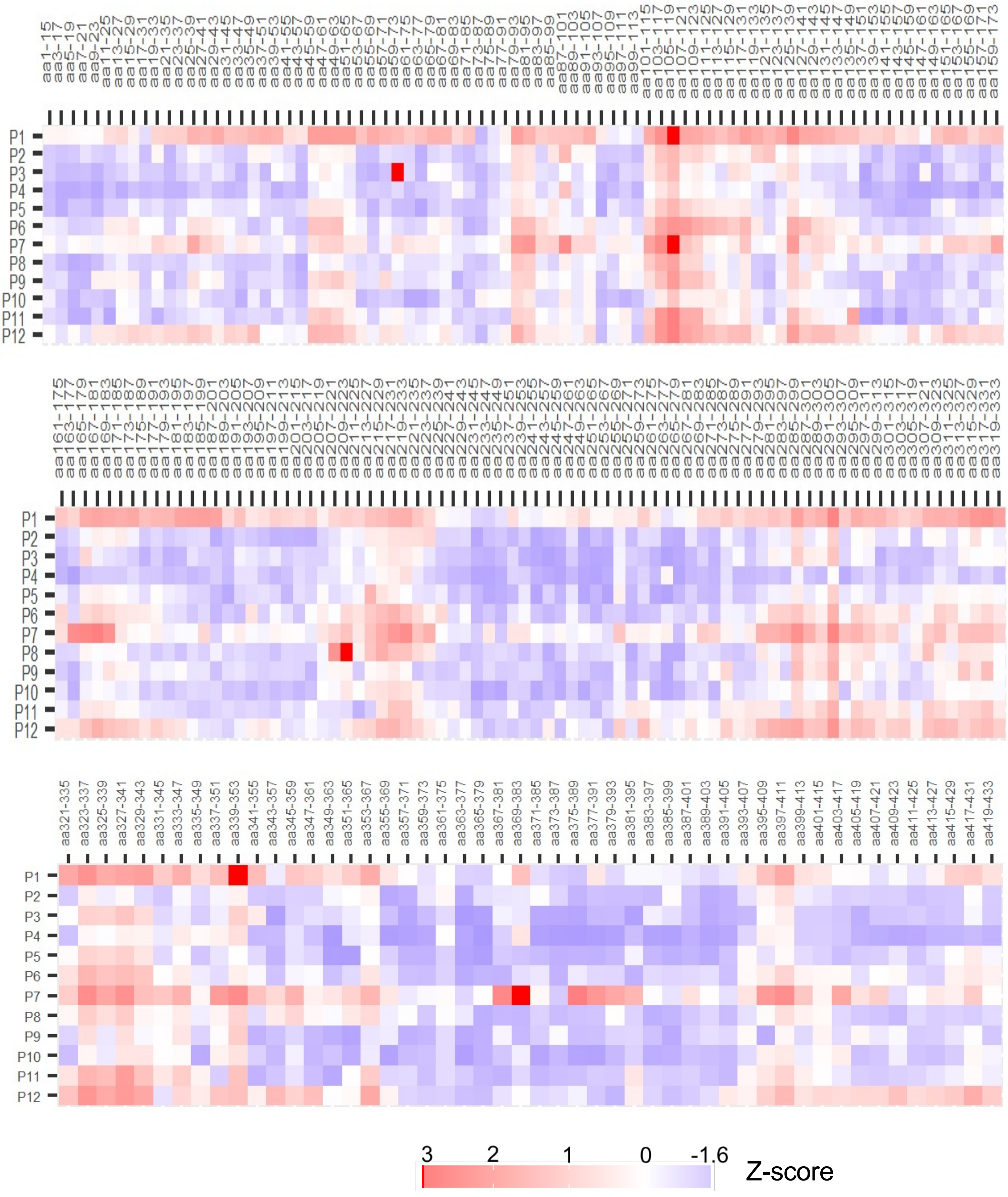

Supplementary Figure S2a: Heatmap showing IgG response for peptide against orf1ab non-structural protein (nsp1, nsp2). The amino acid sequence of the corresponding protein is denoted as “aa1-15” and the COVID-19 patients included in the study are presented as p1-p12. Immunogenic response against the peptide was visualised using Z-score of fluorescence intensity. Peptides with a Z-score greater than 3 in at least one COVID-19 patients was defined as immunogenic.

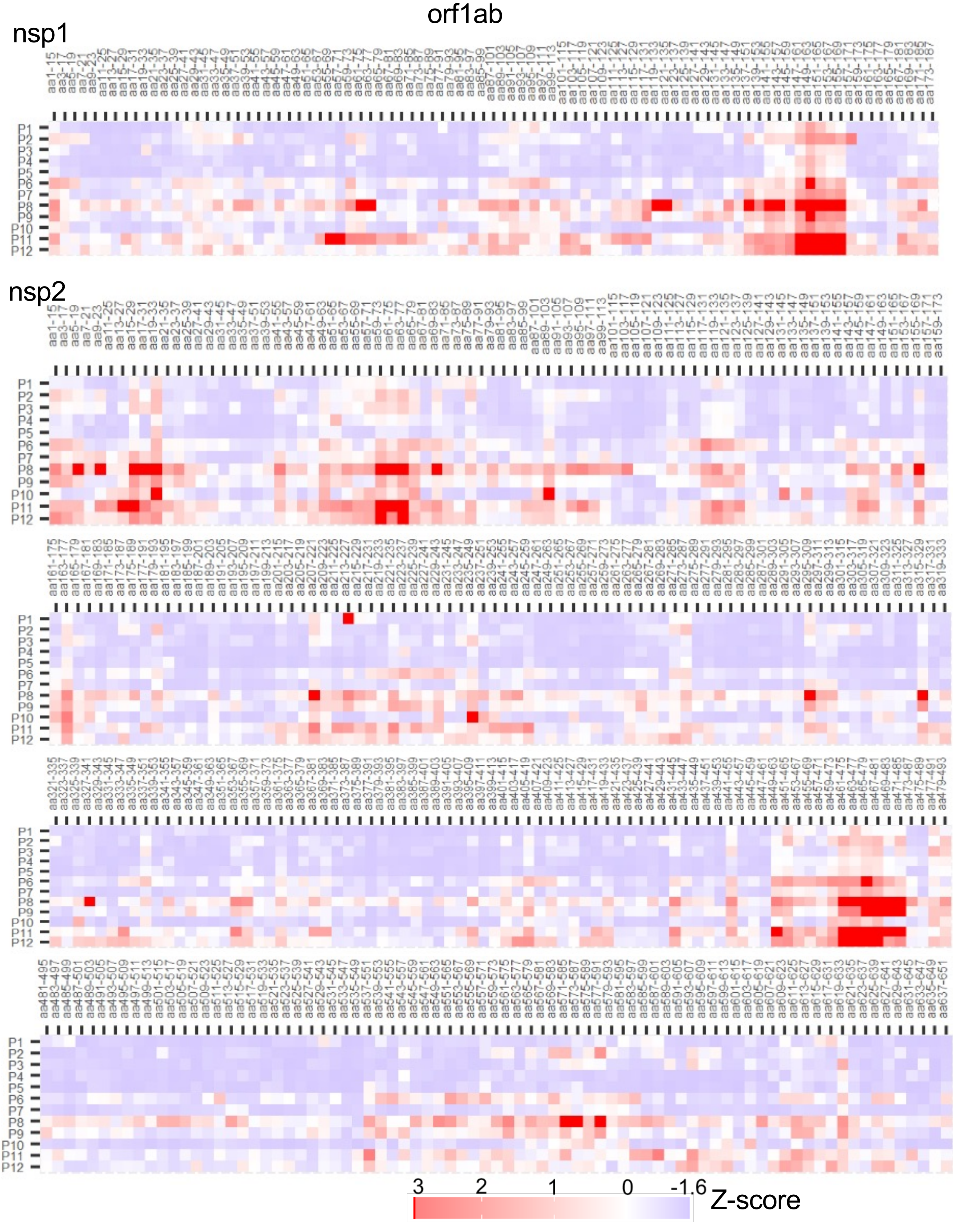

Supplementary Figure S2b: Heatmap showing IgG response for peptide against orf1ab non-structural protein (nsp3). The amino acid sequence of the corresponding protein is denoted as “aa1-15” and the COVID-19 patients included in the study are presented as p1-p12. Immunogenic response against the peptide was visualised using Z-score of fluorescence intensity. Peptides with a Z-score greater than 3 in at least one COVID-19 patients was defined as immunogenic.

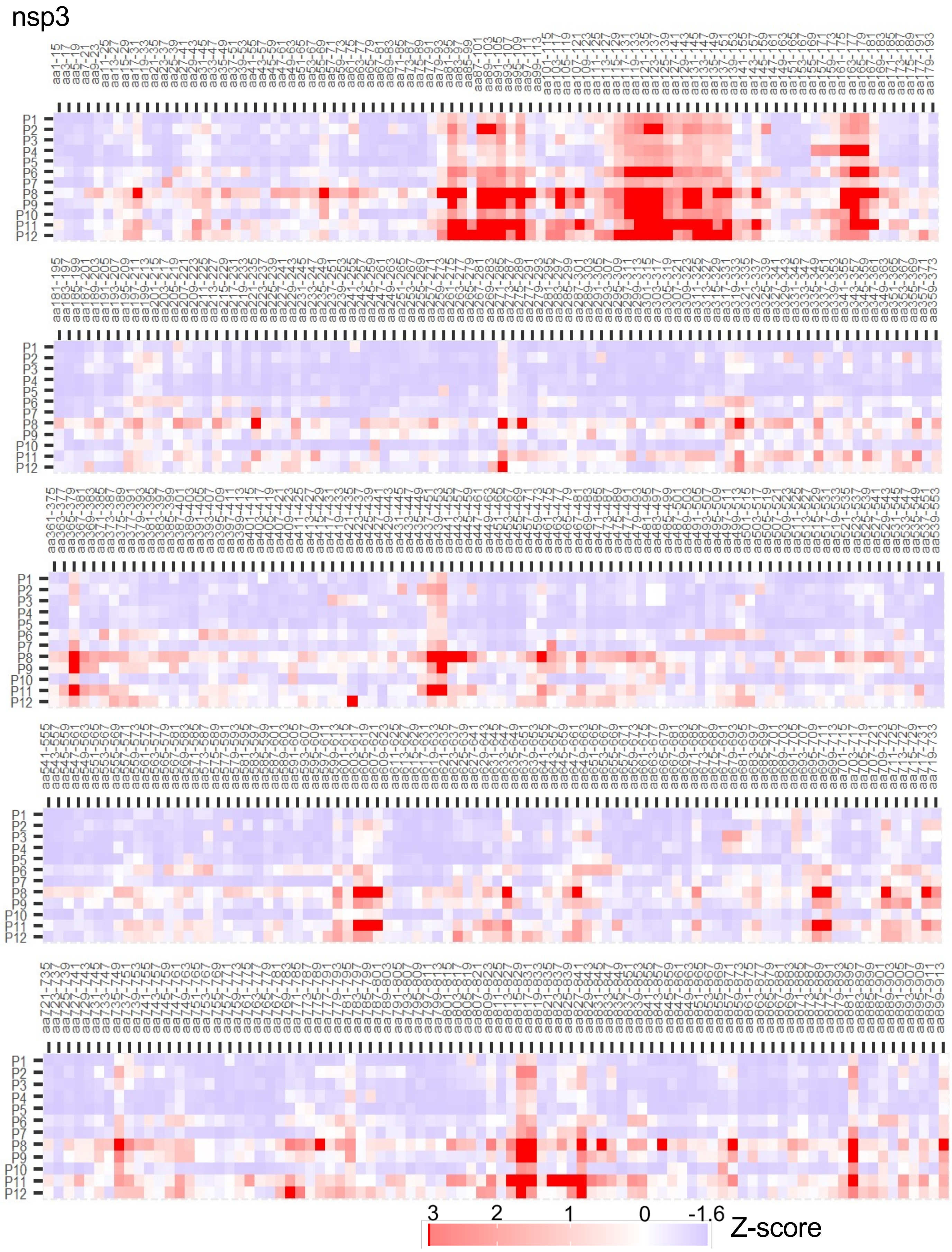

Supplementary Figure S2b contd…

nsp3

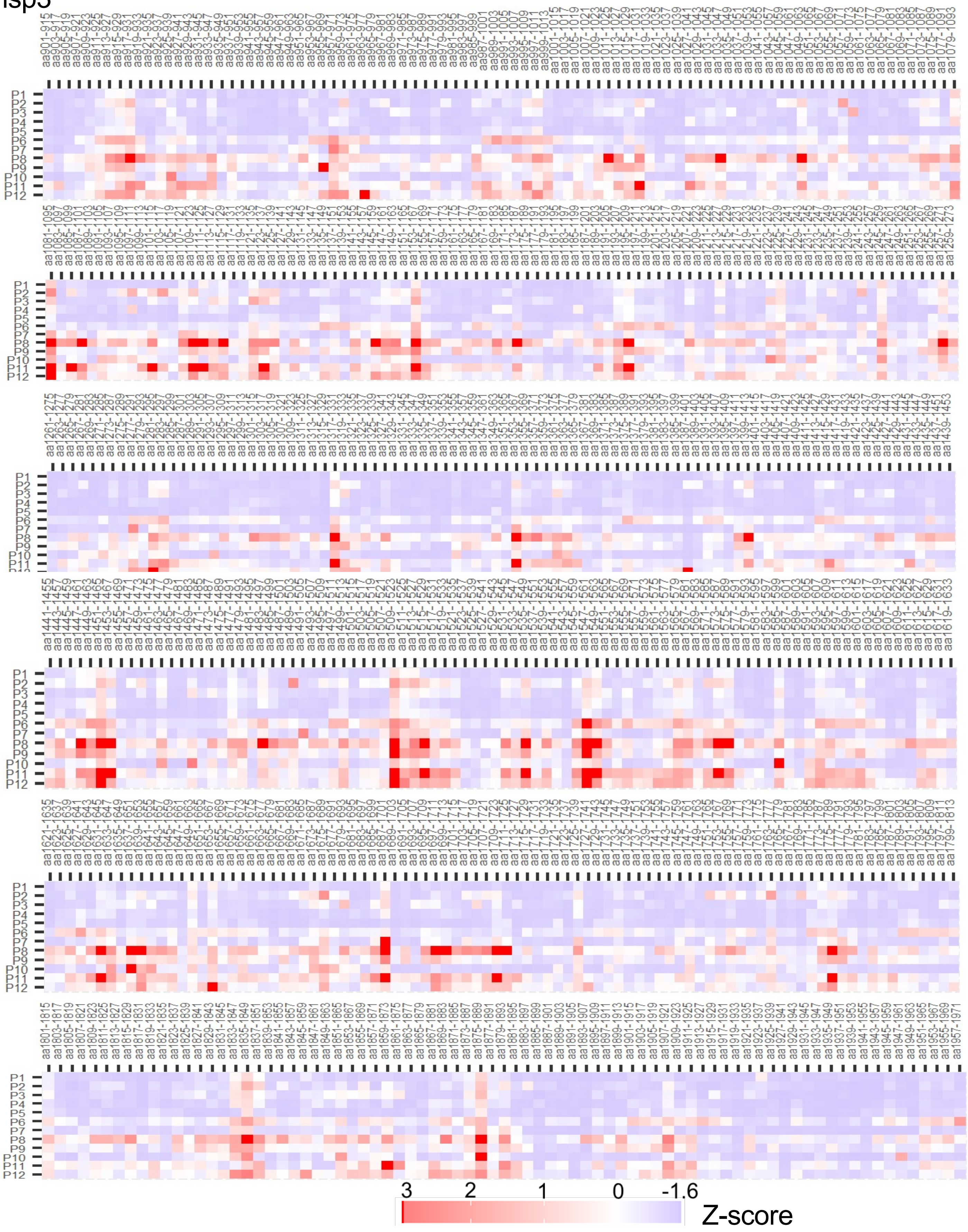

Supplementary Figure S2c: Heatmap showing IgG response for peptide against orf1ab non-structural protein (nsp4, nsp5). The amino acid sequence of the corresponding protein is denoted as “aa1-15” and the COVID-19 patients included in the study are presented as p1-p12. Immunogenic response against the peptide was visualised using Z-score of fluorescence intensity. Peptides with a Z-score greater than 3 in at least one COVID-19 patients was defined as immunogenic.

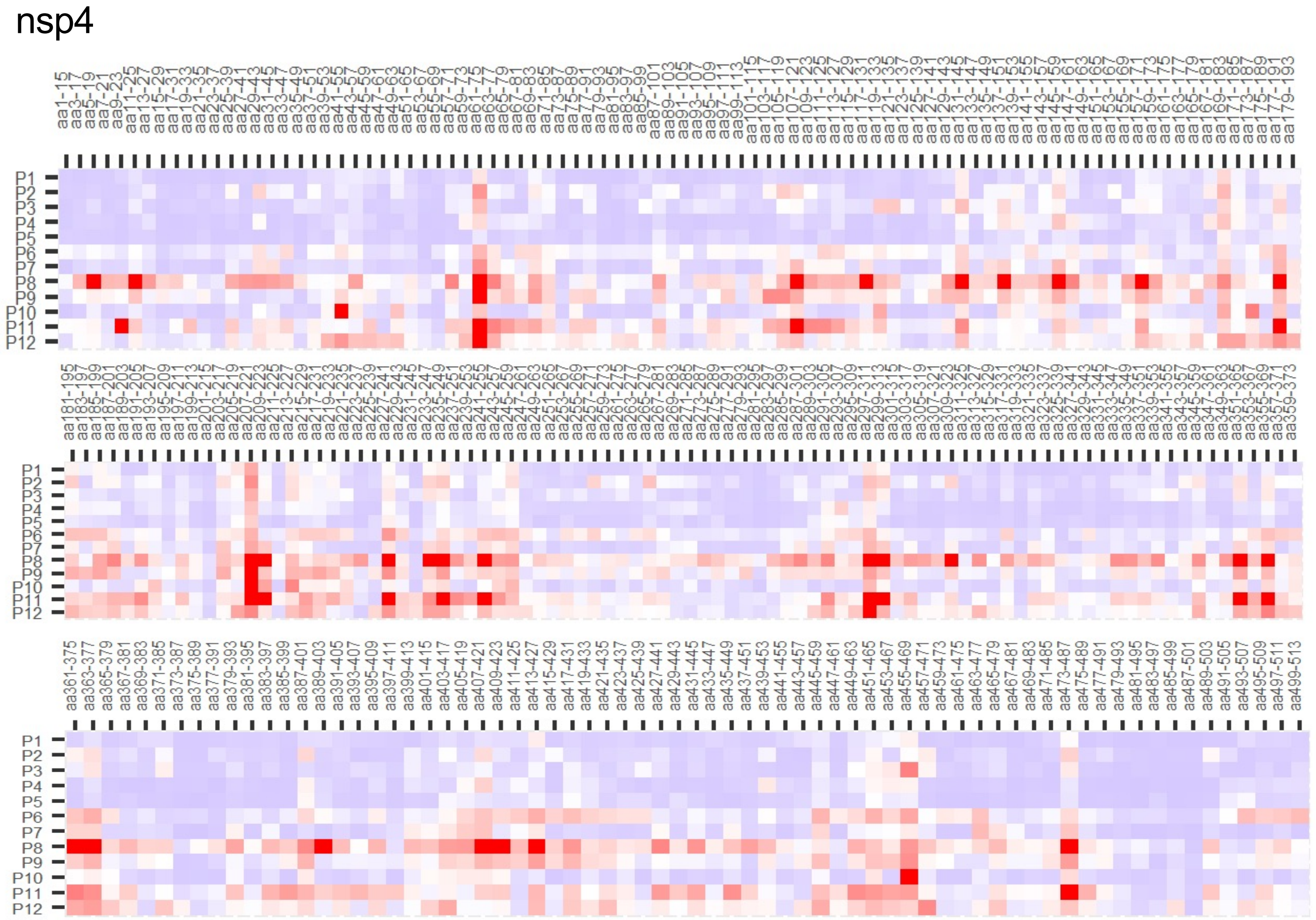

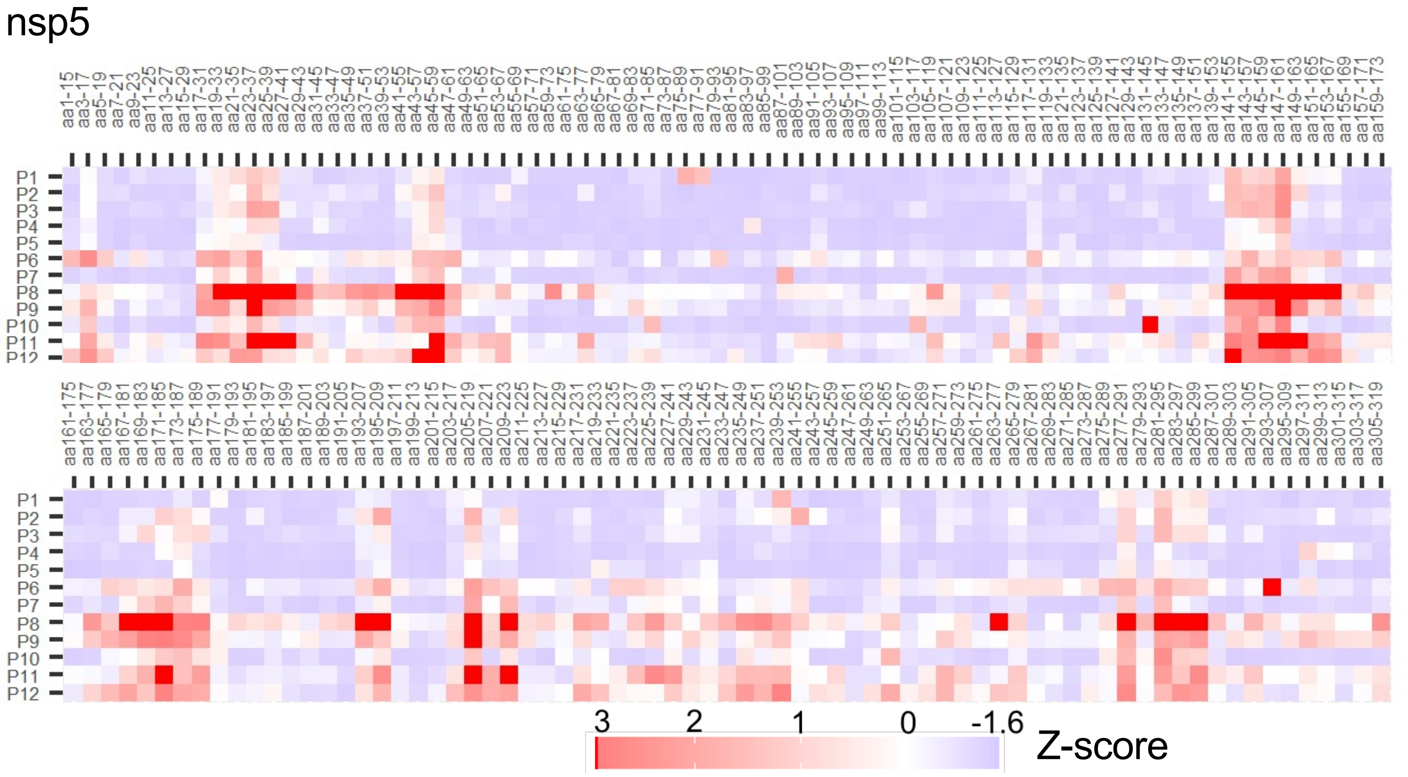

Supplementary Figure S2d: Heatmap showing IgG response for peptide against orf1ab non-structural protein (nsp6, nsp7, nsp8, nsp9). The amino acid sequence of the corresponding protein is denoted as "aa1-15" and the COVID-19 patients included in the study are presented as p1-p12. Immunogenic response against the peptide was visualised using Z-score of fluorescence intensity. Peptides with a Z-score greater than 3 in at least one COVID-19 patients was defined as immunogenic.

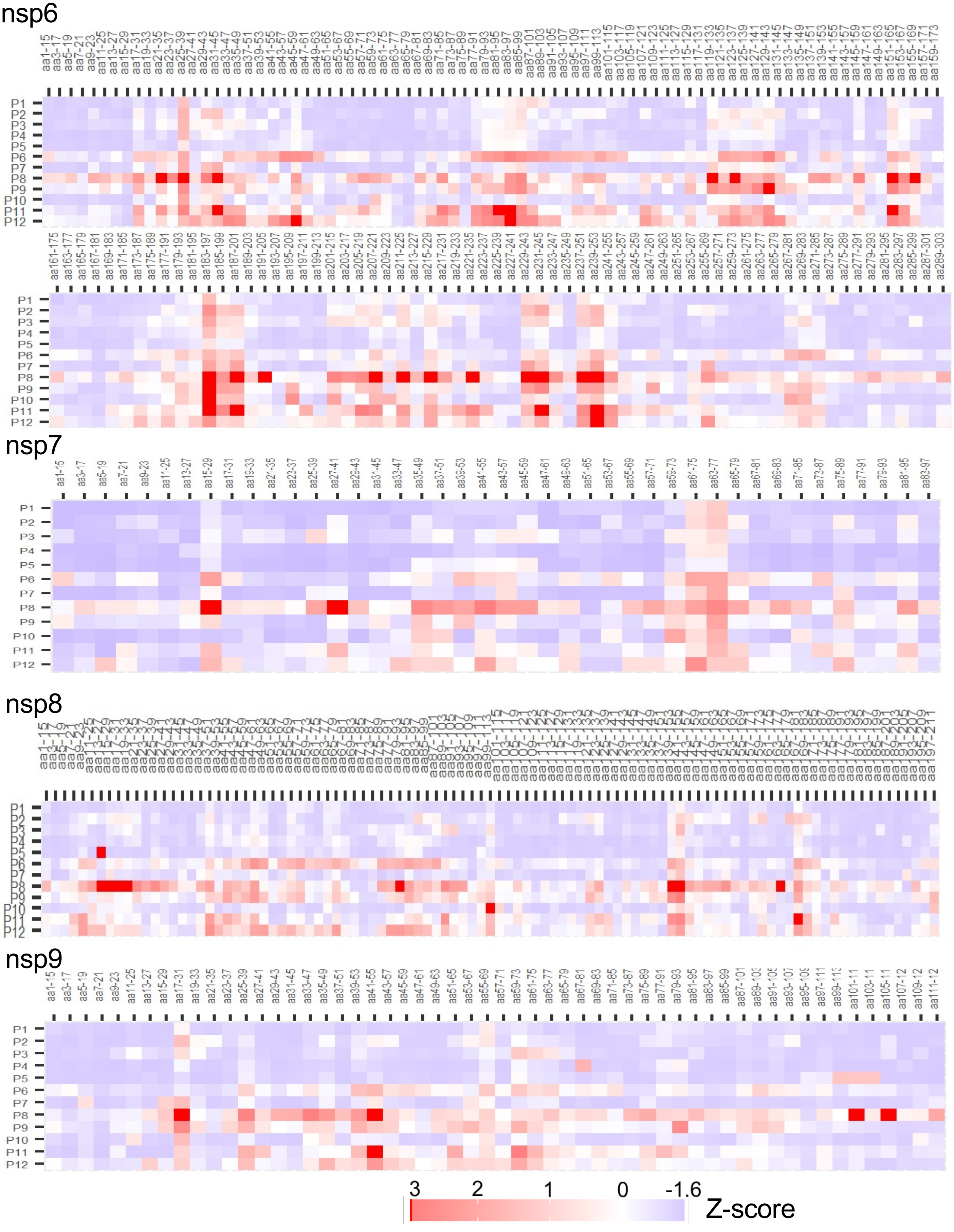

Supplementary Figure S2e: Heatmap showing IgG response for peptide against orf1ab non-structural protein (nsp10, nsp12). The amino acid sequence of the corresponding protein is denoted as "aa1-15" and the COVID-19 patients included in the study are presented as p1-p12. Immunogenic response against the peptide was visualised using Z-score of fluorescence intensity. Peptides with a Z-score greater than 3 in at least one COVID-19 patients was defined as immunogenic.

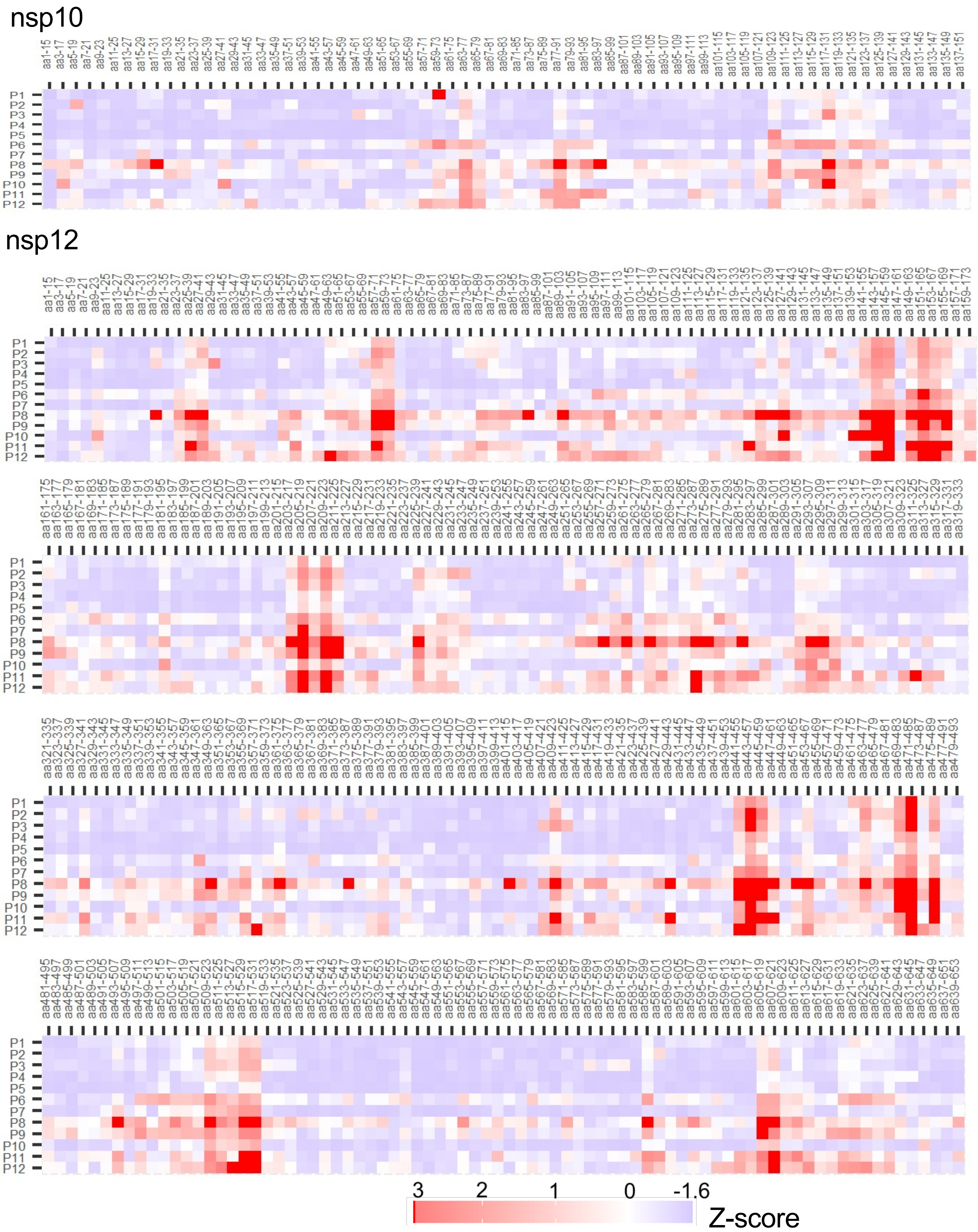

Supplementary Figure S2e contd…

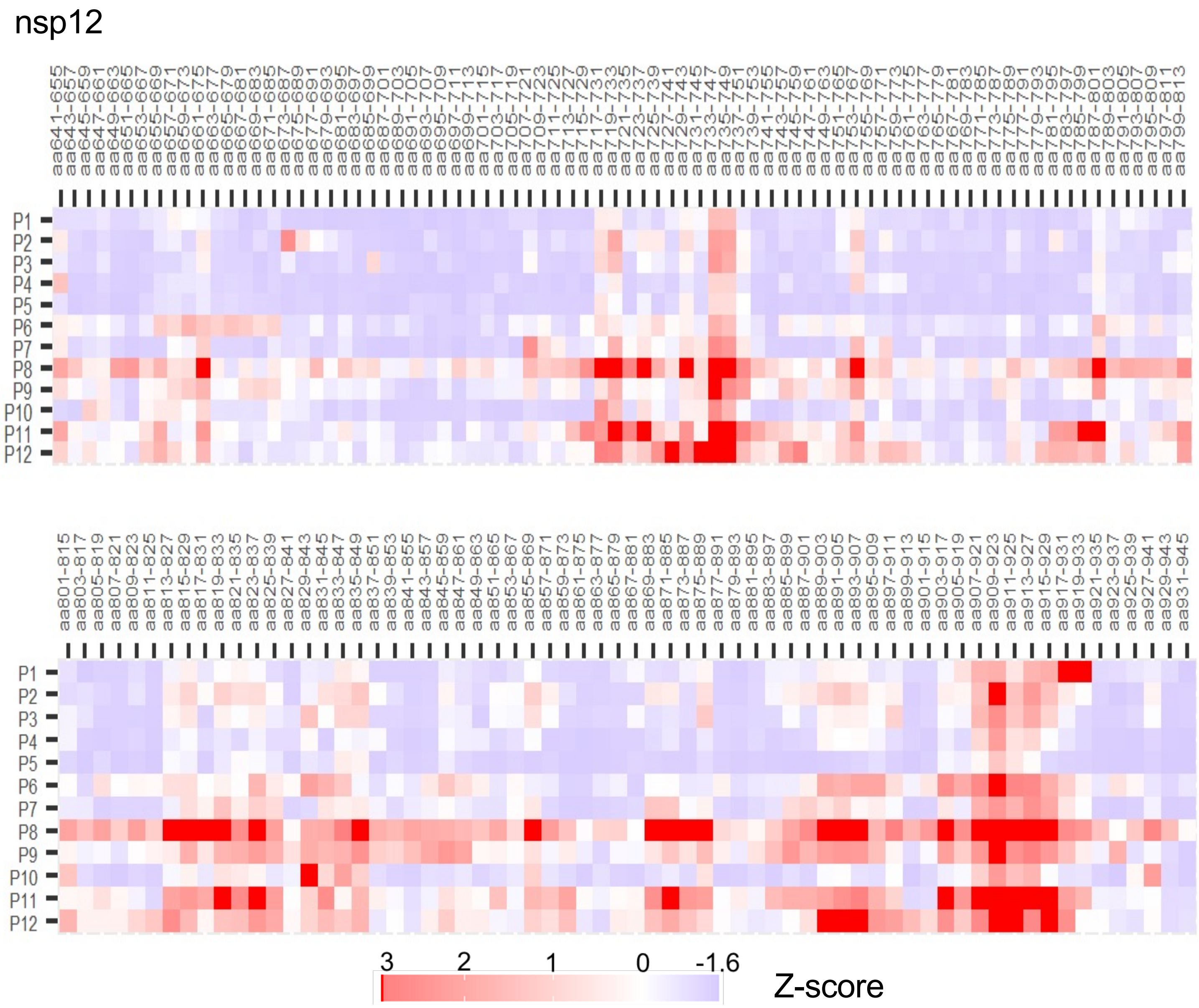

Supplementary Figure S2f: Heatmap showing IgG response for peptide against orf1ab non-structural protein (nsp13). The amino acid sequence of the corresponding protein is denoted as “aa1-15” and the COVID-19 patients included in the study are presented as p1-p12. Immunogenic response against the peptide was visualised using Z-score of fluorescence intensity. Peptides with a Z-score greater than 3 in at least one COVID-19 patients was defined as immunogenic.

nsp13

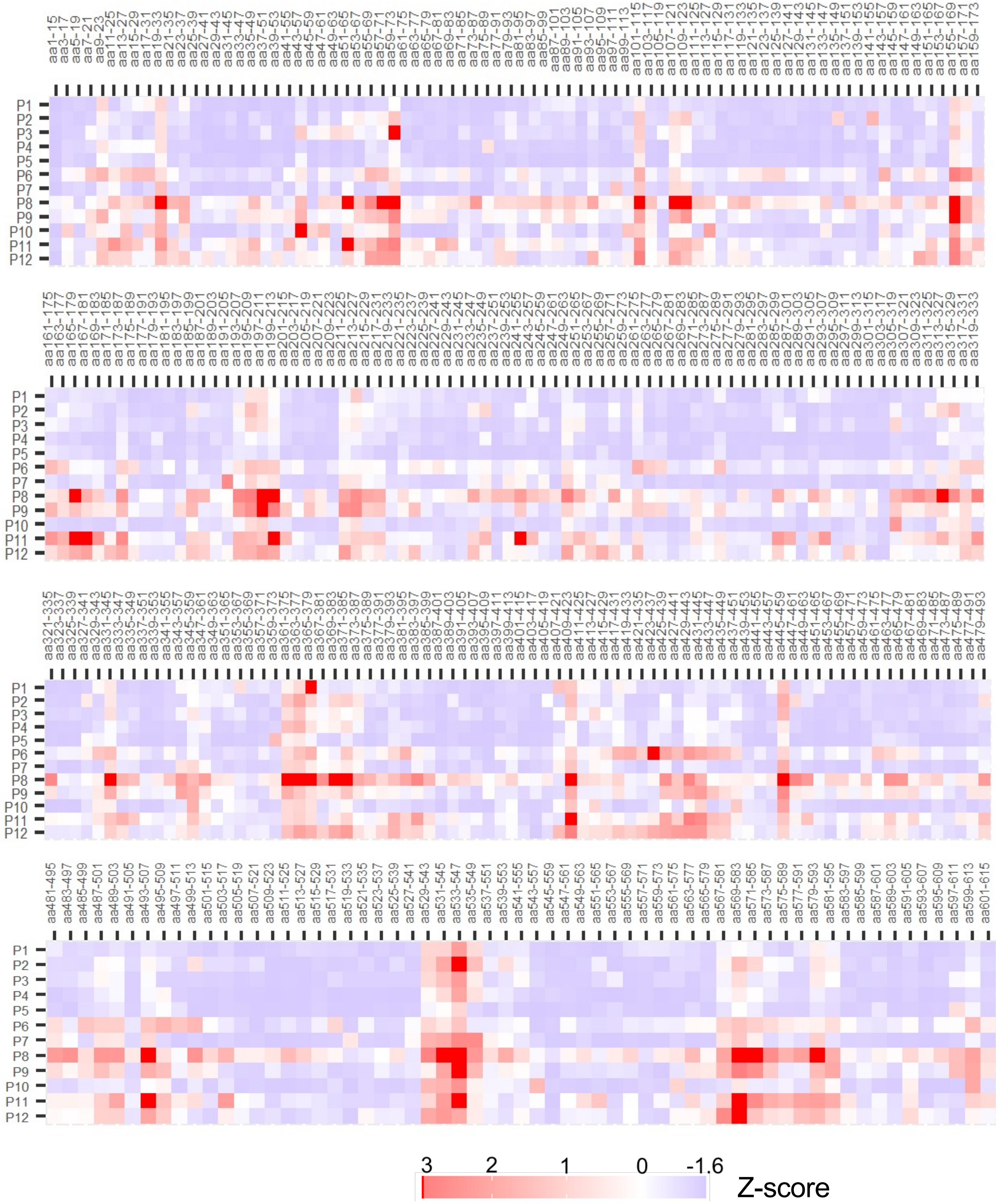

Supplementary Figure S2g: Heatmap showing IgG response for peptide against orf1ab non-structural protein (nsp14, nsp15). The amino acid sequence of the corresponding protein is denoted as “aa1-15” and the COVID-19 patients included in the study are presented as p1-p12. Immunogenic response against the peptide was visualised using Z-score of fluorescence intensity. Peptides with a Z-score greater than 3 in at least one COVID-19 patients was defined as immunogenic.

nsp14

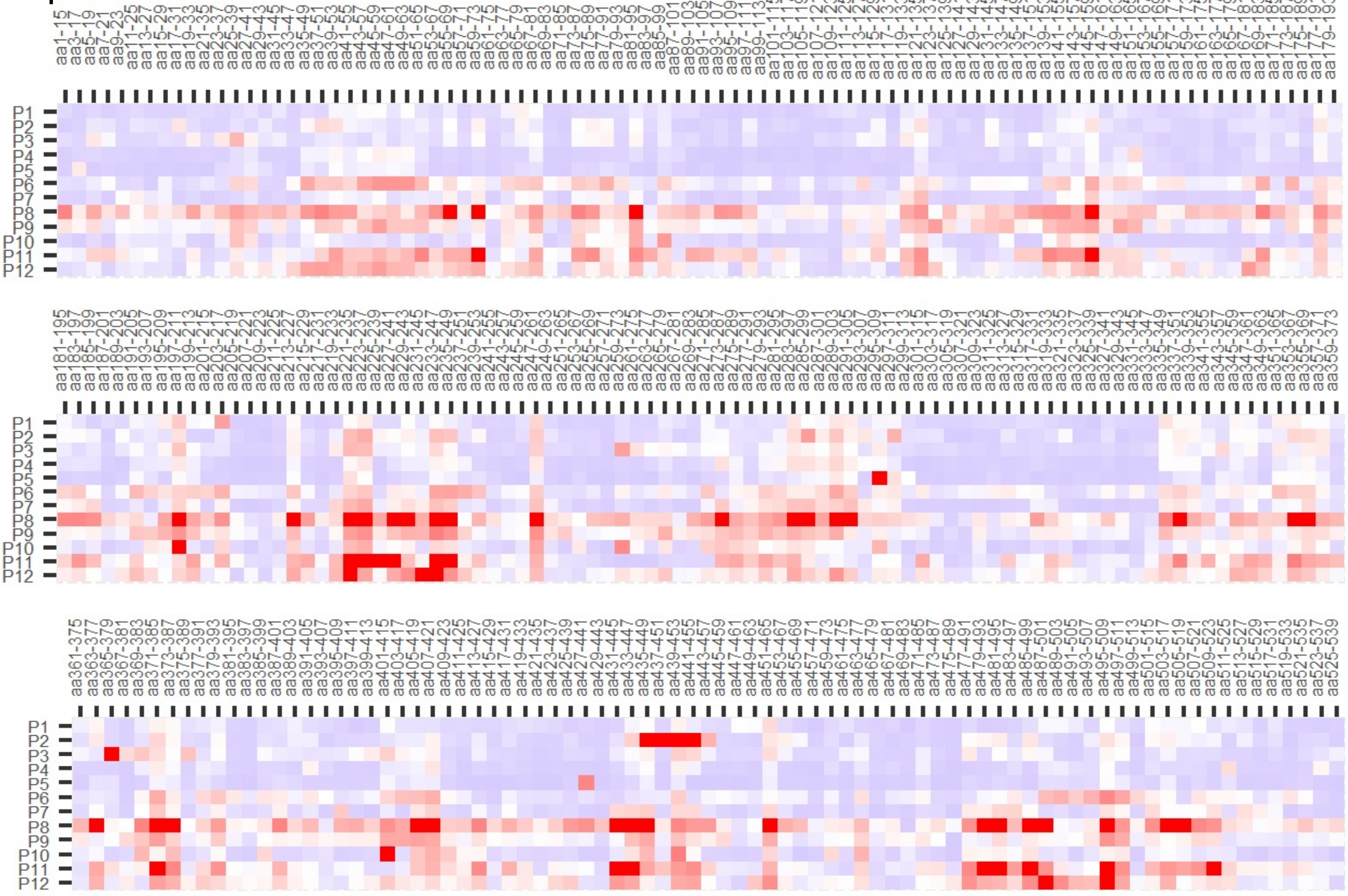

nsp15

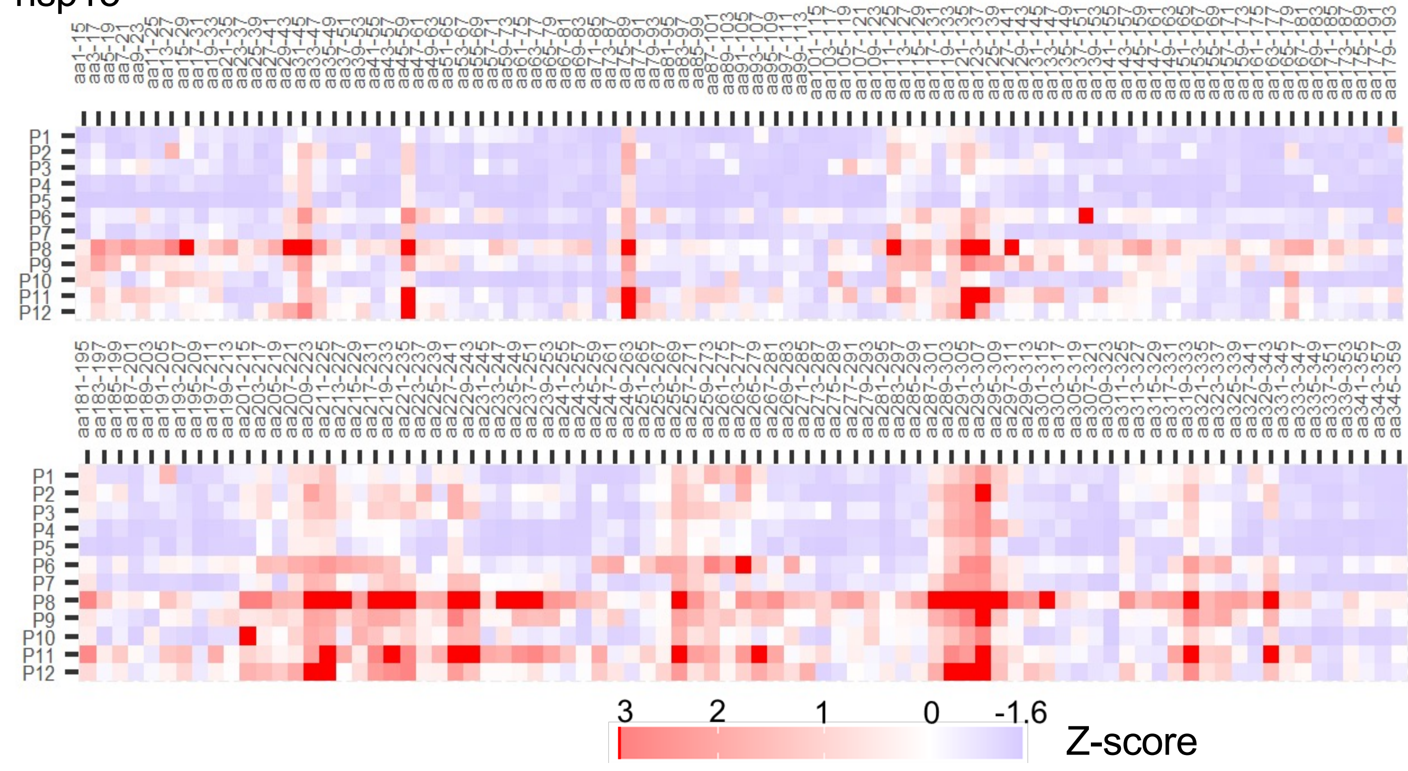

Supplementary Figure S2h: Heatmap showing IgG response for peptide against orf1ab non-structural, structural and accessory proteins (nsp16, membrane, envelop and orf8). The amino acid sequence of the corresponding protein is denoted as “aa1-15” and the COVID-19 patients included in the study are presented as p1-p12. Immunogenic response against the peptide was visualised using Z-score of fluorescence intensity. Peptides with a Z-score greater than 3 in at least one COVID-19 patients was defined as immunogenic.

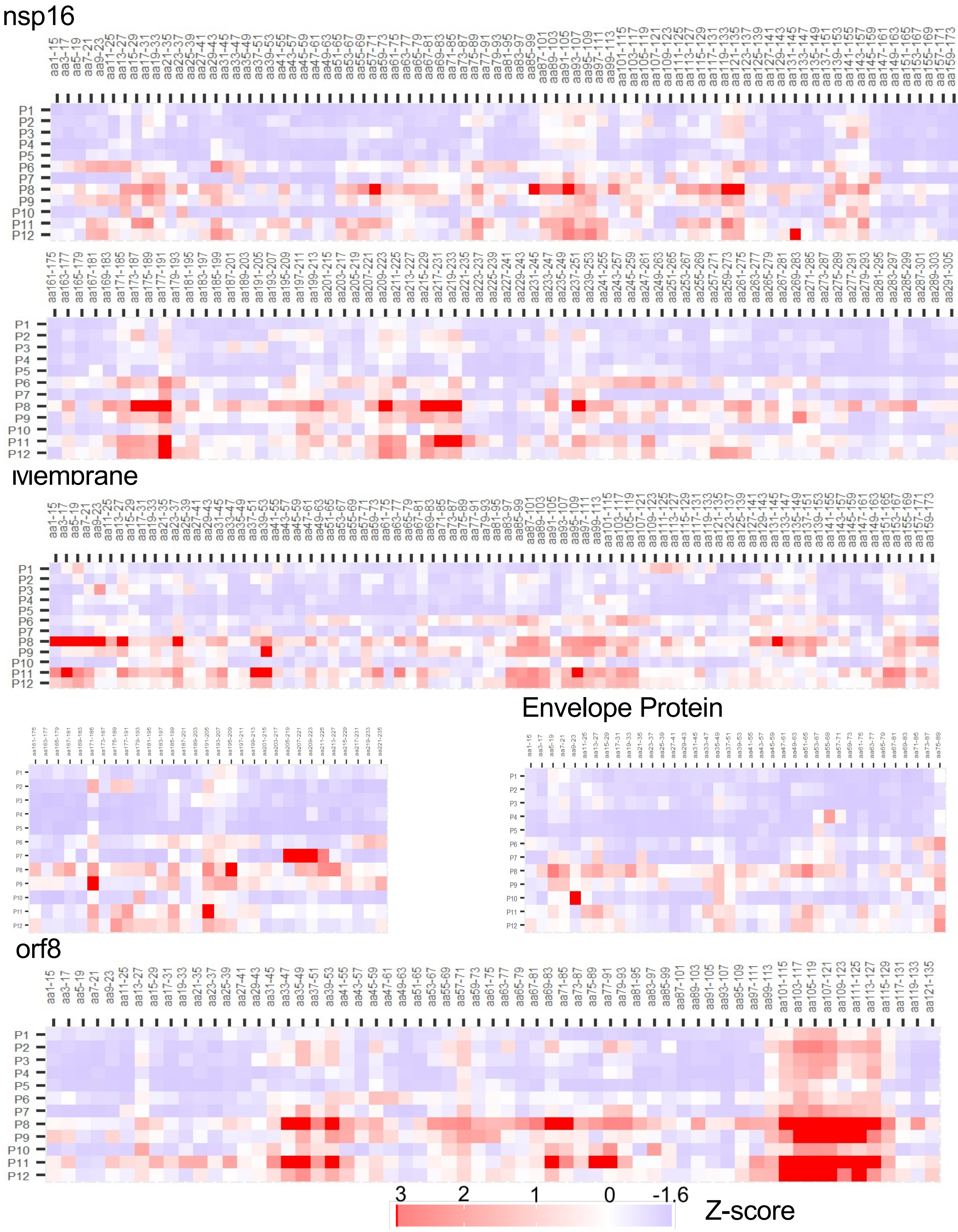

Supplementary Figure S2i: Heatmap showing IgG response for peptide against accessory proteins (orf3a, orf7a, orf6, orf10). The amino acid sequence of the corresponding protein is denoted as “aa1-15” and the COVID-19 patients included in the study are presented as p1-p12. Immunogenic response against the peptide was visualised using Z-score of fluorescence intensity. Peptides with a Z-score greater than 3 in at least one COVID-19 patients was defined as immunogenic.

orf3a

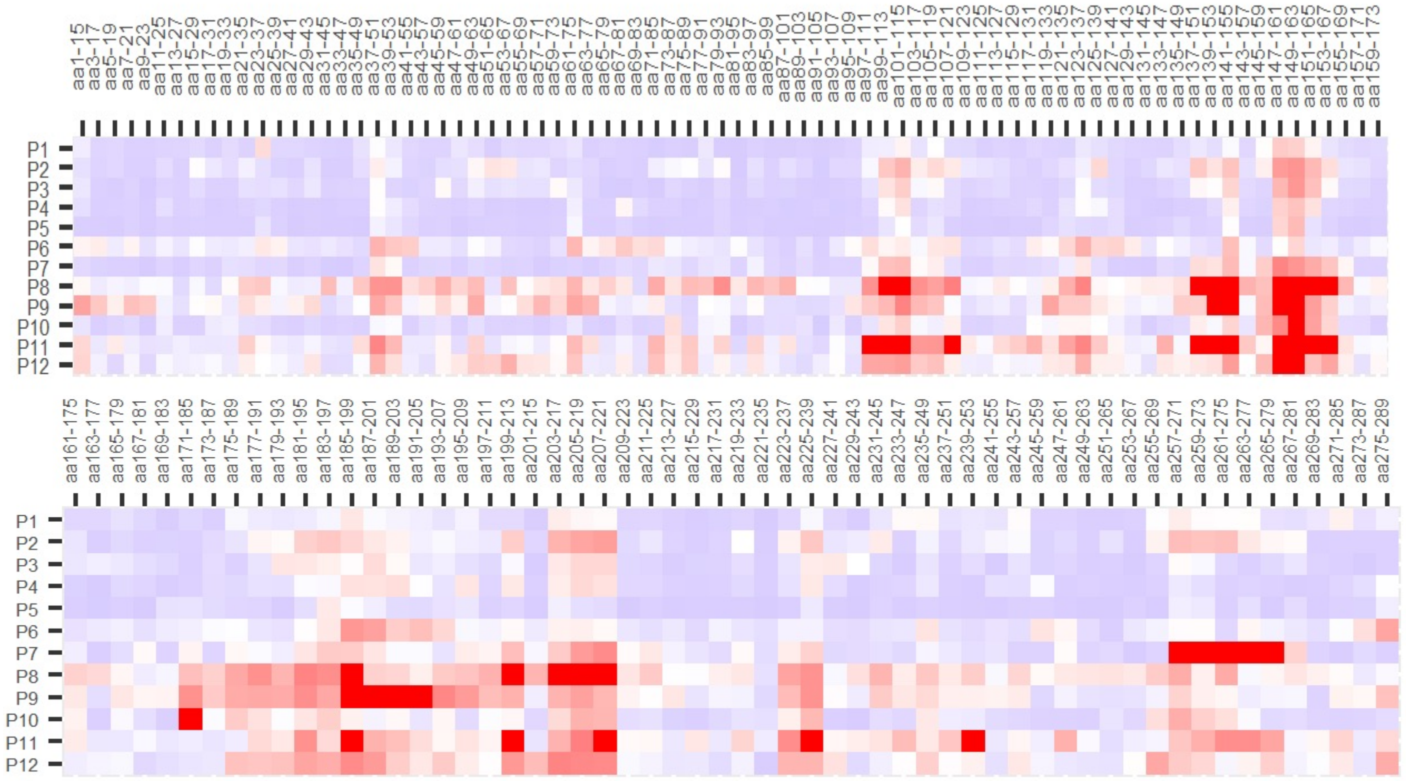

orf7a

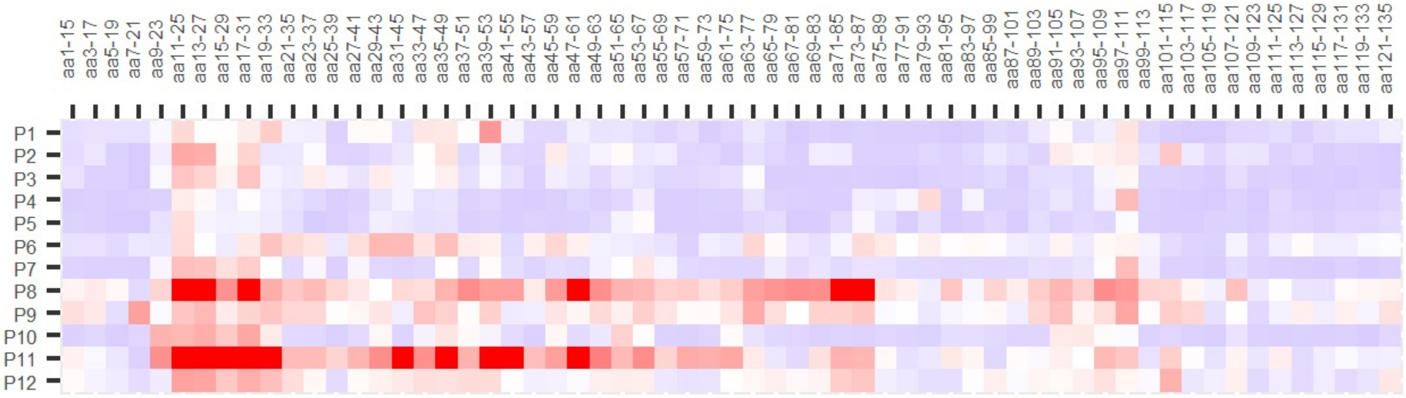

orf6

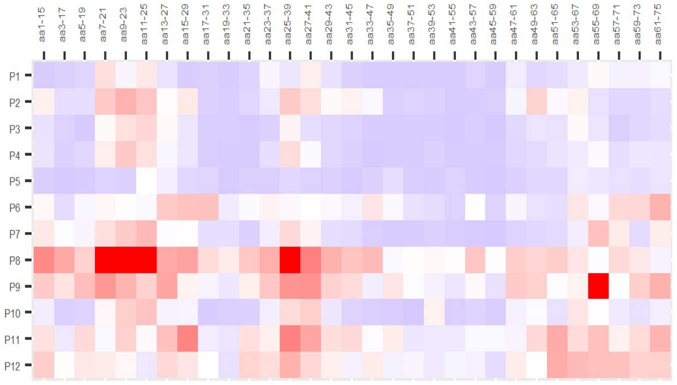

orf10

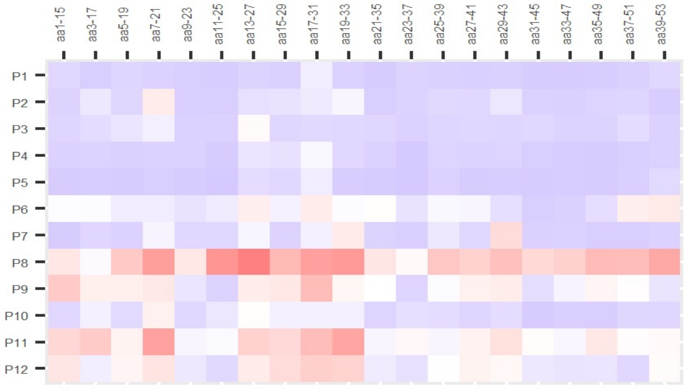

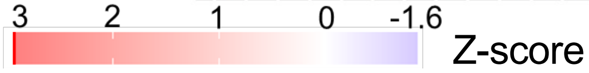

Supplementary Figure S2j: Heatmap showing IgG response for peptide against structural proteins (Spike). The amino acid sequence of the corresponding protein is denoted as “aa1-15” and the COVID-19 patients included in the study are presented as p1-p12. Immunogenic response against the peptide was visualised using Z-score of fluorescence intensity. Peptides with a Z-score greater than 3 in at least one COVID-19 patients was defined as immunogenic.

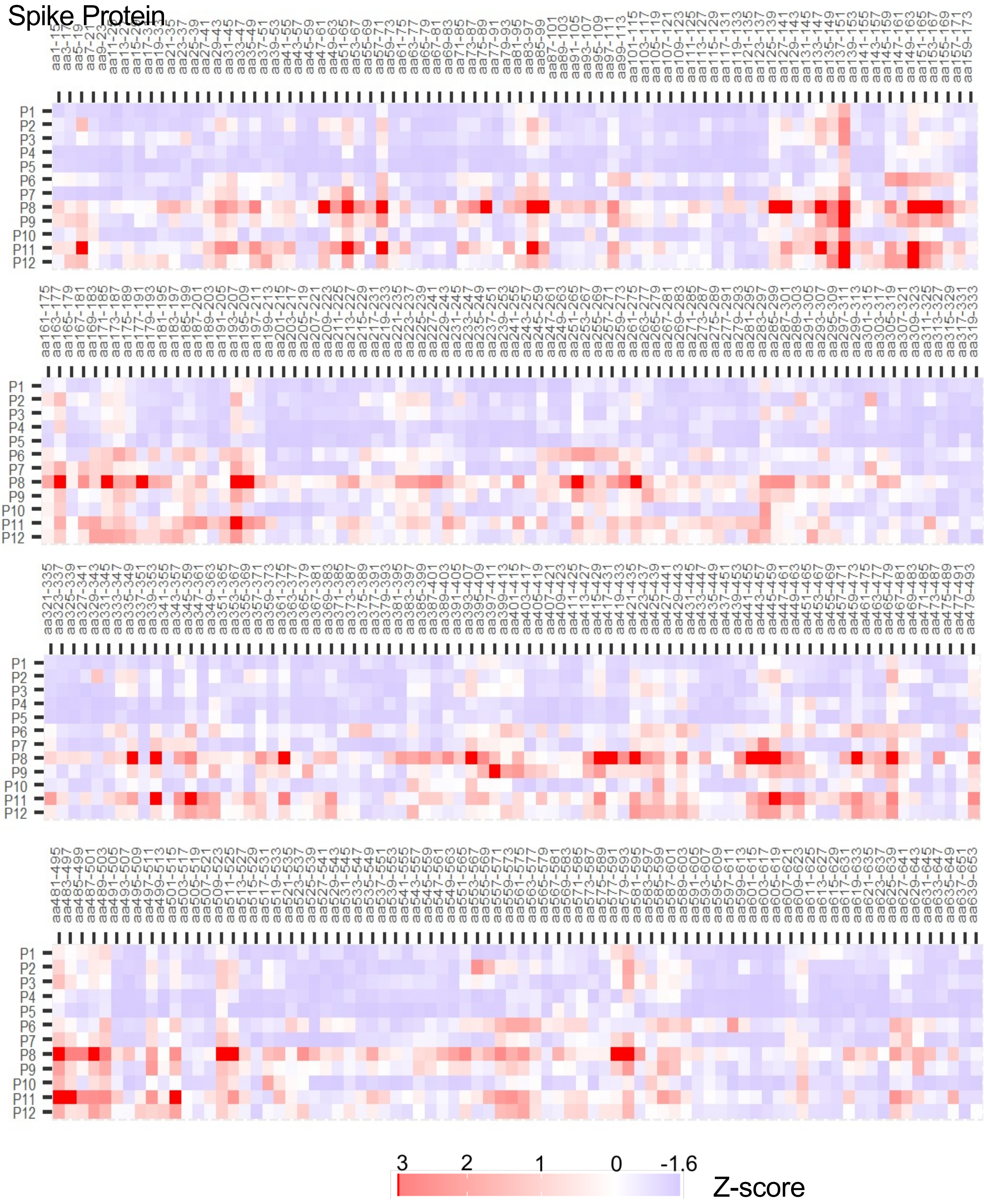

Supplementary Figure S2j contd…

Spike Protein

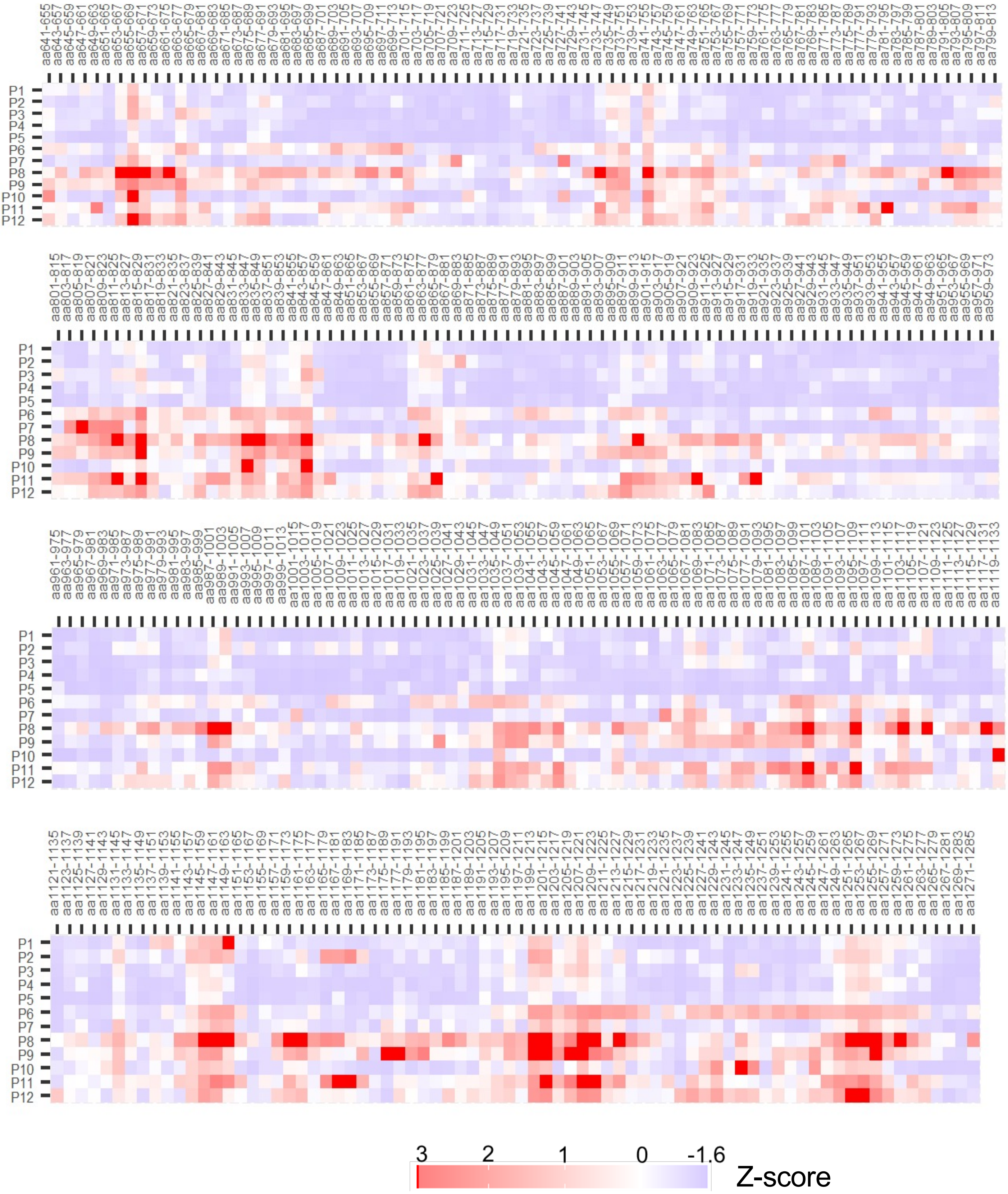

Supplementary Figure S2k: Heatmap showing IgG response for peptide against structural proteins (Nucleocapsid Phosphoprotein). The amino acid sequence of the corresponding protein is denoted as “aa1-15” and the COVID-19 patients included in the study are presented as p1-p12. Immunogenic response against the peptide was visualised using Z-score of fluorescence intensity. Peptides with a Z-score greater than 3 in at least one COVID-19 patients was defined as immunogenic.

Nucleocapsid Phosphoprotein

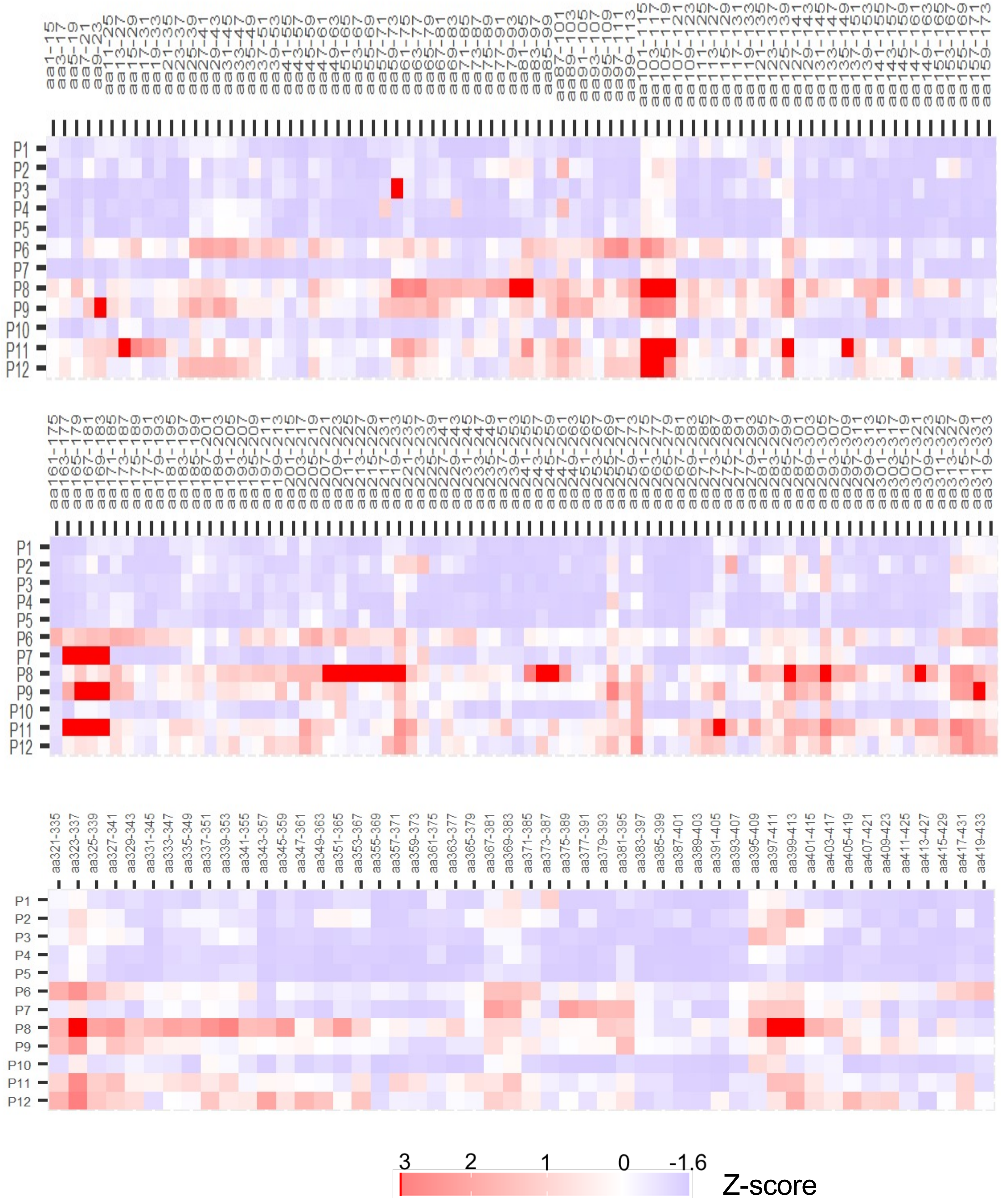

Supplementary Figure S3a: Representative dot plots showing patient-wise comparison of IgG response against mutant and wild type peptides i) Orf1ab Polyprotein, T1638A ii) Spike Protein, D138Y (P.1 Manaus)

i

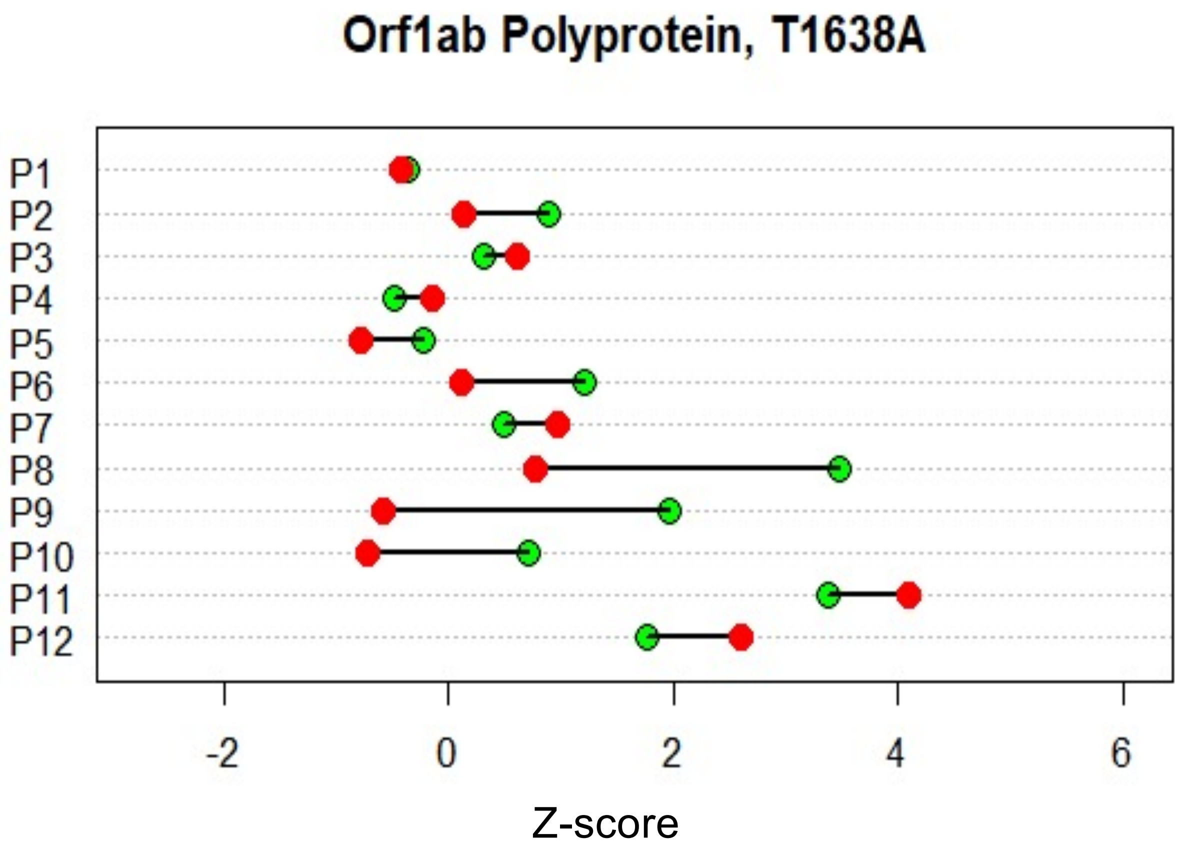

ii

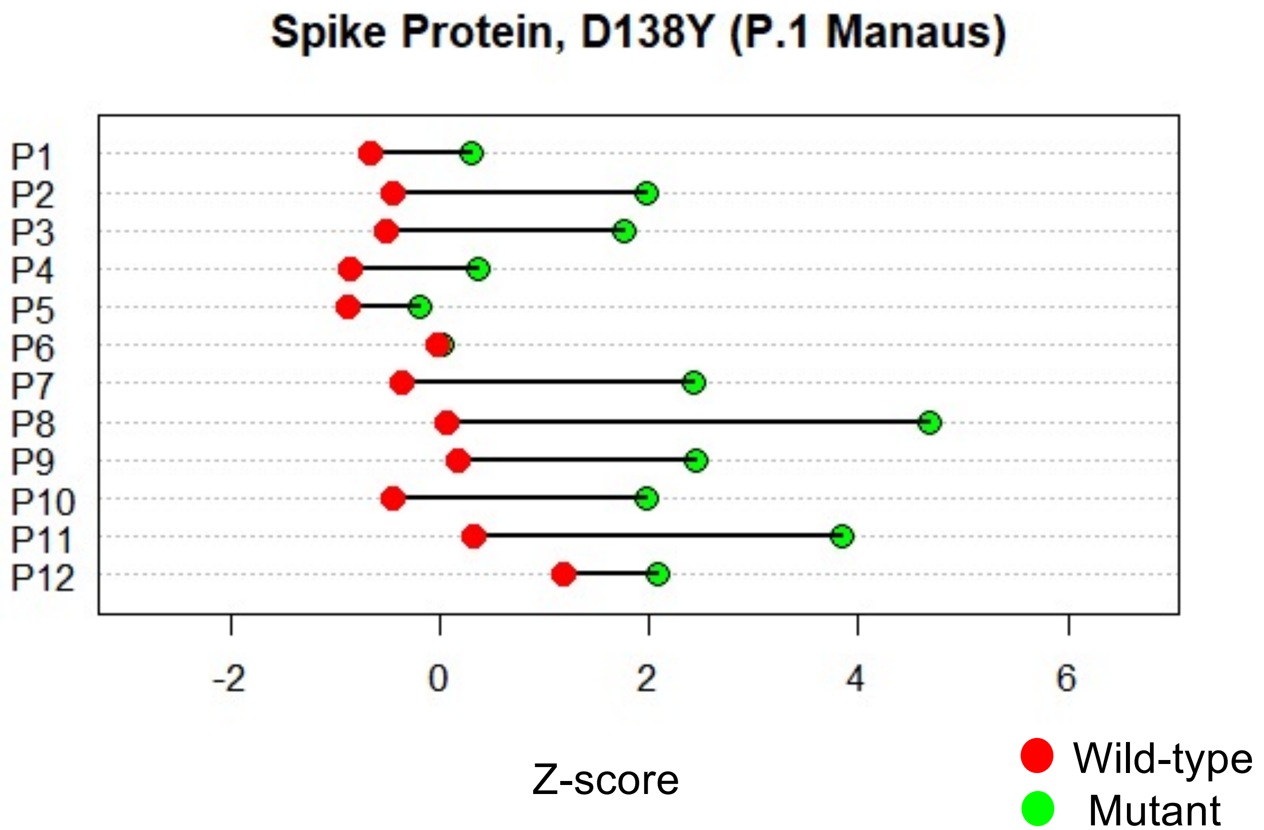

Supplementary Figure S3b: Representative dot plots showing patient-wise comparison of IgG response against mutant and wild type peptides i) Spike Protein, D111H (B1.1.7) ii) Spike Protein, H655Y (P.1 Manaus)

i

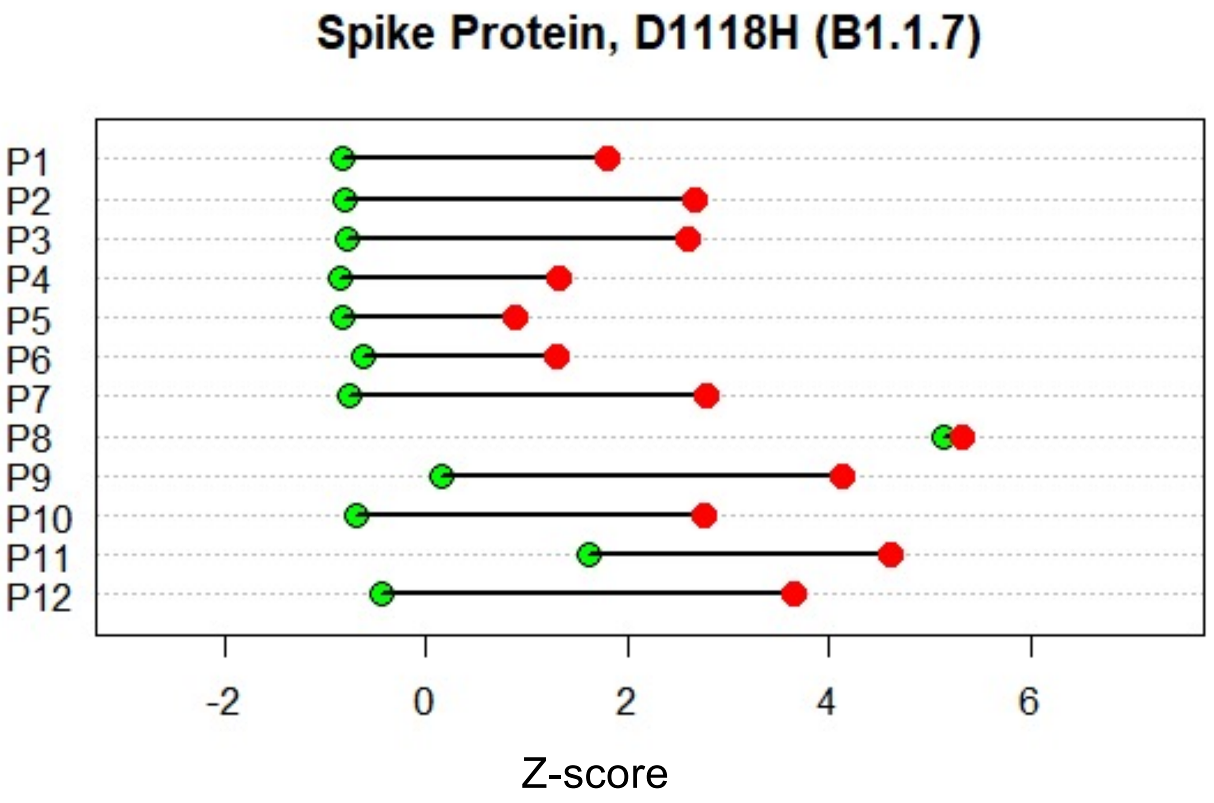

ii

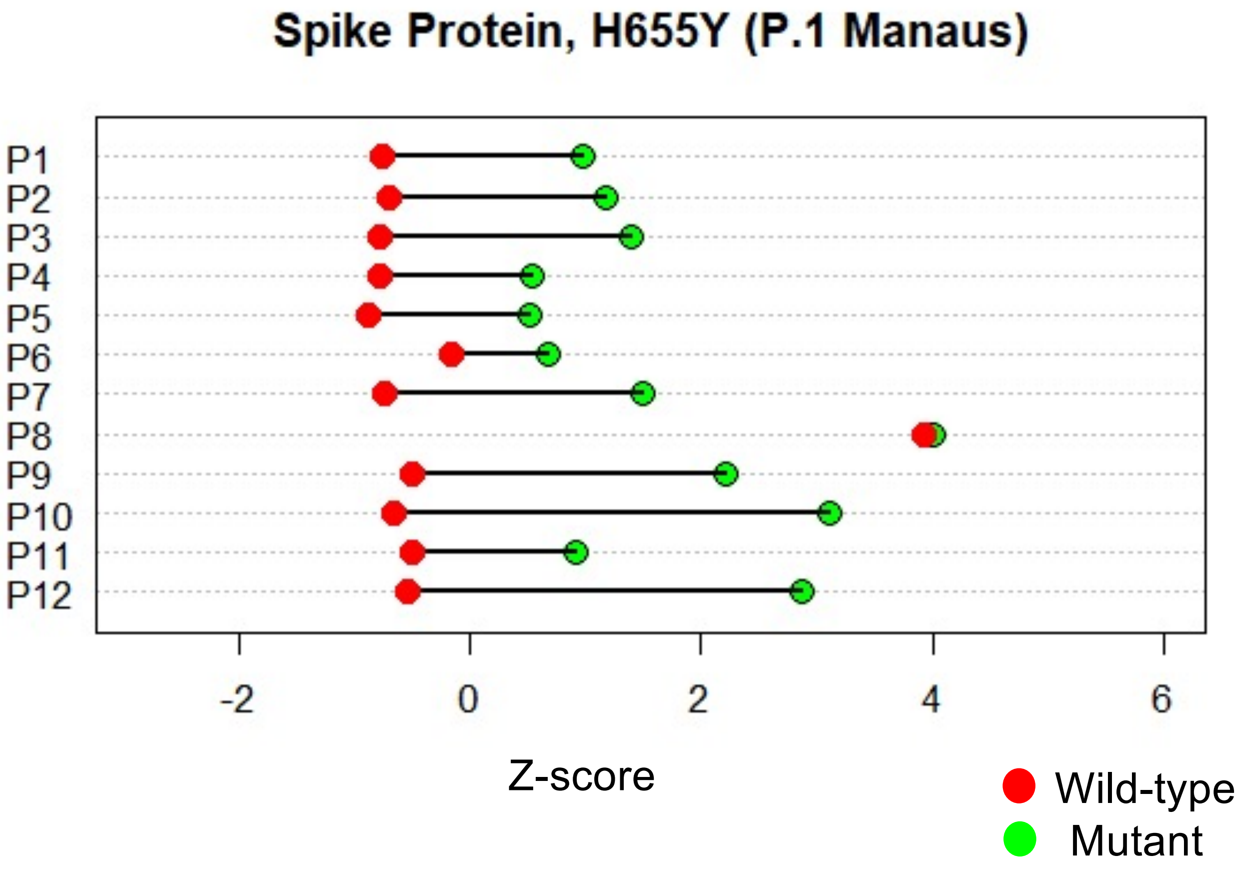

Supplementary Figure S3c: Representative dot plots showing patient-wise comparison of IgG response against mutant and wild type peptides i) Nucleocapsid Phophoprotein, P80R (P.1 Manaus) ii) Orf1ab Polyprotein, V1629A

i

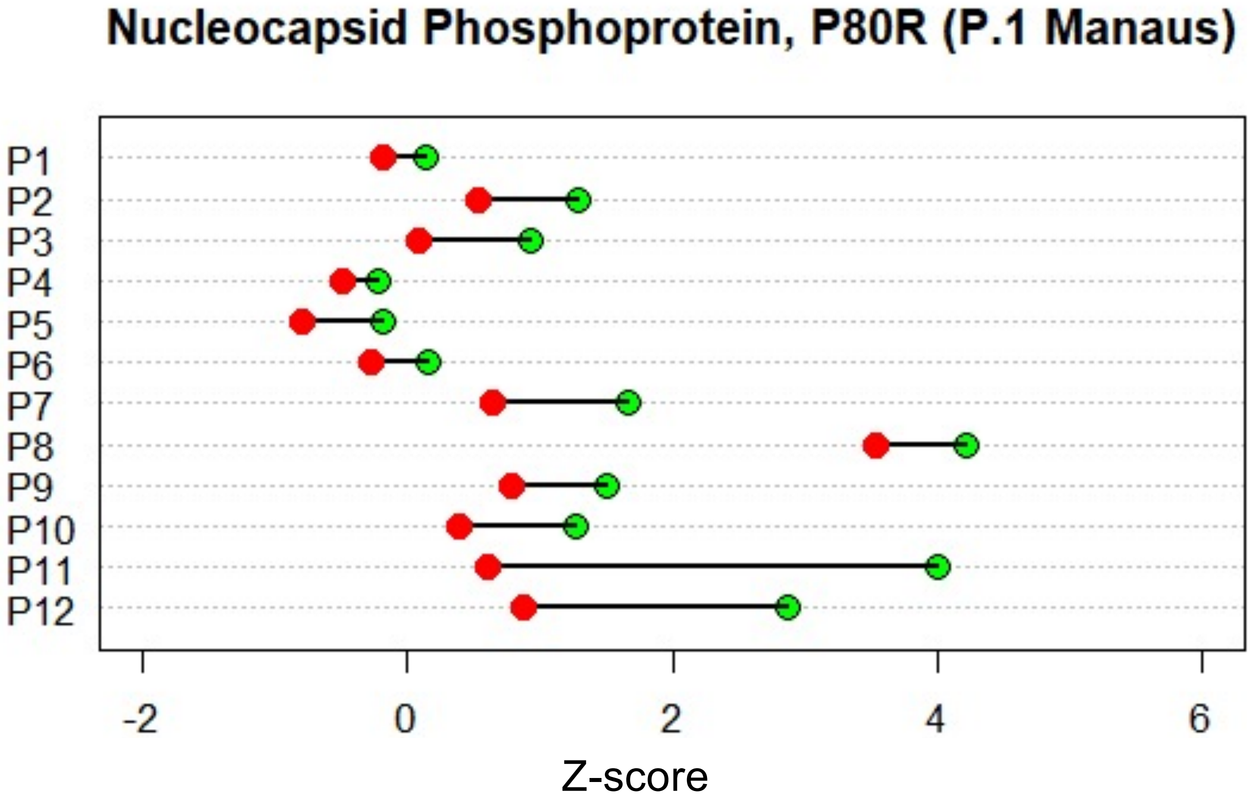

ii

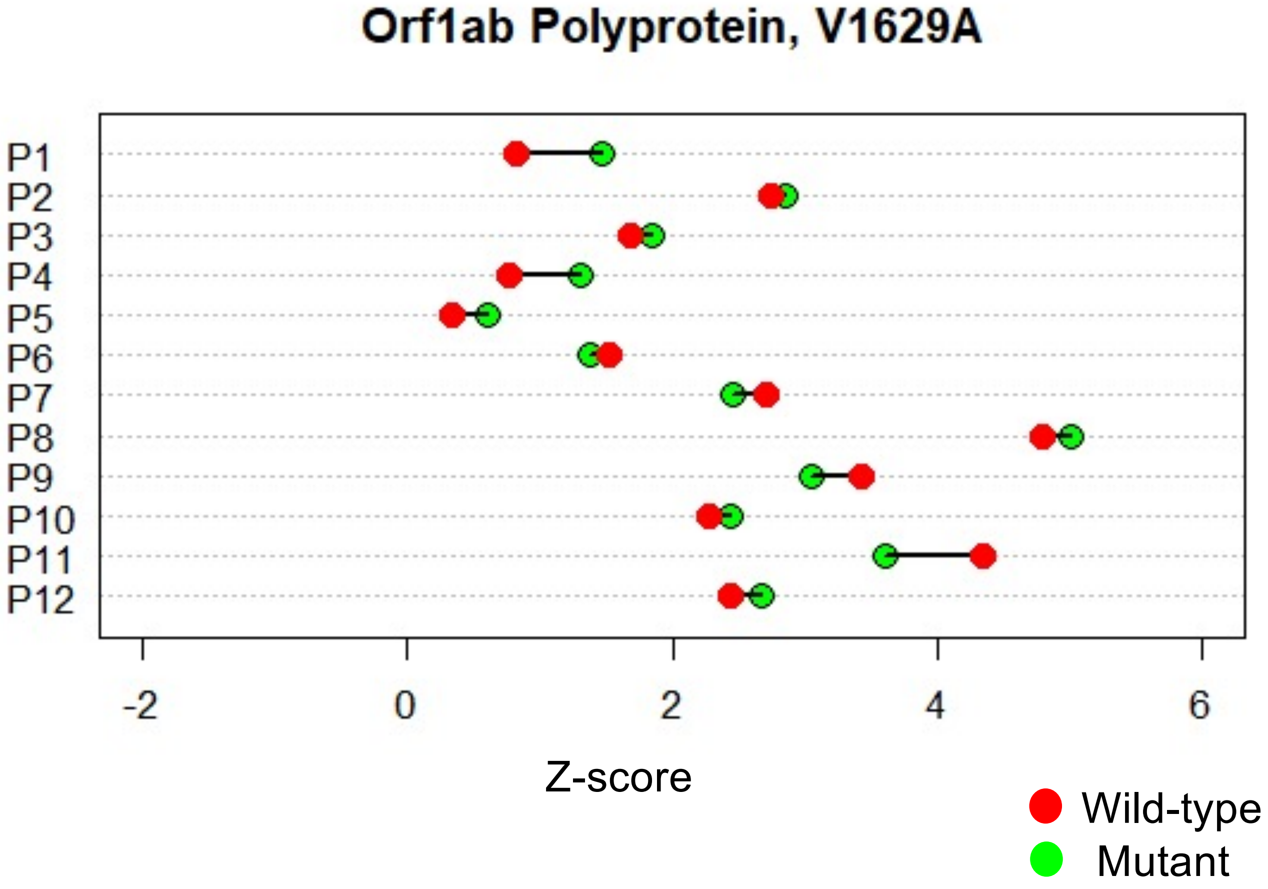

Supplementary Figure S3d: Representative dot plots showing patient-wise comparison of IgG response against mutant and wild type peptides i) Spike Protein, R190S (P.1 Manaus) ii) Spike Protein, R246I (501.V2)

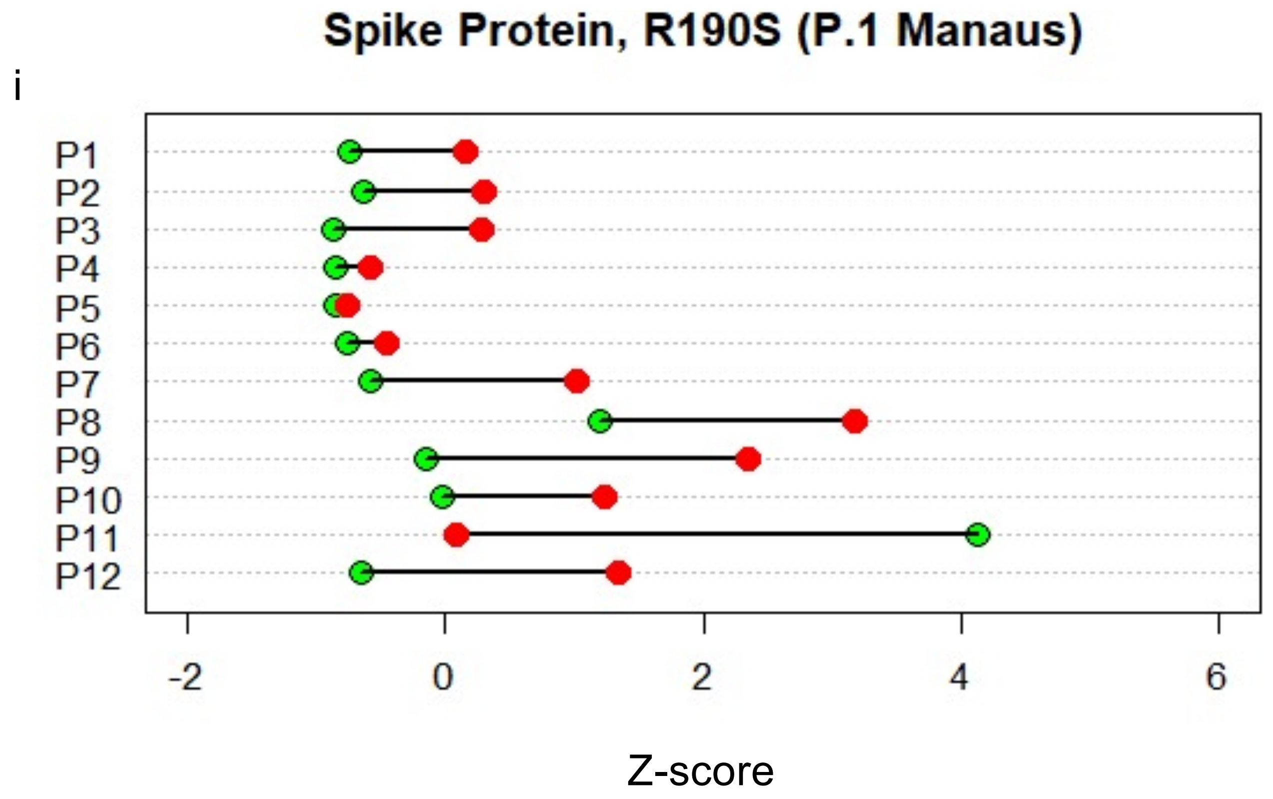

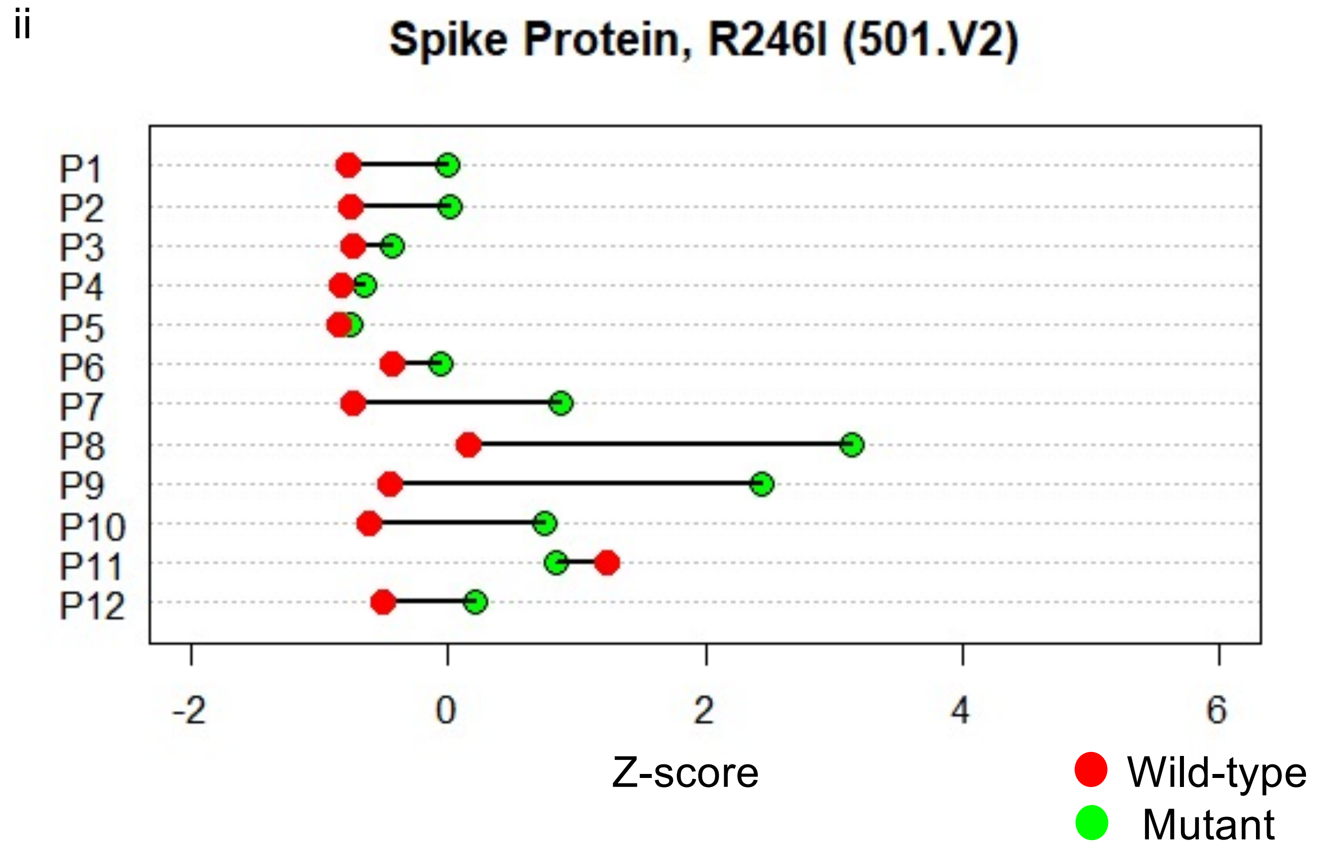

Supplementary Figure S3e: Representative dot plots showing patient-wise comparison of IgG response against mutant and wild type peptides i) Spike Protein, N501Y(B1.1.7) ii) Spike Protein, P26S (P.1 Manaus)

i

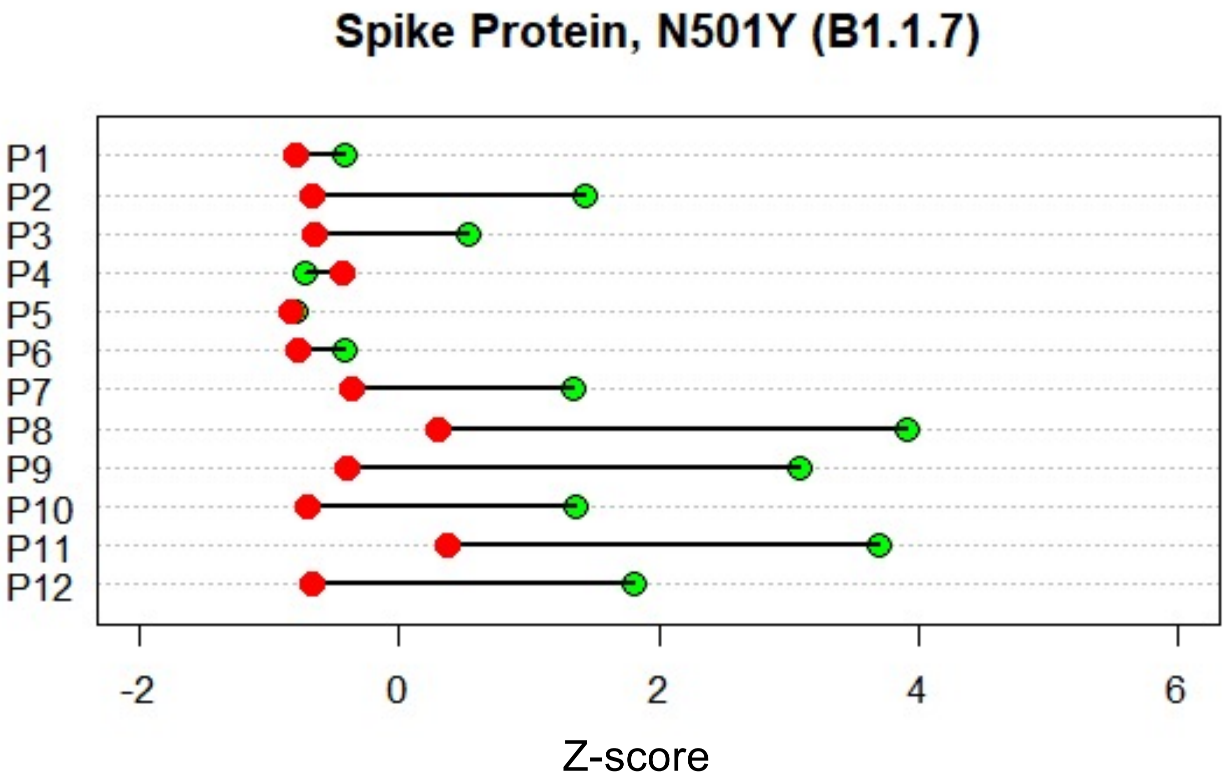

ii

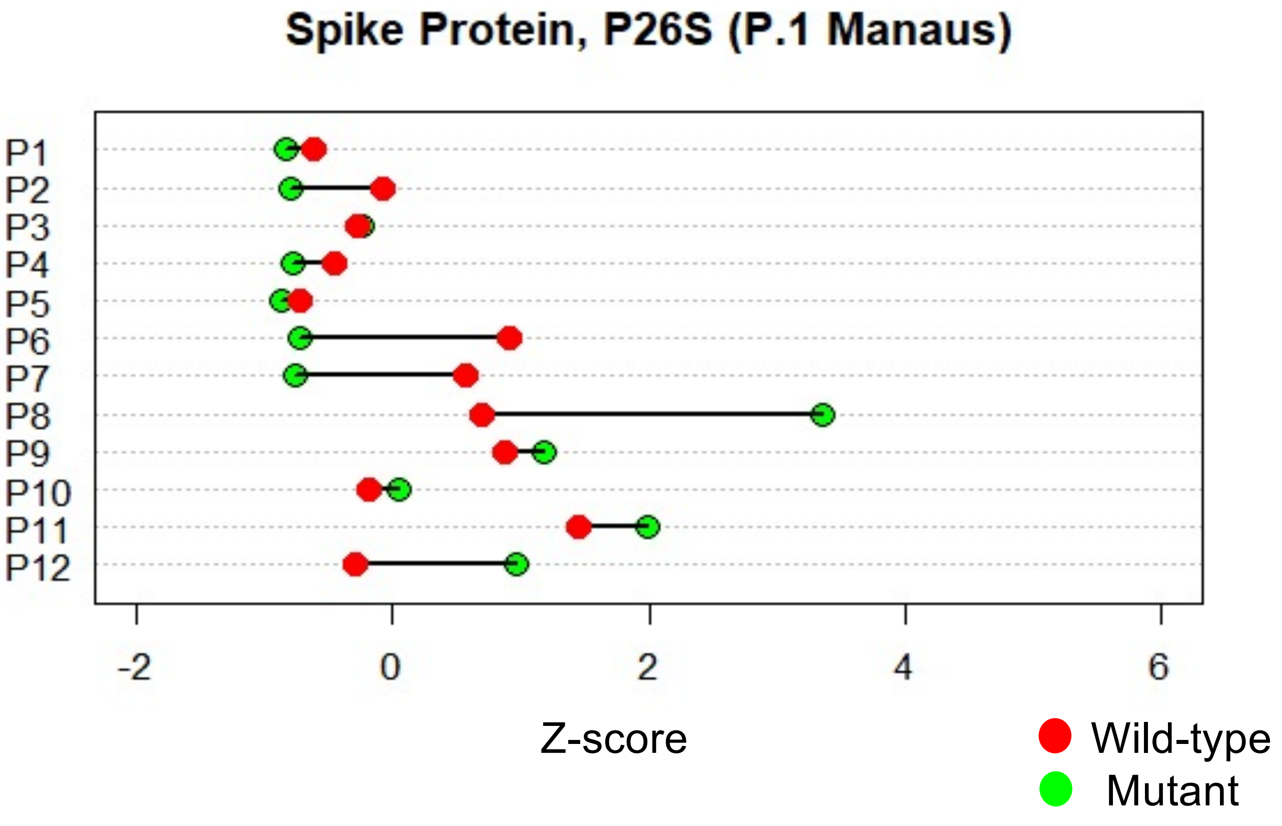

Supplementary Figure S4a: Representative dot plots showing patient-wise comparison of IgA response against mutant and wild type i) Spike Protein, D138Y (P.1 Manaus), ii) Spike Protein, H655Y (P.1 Manaus)

i

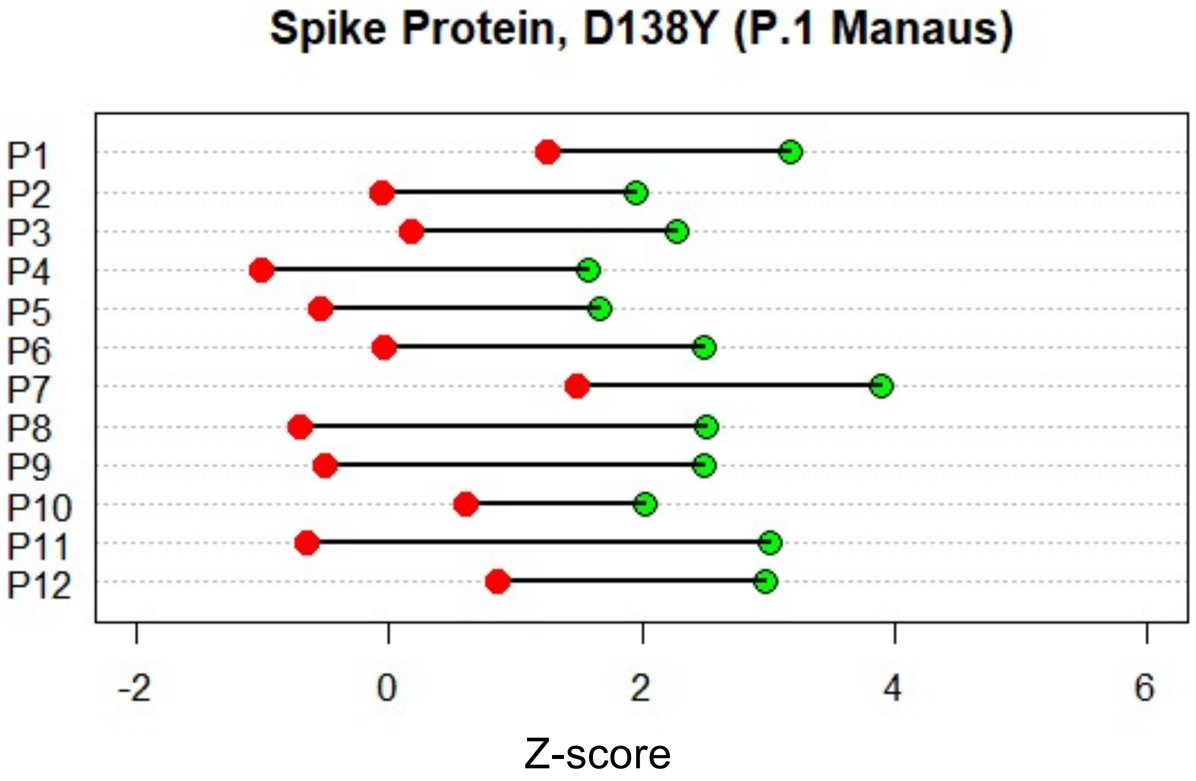

ii

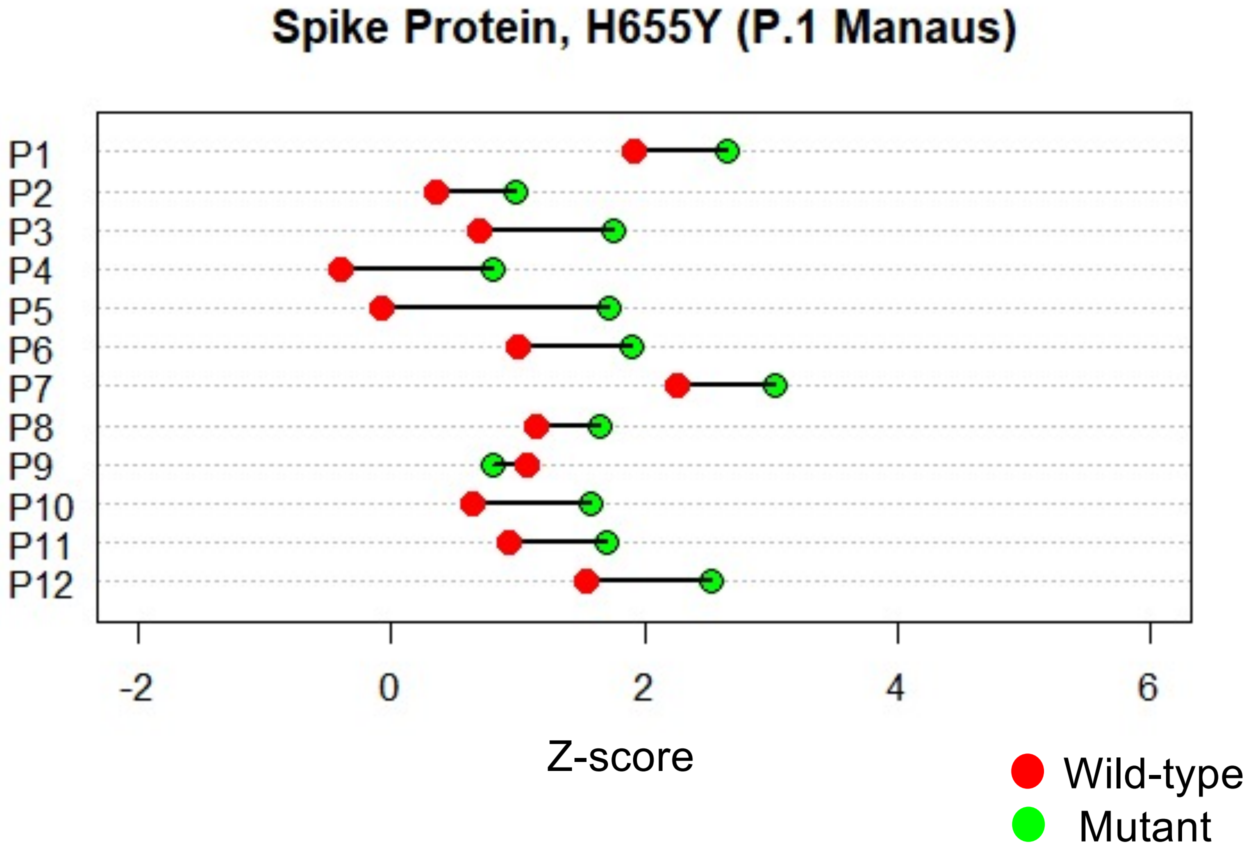

Supplementary Figure S4b: Representative dot plots showing patient-wise comparison of IgA response against mutant and wild type peptides i) Nucleocapsid Phophoprotein, P80R(P.1 Manaus), ii) Orf1ab Polyprotein, V1629A, iii) Spike Protein, N501Y(B1.1.7)

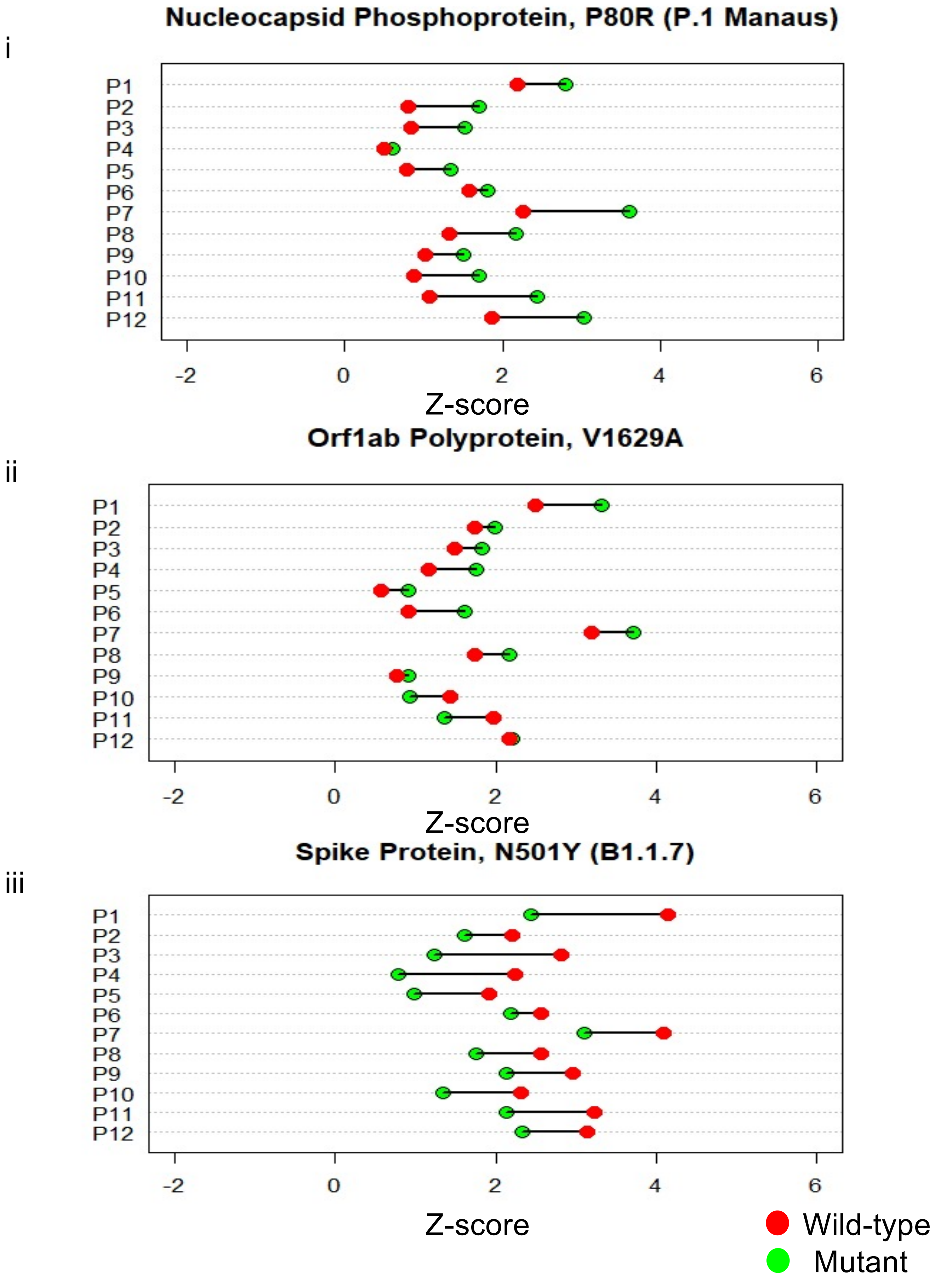

Supplement: Supplementary file 1 [file viruses-15-00248-s001.zip › Supplementary_Figure_Microarray.pdf]
