# Supplementary material for: Humoral Immune Response Profile of COVID-19 Reveals Severity and Variant-Specific Epitopes: Lessons from SARS-CoV-2 Peptide Microarray
Source: Viruses. 2023 Jan 15;15(1):248. doi: 10.3390/v15010248 (PMC9866125; doi:10.3390/v15010248)
Supplement: Supplementary file 1 [file viruses-15-00248-s001.zip › Table S1.docx]

Table S1. Clinical Characteristics of the patients

| Severe | Microarray slide No. | Sample ID | Age | Sex | Date of Hospitalisation | Date of sample collection | Date of Death/discharge | Discharged/Dead | Days between hospitalisation and symptom onset | Days between hospitalisation and sample collection | RT-PCR result | Severity status | SPO2 | Other symptoms | Comorbidities | Ferretin (22-322 ng/ml) | CRP (< 5 mg/l) | D-dimer (<1 ug FEU/ml) | IL-6 (0-7.0 pg/ml) |
| --- | --- | --- | --- | --- | --- | --- | --- | --- | --- | --- | --- | --- | --- | --- | --- | --- | --- | --- | --- |
| I | 002895_01 | 40 | 46 | F | 30/3/2021 | 6/4/2021 | 16/4/2021 | Discharged | 8 | 6 | Positive Severe | Severe | 94% on Bipap with FiO2 50% | B/L Pneumonia | DM | 264.2 | 100 | 5.31 | 34.8 |
| II | 002895_02 | 82 | 68 | F | 19/3/2021 | 1/4/2021 | 19/4/2021 | discharged | 5 | 13 | Positive Severe | Severe | 94% on 8 lit O2 | B/L Pneumonia | None | N/A | N/A | N/A | N/A |
| III | 002895_04 | 42 | 64 | M | 28/3/2021 | 4/4/2021 | 7/5/2021 | Discharged | 3 | 8 | Positive Severe | Severe | 89% on FiO2 85% | B/L Pneumonia | None | 2100 | 100 | 1.41 | 23.1 |
| IV | 002895_05 | 37 | 64 | M | 31/3/2021 | 6/4/2021 | 20/4/2021 | Discharged | 5 | 7 | Positive Severe | Severe | 88% at 12 lt O2 | B/L Crepts, ARDS | none | 409.3 | 100 | 1.47 | 7.2 |
| V | 002895_09 | 85 | 37 | M | 1/4/2021 | 8/4/2021 | 18/4/2021 | Discharged | 8 | 8 | Positive severe | Severe | 99% on 100 FiO2 | Hemoptysis, pneumonia+ARDS | None | N/A | N/A | N/A | N/A |
| VI | 002896_04 | 84 | 65 | F | 2/4/2021 | 12/4/2021 | 1/5/2021 | Death | 10 | 10 | Positive Severe | Severe | 93% ON 100% FiO2 | ARDS | DM & HTN | N/A | N/A | N/A | N/A |
| Non-Severe |  |  |  |  |  |  |  |  |  |  |  |  |  |  |  |  |  |  |  |
| I | 002985_03 | 24 | 58 | M | 3/29/2021 | 1/4/2021 | 6/4/2021 | Discharged | 3 | 4 | Positive | Non-severe | 98% | No | None | N/A | N/A | N/A | N/A |
| II | 002895_06 | 26 | 68 | M | 3/29/2021 | 1/4/2021 | 3/4/2021 | Discharged | 5 | 4 | Positive | Non-severe | 97% | Acidity | None | N/A | N/A | N/A | N/A |
| III | 002895_07 | 54 | 49 | M | 3/30/2021 | 3/4/2021 | 5/4/2021 | Discharged | 4 | 6 | Positive | Non-severe | 97% | Cold | HTN | N/A | N/A | N/A | N/A |
| IV | 002895_08 | 29 | 61 | F | 3/29/2021 | 1/4/2021 | 5/4/2021 | Discharged | 4 | 4 | Positive | Non-severe | 98% | Cold, Bodyache | None | N/A | N/A | N/A | N/A |
| V | 002896_05 | 22 | 52 | M | 3/29/2021 | 1/4/2021 | 4/4/2021 | Discharged | 4 | 4 | Positive | Non-severe | 99% | No | None | N/A | N/A | N/A | N/A |
| VI | 002907_13 | 53 | 48 | M | 3/30/2021 | 3/4/2021 | 5/4/2021 | Discharged | 4 | 6 | Positive | Non-severe | 98% | headache | None | 98.7 | 9.9 | 0.27 | 2.7 |
| Excluded |  |  |  |  |  |  |  |  |  |  |  |  |  |  |  |  |  |  |  |
| I | 002907_12 | 69 | 58 | F | 4/4/2021 | 12/4/2021 | 18/4/2021 | Death |  | 8 | Positive Severe | Severe | 96% on 100 FiO2 | COVID-19 ARDS | None | 560 | 32 | 5.72 | 16.2 |
| II | 002907_14 | 33 | 53 | M | 3/29/2021 | 6/4/2021 | 7/4/2021 | discharged | 5 | 9 | Positive | Non-severe | 98% | No | DM | N/A | N/A | N/A | N/A |
