# Supplementary material for: Humoral Immune Response Profile of COVID-19 Reveals Severity and Variant-Specific Epitopes: Lessons from SARS-CoV-2 Peptide Microarray
Source: Viruses. 2023 Jan 15;15(1):248. doi: 10.3390/v15010248 (PMC9866125; doi:10.3390/v15010248)
Supplement: Supplementary file 1 [file viruses-15-00248-s001.zip › Table S10.docx]

| **Peptide** | **Protein** | **Mutation** |
| --- | --- | --- |
| SEDNQTTIIQTIVEV | Orf1ab Polyprotein | T1001I (B1.1.7) |
| RARAGEADNFCALIL | Orf1ab Polyprotein | A1708D (B1.1.7) |
| FSKLINITIWFLLLS | Orf1ab Polyprotein | I2230T (B1.1.7) |
| DMVDTSLKLKDCVMY | Orf1ab Polyprotein | SGF 3675-3677 deletion (B1.1.7) |
| FQENWNTAASSGVTR | Orf1ab Polyprotein | K164A |
| QENWNTAASSGVTRE | Orf1ab Polyprotein | H165A |
| PNDDTLRAEAFEYYH | Orf1ab Polyprotein | V1629A |
| DTLRVEAAEYYHTTD | Orf1ab Polyprotein | F1632A |
| AFEYYHTADPSFLGR | Orf1ab Polyprotein | T1638A |
| IKWADNNCYLATALL | Orf1ab Polyprotein | C1674S |
| ASEYTGNTQCGHYKH | Orf1ab Polyprotein | Y1831T |
| ASEYTGNGQCGHYKH | Orf1ab Polyprotein | Y1831G |
| LMIERFVALAIDAYP | Orf1ab Polyprotein | S5253A |
| NVTWFHAISGTNGTK | Spike Protein | HV69-79 deletion (B1.1.7) |
| VTWFHAISGTNGTKR | Spike Protein | HV69-79 deletion (B1.1.7) |
| NDPFLGVYHKNNKSW | Spike Protein | Y144 deletion (B1.1.7) |
| SYGFQPTYGVGYQPY | Spike Protein | N501Y (B1.1.7) |
| QQFGRDIDDTTDAVR | Spike Protein | A570D (B1.1.7) |
| YQTQTNSHRRARSVA | Spike Protein | P681H (B1.1.7) |
| LQYGSFCIQLNRALT | Spike Protein | T761T (B1.1.7) |
| SVLNDILARLDKVEA | Spike Protein | S982A (B1.1.7) |
| EPQIITTHNTFVSGN | Spike Protein | D1118H (B1.1.7) |
| QVAVLYQGVNCTEVP | Spike Protein | D614G (Dominant European variant) |
| GGNYNYLFRLFRKSN | Spike Protein | Y453F (Cluster 5/Denmark) |
| WYIRVGAIKSAPLIE | Orf8 Protein | R52I (B1.1.7) |
| GSKSPIQCIDIGNYT | Orf8 Protein | Y73C (B1.1.7) |
| LFSTVFPLTSFGPLV | Orf1ab Polyprotein | P323L (B1.1.7) |
| MSLNGPQNQRNAPRI | Nucleocapsid Phosphoprotein | D3L (B1.1.7) |
| NQLESKMFGKGQQQQ | Nucleocapsid Phosphoprotein | S235F (B1.1.7) |
| TNGTKRFANPVLPFN | Spike Protein | D80A (501.V2) |
| TPINLVRGLPQGFSA | Spike Protein | D215G (501.V2) |
| STPCNGVKGFNCYFP | Spike Protein | E484K (501.V2, B1.1.28, P.1 Manaus) |
| AYTMSLGVENSVAYS | Spike Protein | A701V (501.V2) |
| VSSQCVNFTTRTQLP | Spike Protein | L18F (501.V2) |
| QTLLALHISYLTPGD | Spike Protein | R246I (501.V2) |
| IAPGQTGNIADYNYK | Spike Protein | K417N (501.V2) |
| SYGFQPTKGVGYQPY | Spike Protein | N501K (P.1 Manaus) |
| IAPGQTGTIADYNYK | Spike Protein | K417T (P.1 Manaus) |
| SQCVNLTNRTQLPPA | Spike Protein | T20N (P.1 Manaus) |
| TTRTQLPSAYTNSFT | Spike Protein | P26S (P.1 Manaus) |
| CEFQFCNYPFLGVYY | Spike Protein | D138Y (P.1 Manaus) |
| QGNFKNLSEFVFKNI | Spike Protein | R190S (P.1 Manaus) |
| GCLIGAEYVNNSYEC | Spike Protein | H655Y (P.1 Manaus) |
| ASANLAAIKMSECVL | Spike Protein | T1027I (P.1 Manaus) |
| PFTINCQKPKLGSLV | Orf8 Protein | E92K (P.1 Manaus) |
| PINTNSSRDDQIGYY | Nucleocapsid Phosphoprotein | P80R (P.1 Manaus) |
| NLYDKLVLSFLEMKS | Orf1ab Polyprotein | S1188L (P.1 Manaus) |
| TCGKQATQYLVQQES | Orf1ab Polyprotein | K1795Q (P.1 Manaus) |
| IPARARVDCFDKFKV | Orf1ab Polyprotein | E5665D (P.1 Manaus) |

Table S10: List of peptides with mutations
