# Supplementary material for: Humoral Immune Response Profile of COVID-19 Reveals Severity and Variant-Specific Epitopes: Lessons from SARS-CoV-2 Peptide Microarray
Source: Viruses. 2023 Jan 15;15(1):248. doi: 10.3390/v15010248 (PMC9866125; doi:10.3390/v15010248)
Supplement: Supplementary file 1 [file viruses-15-00248-s001.zip › Table S2.docx]

Table S2. IgA reactive peptides from the SARS-CoV-2 proteome

| Protein | Sequence | Peptide | NS1 | NS2 | NS3 | NS4 | NS5 | NS6 | SV1 | SV2 | SV3 | SV4 | SV5 | SV6 |
| --- | --- | --- | --- | --- | --- | --- | --- | --- | --- | --- | --- | --- | --- | --- |
| nsp1 | aa121-135 | IPVAYRKVLLRKNGN | 0.881 | -1.140 | -0.401 | -1.522 | -1.010 | -0.929 | 0.631 | 3.605 | 0.458 | -0.723 | -0.801 | -1.270 |
| nsp1 | aa147-161 | LKSFDLGDELGTDPY | 2.530 | 1.030 | 1.150 | 1.009 | 1.310 | 1.582 | 3.058 | 1.735 | 0.383 | 1.125 | 1.754 | 2.260 |
| nsp1 | aa149-163 | SFDLGDELGTDPYED | 3.670 | 1.350 | 1.760 | 1.834 | 1.630 | 2.225 | 3.697 | 1.615 | -0.466 | 1.279 | 1.425 | 2.690 |
| nsp1 | aa151-165 | DLGDELGTDPYEDFQ | 3.260 | 1.140 | 1.300 | 1.212 | 1.020 | 1.432 | 3.435 | 1.692 | 0.552 | 0.771 | 1.298 | 2.370 |
| nsp2 | aa15-29 | YTRYVDNNFCGPDGY | 2.850 | 1.330 | 1.320 | 0.418 | 0.898 | 1.609 | 3.081 | 1.613 | 1.615 | 1.422 | 2.276 | 2.230 |
| nsp2 | aa295-309 | NIVGDFKLNEEIAII | 3.200 | 0.615 | -0.027 | -0.424 | 0.529 | -0.471 | 2.646 | 1.283 | 0.558 | 0.623 | 0.870 | 1.740 |
| nsp2 | aa465-479 | EEKFKEGVEFLRDGW | 2.450 | 1.390 | 1.220 | 0.348 | 1.390 | 1.886 | 3.367 | 1.876 | 2.073 | 1.072 | 2.513 | 2.700 |
| nsp2 | aa467-481 | KFKEGVEFLRDGWEI | 2.360 | 1.400 | 1.080 | 0.466 | 1.380 | 1.344 | 3.173 | 1.962 | 2.227 | 1.095 | 2.278 | 2.860 |
| nsp3 | aa23-37 | DTVIEVQGYKSVNIT | -0.430 | -0.866 | -0.893 | -1.149 | -1.280 | -1.066 | 3.227 | -0.865 | -1.439 | -0.930 | -0.214 | 0.261 |
| nsp3 | aa81-95 | VSELLTPLGIDLDEW | 3.670 | 2.260 | 1.880 | 1.318 | 1.580 | 2.321 | 3.633 | 2.576 | 1.842 | 1.708 | 2.618 | 3.200 |
| nsp3 | aa87-101 | PLGIDLDEWSMATYY | 3.870 | 2.960 | 2.010 | 1.481 | 1.770 | 2.600 | 4.292 | 2.847 | 2.671 | 2.445 | 3.373 | 3.170 |
| nsp3 | aa89-103 | GIDLDEWSMATYYLF | 3.690 | 2.840 | 2.070 | 1.516 | 1.860 | 2.704 | 3.964 | 2.631 | 2.567 | 2.338 | 3.257 | 3.140 |
| nsp3 | aa91-105 | DLDEWSMATYYLFDE | 3.950 | 2.200 | 2.320 | 1.699 | 1.490 | 2.454 | 3.766 | 2.300 | 1.985 | 1.728 | 2.576 | 3.020 |
| nsp3 | aa95-109 | WSMATYYLFDESGEF | 3.580 | 1.840 | 2.110 | 1.291 | 1.180 | 1.752 | 3.636 | 2.137 | 2.498 | 1.691 | 2.598 | 2.860 |
| nsp3 | aa117-131 | CSFYPPDEDEEEGDC | 3.620 | 1.660 | 1.940 | 1.658 | 1.080 | 1.884 | 3.415 | 1.706 | 1.161 | 1.955 | 0.396 | 2.630 |
| nsp3 | aa119-133 | FYPPDEDEEEGDCEE | 3.810 | 2.010 | 1.960 | 1.701 | 1.610 | 2.100 | 3.532 | 1.700 | 0.630 | 2.116 | 0.851 | 2.500 |
| nsp3 | aa121-135 | PPDEDEEEGDCEEEE | 3.910 | 2.380 | 1.990 | 1.627 | 1.600 | 2.256 | 3.104 | 1.732 | 0.083 | 2.204 | 1.453 | 2.610 |
| nsp3 | aa123-137 | DEDEEEGDCEEEEFE | 4.180 | 2.360 | 2.210 | 2.022 | 1.810 | 2.603 | 3.410 | 2.035 | 0.415 | 2.421 | 2.129 | 2.760 |
| nsp3 | aa129-143 | GDCEEEEFEPSTQYE | 3.170 | 1.980 | 2.130 | 1.504 | 1.190 | 2.177 | 3.256 | 1.911 | 1.058 | 1.270 | 1.842 | 1.970 |
| nsp3 | aa131-145 | CEEEEFEPSTQYEYG | 3.620 | 1.930 | 1.950 | 1.599 | 1.400 | 2.148 | 3.395 | 1.995 | 1.237 | 1.561 | 2.418 | 2.320 |
| nsp3 | aa133-147 | EEEFEPSTQYEYGTE | 2.300 | 1.560 | 1.760 | 0.739 | 1.130 | 2.387 | 3.007 | 0.246 | -0.025 | 1.215 | 2.596 | 2.210 |
| nsp3 | aa161-175 | SAALQPEEEQEEDWL | 3.300 | 1.920 | 1.620 | 1.591 | 1.160 | 2.108 | 3.057 | 1.862 | 0.810 | 1.752 | 2.063 | 2.440 |
| nsp3 | aa163-177 | ALQPEEEQEEDWLDD | 3.910 | 1.990 | 1.830 | 2.007 | 1.310 | 2.199 | 3.497 | 1.787 | 0.328 | 2.040 | 1.979 | 1.990 |
| nsp3 | aa165-179 | QPEEEQEEDWLDDDS | 3.430 | 1.720 | 1.610 | 1.917 | 1.210 | 2.142 | 3.264 | 1.361 | 0.547 | 1.504 | 2.050 | 1.990 |
| nsp3 | aa365-379 | RTNVYLAVFDKNLYD | 3.150 | 0.593 | 1.290 | 0.116 | 0.277 | 1.058 | 3.006 | 1.930 | 2.114 | 0.178 | 2.018 | 2.020 |
| nsp3 | aa437-451 | TLEETKFLTENLLLY | 2.800 | 1.650 | 2.030 | 1.681 | 1.390 | 2.025 | 3.213 | 1.845 | 1.586 | 1.191 | 2.266 | 2.130 |
| nsp3 | aa439-453 | EETKFLTENLLLYID | 3.560 | 1.590 | 1.910 | 1.964 | 0.886 | 1.798 | 3.552 | 1.805 | 1.280 | 0.956 | 2.213 | 2.020 |
| nsp3 | aa443-457 | FLTENLLLYIDINGN | 1.220 | 0.477 | 0.268 | 0.017 | -0.844 | -0.201 | 1.493 | 3.459 | 0.991 | 0.546 | 0.751 | 0.485 |
| nsp3 | aa605-619 | KIQEGVVDYGARFYF | 2.650 | 1.490 | 0.586 | 0.903 | 0.983 | 2.208 | 3.232 | 1.768 | 2.185 | 1.398 | 2.553 | 2.560 |
| nsp3 | aa815-829 | VLPNDDTLRVEAFEY | 3.130 | 2.080 | 1.910 | 1.686 | 1.310 | 1.609 | 3.241 | 1.863 | 0.894 | 1.564 | 2.383 | 2.630 |
| nsp3 | aa817-831 | PNDDTLRVEAFEYYH | 2.490 | 1.730 | 1.480 | 1.166 | 0.568 | 0.912 | 3.196 | 1.740 | 0.769 | 1.422 | 1.978 | 2.160 |
| nsp3 | aa827-841 | FEYYHTTDPSFLGRY | 2.320 | 1.550 | 1.600 | 0.859 | 0.453 | 1.356 | 3.166 | 1.840 | 1.289 | 1.527 | 2.694 | 2.410 |
| nsp3 | aa881-895 | IELKFNPPALQDAYY | 3.470 | 1.910 | 2.040 | 0.835 | 1.210 | 1.600 | 3.660 | 2.373 | 2.209 | 1.942 | 2.612 | 2.670 |
| nsp3 | aa899-913 | AGEAANFCALILAYC | 3.090 | 0.745 | 1.510 | 1.046 | 0.893 | 1.350 | 2.658 | 1.957 | 2.247 | 1.307 | 2.712 | 2.460 |
| nsp3 | aa1011-1025 | LKHGTFTCASEYTGN | 0.839 | -0.102 | 0.301 | -1.168 | -1.160 | -0.369 | 0.633 | 3.313 | 1.198 | 0.658 | -0.164 | 1.040 |
| nsp3 | aa1057-1071 | PITDVFYKENSYTTT | 0.455 | 2.640 | -0.119 | 0.792 | 0.857 | 1.284 | 3.219 | -0.618 | -0.986 | 0.897 | 0.479 | 1.290 |
| nsp3 | aa1081-1095 | GVVCTEIDPKLDNYY | 3.320 | 2.250 | 1.510 | 1.622 | 0.956 | 1.552 | 3.542 | 2.234 | 1.488 | 1.655 | 2.724 | 2.830 |
| nsp3 | aa1153-1167 | TFFPDLNGDVVAIDY | 3.360 | 1.190 | 2.120 | 0.484 | 0.747 | 1.365 | 3.362 | 1.916 | 1.930 | 1.065 | 1.838 | 2.600 |
| nsp3 | aa1281-1295 | ITEEVGHTDLMAAYV | 2.380 | 0.725 | 0.610 | -0.393 | -0.428 | 0.527 | 2.304 | 1.223 | 1.100 | 0.710 | 1.121 | 3.250 |
| nsp3 | aa1509-1523 | FKWDLTAFGLVAEWF | 3.120 | 1.980 | 1.510 | 1.250 | 1.230 | 2.840 | 3.882 | 2.211 | 2.589 | 1.963 | 2.634 | 2.790 |
| nsp3 | aa1511-1525 | WDLTAFGLVAEWFLA | 2.810 | 1.510 | 0.934 | 0.837 | 0.859 | 2.291 | 3.130 | 0.450 | 2.318 | 1.456 | 2.202 | 2.730 |
| nsp3 | aa1513-1527 | LTAFGLVAEWFLAYI | 3.170 | 1.630 | 1.270 | 0.691 | 1.070 | 2.600 | 3.425 | 1.431 | 2.466 | 1.810 | 2.534 | 2.570 |
| nsp3 | aa1515-1529 | AFGLVAEWFLAYILF | 2.260 | 1.170 | 0.899 | 0.063 | 0.871 | 2.530 | 3.339 | 1.464 | 2.239 | 1.379 | 2.564 | 2.430 |
| nsp3 | aa1535-1549 | VLGLAAIMQLFFSYF | 2.260 | 1.140 | 0.695 | 0.519 | 0.680 | 1.705 | 3.078 | 1.437 | 2.016 | 1.365 | 2.423 | 2.580 |
| nsp3 | aa1547-1561 | SYFAVHFISNSWLMW | 2.110 | 1.210 | 0.870 | 0.432 | 0.769 | 2.368 | 3.134 | 1.154 | 1.880 | 0.938 | 2.419 | 2.410 |
| nsp3 | aa1549-1563 | FAVHFISNSWLMWLI | 2.360 | 0.646 | 0.703 | 0.072 | 0.385 | 1.877 | 3.042 | 1.354 | 1.286 | 1.258 | 2.274 | 2.160 |
| nsp3 | aa1573-1587 | SAMVRMYIFFASFYY | 3.080 | 1.640 | 1.340 | 0.992 | 1.440 | 2.374 | 3.205 | 2.142 | 2.623 | 1.892 | 2.989 | 2.540 |
| nsp3 | aa1575-1589 | MVRMYIFFASFYYVW | 2.950 | 0.968 | 0.565 | 0.395 | 1.090 | 2.533 | 3.085 | 1.681 | 2.126 | 1.248 | 2.692 | 2.330 |
| nsp3 | aa1675-1689 | PINPTDQSSYIVDSV | 1.080 | 2.890 | 0.025 | -0.352 | 0.005 | 0.782 | 3.766 | 0.182 | 0.545 | 1.945 | 1.186 | 2.150 |
| nsp4 | aa61-75 | DTCFANKHADFDTWF | 2.330 | 1.690 | 1.450 | 1.280 | 0.393 | 2.221 | 3.465 | 2.129 | 1.407 | 1.346 | 2.472 | 2.710 |
| nsp4 | aa145-159 | FATSACVLAAECTIF | 2.160 | 0.921 | 0.891 | 1.616 | 0.652 | 1.489 | 3.110 | 1.500 | 1.675 | 0.733 | 1.590 | 1.130 |
| nsp4 | aa207-221 | EGSVRVVTTFDSEYC | 3.880 | 2.030 | 2.050 | 1.494 | 1.390 | 2.324 | 3.712 | 2.174 | 2.082 | 2.171 | 2.325 | 2.710 |
| nsp4 | aa339-353 | PGVYSVIYLYLTFYL | 2.350 | 0.964 | 0.762 | 0.101 | 0.962 | 3.028 | 2.516 | 1.606 | 2.013 | 1.343 | 2.633 | 2.340 |
| nsp4 | aa379-393 | IAYIICISTKHFYWF | 2.300 | 0.014 | -0.447 | -0.234 | 0.442 | 1.660 | 3.046 | 1.200 | 1.284 | 0.771 | 2.080 | 1.770 |
| nsp4 | aa407-421 | VSFSTFEEAALCTFL | 1.600 | 1.140 | 0.711 | 1.768 | 0.644 | 1.187 | 3.135 | 1.637 | 1.450 | 0.654 | 1.625 | 0.459 |
| nsp5 | aa23-37 | GTTTLNGLWLDDVVY | 3.550 | 1.310 | 2.570 | 1.633 | 1.080 | 1.772 | 3.543 | 2.030 | 2.121 | 1.319 | 2.509 | 2.540 |
| nsp5 | aa25-39 | TTLNGLWLDDVVYCP | 3.230 | 0.853 | 2.230 | 1.482 | 0.657 | 0.963 | 2.131 | 1.729 | 1.683 | 1.085 | 2.252 | 1.980 |
| nsp5 | aa141-155 | LNGSCGSVGFNIDYD | 3.700 | 1.420 | 1.910 | 0.088 | 1.040 | 1.619 | 3.667 | 2.077 | 1.592 | 1.653 | 2.064 | 3.020 |
| nsp5 | aa143-157 | GSCGSVGFNIDYDCV | 3.430 | 1.580 | 2.160 | 0.661 | 1.110 | 1.921 | 3.507 | 1.943 | 1.946 | 1.856 | 2.341 | 2.730 |
| nsp5 | aa145-159 | CGSVGFNIDYDCVSF | 3.570 | 1.680 | 2.280 | 1.478 | 1.310 | 2.195 | 3.981 | 2.327 | 2.296 | 1.792 | 2.631 | 2.560 |
| nsp5 | aa147-161 | SVGFNIDYDCVSFCY | 4.530 | 2.380 | 3.020 | 2.265 | 1.830 | 2.461 | 4.306 | 2.559 | 2.791 | 2.365 | 3.102 | 2.920 |
| nsp5 | aa205-219 | LAWLYAAVINGDRWF | 1.910 | 1.560 | 1.080 | 0.956 | 0.445 | 1.895 | 3.214 | 2.092 | 1.701 | 1.518 | 2.465 | 1.540 |
| nsp5 | aa277-291 | NGRTILGSALLEDEF | 2.850 | 0.342 | 1.360 | 0.578 | 1.070 | 1.209 | 3.068 | 1.252 | 0.578 | 1.016 | 1.216 | 2.270 |
| nsp5 | aa281-295 | ILGSALLEDEFTPFD | 3.060 | 0.914 | 1.550 | 0.355 | 0.820 | 0.738 | 3.083 | 1.351 | 0.546 | 1.195 | 0.811 | 1.580 |
| nsp6 | aa25-39 | LVQSTQWSLFFFLYE | 3.460 | 1.780 | 2.100 | 2.102 | 1.250 | 2.335 | 3.670 | 1.947 | 1.687 | 1.612 | 2.650 | 2.130 |
| nsp6 | aa183-197 | MFLARGIVFMCVEYC | 3.600 | 2.260 | 2.170 | 1.714 | 1.050 | 2.009 | 3.776 | 2.172 | 1.837 | 2.135 | 2.657 | 2.240 |
| nsp6 | aa187-201 | RGIVFMCVEYCPIFF | 1.810 | 1.260 | 0.913 | 1.066 | 0.611 | 2.433 | 3.506 | 2.000 | 1.218 | 1.621 | 2.210 | 1.820 |
| nsp6 | aa211-225 | MLVYCFLGYFCTCYF | 3.210 | 1.570 | 1.550 | 1.137 | 1.060 | 2.778 | 3.417 | 2.026 | 2.413 | 1.760 | 2.909 | 2.400 |
| nsp6 | aa215-229 | CFLGYFCTCYFGLFC | 2.880 | 1.320 | 1.360 | 0.866 | 1.010 | 2.638 | 3.101 | 1.719 | 2.074 | 1.654 | 2.649 | 2.230 |
| nsp6 | aa229-243 | CLLNRYFRLTLGVYD | 3.240 | 1.340 | 1.210 | 0.086 | 0.709 | 1.599 | 3.041 | 1.574 | 1.819 | 1.326 | 1.797 | 1.570 |
| nsp6 | aa231-245 | LNRYFRLTLGVYDYL | 3.280 | 1.860 | 1.550 | 1.005 | 1.290 | 2.394 | 3.156 | 1.861 | 2.424 | 1.549 | 2.748 | 2.370 |
| nsp6 | aa239-253 | LGVYDYLVSTQEFRY | 2.520 | 0.939 | 1.590 | 0.672 | 1.050 | 1.737 | 3.200 | 1.981 | 1.846 | 1.266 | 2.457 | 2.360 |
| nsp8 | aa141-155 | CDGTTFTYASALWEI | 3.120 | 1.500 | 2.020 | 1.626 | 1.370 | 2.491 | 3.081 | 2.123 | 2.173 | 1.756 | 2.782 | 2.440 |
| nsp9 | aa17-31 | TTQTACTDDNALAYY | 2.870 | 1.690 | 1.620 | 0.880 | 1.010 | 2.218 | 3.328 | 2.022 | 2.142 | 1.646 | 1.615 | 2.410 |
| nsp9 | aa101-115 | VLGSLAATVRLQAGN | 0.336 | -1.140 | -0.820 | -1.255 | 1.960 | -0.582 | -0.188 | 3.808 | -1.377 | -1.068 | -1.103 | -1.020 |
| nsp10 | aa63-77 | MDQESFGGASCCLYC | 3.030 | 1.900 | 1.850 | 0.665 | 1.110 | 2.396 | 2.926 | 1.779 | 2.548 | 2.028 | 2.678 | 2.490 |
| nsp12 | aa25-39 | TGTSTDVVYRAFDIY | 3.050 | 1.500 | 2.560 | 1.131 | 1.160 | 2.393 | 3.078 | 1.826 | 1.881 | 1.495 | 2.686 | 2.750 |
| nsp12 | aa27-41 | TSTDVVYRAFDIYND | 3.410 | 1.650 | 1.480 | 1.014 | 0.284 | 1.283 | 3.243 | 1.570 | 1.433 | 1.387 | 1.581 | 2.220 |
| nsp12 | aa57-71 | FQEKDEDDNLIDSYF | 3.730 | 1.880 | 2.330 | 1.247 | 1.000 | 1.779 | 3.857 | 2.206 | 1.887 | 1.529 | 1.616 | 2.240 |
| nsp12 | aa59-73 | EKDEDDNLIDSYFVV | 3.390 | 1.600 | 1.710 | 1.248 | 1.150 | 2.078 | 3.759 | 1.487 | 1.005 | 1.231 | 1.322 | 1.870 |
| nsp12 | aa125-139 | MADLVYALRHFDEGN | 0.440 | -0.437 | 0.497 | -0.232 | -1.140 | -0.041 | 0.886 | 3.298 | -0.878 | -0.169 | -0.600 | 1.250 |
| nsp12 | aa141-155 | DTLKEILVTYNCCDD | 3.350 | 1.340 | 2.250 | 1.937 | 0.906 | 0.812 | 3.105 | 1.597 | -0.929 | 1.716 | 1.068 | 2.310 |
| nsp12 | aa143-157 | LKEILVTYNCCDDDY | 4.350 | 2.320 | 2.930 | 2.713 | 1.830 | 2.039 | 4.063 | 2.608 | 0.771 | 2.066 | 2.267 | 3.250 |
| nsp12 | aa145-159 | EILVTYNCCDDDYFN | 4.220 | 2.150 | 2.570 | 2.701 | 0.966 | 2.022 | 4.032 | 2.134 | 0.759 | 1.929 | 2.026 | 2.840 |
| nsp12 | aa149-163 | TYNCCDDDYFNKKDW | 3.320 | 1.210 | 1.340 | 1.390 | 0.129 | 2.388 | 3.387 | 2.022 | 0.613 | 0.848 | 1.626 | 1.950 |
| nsp12 | aa151-165 | NCCDDDYFNKKDWYD | 4.530 | 2.290 | 2.500 | 2.408 | 1.500 | 2.813 | 4.299 | 2.696 | 1.675 | 1.975 | 2.570 | 3.170 |
| nsp12 | aa153-167 | CDDDYFNKKDWYDFV | 3.530 | 1.770 | 2.110 | 1.877 | 1.340 | 2.826 | 3.752 | 2.326 | 1.571 | 1.718 | 2.661 | 3.260 |
| nsp12 | aa155-169 | DDYFNKKDWYDFVEN | 3.020 | 1.210 | 1.260 | 0.859 | -0.144 | 2.044 | 3.076 | 1.111 | 1.219 | 0.366 | 1.245 | 2.350 |
| nsp12 | aa205-219 | VLTLDNQDLNGNWYD | 4.020 | 2.050 | 2.250 | 1.007 | 1.570 | 2.180 | 3.942 | 2.056 | 2.273 | 1.702 | 2.479 | 2.490 |
| nsp12 | aa207-221 | TLDNQDLNGNWYDFG | 2.610 | 1.350 | 1.330 | 0.635 | 1.130 | 2.141 | 3.017 | 1.260 | 1.759 | 1.428 | 2.300 | 2.010 |
| nsp12 | aa209-223 | DNQDLNGNWYDFGDF | 3.980 | 2.070 | 2.550 | 1.698 | 1.480 | 2.280 | 4.170 | 2.136 | 2.368 | 1.693 | 2.772 | 2.620 |
| nsp12 | aa225-239 | QTTPGSGVPVVDSYY | 1.700 | 1.390 | 1.550 | 0.993 | 0.796 | 1.887 | 3.183 | 1.591 | 2.514 | 1.645 | 1.941 | 1.900 |
| nsp12 | aa227-241 | TPGSGVPVVDSYYSL | 0.980 | 1.320 | 1.330 | 0.860 | 0.783 | 2.606 | 3.061 | 0.820 | 2.137 | 1.091 | 1.632 | 1.720 |
| nsp12 | aa233-247 | PVVDSYYSLLMPILT | 1.150 | 2.100 | 0.237 | 1.329 | 0.260 | 1.736 | 3.293 | 0.019 | 1.158 | 1.008 | 0.442 | 0.559 |
| nsp12 | aa339-353 | VPFVVSTGYHFRELG | 2.160 | -0.474 | 0.543 | -0.337 | 0.232 | -0.077 | 3.145 | -0.291 | 1.030 | 0.419 | 0.774 | 0.702 |
| nsp12 | aa409-423 | QTVKPGNFNKDFYDF | 3.080 | 1.320 | 1.620 | 0.969 | 0.708 | 1.262 | 3.230 | 1.976 | 1.024 | 0.830 | 1.913 | 2.260 |
| nsp12 | aa441-455 | FFFAQDGNAAISDYD | 3.340 | 1.230 | 1.700 | 0.684 | 0.903 | 1.384 | 3.642 | 1.944 | 1.870 | 1.219 | 1.742 | 2.870 |
| nsp12 | aa443-457 | FAQDGNAAISDYDYY | 4.610 | 2.950 | 3.320 | 2.660 | 2.140 | 2.940 | 4.486 | 3.123 | 3.085 | 2.600 | 3.380 | 3.220 |
| nsp12 | aa445-459 | QDGNAAISDYDYYRY | 4.000 | 2.400 | 2.390 | 1.717 | 1.340 | 2.528 | 4.060 | 2.699 | 2.810 | 2.115 | 3.265 | 2.450 |
| nsp12 | aa461-475 | LPTMCDIRQLLFVVE | 3.110 | 0.611 | 0.846 | -1.000 | 0.849 | 0.635 | 2.498 | -0.091 | 0.663 | 0.428 | 1.450 | 1.270 |
| nsp12 | aa463-477 | TMCDIRQLLFVVEVV | 3.720 | 1.930 | 2.230 | 0.713 | 1.140 | 1.262 | 3.451 | 0.761 | 1.183 | 1.106 | 1.963 | 1.480 |
| nsp12 | aa467-481 | IRQLLFVVEVVDKYF | 2.660 | 1.090 | 0.931 | 0.651 | 0.298 | 0.644 | 3.149 | 1.322 | 1.420 | 0.460 | 2.369 | 0.013 |
| nsp12 | aa469-483 | QLLFVVEVVDKYFDC | 4.030 | 1.670 | 2.860 | 1.944 | 1.500 | 1.929 | 3.853 | 2.090 | 1.923 | 2.338 | 2.427 | 2.080 |
| nsp12 | aa471-485 | LFVVEVVDKYFDCYD | 5.010 | 2.910 | 3.500 | 2.530 | 2.100 | 2.075 | 4.414 | 3.178 | 2.563 | 2.456 | 3.185 | 2.650 |
| nsp12 | aa475-489 | EVVDKYFDCYDGGCI | 3.710 | 1.330 | 2.710 | 1.259 | 1.070 | 1.468 | 3.312 | 2.153 | 2.345 | 1.936 | 2.372 | 1.890 |
| nsp12 | aa515-529 | LYYDSMSYEDQDALF | 2.930 | 1.420 | 1.590 | 1.639 | 0.783 | 1.949 | 3.516 | 1.989 | 0.421 | 1.026 | 2.035 | 2.500 |
| nsp12 | aa517-531 | YDSMSYEDQDALFAY | 3.060 | 1.470 | 1.830 | 1.808 | 1.290 | 2.211 | 3.623 | 1.869 | 1.382 | 1.236 | 2.362 | 3.110 |
| nsp12 | aa719-733 | KYVRNLQHRLYECLY | 2.770 | 1.700 | 1.490 | 0.613 | 1.110 | 1.645 | 3.196 | 1.810 | 1.679 | 1.456 | 2.556 | 2.310 |
| nsp12 | aa733-747 | YRNRDVDTDFVNEFY | 3.630 | 1.460 | 2.360 | 1.493 | 1.390 | 1.672 | 3.944 | 1.994 | 2.214 | 1.527 | 2.449 | 2.780 |
| nsp12 | aa735-749 | NRDVDTDFVNEFYAY | 3.750 | 1.420 | 2.170 | 1.769 | 1.800 | 1.896 | 3.893 | 1.913 | 2.174 | 1.522 | 2.701 | 2.880 |
| nsp12 | aa753-767 | HFSMMILSDDAVVCF | 1.820 | 0.769 | 0.853 | 1.404 | -0.376 | 0.585 | 3.075 | 1.959 | 1.702 | -0.179 | 2.168 | 1.140 |
| nsp12 | aa819-833 | MLVKQGDDYVYLPYP | 2.400 | 1.340 | 1.320 | -0.037 | 0.756 | 2.041 | 3.063 | 1.523 | 1.094 | 1.521 | 1.803 | 1.890 |
| nsp12 | aa831-845 | PYPDPSRILGAGCFV | 1.390 | 0.809 | 0.454 | -0.667 | 0.246 | 2.133 | 3.127 | 0.496 | 1.208 | 1.106 | 1.306 | 1.360 |
| nsp12 | aa869-883 | PLTKHPNQEYADVFH | 0.684 | -0.265 | -0.475 | 0.131 | -0.324 | -0.987 | 3.077 | 0.998 | 0.667 | -0.154 | 0.845 | 0.561 |
| nsp12 | aa877-891 | EYADVFHLYLQYIRK | -0.218 | -1.570 | -1.400 | -0.475 | -0.237 | 0.615 | -0.505 | -0.488 | -0.374 | -0.343 | -0.546 | 3.050 |
| nsp12 | aa907-921 | MLTNDNTSRYWEPEF | 2.270 | 1.210 | 1.620 | 1.227 | 1.010 | 2.094 | 3.207 | 1.250 | 1.246 | 1.320 | 1.721 | 1.810 |
| nsp12 | aa909-923 | TNDNTSRYWEPEFYE | 3.650 | 2.340 | 2.750 | 2.413 | 1.610 | 2.912 | 4.036 | 1.878 | 2.049 | 1.615 | 2.339 | 2.790 |
| nsp12 | aa911-925 | DNTSRYWEPEFYEAM | 2.100 | 1.310 | 1.680 | 1.192 | 1.090 | 2.172 | 3.498 | 1.182 | 1.449 | 1.565 | 1.808 | 2.120 |
| nsp12 | aa913-927 | TSRYWEPEFYEAMYT | 2.890 | 1.950 | 2.380 | 1.447 | 1.100 | 2.592 | 3.760 | 2.059 | 1.918 | 1.796 | 2.426 | 2.080 |
| nsp13 | aa19-33 | ACIRRPFLCCKCCYD | 2.920 | 0.970 | 1.500 | 0.584 | 0.389 | 1.519 | 3.154 | 1.625 | 0.753 | 1.249 | 1.612 | 1.360 |
| nsp13 | aa59-73 | TDVTQLYLGGMSYYC | 2.830 | 1.670 | 2.100 | 1.149 | 1.290 | 2.099 | 3.322 | 1.711 | 2.748 | 1.911 | 2.642 | 2.590 |
| nsp13 | aa107-121 | FNAIATCDWTNAGDY | 2.340 | 1.250 | 1.420 | 1.239 | 0.845 | 1.589 | 3.289 | 1.590 | 1.016 | 1.061 | 1.663 | 2.190 |
| nsp13 | aa109-123 | AIATCDWTNAGDYIL | 2.130 | 1.480 | 1.320 | 1.073 | 0.610 | 1.559 | 3.365 | 1.479 | 1.103 | 1.198 | 1.875 | 1.950 |
| nsp13 | aa195-209 | QIGEYTFEKGDYGDA | 2.460 | 1.180 | 1.110 | 0.721 | 0.516 | 1.005 | 3.115 | 0.410 | 2.029 | 0.760 | 1.082 | 1.790 |
| nsp13 | aa363-377 | ALPETTADIVVFDEI | 2.450 | 1.580 | 1.310 | 1.518 | 1.030 | 0.688 | 3.051 | 1.226 | 0.073 | 0.172 | -0.068 | 1.970 |
| nsp13 | aa445-459 | CPAEIVDTVSALVYD | 3.420 | 1.610 | 2.100 | 1.060 | 0.953 | 1.035 | 3.551 | 2.042 | 1.375 | 1.588 | 2.053 | 1.730 |
| nsp13 | aa531-545 | TQTVDSSQGSEYDYV | 2.620 | 1.800 | 1.800 | 1.564 | 1.060 | 2.000 | 3.357 | 1.637 | 1.747 | 1.208 | 1.869 | 2.520 |
| nsp13 | aa533-547 | TVDSSQGSEYDYVIF | 3.910 | 2.500 | 2.570 | 2.271 | 1.590 | 2.345 | 4.005 | 2.433 | 1.889 | 1.550 | 2.467 | 2.000 |
| nsp14 | aa121-135 | PTGYVDTPNNTDFSR | 1.090 | 0.326 | -0.296 | -0.174 | -0.411 | 1.160 | 3.341 | -0.174 | 0.758 | 0.697 | 0.631 | 1.010 |
| nsp14 | aa143-157 | GDQFKHLIPLMYKGL | 0.166 | -0.347 | -0.514 | -1.248 | -1.550 | -0.962 | 0.312 | -0.260 | -0.399 | -0.196 | 3.039 | -0.305 |
| nsp14 | aa221-235 | SDTYACWHHSIGFDY | 2.220 | 1.470 | 1.890 | 0.380 | 1.250 | 1.975 | 3.255 | 1.982 | 1.931 | 1.058 | 1.971 | 2.550 |
| nsp14 | aa223-237 | TYACWHHSIGFDYVY | 3.010 | 1.920 | 2.020 | 1.052 | 1.070 | 2.448 | 3.617 | 2.385 | 2.331 | 1.726 | 2.768 | 2.400 |
| nsp14 | aa247-261 | WGFTGNLQSNHDLYC | 3.090 | 1.350 | 1.540 | 0.485 | 0.409 | 1.653 | 3.130 | 1.651 | 2.054 | 1.420 | 1.254 | 2.020 |
| nsp14 | aa285-299 | CFVKRVDWTIEYPII | 2.420 | 2.420 | 0.822 | 1.420 | 0.745 | 1.412 | 3.225 | 1.512 | 1.409 | 1.180 | 1.543 | 1.980 |
| nsp14 | aa295-309 | EYPIIGDELKINAAC | 1.830 | 0.407 | 0.575 | 0.327 | 3.310 | 0.703 | 1.817 | -0.289 | 0.268 | 0.736 | 0.228 | 0.925 |
| nsp14 | aa355-369 | PCSDKAYKIEELFYS | 2.180 | 1.280 | 1.180 | 0.269 | 0.919 | 1.825 | 3.039 | 1.558 | 1.280 | 0.986 | 1.910 | 2.100 |
| nsp14 | aa365-379 | ELFYSYATHSDKFTD | 0.906 | -0.280 | 3.470 | -1.250 | -0.235 | -0.209 | 0.996 | -0.454 | 0.453 | -0.302 | -0.214 | 0.587 |
| nsp14 | aa371-385 | ATHSDKFTDGVCLFW | 2.290 | 1.320 | 1.190 | 0.523 | 0.625 | 2.206 | 3.107 | 2.428 | 1.830 | 1.049 | 2.473 | 2.010 |
| nsp14 | aa497-511 | AYNMMISAGFSLWVY | 2.790 | 1.530 | 1.210 | 0.827 | 1.370 | 2.665 | 3.211 | 1.764 | 2.383 | 1.696 | 2.974 | 2.780 |
| nsp15 | aa75-89 | NLGVDIAANTVIWDY | 3.530 | 1.870 | 2.090 | 1.408 | 2.040 | 2.550 | 3.537 | 1.888 | 1.890 | 1.646 | 2.609 | 2.840 |
| nsp15 | aa121-135 | TVFFDGRVDGQVDLF | 2.670 | 1.070 | 1.290 | 0.359 | 0.348 | 1.330 | 3.131 | 1.439 | 1.486 | 0.234 | 1.787 | 2.590 |
| nsp15 | aa209-223 | QMEIDFLELAMDEFI | 2.990 | 1.510 | 1.570 | 0.732 | 0.868 | 1.531 | 3.432 | 1.380 | 0.366 | 1.081 | 1.290 | 2.370 |
| nsp15 | aa211-225 | EIDFLELAMDEFIER | 3.400 | 0.970 | 1.500 | 0.202 | 0.787 | 1.800 | 3.103 | 1.419 | -0.293 | 0.778 | 0.775 | 2.310 |
| nsp15 | aa219-233 | MDEFIERYKLEGYAF | 2.440 | 1.210 | 1.410 | 0.437 | 1.050 | 1.667 | 3.054 | 1.726 | 1.190 | 1.061 | 1.612 | 2.470 |
| nsp15 | aa227-241 | KLEGYAFEHIVYGDF | 2.840 | 1.510 | 1.980 | 0.428 | 1.090 | 1.730 | 3.069 | 2.164 | 1.749 | 0.986 | 1.880 | 1.930 |
| nsp15 | aa255-269 | LAKRFKESPFELEDF | 3.060 | 0.851 | 1.450 | 1.140 | 0.868 | 0.931 | 3.010 | 0.710 | 0.402 | 0.671 | 1.212 | 1.670 |
| nsp15 | aa289-303 | SKCVCSVIDLLLDDF | 2.900 | 0.884 | 1.680 | 1.965 | 1.110 | 1.495 | 3.680 | 1.923 | 0.999 | 0.632 | 1.442 | 2.480 |
| nsp15 | aa291-305 | CVCSVIDLLLDDFVE | 3.300 | 1.130 | 2.090 | 2.380 | 1.160 | 1.907 | 3.845 | 2.183 | 0.792 | 0.901 | 1.076 | 2.330 |
| nsp15 | aa293-307 | CSVIDLLLDDFVEII | 4.300 | 2.450 | 2.870 | 2.808 | 1.990 | 2.152 | 4.261 | 2.736 | 1.226 | 1.706 | 2.123 | 2.680 |
| nsp15 | aa319-333 | VKVTIDYTEISFMLW | 2.790 | 1.510 | 1.400 | 1.535 | 1.220 | 2.521 | 3.034 | 1.942 | 1.949 | 1.606 | 2.540 | 2.220 |
| nsp16 | aa91-105 | PTGTLLVDSDLNDFV | 1.680 | 0.334 | -0.003 | 0.897 | -0.036 | 0.192 | 3.487 | 0.849 | -1.195 | 0.239 | 0.537 | 2.270 |
| nsp16 | aa115-129 | DCATVHTANKWDLII | 2.300 | 0.545 | 0.666 | 0.005 | 0.029 | 0.942 | 3.510 | 1.274 | 0.727 | 0.300 | 1.875 | 1.460 |
| nsp16 | aa117-131 | ATVHTANKWDLIISD | 2.280 | 0.181 | 0.424 | 0.765 | -0.321 | 0.337 | 3.340 | 0.863 | 1.169 | -0.160 | 1.505 | 0.643 |
| nsp16 | aa119-133 | VHTANKWDLIISDMY | 2.660 | 1.120 | 1.220 | 1.048 | 0.111 | 0.956 | 3.685 | 1.336 | 1.423 | 0.727 | 1.957 | 1.790 |
| nsp16 | aa121-135 | TANKWDLIISDMYDP | 2.830 | 1.080 | 1.630 | 1.082 | 0.276 | 1.142 | 3.166 | 1.016 | 1.296 | 1.269 | 1.766 | 1.650 |
| nsp16 | aa123-137 | NKWDLIISDMYDPKT | 1.810 | -0.545 | 0.697 | 1.372 | -0.726 | -0.166 | 4.252 | -0.429 | 0.466 | -0.086 | 0.760 | 0.502 |
| nsp16 | aa143-157 | ENDSKEGFFTYICGF | 2.330 | 1.160 | 1.500 | 1.105 | 0.872 | 1.504 | 3.114 | 1.773 | 0.810 | 1.357 | 2.213 | 1.940 |
| nsp16 | aa177-191 | WNADLYKLMGHFAWW | 2.520 | 1.210 | 1.140 | 1.047 | 0.768 | 2.462 | 3.263 | 1.981 | 2.211 | 1.355 | 2.410 | 2.560 |
| nsp16 | aa237-251 | PIQLSSYSLFDMSKF | 0.615 | 0.840 | -0.473 | -0.027 | 0.301 | 0.935 | 3.058 | 0.928 | -0.307 | 0.128 | 0.031 | 0.213 |
| Spike Protein | aa57-71 | TQDLFLPFFSNVTWF | 1.910 | 1.110 | 0.922 | 0.935 | 0.649 | 2.063 | 3.009 | 1.653 | 1.644 | 0.929 | 2.345 | 1.660 |
| Spike Protein | aa137-151 | CEFQFCNDPFLGVYY | 4.140 | 2.210 | 2.810 | 2.233 | 1.920 | 2.558 | 4.088 | 2.559 | 2.962 | 2.320 | 3.231 | 3.130 |
| Spike Protein | aa163-177 | FRVYSSANNCTFEYV | 2.180 | 1.350 | 1.170 | 1.509 | 0.923 | 1.382 | 3.338 | 1.354 | 1.660 | 1.335 | 2.058 | 1.880 |
| Spike Protein | aa193-207 | KNLREFVFKNIDGYF | 2.410 | 1.360 | 0.882 | 1.091 | 0.181 | 1.674 | 3.167 | 1.821 | 1.664 | 1.069 | 2.281 | 1.840 |
| Spike Protein | aa259-273 | DSSSGWTAGAAAYYV | 2.780 | 1.480 | 1.360 | 0.815 | 1.440 | 2.123 | 3.022 | 1.904 | 2.488 | 1.887 | 2.927 | 2.740 |
| Spike Protein | aa261-275 | SSGWTAGAAAYYVGY | 3.260 | 1.980 | 1.730 | 1.203 | 1.650 | 2.584 | 3.287 | 2.007 | 2.873 | 2.126 | 3.033 | 2.990 |
| Spike Protein | aa349-363 | NATRFASVYAWNRKR | 0.955 | 0.248 | 0.159 | 0.582 | 1.620 | -0.677 | 0.670 | -0.663 | -0.091 | -0.097 | -0.377 | 3.420 |
| Spike Protein | aa579-593 | TDAVRDPQTLEILDI | 2.930 | 1.420 | 1.320 | 0.989 | 0.361 | 0.157 | 3.262 | 0.940 | 0.619 | -0.593 | 0.723 | 1.560 |
| Spike Protein | aa655-669 | CLIGAEHVNNSYECD | 3.200 | 1.200 | 1.940 | 0.362 | 0.650 | 1.546 | 3.213 | 2.217 | 0.936 | 1.659 | 1.401 | 2.310 |
| Spike Protein | aa677-691 | CASYQTQTNSPRRAR | 0.530 | 0.106 | -0.639 | -0.420 | 0.995 | -1.095 | 0.679 | -0.323 | -0.408 | -0.323 | -0.041 | 3.910 |
| Spike Protein | aa869-883 | PLLTDEMIAQYTSAL | 1.730 | 2.140 | 0.069 | 0.896 | 0.479 | 1.187 | 3.172 | 0.420 | -0.869 | 1.325 | 1.831 | 1.540 |
| Spike Protein | aa1145-1159 | DPLQPELDSFKEELD | 3.280 | 0.909 | 1.250 | 0.571 | 0.758 | 0.959 | 2.409 | 1.960 | 0.248 | 0.199 | 0.737 | 1.810 |
| Spike Protein | aa1199-1213 | LNESLIDLQELGKYE | 3.140 | 1.450 | 1.350 | 1.255 | 0.701 | 1.211 | 2.328 | 1.313 | 1.124 | 0.526 | 1.676 | 1.950 |
| Spike Protein | aa1201-1215 | ESLIDLQELGKYEQY | 3.130 | 1.390 | 1.570 | 1.520 | 1.100 | 1.857 | 2.963 | 1.543 | 1.774 | 0.871 | 2.177 | 2.360 |
| Spike Protein | aa1207-1221 | QELGKYEQYIKWPWY | 3.300 | 1.340 | 1.720 | 1.788 | 1.290 | 2.280 | 3.427 | 2.035 | 2.420 | 1.325 | 2.820 | 2.450 |
| Spike Protein | aa1209-1223 | LGKYEQYIKWPWYIW | 2.970 | 0.804 | 0.922 | 1.384 | 1.260 | 2.676 | 3.020 | 1.676 | 2.045 | 1.074 | 2.629 | 2.510 |
| Spike Protein | aa1253-1267 | CCSCGSCCKFDEDDS | 2.440 | 1.070 | 1.430 | 1.313 | 0.099 | 1.063 | 3.140 | 1.367 | 0.338 | 0.875 | 1.268 | 2.970 |
| Orf3a Protein | aa99-113 | HLLLVAAGLEAPFLY | 2.410 | 1.620 | 1.510 | 1.221 | 1.060 | 1.572 | 3.138 | 2.005 | 1.335 | 0.955 | 2.811 | 2.170 |
| Orf3a Protein | aa101-115 | LLVAAGLEAPFLYLY | 3.010 | 2.050 | 1.960 | 1.717 | 1.530 | 2.349 | 3.590 | 1.964 | 2.033 | 1.609 | 2.793 | 2.780 |
| Orf3a Protein | aa105-119 | AGLEAPFLYLYALVY | 2.970 | 1.790 | 1.670 | 1.222 | 1.290 | 2.636 | 3.269 | 1.784 | 1.811 | 1.593 | 2.661 | 2.720 |
| Orf3a Protein | aa141-155 | SKNPLLYDANYFLCW | 2.930 | 1.600 | 1.670 | 0.838 | 1.260 | 2.507 | 3.422 | 2.009 | 2.675 | 1.589 | 3.026 | 2.540 |
| Orf3a Protein | aa147-161 | YDANYFLCWHTNCYD | 3.750 | 1.950 | 2.140 | 0.905 | 1.290 | 1.729 | 4.012 | 2.031 | 1.850 | 1.728 | 2.130 | 2.840 |
| Orf3a Protein | aa149-163 | ANYFLCWHTNCYDYC | 3.780 | 2.350 | 2.800 | 1.626 | 2.080 | 2.528 | 4.159 | 2.386 | 2.599 | 2.328 | 2.717 | 3.340 |
| Orf3a Protein | aa151-165 | YFLCWHTNCYDYCIP | 3.250 | 1.880 | 2.150 | 1.084 | 1.160 | 2.162 | 3.621 | 2.077 | 2.208 | 2.076 | 2.584 | 2.740 |
| Orf3a Protein | aa205-219 | DCVVLHSYFTSDYYQ | 2.490 | 1.620 | 1.020 | 1.624 | 0.965 | 1.067 | 3.561 | 1.722 | 2.246 | 1.332 | 2.038 | 2.310 |
| Orf3a Protein | aa207-221 | VVLHSYFTSDYYQLY | 2.950 | 2.080 | 1.610 | 1.780 | 1.390 | 1.890 | 4.011 | 2.043 | 2.560 | 1.911 | 2.884 | 2.730 |
| Membrane Glycoprotein | aa23-37 | LEQWNLVIGFLFLTW | 2.470 | 0.849 | 0.889 | 0.463 | 0.705 | 2.370 | 3.095 | 1.376 | 1.276 | 1.312 | 2.438 | 2.260 |
| Orf6 Protein | aa11-25 | LVDFQVTIAEILLII | 3.010 | 1.170 | 1.290 | 0.965 | 0.861 | 1.705 | 3.575 | 1.444 | 1.309 | 1.246 | 2.100 | 1.350 |
| Orf6 Protein | aa25-39 | IMRTFKVSIWNLDYI | 2.380 | 1.490 | 1.220 | 1.408 | 0.931 | 1.533 | 3.127 | 2.002 | 1.734 | 0.978 | 2.186 | 2.140 |
| Orf7a Protein | aa11-25 | ILFLALITLATCELY | 3.320 | 1.930 | 1.950 | 1.230 | 1.560 | 2.572 | 3.580 | 1.950 | 1.504 | 1.717 | 2.652 | 2.750 |
| Orf7a Protein | aa13-27 | FLALITLATCELYHY | 2.610 | 1.500 | 1.300 | 1.126 | 1.100 | 1.849 | 3.149 | 1.796 | 1.162 | 1.653 | 2.902 | 2.590 |
| Orf7a Protein | aa97-111 | QEEVQELYSPIFLIV | 2.770 | 0.947 | 1.070 | 1.775 | 1.180 | 1.802 | 3.610 | 1.045 | 1.199 | 0.236 | 1.784 | 1.770 |
| Orf8 Protein | aa77-91 | PIQYIDIGNYTVSCL | 1.930 | 2.010 | 0.859 | 1.395 | 1.020 | 1.990 | 3.575 | 1.509 | 1.748 | 1.719 | 1.983 | 1.940 |
| Orf8 Protein | aa79-93 | QYIDIGNYTVSCLPF | 1.150 | 0.771 | 0.509 | 0.903 | 0.756 | 1.657 | 3.026 | 1.189 | 1.041 | 1.008 | 1.378 | 1.960 |
| Orf8 Protein | aa101-115 | KLGSLVVRCSFYEDF | 2.740 | 1.620 | 1.920 | 1.788 | 1.740 | 1.829 | 3.668 | 1.998 | 1.285 | 0.651 | 2.101 | 2.400 |
| Orf8 Protein | aa103-117 | GSLVVRCSFYEDFLE | 3.620 | 2.380 | 2.480 | 2.204 | 1.880 | 2.266 | 3.633 | 1.925 | 1.831 | 1.517 | 2.099 | 2.560 |
| Orf8 Protein | aa105-119 | LVVRCSFYEDFLEYH | 3.710 | 2.280 | 2.230 | 2.426 | 1.090 | 1.848 | 4.091 | 2.617 | 2.068 | 1.161 | 2.490 | 2.370 |
| Orf8 Protein | aa107-121 | VRCSFYEDFLEYHDV | 3.940 | 1.920 | 2.340 | 1.987 | 1.010 | 1.588 | 3.640 | 2.334 | 1.757 | 0.820 | 2.035 | 2.280 |
| Orf8 Protein | aa111-125 | FYEDFLEYHDVRVVL | 2.090 | 1.490 | 1.400 | 1.422 | 0.819 | 1.557 | 3.000 | 1.819 | 0.201 | -0.163 | 1.536 | 2.020 |
| Orf8 Protein | aa113-127 | EDFLEYHDVRVVLDF | 2.790 | 1.460 | 1.720 | 1.897 | 1.370 | 2.022 | 3.111 | 1.434 | 0.415 | 0.463 | 1.274 | 2.290 |
| Nucleocapsid Phosphoprotein | aa59-73 | WFTALTQHGKEDLKF | 1.570 | -0.632 | 3.400 | -0.596 | -1.120 | -0.548 | 0.828 | -0.081 | -0.396 | -0.889 | 0.205 | 0.540 |
| Nucleocapsid Phosphoprotein | aa105-119 | DGKMKDLSPRWYFYY | 3.120 | 1.620 | 1.590 | 1.071 | 1.680 | 2.460 | 3.032 | 2.133 | 2.298 | 1.316 | 2.910 | 2.890 |
| Nucleocapsid Phosphoprotein | aa209-223 | SRGTSPARMAGNGGD | 1.160 | -0.095 | 0.037 | 0.066 | -0.368 | 0.916 | 1.432 | 3.133 | 0.264 | 0.584 | -0.594 | 0.449 |
| Nucleocapsid Phosphoprotein | aa339-353 | TYTGAIKLDDKDPNF | 3.070 | 0.288 | 1.050 | 0.703 | 0.009 | 0.375 | 2.582 | 0.971 | 1.105 | 0.641 | 1.368 | 1.400 |
| Nucleocapsid Phosphoprotein | aa369-383 | TFPPTEPKKDKKKKA | 1.420 | -0.363 | -0.386 | 0.579 | -0.977 | -0.544 | 3.133 | -1.190 | -1.166 | -0.289 | -0.642 | 0.017 |

v
