# Supplementary material for: Humoral Immune Response Profile of COVID-19 Reveals Severity and Variant-Specific Epitopes: Lessons from SARS-CoV-2 Peptide Microarray
Source: Viruses. 2023 Jan 15;15(1):248. doi: 10.3390/v15010248 (PMC9866125; doi:10.3390/v15010248)
Supplement: Supplementary file 1 [file viruses-15-00248-s001.zip › Table S3.docx]

Table S3. IgG reactive peptides from SARS-CoV-2 proteome

| Protein | Sequence | Peptide | NS1 | NS2 | NS3 | NS4 | NS5 | NS6 | SV1 | SV2 | SV3 | SV4 | SV5 | SV6 |
| --- | --- | --- | --- | --- | --- | --- | --- | --- | --- | --- | --- | --- | --- | --- |
| nsp1 | aa55-69 | DGTCGLVEVEKGVLP | -0.624 | -0.746 | -0.196 | -0.408 | -0.706 | 0.288 | -0.162 | 0.515 | -0.162 | -0.569 | 3.508 | 0.086 |
| nsp1 | aa57-71 | TCGLVEVEKGVLPQL | -0.811 | -0.795 | -0.780 | -0.816 | -0.857 | -0.548 | -0.617 | 1.430 | -0.645 | -0.780 | 3.208 | -0.169 |
| nsp1 | aa61-75 | VEVEKGVLPQLEQPY | -0.564 | 0.682 | 0.280 | -0.790 | -0.469 | -0.221 | -0.049 | 3.450 | 0.321 | 0.644 | 2.739 | 0.557 |
| nsp1 | aa63-77 | VEKGVLPQLEQPYVF | -0.611 | -0.445 | -0.464 | -0.814 | -0.737 | -0.433 | -0.220 | 3.100 | 0.034 | -0.622 | 2.671 | 0.313 |
| nsp1 | aa119-133 | GEIPVAYRKVLLRKN | -0.397 | -0.651 | -0.832 | -0.641 | -0.722 | -0.017 | -0.719 | 3.440 | -0.048 | -0.710 | -0.292 | -0.476 |
| nsp1 | aa121-135 | IPVAYRKVLLRKNGN | -0.739 | -0.759 | -0.778 | -0.776 | -0.856 | -0.761 | -0.783 | 6.070 | 0.400 | -0.856 | 0.478 | -0.616 |
| nsp1 | aa137-151 | GAGGHSYGADLKSFD | -0.288 | 1.232 | -0.506 | -0.723 | -0.703 | 0.886 | 1.475 | 3.510 | 1.140 | 0.362 | 2.023 | 1.513 |
| nsp1 | aa141-155 | HSYGADLKSFDLGDE | 0.232 | 0.881 | 0.052 | -0.173 | -0.017 | 0.611 | 0.768 | 4.050 | 1.930 | 1.269 | 2.215 | 2.537 |
| nsp1 | aa143-157 | YGADLKSFDLGDELG | 0.063 | 0.697 | -0.001 | -0.016 | 0.353 | 1.028 | 0.780 | 3.050 | 1.650 | 0.896 | 1.998 | 2.101 |
| nsp1 | aa147-161 | LKSFDLGDELGTDPY | 0.904 | 1.639 | 0.178 | 0.936 | 0.512 | 1.588 | 2.025 | 4.350 | 2.180 | 1.982 | 4.528 | 3.433 |
| nsp1 | aa149-163 | SFDLGDELGTDPYED | 2.040 | 2.482 | 1.190 | 1.653 | 1.523 | 3.033 | 2.570 | 4.540 | 3.030 | 2.728 | 3.895 | 4.569 |
| nsp1 | aa151-165 | DLGDELGTDPYEDFQ | 1.327 | 2.221 | 0.602 | 0.869 | 0.593 | 1.734 | 2.346 | 4.540 | 2.570 | 1.668 | 3.352 | 4.035 |
| nsp1 | aa153-167 | GDELGTDPYEDFQEN | 0.415 | 2.320 | 0.485 | 0.223 | 0.070 | 1.120 | 1.955 | 3.550 | 2.340 | 1.895 | 3.222 | 3.477 |
| nsp1 | aa155-169 | ELGTDPYEDFQENWN | -0.009 | 1.759 | 0.610 | 0.341 | -0.106 | 1.677 | 2.219 | 4.390 | 2.450 | 1.574 | 3.960 | 3.946 |
| nsp2 | aa5-19 | ELMRELNGGAYTRYV | -0.260 | 0.237 | 0.114 | -0.724 | -0.607 | 0.908 | 0.624 | 3.110 | 0.844 | -0.569 | 1.576 | 1.253 |
| nsp2 | aa9-23 | ELNGGAYTRYVDNNF | -0.775 | 0.582 | 0.141 | -0.820 | -0.763 | -0.650 | 1.105 | 3.270 | -0.414 | -0.088 | 2.539 | 0.539 |
| nsp2 | aa13-27 | GAYTRYVDNNFCGPD | -0.504 | -0.655 | -0.142 | -0.795 | -0.793 | -0.315 | 0.633 | 0.474 | 0.275 | 0.976 | 3.817 | 1.043 |
| nsp2 | aa15-29 | YTRYVDNNFCGPDGY | 0.263 | 1.108 | 1.140 | -0.005 | -0.179 | 0.211 | 1.708 | 4.490 | 2.270 | 1.756 | 4.304 | 2.682 |
| nsp2 | aa17-31 | RYVDNNFCGPDGYPL | -0.509 | -0.029 | 0.797 | -0.831 | -0.824 | -0.152 | 0.916 | 3.110 | 1.310 | 0.857 | 2.977 | 1.976 |
| nsp2 | aa19-33 | VDNNFCGPDGYPLEC | 0.876 | 1.513 | 1.480 | 0.228 | 1.155 | 1.637 | 1.530 | 4.040 | 2.550 | 4.601 | 1.936 | 2.483 |
| nsp2 | aa59-73 | RGVYCCREHEHEIAW | 0.543 | 1.478 | 0.900 | 0.270 | -0.316 | 1.257 | 1.715 | 4.120 | 1.970 | 1.275 | 3.765 | 3.076 |
| nsp2 | aa61-75 | VYCCREHEHEIAWYT | -0.013 | 0.882 | 0.703 | -0.063 | -0.362 | 0.500 | 1.138 | 3.690 | 0.902 | 0.433 | 3.250 | 2.686 |
| nsp2 | aa63-77 | CCREHEHEIAWYTER | -0.185 | 1.449 | 1.160 | 0.257 | -0.341 | 1.520 | 1.461 | 4.130 | 2.030 | 1.250 | 3.755 | 3.187 |
| nsp2 | aa69-83 | HEIAWYTERSEKSYE | -0.349 | 0.730 | 0.198 | -0.136 | -0.383 | 1.310 | 0.605 | 3.620 | 0.455 | 0.573 | 2.334 | 1.688 |
| nsp2 | aa89-103 | EIKLAKKFDTFNGEC | -0.439 | -0.020 | 0.178 | -0.225 | 0.240 | 0.828 | 0.154 | 1.970 | 1.520 | 3.179 | 1.337 | 1.106 |
| nsp2 | aa155-169 | KCDHCGETSWQTGDF | -0.565 | 0.539 | -0.218 | -0.586 | -0.404 | -0.148 | 0.665 | 3.510 | 1.970 | 0.364 | 2.574 | 1.722 |
| nsp2 | aa207-221 | HNSEVGPEHSLAEYH | 0.720 | 0.861 | 0.197 | -0.456 | -0.709 | -0.334 | 0.508 | 3.900 | 1.340 | 0.847 | 2.994 | 1.563 |
| nsp2 | aa213-227 | PEHSLAEYHNESGLK | 3.308 | -0.687 | -0.562 | -0.685 | -0.820 | 0.347 | -0.530 | 2.830 | 1.590 | -0.832 | 2.246 | 0.388 |
| nsp2 | aa235-249 | RTIAFGGCVFSYVGC | 0.034 | -0.135 | 1.090 | -0.195 | -0.442 | 0.671 | 0.321 | 1.530 | 1.490 | 4.130 | 0.849 | 0.850 |
| nsp2 | aa295-309 | NIVGDFKLNEEIAII | 0.402 | 0.682 | 0.096 | -0.728 | -0.709 | -0.239 | 0.777 | 3.550 | 2.130 | 0.521 | 1.624 | 0.420 |
| nsp2 | aa315-329 | ASTSAFVETVKGLDY | -0.331 | -0.013 | -0.085 | -0.527 | -0.450 | 0.220 | 0.292 | 3.470 | 2.610 | -0.075 | 1.783 | 1.055 |
| nsp2 | aa327-341 | LDYKAFKQIVESCGN | -0.570 | -0.670 | -0.675 | -0.682 | -0.801 | -0.433 | -0.722 | 4.970 | -0.258 | -0.667 | -0.022 | 1.233 |
| nsp2 | aa449-463 | IFGTVYEKLKPVLDW | -0.367 | 0.098 | -0.071 | 0.004 | 0.098 | 1.906 | 0.745 | 2.890 | 1.630 | 0.030 | 3.451 | 2.373 |
| nsp2 | aa461-475 | LDWLEEKFKEGVEFL | -0.041 | 1.784 | 0.929 | 0.455 | 0.741 | 2.457 | 1.739 | 4.650 | 2.920 | 1.206 | 3.904 | 3.500 |
| nsp2 | aa463-477 | WLEEKFKEGVEFLRD | 1.211 | 1.137 | 0.600 | 0.224 | 0.470 | 2.494 | 1.259 | 4.050 | 2.910 | 1.698 | 3.404 | 3.508 |
| nsp2 | aa465-479 | EEKFKEGVEFLRDGW | 0.177 | 1.301 | 0.996 | 0.662 | 0.749 | 3.009 | 1.946 | 4.700 | 3.290 | 0.949 | 4.713 | 3.608 |
| nsp2 | aa467-481 | KFKEGVEFLRDGWEI | -0.036 | 1.524 | 1.290 | 0.633 | 0.586 | 2.632 | 1.609 | 4.430 | 3.460 | 1.535 | 3.964 | 3.690 |
| nsp2 | aa469-483 | KEGVEFLRDGWEIVK | -0.053 | 0.297 | -0.513 | 0.010 | -0.020 | 1.750 | 0.628 | 3.600 | 3.030 | -0.486 | 3.094 | 2.184 |
| nsp2 | aa471-485 | GVEFLRDGWEIVKFI | 0.013 | 1.442 | 0.757 | -0.199 | -0.124 | 1.108 | 0.765 | 4.170 | 3.280 | -0.369 | 3.058 | 2.654 |
| nsp2 | aa571-585 | EIIFLEGETLPTEVL | -0.648 | 1.512 | 0.246 | -0.721 | -0.522 | 0.245 | 0.406 | 3.450 | 1.350 | 0.311 | 1.696 | -0.249 |
| nsp2 | aa573-587 | IFLEGETLPTEVLTE | -0.321 | -0.469 | 0.375 | -0.799 | -0.203 | -0.205 | -0.385 | 3.870 | 0.208 | 0.694 | -0.221 | 0.359 |
| nsp2 | aa577-591 | GETLPTEVLTEEVVL | -0.183 | 2.363 | -0.059 | -0.734 | -0.713 | 0.307 | -0.019 | 3.500 | 2.050 | -0.103 | 0.316 | -0.028 |
| nsp3 | aa17-31 | KVTFGDDTVIEVQGY | 0.279 | 0.548 | 0.241 | -0.012 | -0.518 | -0.115 | 0.287 | 3.350 | 1.610 | 0.412 | 2.032 | 1.853 |
| nsp3 | aa55-69 | YTVELGTEVNEFACV | 0.182 | -0.355 | 0.545 | 0.212 | -0.197 | -0.256 | 0.593 | 3.200 | 1.950 | 1.967 | 0.172 | 1.146 |
| nsp3 | aa79-93 | QPVSELLTPLGIDLD | 0.697 | 0.867 | -0.181 | 0.020 | -0.082 | 0.675 | 0.660 | 3.230 | -0.267 | 0.889 | 1.803 | 2.742 |
| nsp3 | aa81-95 | VSELLTPLGIDLDEW | 1.521 | 2.935 | 2.070 | 1.345 | 1.285 | 2.952 | 2.164 | 5.640 | 3.090 | 2.430 | 4.345 | 3.849 |
| nsp3 | aa83-97 | ELLTPLGIDLDEWSM | 0.794 | 1.398 | 0.982 | 0.422 | 1.205 | 2.688 | 1.024 | 4.510 | 1.680 | 1.416 | 3.243 | 3.007 |
| nsp3 | aa85-99 | LTPLGIDLDEWSMAT | -0.632 | -0.566 | -0.789 | -0.193 | -0.385 | 1.474 | -0.427 | 0.134 | 0.304 | -0.242 | 2.650 | 3.139 |
| nsp3 | aa87-101 | PLGIDLDEWSMATYY | 1.158 | 3.015 | 1.480 | 0.939 | 0.382 | 2.380 | 2.659 | 5.310 | 3.380 | 2.783 | 4.965 | 3.941 |
| nsp3 | aa89-103 | GIDLDEWSMATYYLF | 0.754 | 3.151 | 1.340 | 0.749 | 0.639 | 2.316 | 2.283 | 5.070 | 3.240 | 2.148 | 4.518 | 3.408 |
| nsp3 | aa91-105 | DLDEWSMATYYLFDE | 1.724 | 2.826 | 2.340 | 1.475 | 1.226 | 2.915 | 2.319 | 5.320 | 3.530 | 2.357 | 4.037 | 3.411 |
| nsp3 | aa93-107 | DEWSMATYYLFDESG | 0.045 | 0.713 | 0.021 | 0.332 | -0.037 | 1.661 | 1.004 | 3.250 | 2.700 | 1.105 | 3.021 | 2.744 |
| nsp3 | aa95-109 | WSMATYYLFDESGEF | 1.579 | 2.324 | 1.750 | 1.138 | 0.517 | 2.159 | 2.112 | 4.920 | 3.990 | 2.389 | 4.084 | 3.690 |
| nsp3 | aa97-111 | MATYYLFDESGEFKL | -0.432 | -0.186 | -0.496 | -0.376 | -0.345 | 1.350 | -0.025 | 3.380 | 1.690 | -0.468 | 2.225 | 2.049 |
| nsp3 | aa103-117 | FDESGEFKLASHMYC | 0.106 | 0.103 | 1.510 | -0.345 | -0.813 | 1.449 | 0.423 | 3.660 | 3.080 | 0.746 | 2.979 | 2.955 |
| nsp3 | aa107-121 | GEFKLASHMYCSFYP | -0.360 | -0.313 | -0.132 | -0.719 | -0.839 | 0.352 | -0.181 | 3.260 | 2.480 | 0.076 | 3.357 | 0.931 |
| nsp3 | aa115-129 | MYCSFYPPDEDEEEG | 1.217 | 1.585 | 1.700 | 1.162 | 1.076 | 2.776 | 0.990 | 2.960 | 2.950 | 2.160 | 1.563 | 3.119 |
| nsp3 | aa117-131 | CSFYPPDEDEEEGDC | 2.101 | 2.437 | 1.860 | 1.887 | 1.306 | 3.188 | 1.957 | 4.670 | 3.790 | 3.783 | 2.331 | 3.869 |
| nsp3 | aa119-133 | FYPPDEDEEEGDCEE | 2.445 | 2.796 | 1.760 | 2.028 | 1.761 | 3.378 | 1.992 | 4.890 | 3.810 | 3.875 | 3.652 | 3.822 |
| nsp3 | aa121-135 | PPDEDEEEGDCEEEE | 2.692 | 3.043 | 1.660 | 2.260 | 1.845 | 3.467 | 1.560 | 4.610 | 3.610 | 3.952 | 4.203 | 3.766 |
| nsp3 | aa123-137 | DEDEEEGDCEEEEFE | 2.544 | 3.087 | 1.730 | 2.295 | 1.986 | 3.549 | 1.638 | 5.190 | 3.880 | 3.888 | 4.467 | 3.764 |
| nsp3 | aa125-139 | DEEEGDCEEEEFEPS | 1.655 | 1.934 | 1.070 | 1.954 | 1.698 | 3.036 | 1.248 | 2.880 | 2.780 | 2.464 | 3.868 | 3.004 |
| nsp3 | aa127-141 | EEGDCEEEEFEPSTQ | 1.345 | 1.670 | 0.422 | 1.376 | 1.080 | 2.759 | 1.552 | 2.540 | 2.120 | 1.823 | 3.581 | 2.108 |
| nsp3 | aa129-143 | GDCEEEEFEPSTQYE | 1.574 | 2.646 | 1.580 | 1.493 | 1.327 | 2.636 | 1.715 | 4.220 | 3.170 | 2.480 | 3.610 | 2.692 |
| nsp3 | aa131-145 | CEEEEFEPSTQYEYG | 1.893 | 2.485 | 1.430 | 1.618 | 1.452 | 2.243 | 1.720 | 4.680 | 3.140 | 2.560 | 4.032 | 3.261 |
| nsp3 | aa133-147 | EEEFEPSTQYEYGTE | 1.163 | 1.843 | 1.210 | 0.804 | 0.925 | 2.552 | 1.523 | 1.960 | 1.910 | 2.182 | 3.989 | 3.312 |
| nsp3 | aa135-149 | EFEPSTQYEYGTEDD | 1.359 | 2.029 | 1.240 | 1.130 | 1.037 | 2.272 | 1.373 | 3.640 | 2.300 | 2.466 | 2.788 | 3.280 |
| nsp3 | aa137-151 | EPSTQYEYGTEDDYQ | 0.707 | 1.768 | 1.080 | 1.101 | 0.305 | 1.396 | 1.859 | 4.090 | 2.040 | 1.897 | 3.101 | 3.296 |
| nsp3 | aa143-157 | EYGTEDDYQGKPLEF | 0.053 | 0.476 | 0.352 | 0.350 | -0.022 | 1.418 | 1.065 | 3.270 | 1.790 | 0.360 | 3.894 | 2.222 |
| nsp3 | aa161-175 | SAALQPEEEQEEDWL | 1.746 | 2.038 | 1.290 | 3.140 | 0.919 | 2.772 | 0.898 | 4.410 | 3.210 | 2.186 | 2.805 | 2.729 |
| nsp3 | aa163-177 | ALQPEEEQEEDWLDD | 2.464 | 2.499 | 1.460 | 3.205 | 1.385 | 3.197 | 1.406 | 4.850 | 3.020 | 3.331 | 3.357 | 2.875 |
| nsp3 | aa165-179 | QPEEEQEEDWLDDDS | 2.253 | 2.154 | 1.410 | 3.391 | 1.326 | 3.059 | 1.206 | 4.160 | 2.940 | 2.510 | 3.361 | 2.749 |
| nsp3 | aa167-181 | EEEQEEDWLDDDSQQ | 0.224 | 0.699 | 0.376 | 0.221 | 0.547 | 2.802 | 0.500 | 3.250 | 1.410 | 0.678 | 3.127 | -0.331 |
| nsp3 | aa221-235 | NSFSGYLKLTDNVYI | -0.722 | -0.468 | -0.624 | -0.687 | -0.828 | -0.541 | 1.800 | 3.070 | 0.905 | -0.727 | 2.152 | 0.145 |
| nsp3 | aa271-285 | NKATNNAMQVESDDY | 0.275 | 0.604 | 0.710 | 0.243 | 0.298 | -0.197 | 0.054 | 3.620 | 0.480 | 0.944 | 2.410 | 3.224 |
| nsp3 | aa275-289 | NNAMQVESDDYIATN | -0.818 | -0.705 | -0.827 | -0.725 | -0.628 | -0.611 | -0.643 | 3.670 | -0.090 | -0.758 | 1.464 | -0.003 |
| nsp3 | aa319-333 | KGEDIQLLKSAYENF | -0.607 | 0.567 | 0.086 | -0.402 | -0.473 | 1.734 | 0.733 | 3.680 | 1.330 | -0.028 | 2.870 | 2.680 |
| nsp3 | aa365-379 | RTNVYLAVFDKNLYD | 0.694 | 1.351 | 1.010 | 0.055 | -0.322 | 0.618 | 2.122 | 4.600 | 3.220 | 0.905 | 3.380 | 1.680 |
| nsp3 | aa421-435 | KQDDKKIKACVEEVT | -0.733 | -0.709 | 0.394 | -0.738 | -0.802 | -0.496 | -0.717 | 0.273 | -0.341 | -0.664 | -0.555 | 3.694 |
| nsp3 | aa437-451 | TLEETKFLTENLLLY | 0.660 | 1.970 | 1.170 | 0.572 | 0.575 | 0.719 | 1.314 | 4.050 | 2.200 | 1.009 | 3.215 | 1.281 |
| nsp3 | aa439-453 | EETKFLTENLLLYID | 1.473 | 2.183 | 1.580 | 1.288 | 0.579 | 0.708 | 1.905 | 4.420 | 3.250 | 1.633 | 3.445 | 0.423 |
| nsp3 | aa441-455 | TKFLTENLLLYIDIN | -0.530 | -0.452 | -0.278 | -0.591 | -0.729 | -0.616 | -0.123 | 3.040 | 0.775 | -0.672 | 1.107 | 1.015 |
| nsp3 | aa443-457 | FLTENLLLYIDINGN | -0.611 | -0.493 | -0.581 | -0.761 | -0.829 | -0.353 | -0.484 | 5.820 | -0.088 | -0.037 | 1.026 | 0.187 |
| nsp3 | aa459-473 | HPDSATLVSDIDITF | -0.503 | 0.700 | -0.077 | -0.250 | -0.762 | -0.295 | 0.026 | 4.110 | 1.470 | 0.429 | 2.659 | 1.591 |
| nsp3 | aa603-617 | GIKIQEGVVDYGARF | -0.474 | 0.635 | 0.211 | 0.085 | -0.076 | 1.755 | 0.762 | 3.330 | 2.440 | 0.081 | 3.887 | 2.316 |
| nsp3 | aa605-619 | KIQEGVVDYGARFYF | -0.642 | 1.186 | 0.089 | 0.067 | -0.256 | 1.090 | 1.341 | 3.860 | 2.130 | 0.458 | 4.352 | 2.229 |
| nsp3 | aa607-621 | QEGVVDYGARFYFYT | -0.236 | 0.575 | 0.315 | -0.417 | -0.674 | 0.115 | -0.034 | 3.160 | 1.330 | 0.376 | 3.188 | 0.342 |
| nsp3 | aa633-647 | LNDLNETLVTMPLGY | -0.065 | 0.423 | 0.686 | -0.350 | -0.490 | 0.973 | 0.051 | 3.430 | 2.420 | 0.533 | 2.908 | 2.013 |
| nsp3 | aa647-661 | YVTHGLNLEEAARYM | -0.336 | -0.037 | 0.787 | -0.129 | -0.106 | 1.678 | 0.983 | 3.140 | 1.520 | 0.632 | 2.134 | 2.177 |
| nsp3 | aa695-709 | HFIETISLAGSYKDW | 0.251 | -0.185 | 0.344 | -0.060 | -0.325 | 1.067 | 0.957 | 4.070 | 2.100 | -0.376 | 3.621 | 2.207 |
| nsp3 | aa697-711 | IETISLAGSYKDWSY | 0.225 | -0.312 | -0.143 | -0.354 | -0.081 | 1.855 | 0.404 | 3.920 | 1.900 | 1.140 | 3.327 | 1.453 |
| nsp3 | aa709-723 | WSYSGQSTQLGIEFL | -0.294 | 1.555 | 0.318 | -0.118 | -0.439 | -0.031 | 0.973 | 3.110 | 2.500 | 0.260 | 1.719 | 1.717 |
| nsp3 | aa717-731 | QLGIEFLKRGDKSVY | -0.484 | 1.034 | -0.049 | -0.465 | -0.790 | 0.450 | 1.035 | 3.680 | 1.830 | 0.211 | 2.868 | 0.777 |
| nsp3 | aa735-749 | NPTTFHLDGEVITFD | 0.100 | 1.751 | 0.872 | -0.090 | -0.282 | 1.226 | 1.272 | 4.000 | 2.850 | 1.865 | 2.250 | 1.327 |
| nsp3 | aa769-783 | VDNINLHTQVVDMSM | -0.510 | -0.545 | -0.572 | -0.157 | -0.756 | 0.539 | -0.760 | 2.910 | 0.359 | -0.654 | 2.082 | 3.097 |
| nsp3 | aa775-789 | HTQVVDMSMTYGQQF | -0.828 | -0.039 | -0.689 | -0.399 | -0.777 | -0.112 | 0.135 | 3.610 | -0.178 | -0.470 | 1.016 | 1.138 |
| nsp3 | aa813-827 | FYVLPNDDTLRVEAF | -0.590 | 0.363 | -0.342 | -0.237 | -0.417 | -0.391 | 0.762 | 2.900 | 1.940 | -0.492 | 3.277 | 1.735 |
| nsp3 | aa815-829 | VLPNDDTLRVEAFEY | 1.227 | 2.796 | 1.970 | 1.098 | 0.978 | 2.027 | 2.048 | 4.870 | 3.180 | 2.514 | 4.359 | 3.324 |
| nsp3 | aa817-831 | PNDDTLRVEAFEYYH | 0.827 | 2.737 | 1.680 | 0.760 | 0.336 | 1.521 | 2.712 | 4.800 | 3.430 | 2.269 | 4.350 | 2.437 |
| nsp3 | aa821-835 | TLRVEAFEYYHTTDP | -0.722 | -0.442 | -0.687 | -0.634 | -0.565 | -0.559 | 0.174 | 0.959 | -0.433 | -0.524 | 7.601 | -0.052 |
| nsp3 | aa823-837 | RVEAFEYYHTTDPSF | -0.409 | 0.196 | 0.121 | -0.157 | -0.706 | -0.187 | 1.072 | 2.250 | 0.280 | 0.037 | 6.219 | 0.274 |
| nsp3 | aa825-839 | EAFEYYHTTDPSFLG | -0.433 | 0.136 | 0.607 | -0.149 | -0.777 | 0.119 | 0.975 | 0.759 | -0.578 | -0.718 | 4.093 | 2.595 |
| nsp3 | aa827-841 | FEYYHTTDPSFLGRY | 0.180 | 1.355 | 1.300 | 0.035 | -0.133 | 1.015 | 2.420 | 4.670 | 2.720 | 1.881 | 5.385 | 3.314 |
| nsp3 | aa831-845 | HTTDPSFLGRYMSAL | -0.623 | -0.664 | -0.632 | -0.791 | -0.832 | 0.280 | -0.698 | 3.040 | 0.974 | -0.662 | 2.375 | 0.344 |
| nsp3 | aa843-857 | SALNHTKKWKYPQVN | -0.864 | -0.728 | -0.867 | -0.875 | -0.834 | -0.431 | -0.857 | 3.520 | -0.717 | -0.837 | 0.067 | -0.201 |
| nsp3 | aa857-871 | NGLTSIKWADNNCYL | -0.361 | 0.494 | -0.268 | -0.533 | -0.620 | -0.036 | 0.480 | 3.370 | 1.980 | -0.172 | 2.682 | 0.394 |
| nsp3 | aa881-895 | IELKFNPPALQDAYY | 0.758 | 2.227 | 1.610 | 0.385 | -0.209 | 0.624 | 2.370 | 5.000 | 3.580 | 2.572 | 4.131 | 2.841 |
| nsp3 | aa899-913 | AGEAANFCALILAYC | 0.021 | 0.387 | 1.170 | 0.243 | -0.073 | 0.830 | 0.777 | 3.310 | 2.080 | 1.539 | 1.906 | 1.457 |
| nsp3 | aa917-931 | VGELGDVRETMSYLF | -0.176 | 1.506 | 0.395 | 0.548 | 0.157 | 1.742 | 1.077 | 3.750 | 1.560 | 0.623 | 2.761 | 2.412 |
| nsp3 | aa955-969 | TTLKGVEAVMYMGTL | -0.816 | -0.711 | -0.776 | -0.789 | -0.565 | 1.380 | -0.810 | -0.360 | 3.420 | -0.839 | -0.305 | 1.756 |
| nsp3 | aa963-977 | VMYMGTLSYEQFKKG | -0.736 | -0.841 | -0.857 | -0.767 | -0.640 | 1.080 | -0.762 | 0.734 | -0.168 | -0.832 | -0.265 | 6.006 |
| nsp3 | aa1011-1025 | LKHGTFTCASEYTGN | -0.850 | -0.611 | -0.237 | -0.828 | -0.859 | -0.377 | -0.716 | 5.660 | 1.310 | 0.251 | 0.030 | -0.003 |
| nsp3 | aa1017-1031 | TCASEYTGNYQCGHY | -0.750 | 0.776 | 0.073 | -0.670 | -0.777 | -0.363 | 0.918 | 2.710 | 1.490 | 0.153 | 3.180 | 2.044 |
| nsp3 | aa1033-1047 | HITSKETLYCIDGAL | -0.700 | -0.519 | -0.474 | -0.582 | -0.805 | -0.127 | -0.435 | 3.410 | 0.986 | -0.028 | 1.235 | 0.676 |
| nsp3 | aa1049-1063 | TKSSEYKGPITDVFY | -0.625 | 0.944 | 0.052 | -0.077 | -0.468 | 0.128 | 0.609 | 4.250 | 0.937 | 0.336 | 2.368 | 0.790 |
| nsp3 | aa1081-1095 | GVVCTEIDPKLDNYY | 1.095 | 2.620 | 1.130 | 0.310 | -0.153 | -0.288 | 1.843 | 4.990 | 2.610 | 1.790 | 4.295 | 3.531 |
| nsp3 | aa1085-1099 | TEIDPKLDNYYKKDN | -0.510 | 0.664 | -0.725 | -0.688 | -0.834 | -0.197 | 0.947 | 2.410 | -0.141 | 0.045 | 3.111 | 0.606 |
| nsp3 | aa1087-1101 | IDPKLDNYYKKDNSY | -0.576 | -0.513 | -0.700 | -0.783 | -0.827 | -0.704 | -0.357 | 3.030 | -0.239 | -0.298 | 2.839 | 1.724 |
| nsp3 | aa1101-1115 | YFTEQPIDLVPNQPY | -0.769 | 0.926 | 0.142 | -0.735 | -0.781 | -0.231 | 0.656 | 2.750 | 0.525 | -0.182 | 3.102 | 1.044 |
| nsp3 | aa1109-1123 | LVPNQPYPNASFDNF | -0.793 | 1.259 | 0.052 | -0.546 | -0.832 | -0.448 | 1.372 | 3.730 | 1.440 | 0.543 | 3.686 | 2.518 |
| nsp3 | aa1111-1125 | PNQPYPNASFDNFKF | 0.323 | -0.428 | -0.475 | -0.590 | -0.792 | -0.238 | 0.317 | 3.060 | 0.817 | -0.677 | 3.056 | 0.736 |
| nsp3 | aa1115-1129 | YPNASFDNFKFVCDN | -0.149 | -0.166 | 0.315 | -0.392 | -0.683 | -0.358 | -0.318 | 3.140 | 1.170 | -0.503 | 1.711 | 2.018 |
| nsp3 | aa1123-1137 | FKFVCDNIKFADDLN | -0.752 | 0.847 | 1.240 | -0.763 | -0.398 | -0.281 | 0.147 | 2.710 | 1.520 | 0.053 | 3.068 | 2.981 |
| nsp3 | aa1145-1159 | PASRELKVTFFPDLN | -0.629 | -0.635 | -0.193 | -0.678 | -0.438 | 0.018 | 0.114 | 3.350 | 1.330 | -0.267 | 0.591 | 2.385 |
| nsp3 | aa1153-1167 | TFFPDLNGDVVAIDY | 0.370 | 1.210 | 1.920 | 0.340 | 0.443 | 1.644 | 1.787 | 4.620 | 2.950 | 1.674 | 3.116 | 2.670 |
| nsp3 | aa1173-1187 | SFKKGAKLLHKPIVW | -0.499 | -0.724 | -0.616 | -0.577 | -0.474 | 0.980 | -0.423 | 3.200 | 1.010 | -0.533 | 1.990 | 0.707 |
| nsp3 | aa1195-1209 | KATYKPNTWCIRCLW | 0.081 | 0.377 | -0.069 | -0.005 | 0.031 | 1.663 | 0.436 | 3.250 | 1.950 | 0.216 | 3.658 | 2.409 |
| nsp3 | aa1257-1271 | LECNVKTTEVVGDII | 0.452 | 0.867 | 0.322 | 0.679 | -0.479 | 0.237 | 1.085 | 3.080 | 2.460 | 1.613 | 0.895 | -0.196 |
| nsp3 | aa1281-1295 | ITEEVGHTDLMAAYV | -0.741 | 0.122 | 0.192 | -0.635 | -0.846 | 0.988 | 0.205 | 2.440 | 1.230 | 0.185 | 2.263 | 3.559 |
| nsp3 | aa1317-1331 | LATHGLAAVNSVPWD | -0.012 | 0.711 | 0.000 | -0.030 | -0.692 | 0.833 | 1.616 | 3.980 | 1.810 | 1.410 | 3.241 | 1.522 |
| nsp3 | aa1353-1367 | TRCLNRVCTNYMPYF | -0.395 | 0.703 | -0.240 | -0.373 | -0.620 | 0.258 | 1.919 | 3.730 | 0.902 | 0.413 | 3.230 | 1.313 |
| nsp3 | aa1387-1401 | ASMPTTIAKNTVKSV | -0.857 | -0.060 | -0.852 | -0.876 | -0.828 | -0.695 | -0.876 | -0.803 | -0.811 | -0.829 | -0.763 | 3.056 |
| nsp3 | aa1399-1413 | KSVGKFCLEASFNYL | -0.419 | 0.822 | -0.102 | -0.665 | -0.764 | -0.309 | 0.470 | 3.270 | 1.230 | 0.470 | 2.237 | 0.377 |
| nsp3 | aa1447-1461 | VLMSNLGMPSYCTGY | -0.028 | 0.045 | -0.665 | -0.153 | -0.587 | 1.081 | 0.030 | 3.090 | 2.850 | -0.274 | 2.574 | 0.956 |
| nsp3 | aa1451-1465 | NLGMPSYCTGYREGY | 0.492 | 1.388 | 0.800 | 0.786 | 0.888 | 2.171 | 1.709 | 4.610 | 2.250 | 1.191 | 4.446 | 3.290 |
| nsp3 | aa1453-1467 | GMPSYCTGYREGYLN | -0.209 | 0.871 | -0.526 | 0.224 | -0.531 | 1.301 | 0.680 | 3.990 | 1.020 | 0.430 | 3.781 | 1.951 |
| nsp3 | aa1483-1497 | CSVCLSGLDSLDTYP | -0.172 | 0.239 | 0.163 | -0.018 | -0.784 | 1.441 | 0.315 | 3.720 | 0.510 | 0.906 | 1.252 | 0.747 |
| nsp3 | aa1509-1523 | FKWDLTAFGLVAEWF | 0.673 | 2.299 | 1.270 | 0.704 | 0.555 | 2.628 | 2.336 | 4.970 | 3.250 | 1.376 | 4.277 | 3.492 |
| nsp3 | aa1515-1529 | AFGLVAEWFLAYILF | -0.569 | 0.870 | 0.081 | -0.680 | -0.198 | 0.415 | 1.058 | 3.570 | 2.350 | 0.158 | 3.158 | 1.979 |
| nsp3 | aa1535-1549 | VLGLAAIMQLFFSYF | -0.570 | 0.929 | 0.569 | -0.630 | -0.740 | 0.323 | 0.931 | 3.510 | 2.230 | 0.559 | 3.257 | 2.155 |
| nsp3 | aa1547-1561 | SYFAVHFISNSWLMW | -0.400 | 1.060 | 0.741 | 0.503 | -0.114 | 3.200 | 1.406 | 3.480 | 3.230 | 0.592 | 3.955 | 3.168 |
| nsp3 | aa1549-1563 | FAVHFISNSWLMWLI | 0.071 | 0.532 | 0.452 | -0.061 | -0.451 | 2.382 | 0.501 | 3.230 | 1.950 | 1.080 | 3.497 | 2.422 |
| nsp3 | aa1573-1587 | SAMVRMYIFFASFYY | -0.145 | 0.967 | 0.046 | -0.315 | -0.612 | 0.175 | 0.949 | 4.020 | 1.460 | 0.091 | 3.071 | 1.449 |
| nsp3 | aa1575-1589 | MVRMYIFFASFYYVW | -0.110 | 0.549 | -0.306 | -0.400 | -0.272 | 1.143 | 0.888 | 3.570 | 0.765 | -0.482 | 2.898 | 1.819 |
| nsp3 | aa1585-1599 | FYYVWKSYVHVVDGC | -0.291 | -0.106 | 0.550 | -0.164 | -0.549 | 0.433 | 0.407 | 2.330 | -0.009 | 3.884 | 0.727 | 1.694 |
| nsp3 | aa1631-1645 | VYANGGKGFCKLHNW | -0.389 | -0.167 | -0.347 | -0.277 | -0.719 | 1.208 | 0.377 | 3.420 | 0.963 | -0.296 | 3.734 | 1.394 |
| nsp3 | aa1637-1651 | KGFCKLHNWNCVNCD | -0.591 | -0.146 | 0.006 | -0.593 | -0.560 | -0.227 | 0.270 | 3.000 | 0.808 | 4.140 | 0.224 | 0.866 |
| nsp3 | aa1639-1653 | FCKLHNWNCVNCDTF | -0.762 | 0.394 | -0.502 | -0.582 | -0.569 | -0.401 | 0.576 | 3.160 | 0.310 | 1.686 | 2.112 | 2.543 |
| nsp3 | aa1653-1667 | FCAGSTFISDEVARD | 0.380 | -0.281 | -0.107 | -0.333 | -0.447 | 0.734 | -0.003 | 1.490 | 1.470 | 0.995 | 0.800 | 3.007 |
| nsp3 | aa1687-1701 | DSVTVKNGSIHLYFD | 0.160 | 0.760 | 0.829 | 0.275 | -0.719 | 0.366 | 3.160 | 4.310 | 2.870 | 0.541 | 3.123 | 0.880 |
| nsp3 | aa1697-1711 | HLYFDKAGQKTYERH | -0.769 | -0.354 | -0.093 | -0.703 | -0.783 | -0.208 | 0.051 | 4.290 | 1.550 | -0.456 | 2.604 | 2.356 |
| nsp3 | aa1699-1713 | YFDKAGQKTYERHSL | -0.678 | -0.627 | -0.643 | -0.505 | -0.846 | -0.332 | -0.327 | 3.470 | 0.841 | -0.738 | 1.842 | 1.292 |
| nsp3 | aa1709-1723 | ERHSLSHFVNLDNLR | -0.655 | 0.238 | -0.118 | -0.376 | -0.389 | -0.308 | 0.693 | 4.000 | 0.426 | -0.592 | 3.250 | 1.337 |
| nsp3 | aa1711-1725 | HSLSHFVNLDNLRAN | -0.842 | -0.705 | -0.771 | -0.792 | -0.376 | -0.447 | 0.042 | 3.370 | -0.278 | -0.835 | 1.508 | 1.390 |
| nsp3 | aa1775-1789 | VGDSAEVAVKMFDAY | -0.413 | 0.553 | -0.269 | -0.404 | -0.297 | 1.168 | 0.454 | 3.510 | 1.800 | -0.336 | 3.314 | 2.448 |
| nsp3 | aa1835-1849 | GFVDSDVETKDVVEC | 0.792 | 1.739 | 0.893 | 0.753 | 0.636 | 2.101 | 0.846 | 3.690 | 1.900 | 2.548 | 0.978 | 2.832 |
| nsp3 | aa1859-1873 | EVTGDSCNNYMLTYN | -0.702 | -0.038 | 0.060 | -0.431 | -0.806 | 0.581 | 0.229 | 2.180 | 0.607 | -0.053 | 3.285 | 1.705 |
| nsp3 | aa1875-1889 | VENMTPRDLGACIDC | 0.887 | 1.258 | 1.450 | 0.749 | 0.401 | 1.533 | 1.498 | 3.290 | 1.980 | 3.743 | 1.536 | 2.493 |
| nsp4 | aa5-19 | NWLKQLIKVTLVFLF | -0.807 | -0.741 | -0.538 | -0.717 | -0.801 | -0.052 | -0.475 | 3.170 | 0.361 | -0.243 | 0.218 | 0.111 |
| nsp4 | aa9-23 | QLIKVTLVFLFVAAI | -0.683 | -0.797 | -0.772 | -0.853 | -0.862 | -0.407 | -0.632 | 1.540 | 0.049 | -0.614 | 3.664 | -0.667 |
| nsp4 | aa11-25 | IKVTLVFLFVAAIFY | -0.452 | -0.544 | -0.288 | -0.705 | -0.730 | 0.007 | -0.202 | 3.110 | 1.310 | -0.286 | 1.213 | 0.305 |
| nsp4 | aa41-55 | SEIIGYKAIDGGVTR | -0.366 | -0.303 | -0.669 | -0.367 | -0.254 | 0.640 | -0.628 | 0.214 | 0.896 | 3.042 | -0.035 | 2.334 |
| nsp4 | aa61-75 | DTCFANKHADFDTWF | 0.597 | 2.361 | 1.500 | 0.824 | -0.395 | 1.626 | 2.504 | 5.110 | 3.030 | 1.276 | 4.859 | 3.268 |
| nsp4 | aa107-121 | PGTILRTTNGDFLHF | -0.696 | 0.794 | 0.086 | -0.489 | -0.751 | -0.139 | 1.487 | 3.840 | 1.880 | 0.819 | 4.125 | 1.350 |
| nsp4 | aa117-131 | DFLHFLPRVFSAVGN | -0.773 | -0.727 | -0.519 | -0.856 | -0.865 | 0.068 | 0.663 | 5.600 | 0.461 | -0.328 | 0.452 | -0.046 |
| nsp4 | aa131-145 | NICYTPSKLIEYTDF | 0.382 | 0.388 | 1.460 | 0.124 | -0.323 | 0.958 | 1.046 | 3.610 | 1.600 | 0.559 | 2.612 | 1.463 |
| nsp4 | aa137-151 | SKLIEYTDFATSACV | -0.559 | -0.110 | 0.866 | 0.612 | -0.455 | -0.433 | 1.059 | 6.740 | 1.390 | 0.704 | -0.003 | -0.017 |
| nsp4 | aa145-159 | FATSACVLAAECTIF | 0.261 | 0.872 | 0.234 | 1.281 | -0.375 | -0.148 | 1.533 | 3.530 | 1.780 | 0.636 | 1.245 | 0.719 |
| nsp4 | aa157-171 | TIFKDASGKPVPYCY | -0.170 | -0.077 | 0.173 | -0.563 | -0.397 | 0.450 | -0.007 | 3.140 | 1.770 | 0.645 | 2.101 | 1.467 |
| nsp4 | aa177-191 | EGSVAYESLRPDTRY | -0.189 | 0.453 | 0.982 | 0.285 | -0.286 | 1.584 | 1.704 | 3.580 | 1.510 | 1.249 | 3.524 | 2.485 |
| nsp4 | aa207-221 | EGSVRVVTTFDSEYC | 1.988 | 2.595 | 1.870 | 1.367 | 1.086 | 1.775 | 2.016 | 4.710 | 3.720 | 3.201 | 3.102 | 2.796 |
| nsp4 | aa209-223 | SVRVVTTFDSEYCRH | -0.202 | 0.107 | 0.209 | -0.392 | -0.767 | -0.415 | 1.245 | 3.320 | 1.320 | 2.118 | 3.570 | 0.569 |
| nsp4 | aa227-241 | ERSEAGVCVSTSGRW | 0.101 | 0.342 | 0.495 | 0.094 | 0.360 | 2.398 | 0.590 | 3.190 | 2.570 | 0.277 | 3.320 | 2.445 |
| nsp4 | aa233-247 | VCVSTSGRWVLNNDY | 0.145 | 1.015 | 0.671 | -0.463 | -0.385 | -0.278 | 0.898 | 3.880 | 1.050 | 0.500 | 2.571 | 2.162 |
| nsp4 | aa235-249 | VSTSGRWVLNNDYYR | 0.407 | 1.290 | 0.459 | -0.040 | -0.055 | 0.885 | 1.215 | 4.100 | 2.250 | 0.814 | 4.231 | 2.161 |
| nsp4 | aa241-255 | WVLNNDYYRSLPGVF | -0.413 | -0.155 | -0.146 | -0.336 | -0.387 | 1.378 | 0.748 | 3.330 | 1.040 | -0.033 | 3.304 | 1.543 |
| nsp4 | aa297-311 | LAYYFMRFRRAFGEY | 0.648 | 1.436 | 1.060 | 0.397 | 0.324 | 1.569 | 1.690 | 3.970 | 2.200 | 1.618 | 3.769 | 3.193 |
| nsp4 | aa299-313 | YYFMRFRRAFGEYSH | 0.206 | 1.131 | 0.365 | -0.281 | -0.714 | 0.265 | 1.214 | 3.900 | 1.340 | 0.192 | 4.114 | 1.844 |
| nsp4 | aa309-323 | GEYSHVVAFNTLLFL | -0.753 | 1.029 | -0.518 | -0.809 | -0.773 | -0.232 | 0.344 | 3.020 | 1.100 | -0.318 | 0.858 | 0.561 |
| nsp4 | aa351-365 | FYLTNDVSFLAHIQW | -0.321 | 0.723 | 0.615 | -0.485 | -0.730 | 0.126 | 0.989 | 4.120 | 2.200 | 0.637 | 4.404 | 1.991 |
| nsp4 | aa355-369 | NDVSFLAHIQWMVMF | -0.248 | 0.474 | 0.500 | -0.116 | -0.424 | 1.438 | 0.782 | 3.620 | 1.580 | 0.869 | 3.158 | 2.938 |
| nsp4 | aa361-375 | AHIQWMVMFTPLVPF | -0.717 | 0.286 | -0.285 | -0.779 | -0.796 | 1.279 | 0.943 | 3.190 | 1.060 | 0.042 | 2.766 | 1.850 |
| nsp4 | aa363-377 | IQWMVMFTPLVPFWI | -0.456 | 0.693 | 0.553 | -0.460 | -0.569 | 1.756 | 0.933 | 3.330 | 1.620 | 0.309 | 2.414 | 2.101 |
| nsp4 | aa389-403 | HFYWFFSNYLKRRVV | -0.518 | -0.700 | -0.718 | -0.581 | -0.543 | 0.099 | -0.803 | 3.400 | -0.471 | -0.678 | 1.035 | -0.256 |
| nsp4 | aa407-421 | VSFSTFEEAALCTFL | -0.472 | 0.785 | -0.143 | 1.001 | -0.423 | 1.285 | 1.287 | 3.440 | 1.560 | 0.440 | 1.024 | -0.062 |
| nsp4 | aa409-423 | FSTFEEAALCTFLLN | -0.554 | -0.370 | -0.651 | -0.441 | -0.518 | 0.854 | -0.411 | 3.240 | 1.280 | 0.183 | 1.090 | 0.148 |
| nsp4 | aa413-427 | EEAALCTFLLNKEMY | 0.379 | -0.435 | -0.130 | -0.452 | -0.212 | 1.758 | 0.492 | 3.460 | 2.070 | -0.375 | 1.555 | 1.489 |
| nsp4 | aa455-469 | SGAMDTTSYREAACC | 0.317 | 0.021 | 2.750 | 0.270 | -0.245 | 1.924 | 0.450 | 2.240 | 2.400 | 3.190 | 2.103 | 0.958 |
| nsp4 | aa473-487 | KALNDFSNSGSDVLY | 0.060 | 0.995 | 0.462 | -0.389 | -0.430 | 0.795 | 1.274 | 3.070 | 2.120 | 0.789 | 3.365 | 1.236 |
| nsp5 | aa19-33 | QVTCGTTTLNGLWLD | 0.464 | 0.462 | 0.444 | 0.145 | 0.179 | 2.200 | 1.094 | 3.180 | 2.430 | 0.755 | 2.608 | 0.789 |
| nsp5 | aa21-35 | TCGTTTLNGLWLDDV | 0.847 | 0.088 | 0.746 | -0.089 | 0.392 | 1.263 | 0.207 | 3.070 | 0.924 | 0.389 | 1.457 | 2.361 |
| nsp5 | aa23-37 | GTTTLNGLWLDDVVY | 1.314 | 1.587 | 2.200 | 0.890 | 0.331 | 2.390 | 1.966 | 4.310 | 3.150 | 1.668 | 3.851 | 2.291 |
| nsp5 | aa25-39 | TTLNGLWLDDVVYCP | 0.982 | 0.673 | 2.040 | 0.816 | -0.307 | 0.134 | 0.433 | 3.900 | 2.350 | 0.674 | 3.320 | 0.823 |
| nsp5 | aa27-41 | LNGLWLDDVVYCPRH | -0.460 | -0.573 | -0.204 | -0.719 | -0.792 | 0.173 | -0.041 | 3.650 | 2.080 | -0.561 | 3.595 | 0.703 |
| nsp5 | aa41-55 | HVICTSEDMLNPNYE | -0.457 | 0.183 | -0.313 | -0.649 | -0.721 | 0.321 | 0.125 | 3.460 | 1.760 | 0.962 | 0.203 | 1.098 |
| nsp5 | aa43-57 | ICTSEDMLNPNYEDL | 0.237 | 0.727 | 0.251 | 0.237 | 0.388 | 1.531 | 1.067 | 3.380 | 1.990 | 1.703 | 0.043 | 3.765 |
| nsp5 | aa45-59 | TSEDMLNPNYEDLLI | 0.537 | 1.393 | 0.955 | 0.922 | 0.190 | 1.630 | 1.513 | 3.890 | 2.820 | 1.758 | 3.792 | 3.609 |
| nsp5 | aa131-145 | RPNFTIKGSFLNGSC | -0.522 | -0.658 | -0.403 | -0.845 | -0.691 | -0.001 | -0.783 | -0.002 | -0.210 | 3.530 | 0.401 | -0.016 |
| nsp5 | aa141-155 | LNGSCGSVGFNIDYD | 1.564 | 1.640 | 1.490 | -0.133 | 0.240 | 0.794 | 2.236 | 4.670 | 2.390 | 1.846 | 2.941 | 3.418 |
| nsp5 | aa143-157 | GSCGSVGFNIDYDCV | 0.932 | 1.461 | 1.740 | 0.067 | -0.055 | 1.825 | 1.267 | 4.200 | 2.380 | 2.546 | 2.616 | 2.542 |
| nsp5 | aa145-159 | CGSVGFNIDYDCVSF | 1.108 | 1.651 | 1.640 | 0.733 | -0.057 | 0.727 | 2.118 | 4.640 | 2.980 | 1.491 | 3.775 | 2.649 |
| nsp5 | aa147-161 | SVGFNIDYDCVSFCY | 2.129 | 2.603 | 2.650 | 1.663 | 0.950 | 1.295 | 2.322 | 5.020 | 4.020 | 2.899 | 4.086 | 2.935 |
| nsp5 | aa149-163 | GFNIDYDCVSFCYMH | 0.325 | 0.958 | -0.242 | -0.586 | -0.664 | 0.981 | 0.577 | 4.180 | 2.720 | -0.331 | 3.370 | 1.855 |
| nsp5 | aa151-165 | NIDYDCVSFCYMHHM | -0.148 | -0.626 | -0.507 | -0.742 | -0.744 | 0.879 | -0.175 | 3.800 | 0.891 | -0.675 | 2.310 | 2.771 |
| nsp5 | aa153-167 | DYDCVSFCYMHHMEL | 0.140 | -0.523 | -0.212 | -0.566 | -0.787 | 1.511 | -0.570 | 3.210 | 1.730 | -0.565 | 1.869 | 2.495 |
| nsp5 | aa167-181 | LPTGVHAGTDLEGNF | -0.749 | -0.318 | -0.186 | -0.735 | -0.791 | 0.900 | 0.199 | 3.260 | 2.230 | -0.004 | 1.064 | 2.131 |
| nsp5 | aa169-183 | TGVHAGTDLEGNFYG | -0.705 | -0.315 | 0.941 | -0.729 | -0.732 | 0.532 | 1.103 | 3.270 | 2.900 | 0.531 | 1.846 | 1.347 |
| nsp5 | aa171-185 | VHAGTDLEGNFYGPF | -0.710 | 1.114 | 0.064 | -0.010 | -0.769 | 0.712 | 1.744 | 3.890 | 2.950 | 1.269 | 3.433 | 2.850 |
| nsp5 | aa193-207 | AAGTDTTITVNVLAW | -0.226 | 0.587 | -0.222 | -0.271 | -0.486 | 0.993 | -0.373 | 3.120 | 2.030 | -0.152 | 1.150 | 0.680 |
| nsp5 | aa195-209 | GTDTTITVNVLAWLY | -0.348 | 1.966 | 0.465 | -0.228 | -0.114 | 1.869 | 0.308 | 3.380 | 1.280 | 0.547 | 2.652 | 1.734 |
| nsp5 | aa205-219 | LAWLYAAVINGDRWF | 0.071 | 1.744 | 1.040 | 0.458 | -0.016 | 2.250 | 2.096 | 4.730 | 3.030 | 1.321 | 4.807 | 2.516 |
| nsp5 | aa209-223 | YAAVINGDRWFLNRF | -0.611 | 0.818 | 0.040 | -0.200 | -0.520 | 1.116 | 1.677 | 3.380 | 2.000 | 0.588 | 4.010 | 2.128 |
| nsp5 | aa263-277 | DMCASLKELLQNGMN | -0.599 | -0.571 | -0.625 | -0.826 | -0.802 | 0.454 | -0.546 | 4.190 | -0.012 | -0.738 | -0.150 | 1.357 |
| nsp5 | aa277-291 | NGRTILGSALLEDEF | 0.619 | 1.021 | 1.020 | 0.300 | 0.513 | 1.766 | 1.204 | 3.960 | 1.850 | 1.633 | 2.354 | 2.703 |
| nsp5 | aa281-295 | ILGSALLEDEFTPFD | 1.138 | 1.643 | 1.630 | -0.077 | 0.378 | 1.914 | 1.549 | 4.420 | 2.140 | 2.634 | 2.277 | 1.586 |
| nsp5 | aa283-297 | GSALLEDEFTPFDVV | 0.393 | 0.272 | 0.696 | -0.630 | -0.266 | 1.300 | -0.137 | 3.330 | 1.970 | 1.141 | 1.359 | 2.458 |
| nsp5 | aa285-299 | ALLEDEFTPFDVVRQ | 0.677 | 0.296 | 0.739 | -0.748 | -0.345 | 1.177 | -0.432 | 3.710 | 2.290 | 1.032 | 2.710 | 2.286 |
| nsp5 | aa293-307 | PFDVVRQCSGVTFQS | -0.734 | -0.555 | -0.712 | -0.729 | -0.728 | 3.675 | -0.764 | 0.819 | -0.477 | -0.804 | 0.004 | -0.483 |
| nsp6 | aa21-35 | SLLVLVQSTQWSLFF | -0.568 | -0.179 | 0.082 | -0.450 | -0.690 | 0.964 | 0.718 | 3.510 | 1.200 | 0.166 | 2.312 | 0.887 |
| nsp6 | aa25-39 | LVQSTQWSLFFFLYE | 1.392 | 1.733 | 1.630 | 1.610 | 0.525 | 1.902 | 1.886 | 4.070 | 2.550 | 2.049 | 2.790 | 1.528 |
| nsp6 | aa31-45 | WSLFFFLYENAFLPF | -0.529 | 1.355 | 0.692 | 0.048 | -0.500 | -0.226 | 1.882 | 3.900 | 0.744 | 0.890 | 3.574 | 1.727 |
| nsp6 | aa45-59 | FAMGIIAMSAFAMMF | -0.177 | -0.115 | 0.093 | 0.129 | -0.046 | 2.558 | 0.370 | 2.230 | 1.560 | 0.541 | 1.707 | 3.798 |
| nsp6 | aa81-95 | FNMVYMPASWVMRIM | 0.079 | -0.235 | 0.162 | 0.203 | 0.103 | 2.459 | -0.262 | 0.315 | 1.490 | -0.595 | 3.257 | 2.859 |
| nsp6 | aa83-97 | MVYMPASWVMRIMTW | 0.264 | 0.347 | 0.394 | 0.261 | 0.100 | 2.834 | 0.426 | 2.390 | 2.390 | -0.209 | 3.410 | 3.295 |
| nsp6 | aa119-133 | AVVLLILMTARTVYD | -0.001 | 0.079 | 0.708 | -0.193 | -0.267 | 0.289 | 1.091 | 3.100 | 2.570 | -0.047 | 0.972 | -0.184 |
| nsp6 | aa123-137 | LILMTARTVYDDGAR | -0.593 | 0.683 | 0.308 | -0.081 | -0.359 | 1.920 | 0.474 | 3.010 | 2.390 | 0.258 | 2.036 | 2.521 |
| nsp6 | aa129-143 | RTVYDDGARRVWTLM | -0.051 | 0.565 | 0.157 | 0.230 | -0.353 | 2.845 | 0.409 | 2.250 | 3.040 | -0.107 | 2.483 | 2.716 |
| nsp6 | aa151-165 | KVYYGNALDQAISMW | 0.269 | 0.733 | 0.697 | 0.343 | -0.102 | 1.801 | 0.851 | 3.590 | 2.840 | 0.291 | 3.383 | 2.659 |
| nsp6 | aa155-169 | GNALDQAISMWALII | -0.648 | 0.204 | -0.364 | -0.372 | -0.743 | 0.582 | 0.509 | 3.090 | 1.350 | 0.241 | 0.259 | 1.079 |
| nsp6 | aa183-197 | MFLARGIVFMCVEYC | 1.496 | 2.554 | 2.010 | 1.435 | 0.625 | 1.626 | 2.088 | 4.500 | 3.210 | 3.007 | 3.174 | 2.457 |
| nsp6 | aa187-201 | RGIVFMCVEYCPIFF | -0.387 | 1.384 | 0.682 | 0.596 | -0.236 | 1.300 | 2.133 | 4.410 | 2.030 | 1.502 | 3.554 | 1.081 |
| nsp6 | aa191-205 | FMCVEYCPIFFITGN | -0.398 | -0.625 | -0.415 | -0.735 | -0.777 | -0.272 | -0.638 | 4.910 | 0.043 | -0.355 | 0.205 | -0.218 |
| nsp6 | aa207-221 | LQCIMLVYCFLGYFC | 0.169 | 0.468 | 0.695 | -0.119 | -0.354 | 1.156 | 0.415 | 3.180 | 1.490 | 1.677 | 2.639 | 1.242 |
| nsp6 | aa211-225 | MLVYCFLGYFCTCYF | 0.272 | 0.892 | 0.857 | -0.216 | -0.396 | -0.385 | 0.969 | 3.820 | 0.707 | 0.667 | 2.314 | 1.621 |
| nsp6 | aa215-229 | CFLGYFCTCYFGLFC | -0.051 | 0.631 | 0.742 | -0.126 | -0.097 | 0.873 | 0.685 | 3.130 | 0.482 | 1.392 | 2.021 | 2.001 |
| nsp6 | aa221-235 | CTCYFGLFCLLNRYF | -0.706 | 0.342 | -0.230 | -0.290 | -0.517 | -0.425 | 0.595 | 3.280 | 0.485 | -0.337 | 2.740 | 1.458 |
| nsp6 | aa229-243 | CLLNRYFRLTLGVYD | 0.853 | 1.429 | 0.909 | -0.220 | -0.471 | 0.600 | 2.161 | 4.040 | 2.220 | 0.896 | 1.700 | 0.761 |
| nsp6 | aa231-245 | LNRYFRLTLGVYDYL | 0.646 | 1.964 | 1.010 | -0.038 | -0.156 | 0.932 | 1.420 | 4.240 | 2.740 | 0.502 | 3.114 | 1.886 |
| nsp6 | aa237-251 | LTLGVYDYLVSTQEF | 0.577 | 1.123 | 1.200 | -0.471 | -0.359 | 0.655 | 1.282 | 3.560 | 2.190 | 0.887 | 2.698 | 2.339 |
| nsp6 | aa239-253 | LGVYDYLVSTQEFRY | 0.006 | 1.643 | 1.500 | 0.091 | 0.067 | 1.583 | 2.175 | 4.680 | 2.240 | 1.381 | 4.152 | 3.302 |
| nsp7 | aa15-29 | SVLQQLRVESSSKLW | -0.341 | -0.267 | -0.084 | -0.211 | -0.304 | 1.702 | 0.126 | 3.060 | 0.668 | -0.728 | 1.157 | 1.280 |
| nsp7 | aa27-41 | KLWAQCVQLHNDILL | -0.794 | -0.068 | -0.009 | -0.753 | -0.791 | -0.509 | -0.084 | 3.060 | 0.058 | -0.354 | 1.261 | 0.435 |
| nsp8 | aa13-27 | AFATAQEAYEQAVAN | -0.777 | -0.360 | -0.759 | -0.749 | 3.309 | -0.499 | -0.700 | 3.350 | 0.498 | -0.193 | -0.189 | 1.522 |
| nsp8 | aa15-29 | ATAQEAYEQAVANGD | -0.570 | 0.258 | -0.370 | -0.535 | -0.830 | -0.105 | 0.682 | 3.300 | 0.583 | -0.196 | 0.673 | 0.295 |
| nsp8 | aa17-31 | AQEAYEQAVANGDSE | -0.554 | 0.423 | -0.251 | -0.694 | -0.758 | 0.492 | -0.530 | 3.750 | -0.101 | -0.108 | 0.055 | 0.744 |
| nsp8 | aa19-33 | EAYEQAVANGDSEVV | -0.424 | 0.348 | 0.078 | -0.418 | -0.761 | -0.133 | -0.463 | 4.670 | 0.037 | 0.143 | -0.216 | 0.852 |
| nsp8 | aa79-93 | RAKVTSAMQTMLFTM | -0.548 | -0.814 | -0.146 | -0.079 | -0.379 | 1.738 | -0.811 | 3.320 | 1.040 | -0.826 | 0.049 | 2.356 |
| nsp8 | aa99-113 | NDALNNIINNARDGC | -0.452 | -0.192 | 0.703 | -0.221 | -0.465 | 0.319 | -0.303 | 0.919 | 1.240 | 3.146 | 1.609 | 1.690 |
| nsp8 | aa139-153 | NTCDGTTFTYASALW | -0.319 | 0.266 | 0.320 | -0.279 | -0.207 | 2.014 | 0.705 | 3.380 | 2.640 | 0.288 | 2.576 | 1.591 |
| nsp8 | aa141-155 | CDGTTFTYASALWEI | 0.273 | 0.944 | 1.590 | 0.578 | 0.301 | 1.556 | 1.398 | 4.040 | 2.980 | 0.972 | 2.790 | 2.249 |
| nsp8 | aa163-177 | SKIVQLSEISMDNSP | -0.738 | -0.638 | -0.749 | -0.809 | -0.798 | -0.258 | -0.603 | 5.370 | -0.525 | -0.444 | -0.406 | -0.575 |
| nsp8 | aa167-181 | QLSEISMDNSPNLAW | -0.053 | 1.174 | 0.559 | 0.260 | -0.021 | 2.059 | 0.931 | 2.480 | 1.960 | 0.463 | 3.034 | 2.224 |
| nsp9 | aa17-31 | TTQTACTDDNALAYY | 0.198 | 1.457 | 0.753 | -0.113 | -0.568 | -0.275 | 1.491 | 4.230 | 2.620 | 1.500 | 0.986 | 1.118 |
| nsp9 | aa41-55 | LALLSDLQDLKWARF | -0.787 | 0.253 | -0.568 | -0.669 | -0.453 | 1.604 | 0.518 | 3.400 | 1.220 | -0.273 | 3.292 | 2.329 |
| nsp9 | aa101-115 | VLGSLAATVRLQAGN | -0.774 | -0.758 | -0.737 | -0.847 | 1.277 | -0.219 | -0.733 | 6.170 | -0.481 | -0.740 | -0.272 | -0.268 |
| nsp9 | aa105-119 | LAATVRLQAGNATEV | -0.830 | -0.372 | -0.433 | -0.846 | -0.864 | -0.274 | -0.722 | 4.000 | -0.634 | -0.643 | -0.239 | -0.783 |
| nsp10 | aa17-31 | CAFAVDAAKAYKDYL | -0.281 | -0.504 | -0.716 | -0.803 | -0.695 | -0.369 | -0.472 | 3.100 | -0.104 | -0.669 | -0.007 | 1.207 |
| nsp10 | aa59-73 | PEANMDQESFGGASC | 3.192 | -0.430 | -0.507 | -0.701 | -0.587 | 2.667 | -0.516 | 0.380 | 0.803 | 1.189 | 0.333 | 1.442 |
| nsp10 | aa77-91 | CRCHIDHPNPKGFCD | -0.337 | 0.072 | 0.367 | 0.031 | -0.512 | 1.269 | 1.139 | 3.600 | 1.490 | 2.181 | 2.093 | 2.105 |
| nsp10 | aa83-97 | HPNPKGFCDLKGKYV | -0.797 | -0.587 | -0.752 | -0.741 | -0.767 | 0.139 | 0.023 | 3.160 | -0.089 | -0.745 | 2.047 | 0.117 |
| nsp10 | aa117-131 | CTVCGMWKGYGCSCD | 0.638 | 0.885 | 2.650 | 0.086 | 0.004 | 2.194 | 0.751 | 4.050 | 2.590 | 4.391 | 0.478 | 1.118 |
| nsp12 | aa19-33 | RLTPCGTGTSTDVVY | -0.740 | 0.500 | 0.936 | -0.792 | -0.825 | -0.253 | -0.558 | 3.060 | 0.286 | -0.461 | -0.034 | -0.191 |
| nsp12 | aa25-39 | TGTSTDVVYRAFDIY | 0.735 | 0.302 | 0.958 | -0.464 | 0.095 | 0.434 | 0.664 | 3.230 | 2.100 | 0.864 | 3.125 | 2.536 |
| nsp12 | aa27-41 | TSTDVVYRAFDIYND | 0.950 | 1.721 | 0.876 | 0.134 | 0.045 | 0.138 | 1.335 | 3.870 | 2.680 | 1.867 | 2.846 | 1.959 |
| nsp12 | aa49-63 | FLKTNCCRFQEKDED | 0.534 | -0.010 | -0.152 | -0.392 | -0.247 | 0.419 | 0.178 | 2.080 | 0.238 | -0.263 | 0.132 | 3.175 |
| nsp12 | aa57-71 | FQEKDEDDNLIDSYF | 1.545 | 2.508 | 1.970 | 1.047 | 0.577 | 1.753 | 2.337 | 5.090 | 3.760 | 2.233 | 3.323 | 2.508 |
| nsp12 | aa59-73 | EKDEDDNLIDSYFVV | 1.348 | 2.094 | 1.090 | 1.092 | 0.893 | 2.104 | 2.231 | 4.040 | 3.330 | 1.508 | 2.090 | 1.568 |
| nsp12 | aa83-97 | HEETIYNLLKDCPAV | 0.006 | -0.647 | -0.702 | -0.790 | -0.831 | -0.489 | 0.176 | 3.220 | -0.204 | -0.792 | 0.242 | -0.225 |
| nsp12 | aa89-103 | NLLKDCPAVAKHDFF | -0.518 | -0.101 | -0.148 | -0.128 | 0.133 | -0.529 | 1.088 | 3.570 | 1.360 | -0.450 | 2.465 | 1.584 |
| nsp12 | aa121-135 | TKYTMADLVYALRHF | -0.840 | -0.247 | -0.624 | -0.829 | -0.848 | -0.479 | 0.270 | 2.790 | -0.022 | -0.107 | 3.172 | 0.848 |
| nsp12 | aa123-137 | YTMADLVYALRHFDE | 0.045 | 0.305 | 1.160 | 0.112 | -0.711 | -0.127 | 0.854 | 3.130 | 1.220 | 1.006 | 1.642 | 2.165 |
| nsp12 | aa125-139 | MADLVYALRHFDEGN | -0.569 | -0.526 | -0.259 | -0.267 | -0.744 | -0.357 | 0.069 | 5.880 | -0.279 | 0.749 | 0.230 | 2.527 |
| nsp12 | aa127-141 | DLVYALRHFDEGNCD | 0.448 | 0.255 | 1.360 | 0.524 | -0.174 | 0.668 | 1.211 | 4.830 | 1.770 | 3.726 | 0.465 | 2.896 |
| nsp12 | aa139-153 | NCDTLKEILVTYNCC | -0.522 | 0.585 | -0.327 | 0.451 | -0.609 | -0.455 | -0.670 | 1.690 | 0.391 | 3.571 | -0.314 | 0.006 |
| nsp12 | aa141-155 | DTLKEILVTYNCCDD | 1.297 | 1.401 | 1.760 | 1.455 | 0.716 | -0.369 | 0.726 | 4.250 | 2.390 | 3.684 | -0.187 | 2.669 |
| nsp12 | aa143-157 | LKEILVTYNCCDDDY | 2.377 | 2.720 | 2.550 | 2.083 | 1.789 | 1.473 | 1.955 | 5.370 | 3.700 | 3.630 | 1.225 | 3.813 |
| nsp12 | aa145-159 | EILVTYNCCDDDYFN | 2.109 | 2.563 | 2.270 | 2.074 | 0.860 | 1.381 | 2.063 | 4.770 | 3.690 | 3.132 | 3.032 | 3.293 |
| nsp12 | aa149-163 | TYNCCDDDYFNKKDW | 1.249 | 1.137 | 0.475 | 1.027 | 0.018 | 2.764 | 1.806 | 4.370 | 2.420 | 1.105 | 3.086 | 2.830 |
| nsp12 | aa151-165 | NCCDDDYFNKKDWYD | 2.520 | 2.512 | 2.180 | 1.790 | 1.388 | 3.137 | 2.996 | 5.470 | 3.450 | 2.879 | 3.950 | 4.229 |
| nsp12 | aa153-167 | CDDDYFNKKDWYDFV | 1.182 | 1.700 | 1.510 | 0.995 | 1.338 | 2.499 | 2.024 | 4.870 | 2.540 | 1.849 | 4.103 | 4.337 |
| nsp12 | aa155-169 | DDYFNKKDWYDFVEN | 1.114 | 1.466 | 0.809 | 0.638 | 0.017 | 1.951 | 1.379 | 3.020 | 3.360 | 1.022 | 3.167 | 2.438 |
| nsp12 | aa203-217 | VGVLTLDNQDLNGNW | 0.338 | 1.066 | 0.313 | -0.116 | -0.079 | 1.847 | 1.292 | 3.850 | 2.490 | 0.221 | 2.982 | 2.880 |
| nsp12 | aa205-219 | VLTLDNQDLNGNWYD | 2.034 | 2.587 | 2.260 | 0.825 | 0.905 | 2.723 | 3.047 | 4.890 | 3.480 | 2.271 | 3.822 | 3.084 |
| nsp12 | aa209-223 | DNQDLNGNWYDFGDF | 1.920 | 2.915 | 2.500 | 1.569 | 1.004 | 2.366 | 2.953 | 5.080 | 3.570 | 2.104 | 4.512 | 3.528 |
| nsp12 | aa211-225 | QDLNGNWYDFGDFIQ | 0.101 | 1.348 | 0.897 | -0.022 | -0.203 | 0.783 | 1.389 | 3.630 | 3.210 | 0.565 | 2.989 | 1.589 |
| nsp12 | aa225-239 | QTTPGSGVPVVDSYY | -0.096 | 1.672 | 1.160 | -0.167 | -0.311 | -0.243 | 1.751 | 3.250 | 2.950 | 1.834 | 2.233 | 1.366 |
| nsp12 | aa257-271 | HVDTDLTKPYIKWDL | -0.301 | -0.622 | -0.627 | -0.748 | -0.362 | 0.719 | -0.348 | 3.790 | -0.142 | -0.602 | 1.665 | 1.251 |
| nsp12 | aa261-275 | DLTKPYIKWDLLKYD | 0.860 | -0.353 | 0.243 | -0.257 | -0.443 | 2.126 | 1.355 | 4.280 | 1.300 | 0.120 | 2.416 | 1.723 |
| nsp12 | aa265-279 | PYIKWDLLKYDFTEE | 0.758 | 0.337 | 0.333 | 0.025 | -0.004 | 1.565 | 0.698 | 3.050 | 0.873 | 1.000 | 1.155 | 1.465 |
| nsp12 | aa273-287 | KYDFTEERLKLFDRY | -0.274 | 0.737 | 0.723 | -0.049 | 0.021 | 0.951 | 1.114 | 3.790 | 1.100 | -0.020 | 3.961 | 3.205 |
| nsp12 | aa275-289 | DFTEERLKLFDRYFK | -0.328 | 0.124 | -0.443 | 0.011 | -0.703 | 0.191 | 0.393 | 3.320 | 1.170 | -0.033 | 1.654 | 0.395 |
| nsp12 | aa281-295 | LKLFDRYFKYWDQTY | -0.332 | 1.212 | 0.102 | -0.564 | -0.596 | 0.210 | 0.798 | 3.390 | 0.078 | 0.678 | 2.766 | 0.974 |
| nsp12 | aa293-307 | QTYHPNCVNCLDDRC | -0.276 | 0.029 | 0.402 | -0.342 | -0.094 | 0.555 | 0.488 | 3.330 | 2.370 | 2.861 | 2.315 | 1.915 |
| nsp12 | aa295-309 | YHPNCVNCLDDRCIL | -0.287 | 0.273 | 0.284 | -0.277 | -0.675 | 0.420 | 0.976 | 3.720 | 2.450 | 0.834 | 2.820 | 1.588 |
| nsp12 | aa311-325 | CANFNVLFSTVFPPT | -0.859 | 0.072 | -0.698 | -0.872 | -0.833 | -0.430 | -0.816 | -0.424 | -0.728 | 0.318 | 3.386 | -0.143 |
| nsp12 | aa349-363 | FRELGVVHNQDVNLH | -0.659 | -0.345 | -0.592 | -0.625 | -0.791 | -0.447 | -0.741 | 3.150 | 2.120 | -0.546 | 2.006 | 1.006 |
| nsp12 | aa357-371 | NQDVNLHSSRLSFKE | -0.628 | -0.663 | -0.723 | -0.772 | -0.835 | -0.360 | -0.335 | 0.112 | 0.050 | -0.664 | -0.153 | 3.728 |
| nsp12 | aa361-375 | NLHSSRLSFKELLVY | -0.558 | 0.550 | -0.377 | -0.556 | 0.011 | 0.105 | 0.957 | 3.490 | 1.400 | -0.134 | 1.781 | 1.037 |
| nsp12 | aa373-387 | LVYAADPAMHAASGN | -0.637 | -0.759 | -0.580 | -0.817 | -0.864 | -0.292 | -0.722 | 4.620 | -0.445 | -0.666 | -0.405 | -0.105 |
| nsp12 | aa401-415 | ALTNNVAFQTVKPGN | -0.795 | -0.744 | -0.786 | -0.860 | -0.822 | -0.431 | -0.782 | 4.970 | -0.562 | -0.670 | 0.795 | -0.457 |
| nsp12 | aa409-423 | QTVKPGNFNKDFYDF | 0.719 | 1.810 | 2.310 | 0.161 | -0.140 | 0.357 | 1.602 | 4.510 | 2.170 | 0.761 | 3.741 | 2.748 |
| nsp12 | aa429-443 | FFKEGSSVELKHFFF | -0.799 | -0.053 | -0.711 | -0.728 | -0.632 | -0.334 | -0.248 | 3.500 | -0.222 | -0.003 | 3.465 | 1.513 |
| nsp12 | aa441-455 | FFFAQDGNAAISDYD | 1.611 | 1.919 | 1.640 | 0.632 | 0.588 | -0.083 | 2.473 | 4.520 | 3.260 | 2.052 | 2.185 | 3.531 |
| nsp12 | aa443-457 | FAQDGNAAISDYDYY | 2.529 | 3.737 | 3.190 | 2.181 | 1.646 | 2.049 | 2.998 | 6.120 | 4.350 | 3.588 | 5.043 | 3.903 |
| nsp12 | aa445-459 | QDGNAAISDYDYYRY | 1.818 | 2.987 | 2.280 | 1.399 | 0.168 | 1.648 | 2.734 | 5.530 | 3.650 | 2.347 | 5.148 | 2.514 |
| nsp12 | aa447-461 | GNAAISDYDYYRYNL | -0.393 | 0.588 | 0.498 | -0.133 | -0.584 | 0.042 | 0.814 | 3.380 | 1.390 | 0.294 | 3.815 | 1.339 |
| nsp12 | aa451-465 | ISDYDYYRYNLPTMC | -0.733 | -0.531 | 0.137 | -0.441 | -0.808 | 0.799 | -0.536 | 3.130 | -0.125 | 1.069 | 0.311 | 1.114 |
| nsp12 | aa453-467 | DYDYYRYNLPTMCDI | 0.099 | 0.772 | 0.811 | -0.293 | 0.248 | 0.256 | 0.458 | 3.170 | 1.480 | 1.478 | 1.592 | 2.531 |
| nsp12 | aa463-477 | TMCDIRQLLFVVEVV | 1.788 | 2.437 | 1.930 | 0.571 | 0.578 | 0.767 | 1.260 | 3.050 | 1.940 | 1.467 | 0.549 | 0.382 |
| nsp12 | aa469-483 | QLLFVVEVVDKYFDC | 2.145 | 2.071 | 2.410 | 1.698 | 0.542 | 2.104 | 2.064 | 4.150 | 3.410 | 4.502 | 2.376 | 1.987 |
| nsp12 | aa471-485 | LFVVEVVDKYFDCYD | 3.069 | 3.526 | 3.170 | 2.190 | 1.691 | 2.508 | 2.960 | 6.150 | 4.590 | 3.359 | 4.153 | 3.254 |
| nsp12 | aa475-489 | EVVDKYFDCYDGGCI | 1.559 | 1.764 | 2.380 | 0.855 | 0.249 | 1.576 | 1.633 | 4.210 | 3.410 | 3.044 | 3.009 | 1.704 |
| nsp12 | aa493-507 | QVIVNNLDKSAGFPF | -0.272 | 0.264 | -0.415 | -0.680 | -0.768 | 0.198 | 1.078 | 3.050 | 0.843 | -0.191 | 2.150 | 1.534 |
| nsp12 | aa509-523 | KWGKARLYYDSMSYE | 0.673 | 1.230 | 1.260 | 0.597 | -0.033 | 2.514 | 1.631 | 3.950 | 2.420 | 1.704 | 2.687 | 2.487 |
| nsp12 | aa513-527 | ARLYYDSMSYEDQDA | 0.401 | 0.684 | 0.817 | 0.141 | -0.605 | 1.225 | 1.866 | 2.110 | 1.420 | 1.045 | 0.230 | 3.675 |
| nsp12 | aa515-529 | LYYDSMSYEDQDALF | 0.974 | 1.803 | 1.800 | 1.235 | 0.203 | 1.968 | 2.405 | 4.880 | 2.730 | 1.463 | 3.591 | 3.751 |
| nsp12 | aa517-531 | YDSMSYEDQDALFAY | 0.833 | 1.543 | 1.850 | 1.352 | 0.243 | 1.572 | 2.420 | 4.160 | 2.750 | 1.900 | 3.600 | 4.130 |
| nsp12 | aa585-599 | GATVVIGTSKFYGGW | -0.125 | 0.035 | -0.158 | -0.373 | -0.237 | 2.180 | -0.590 | 3.210 | 1.850 | -0.074 | 2.593 | 1.977 |
| nsp12 | aa605-619 | TVYSDVENPHLMGWD | 0.480 | 0.511 | 0.579 | -0.124 | -0.014 | 2.084 | 1.989 | 3.690 | 3.050 | 1.129 | 2.977 | 2.554 |
| nsp12 | aa607-621 | YSDVENPHLMGWDYP | -0.102 | 1.049 | 1.170 | 0.045 | 0.480 | 2.047 | 1.940 | 4.040 | 2.760 | 1.281 | 4.581 | 3.456 |
| nsp12 | aa661-675 | AQVLSEMVMCGGSLY | -0.150 | 0.500 | 0.316 | -0.355 | -0.356 | 1.342 | 1.152 | 3.610 | 1.730 | 0.956 | 2.380 | 1.675 |
| nsp12 | aa717-731 | ADKYVRNLQHRLYEC | 0.435 | 0.775 | 0.931 | -0.261 | 0.615 | 0.825 | 0.073 | 3.550 | 0.675 | 2.527 | 2.327 | 2.953 |
| nsp12 | aa719-733 | KYVRNLQHRLYECLY | 0.378 | 2.005 | 1.480 | 0.439 | -0.051 | 0.483 | 1.826 | 4.460 | 1.820 | 1.472 | 3.975 | 2.817 |
| nsp12 | aa723-737 | NLQHRLYECLYRNRD | -0.438 | 0.460 | -0.005 | -0.649 | -0.226 | 0.370 | 0.616 | 3.570 | 0.231 | 0.729 | 3.473 | 1.243 |
| nsp12 | aa727-741 | RLYECLYRNRDVDTD | -0.289 | -0.664 | -0.549 | -0.763 | -0.633 | 0.088 | -0.222 | 0.761 | -0.399 | -0.507 | 0.572 | 3.345 |
| nsp12 | aa729-743 | YECLYRNRDVDTDFV | 0.202 | 0.770 | 0.189 | -0.383 | 0.318 | 0.321 | 0.785 | 3.470 | 0.576 | 0.347 | 2.520 | 2.756 |
| nsp12 | aa731-745 | CLYRNRDVDTDFVNE | -0.103 | -0.286 | -0.549 | -0.649 | -0.686 | -0.328 | 0.536 | 0.859 | 0.841 | 0.412 | 0.233 | 3.070 |
| nsp12 | aa733-747 | YRNRDVDTDFVNEFY | 1.559 | 2.239 | 2.370 | 1.020 | 0.834 | 1.847 | 2.666 | 4.520 | 3.550 | 2.622 | 3.664 | 4.016 |
| nsp12 | aa735-749 | NRDVDTDFVNEFYAY | 1.518 | 2.120 | 1.900 | 0.994 | 0.811 | 1.498 | 2.370 | 4.230 | 2.670 | 1.675 | 3.544 | 3.258 |
| nsp12 | aa753-767 | HFSMMILSDDAVVCF | 0.312 | 1.227 | 0.538 | 1.048 | -0.663 | 0.166 | 1.584 | 3.930 | 2.130 | 0.547 | 2.458 | 1.609 |
| nsp12 | aa785-799 | SVLYYQNNVFMSEAK | -0.586 | -0.861 | -0.812 | -0.449 | -0.822 | -0.492 | -0.731 | 2.090 | 0.166 | -0.814 | 3.252 | 1.392 |
| nsp12 | aa787-801 | LYYQNNVFMSEAKCW | -0.094 | -0.171 | 0.284 | 0.222 | -0.171 | 1.565 | 0.685 | 3.610 | 2.240 | -0.179 | 4.087 | 1.907 |
| nsp12 | aa813-827 | FCSQHTMLVKQGDDY | -0.199 | 0.450 | 0.224 | -0.327 | -0.085 | 0.745 | -0.271 | 4.060 | 0.473 | -0.135 | 2.168 | 2.657 |
| nsp12 | aa815-829 | SQHTMLVKQGDDYVY | 0.364 | 0.994 | 0.566 | -0.092 | -0.238 | 0.790 | 0.769 | 4.100 | 1.890 | 0.298 | 2.407 | 1.674 |
| nsp12 | aa817-831 | HTMLVKQGDDYVYLP | -0.428 | 0.099 | -0.790 | -0.581 | -0.823 | 0.001 | 0.290 | 4.050 | 0.628 | -0.383 | 2.022 | 0.227 |
| nsp12 | aa819-833 | MLVKQGDDYVYLPYP | 0.042 | 1.082 | 0.308 | -0.428 | -0.374 | 0.286 | 1.419 | 3.540 | 1.750 | 0.677 | 3.140 | 1.435 |
| nsp12 | aa823-837 | QGDDYVYLPYPDPSR | -0.077 | 0.816 | 0.327 | -0.234 | -0.090 | 1.729 | 1.123 | 3.540 | 2.310 | 0.243 | 4.071 | 2.398 |
| nsp12 | aa829-843 | YLPYPDPSRILGAGC | -0.314 | -0.217 | 1.440 | 0.118 | -0.418 | 2.214 | -0.632 | 1.940 | 1.940 | 3.753 | 1.049 | 1.013 |
| nsp12 | aa835-849 | PSRILGAGCFVDDIV | 0.189 | 1.324 | 0.976 | -0.202 | 0.267 | -0.153 | 0.479 | 3.040 | 2.550 | 1.112 | 1.682 | 1.769 |
| nsp12 | aa855-869 | LMIERFVSLAIDAYP | 0.006 | 1.102 | 0.749 | -0.054 | -0.815 | -0.277 | 0.550 | 3.550 | 0.708 | 1.069 | 1.684 | 1.095 |
| nsp12 | aa869-883 | PLTKHPNQEYADVFH | -0.542 | 0.533 | -0.541 | -0.603 | -0.747 | -0.026 | 1.372 | 3.130 | 0.624 | -0.578 | 2.572 | 1.469 |
| nsp12 | aa871-885 | TKHPNQEYADVFHLY | -0.608 | 0.429 | -0.438 | -0.398 | -0.800 | 0.014 | 1.324 | 4.030 | 1.370 | -0.341 | 3.628 | 2.012 |
| nsp12 | aa873-887 | HPNQEYADVFHLYLQ | -0.237 | -0.608 | -0.591 | -0.790 | -0.810 | -0.388 | -0.072 | 3.750 | 0.685 | -0.628 | 2.407 | 1.407 |
| nsp12 | aa875-889 | NQEYADVFHLYLQYI | 0.218 | 0.741 | 1.260 | -0.378 | -0.597 | -0.358 | 0.295 | 3.080 | 1.590 | 0.684 | 2.261 | 0.909 |
| nsp12 | aa889-903 | IRKLHDELTGHMLDM | 0.318 | 0.985 | 0.224 | 0.177 | -0.822 | 2.012 | 0.650 | 3.900 | 2.250 | -0.073 | 2.123 | 3.162 |
| nsp12 | aa891-905 | KLHDELTGHMLDMYS | 0.166 | 1.307 | 0.373 | 0.002 | -0.773 | 1.805 | 1.101 | 3.740 | 2.420 | 0.026 | 2.602 | 3.194 |
| nsp12 | aa893-907 | HDELTGHMLDMYSVM | 0.119 | 0.819 | 0.310 | 0.078 | -0.710 | 2.144 | 0.188 | 4.010 | 2.360 | -0.483 | 2.904 | 3.836 |
| nsp12 | aa903-917 | MYSVMLTNDNTSRYW | -0.132 | 0.353 | -0.060 | -0.282 | -0.158 | 2.671 | 0.521 | 4.130 | 2.260 | -0.279 | 3.170 | 2.574 |
| nsp12 | aa907-921 | MLTNDNTSRYWEPEF | 1.783 | 1.497 | 1.680 | 0.911 | 0.174 | 2.752 | 1.839 | 3.930 | 2.430 | 1.100 | 3.358 | 2.761 |
| nsp12 | aa909-923 | TNDNTSRYWEPEFYE | 1.911 | 3.057 | 2.640 | 2.301 | 1.338 | 3.625 | 2.441 | 4.890 | 3.470 | 2.376 | 4.008 | 4.011 |
| nsp12 | aa911-925 | DNTSRYWEPEFYEAM | 0.606 | 1.447 | 1.320 | 0.685 | 0.627 | 2.825 | 1.813 | 3.940 | 2.590 | 1.511 | 3.192 | 3.261 |
| nsp12 | aa913-927 | TSRYWEPEFYEAMYT | 1.765 | 2.349 | 1.980 | 0.959 | 0.081 | 2.763 | 2.549 | 4.970 | 2.960 | 1.748 | 4.347 | 2.750 |
| nsp12 | aa915-929 | RYWEPEFYEAMYTPH | 1.934 | 1.473 | 1.040 | -0.056 | -0.668 | 1.891 | 2.070 | 5.020 | 2.390 | -0.037 | 4.656 | 3.453 |
| nsp12 | aa917-931 | WEPEFYEAMYTPHTV | 3.609 | 0.464 | -0.187 | -0.445 | -0.753 | 1.104 | 0.589 | 2.720 | 1.490 | -0.473 | 2.244 | 2.632 |
| nsp12 | aa919-933 | PEFYEAMYTPHTVLQ | 3.866 | 0.040 | -0.680 | -0.668 | -0.781 | 0.260 | -0.022 | 2.490 | 0.399 | -0.439 | 1.725 | 0.031 |
| nsp13 | aa19-33 | ACIRRPFLCCKCCYD | 0.929 | 0.639 | 0.649 | 0.654 | 0.213 | 0.696 | 1.432 | 3.800 | 1.620 | 1.755 | 2.349 | 1.361 |
| nsp13 | aa43-57 | VLSVNPYVCNAPGCD | -0.455 | -0.316 | 1.160 | -0.625 | -0.132 | 0.474 | -0.226 | 1.560 | 1.680 | 4.239 | 0.243 | 0.339 |
| nsp13 | aa51-65 | CNAPGCDVTDVTQLY | -0.656 | 0.059 | 0.617 | -0.758 | -0.707 | -0.414 | -0.044 | 3.110 | 0.037 | 0.701 | 3.004 | 0.966 |
| nsp13 | aa57-71 | DVTDVTQLYLGGMSY | -0.625 | 0.323 | 0.088 | -0.578 | -0.440 | 1.388 | -0.426 | 3.520 | 1.530 | 0.262 | 2.450 | 2.185 |
| nsp13 | aa59-73 | TDVTQLYLGGMSYYC | 0.005 | 1.303 | 4.030 | -0.075 | 0.081 | 0.520 | 0.900 | 4.190 | 2.640 | 1.647 | 2.717 | 2.225 |
| nsp13 | aa101-115 | SDNVTDFNAIATCDW | 0.361 | 1.175 | 1.100 | 0.923 | 0.417 | 1.742 | 1.249 | 4.260 | 2.040 | 1.107 | 2.301 | 2.799 |
| nsp13 | aa107-121 | FNAIATCDWTNAGDY | -0.378 | 0.836 | 0.453 | 0.385 | 0.044 | 0.678 | 0.815 | 3.700 | 1.660 | 0.137 | 1.499 | 1.946 |
| nsp13 | aa109-123 | AIATCDWTNAGDYIL | -0.528 | 1.158 | 0.096 | 0.069 | -0.664 | 0.110 | 0.739 | 4.060 | 2.320 | 0.061 | 1.607 | 1.379 |
| nsp13 | aa155-169 | VREVLSDRELHLSWE | 0.905 | 0.643 | 1.150 | 0.343 | 0.353 | 2.796 | 0.528 | 3.550 | 3.190 | 1.873 | 2.718 | 2.769 |
| nsp13 | aa165-179 | HLSWEVGKPRPPLNR | -0.384 | -0.346 | -0.614 | -0.685 | -0.599 | -0.092 | -0.519 | 3.050 | 1.430 | -0.755 | 3.064 | 1.901 |
| nsp13 | aa167-181 | SWEVGKPRPPLNRNY | -0.481 | -0.369 | -0.519 | -0.839 | -0.569 | -0.072 | -0.485 | 1.870 | 0.799 | -0.689 | 3.288 | 2.196 |
| nsp13 | aa197-211 | GEYTFEKGDYGDAVV | 0.718 | 0.881 | 0.739 | -0.313 | -0.595 | 1.274 | 0.443 | 3.140 | 3.300 | 1.081 | 1.324 | 2.227 |
| nsp13 | aa199-213 | YTFEKGDYGDAVVYR | 0.079 | -0.066 | 0.231 | -0.123 | -0.336 | 1.152 | 0.673 | 3.860 | 2.940 | -0.478 | 3.368 | 2.006 |
| nsp13 | aa241-255 | LVPQEHYVRITGLYP | -0.753 | -0.048 | -0.491 | -0.579 | -0.839 | 0.174 | 0.198 | 2.280 | 1.230 | -0.543 | 3.184 | 0.233 |
| nsp13 | aa313-327 | AAVDALCEKALKYLP | 0.074 | 0.916 | -0.541 | 0.085 | -0.788 | -0.328 | 0.550 | 3.130 | 0.915 | 0.338 | 1.703 | 0.090 |
| nsp13 | aa331-345 | CSRIIPARARVECFD | 0.545 | 0.527 | 0.741 | -0.068 | -0.065 | 1.472 | 0.801 | 3.080 | 1.860 | 0.328 | 1.357 | 1.113 |
| nsp13 | aa361-375 | VNALPETTADIVVFD | 0.271 | 1.304 | 0.770 | 1.452 | 0.343 | 0.796 | 0.985 | 3.180 | 1.860 | 0.882 | 0.854 | 1.441 |
| nsp13 | aa363-377 | ALPETTADIVVFDEI | 0.868 | 1.997 | 1.330 | 1.724 | 0.563 | 1.404 | 1.103 | 3.500 | 1.310 | 0.969 | 1.052 | 2.093 |
| nsp13 | aa365-379 | PETTADIVVFDEISM | 3.800 | 1.038 | 0.219 | 0.652 | -0.145 | 1.742 | 0.465 | 3.220 | 0.736 | 0.680 | 0.721 | 1.817 |
| nsp13 | aa369-383 | ADIVVFDEISMATNY | -0.489 | 0.913 | 0.407 | 0.271 | -0.781 | -0.249 | 0.207 | 4.620 | 0.594 | -0.252 | 0.405 | 1.888 |
| nsp13 | aa371-385 | IVVFDEISMATNYDL | -0.437 | 1.088 | 0.052 | 0.727 | -0.306 | 1.253 | 0.545 | 3.220 | 1.380 | 0.164 | 1.001 | 2.265 |
| nsp13 | aa409-423 | PRTLLTKGTLEPEYF | 1.250 | 1.490 | 1.040 | 0.099 | 0.087 | 1.025 | 1.344 | 4.150 | 1.880 | 1.338 | 3.113 | 2.878 |
| nsp13 | aa423-437 | FNSVCRLMKTIGPDM | -0.580 | -0.763 | -0.609 | -0.266 | -0.266 | 3.091 | -0.527 | 0.183 | -0.511 | -0.456 | 0.420 | 1.827 |
| nsp13 | aa445-459 | CPAEIVDTVSALVYD | 1.333 | 1.852 | 1.650 | 0.446 | 0.190 | 0.963 | 1.858 | 4.450 | 2.630 | 2.099 | 1.312 | 0.986 |
| nsp13 | aa493-507 | QIGVVREFLTRNPAW | -0.403 | 0.162 | -0.060 | -0.105 | -0.306 | 1.340 | 0.893 | 3.700 | 1.840 | -0.044 | 3.018 | 2.042 |
| nsp13 | aa531-545 | TQTVDSSQGSEYDYV | 0.960 | 1.944 | 1.390 | 0.801 | 0.056 | 0.527 | 1.915 | 4.400 | 2.050 | 1.709 | 2.212 | 2.299 |
| nsp13 | aa533-547 | TVDSSQGSEYDYVIF | 2.217 | 3.109 | 2.260 | 2.050 | 0.892 | 1.330 | 2.633 | 5.500 | 3.420 | 2.428 | 3.495 | 1.946 |
| nsp13 | aa569-583 | AKVGILCIMSDRDLY | 0.053 | 1.479 | 0.778 | 0.385 | -0.002 | 1.371 | 1.283 | 4.150 | 2.860 | 0.530 | 3.752 | 3.151 |
| nsp13 | aa571-585 | VGILCIMSDRDLYDK | -0.199 | 0.889 | -0.368 | 0.025 | -0.303 | 1.084 | 0.397 | 3.120 | 2.520 | -0.328 | 2.621 | 1.360 |
| nsp13 | aa579-593 | DRDLYDKLQFTSLEI | 0.349 | 0.601 | 0.471 | -0.257 | -0.464 | 0.329 | 1.116 | 4.630 | 1.560 | 1.110 | 2.173 | 1.593 |
| nsp14 | aa55-69 | ISMMGFKMNYQVNGY | -0.331 | 0.005 | -0.123 | -0.715 | -0.787 | 0.828 | 0.289 | 3.120 | 0.757 | -0.431 | 1.900 | 1.219 |
| nsp14 | aa59-73 | GFKMNYQVNGYPNMF | -0.597 | 0.312 | 0.036 | -0.697 | -0.744 | 0.299 | 0.486 | 3.050 | 0.497 | -0.146 | 3.266 | 1.889 |
| nsp14 | aa81-95 | RHVRAWIGFDVEGCH | -0.362 | 0.026 | 0.206 | -0.541 | -0.830 | -0.352 | 0.138 | 3.370 | 1.990 | 2.020 | 2.720 | 1.533 |
| nsp14 | aa145-159 | QFKHLIPLMYKGLPW | -0.261 | 0.663 | -0.058 | 0.084 | -0.166 | 1.931 | 1.190 | 3.820 | 2.190 | 0.618 | 3.887 | 1.726 |
| nsp14 | aa197-211 | YFVKIGPERTCCLCD | 1.284 | 0.687 | 1.380 | 0.547 | 0.117 | 1.070 | 1.050 | 3.870 | 2.470 | 3.434 | 0.821 | 1.024 |
| nsp14 | aa213-227 | RATCFSTASDTYACW | 0.131 | 0.259 | 0.348 | -0.164 | -0.019 | 1.383 | 0.209 | 3.390 | 1.610 | 1.041 | 1.778 | 1.999 |
| nsp14 | aa221-235 | SDTYACWHHSIGFDY | 0.106 | 1.587 | 1.660 | 0.186 | 0.739 | 1.071 | 1.695 | 4.590 | 2.290 | 1.636 | 4.241 | 3.129 |
| nsp14 | aa223-237 | TYACWHHSIGFDYVY | 0.484 | 1.963 | 1.600 | 0.590 | 0.027 | 1.339 | 2.054 | 4.940 | 2.590 | 1.909 | 4.660 | 2.138 |
| nsp14 | aa225-239 | ACWHHSIGFDYVYNP | -0.636 | 0.274 | -0.315 | -0.835 | -0.850 | -0.635 | -0.323 | 2.080 | 0.535 | -0.179 | 4.148 | 0.161 |
| nsp14 | aa227-241 | WHHSIGFDYVYNPFM | -0.645 | 0.083 | -0.279 | -0.506 | -0.745 | 0.472 | 0.352 | 3.490 | 1.450 | 0.063 | 4.427 | 1.997 |
| nsp14 | aa229-243 | HSIGFDYVYNPFMID | 0.014 | 0.335 | -0.034 | -0.288 | -0.624 | 0.306 | 0.773 | 3.300 | 1.860 | 0.325 | 1.495 | 2.683 |
| nsp14 | aa231-245 | IGFDYVYNPFMIDVQ | -0.469 | -0.276 | -0.199 | -0.287 | -0.583 | -0.267 | 0.456 | 1.960 | 1.760 | -0.624 | 0.300 | 3.047 |
| nsp14 | aa233-247 | FDYVYNPFMIDVQQW | 0.096 | 1.502 | 0.901 | 0.320 | -0.107 | 2.155 | 1.491 | 4.520 | 3.000 | 0.961 | 4.219 | 3.455 |
| nsp14 | aa235-249 | YVYNPFMIDVQQWGF | -0.276 | 1.375 | 0.162 | 0.157 | -0.250 | 2.172 | 1.215 | 3.650 | 2.550 | 0.693 | 3.550 | 2.583 |
| nsp14 | aa247-261 | WGFTGNLQSNHDLYC | 0.997 | 1.225 | 1.120 | 0.518 | -0.289 | 1.241 | 1.457 | 3.750 | 2.630 | 2.151 | 1.806 | 1.556 |
| nsp14 | aa273-287 | DAIMTRCLAVHECFV | -0.587 | -0.075 | -0.032 | 0.692 | -0.685 | -0.441 | 1.272 | 3.430 | 1.370 | 1.348 | 1.809 | 0.344 |
| nsp14 | aa283-297 | HECFVKRVDWTIEYP | 0.612 | 0.817 | 0.487 | 0.762 | -0.390 | 0.424 | 1.414 | 4.620 | 1.850 | 0.666 | 1.831 | 2.017 |
| nsp14 | aa285-299 | CFVKRVDWTIEYPII | -0.010 | 2.334 | 0.266 | 0.750 | -0.183 | 0.429 | 1.515 | 3.270 | 1.460 | 0.553 | 0.475 | 1.212 |
| nsp14 | aa289-303 | RVDWTIEYPIIGDEL | 0.613 | 1.354 | 0.677 | 0.944 | 0.556 | 2.075 | 1.106 | 3.170 | 1.940 | -0.043 | -0.122 | 2.470 |
| nsp14 | aa291-305 | DWTIEYPIIGDELKI | 0.484 | 0.336 | 0.031 | 0.367 | -0.058 | 1.188 | 1.553 | 3.280 | 0.496 | -0.362 | -0.333 | 1.174 |
| nsp14 | aa295-309 | EYPIIGDELKINAAC | -0.171 | -0.266 | 0.430 | -0.077 | 3.056 | 0.411 | -0.097 | 1.200 | 0.366 | 1.727 | 0.001 | 0.497 |
| nsp14 | aa337-351 | AIKCVPQADVEWKFY | 0.144 | 0.393 | 0.256 | -0.050 | -0.392 | 1.110 | 0.684 | 3.140 | 0.703 | -0.120 | 2.886 | 0.763 |
| nsp14 | aa353-367 | AQPCSDKAYKIEELF | 0.366 | 0.965 | -0.282 | -0.187 | -0.009 | 1.069 | -0.092 | 3.200 | 0.533 | 0.198 | 2.946 | 1.663 |
| nsp14 | aa355-369 | PCSDKAYKIEELFYS | -0.011 | 0.887 | 0.419 | 0.161 | 0.066 | 1.058 | 0.916 | 3.090 | 0.905 | 0.872 | 2.045 | 1.322 |
| nsp14 | aa363-377 | IEELFYSYATHSDKF | 0.238 | 0.499 | -0.408 | -0.723 | -0.481 | -0.355 | 0.029 | 3.070 | -0.275 | -0.093 | 2.020 | 1.812 |
| nsp14 | aa365-379 | ELFYSYATHSDKFTD | -0.644 | -0.642 | 3.100 | -0.742 | -0.502 | -0.435 | -0.532 | 0.195 | -0.020 | -0.714 | 0.725 | -0.352 |
| nsp14 | aa371-385 | ATHSDKFTDGVCLFW | 0.287 | 0.973 | 0.780 | 0.429 | -0.195 | 1.954 | 1.278 | 4.710 | 2.280 | 0.266 | 4.204 | 2.202 |
| nsp14 | aa373-387 | HSDKFTDGVCLFWNC | 0.139 | 0.145 | -0.033 | -0.093 | -0.318 | 0.449 | 0.241 | 4.230 | 1.060 | 1.548 | 2.803 | 1.808 |
| nsp14 | aa401-415 | FDTRVLSNLNLPGCD | -0.010 | -0.494 | 1.100 | -0.363 | -0.164 | 0.973 | -0.339 | 2.590 | 2.010 | 3.526 | -0.576 | 1.239 |
| nsp14 | aa405-419 | VLSNLNLPGCDGGSL | -0.495 | -0.341 | -0.064 | -0.224 | -0.292 | 1.531 | 0.589 | 3.050 | 2.170 | 1.362 | 1.342 | 0.382 |
| nsp14 | aa407-421 | SNLNLPGCDGGSLYV | -0.489 | 0.309 | 0.314 | 0.603 | -0.316 | 1.856 | 1.087 | 3.670 | 2.020 | 1.817 | 1.733 | 0.878 |
| nsp14 | aa431-445 | FDKSAFVNLKQLPFF | -0.726 | -0.502 | -0.332 | -0.717 | -0.786 | -0.716 | 0.901 | 3.490 | -0.097 | -0.413 | 3.435 | 2.333 |
| nsp14 | aa433-447 | KSAFVNLKQLPFFYY | -0.162 | 1.110 | -0.074 | -0.318 | -0.679 | -0.485 | 1.024 | 4.200 | 1.510 | 0.678 | 3.363 | 2.020 |
| nsp14 | aa435-449 | AFVNLKQLPFFYYSD | 0.053 | 5.291 | 0.394 | 0.063 | -0.272 | -0.370 | 1.144 | 3.930 | 2.020 | 1.644 | 2.082 | 0.326 |
| nsp14 | aa437-451 | VNLKQLPFFYYSDSP | -0.707 | 4.511 | -0.706 | -0.809 | -0.833 | -0.342 | -0.721 | 0.508 | -0.288 | -0.435 | 1.369 | -0.382 |
| nsp14 | aa439-453 | LKQLPFFYYSDSPCE | 0.346 | 4.185 | 1.150 | 0.336 | -0.368 | 1.175 | 0.869 | 2.810 | 1.440 | 2.961 | 2.312 | 1.988 |
| nsp14 | aa441-455 | QLPFFYYSDSPCESH | -0.345 | 4.306 | 0.927 | -0.238 | -0.855 | -0.588 | 0.874 | 2.340 | 0.714 | 0.142 | 2.570 | 2.130 |
| nsp14 | aa451-465 | PCESHGKQVVSDIDY | 0.843 | 0.578 | 0.809 | -0.233 | -0.270 | 0.106 | 0.951 | 3.890 | 1.890 | 0.945 | 1.783 | 2.612 |
| nsp14 | aa479-493 | LGGAVCRHHANEYRL | -0.833 | 0.244 | -0.684 | -0.787 | -0.770 | -0.154 | 0.402 | 3.080 | -0.290 | 0.193 | 3.015 | 1.880 |
| nsp14 | aa481-495 | GAVCRHHANEYRLYL | -0.813 | 0.452 | -0.011 | -0.742 | -0.666 | 0.069 | 0.935 | 3.620 | 0.883 | 0.344 | 3.830 | 1.705 |
| nsp14 | aa485-499 | RHHANEYRLYLDAYN | -0.556 | 0.617 | 0.192 | -0.802 | -0.836 | -0.438 | 0.064 | 3.780 | 0.468 | 0.652 | 4.550 | 2.648 |
| nsp14 | aa487-501 | HANEYRLYLDAYNMM | -0.138 | -0.562 | -0.159 | -0.365 | -0.544 | 1.581 | -0.666 | 3.780 | 0.179 | -0.489 | 2.528 | 3.520 |
| nsp14 | aa495-509 | LDAYNMMISAGFSLW | -0.006 | 0.713 | 0.202 | 0.025 | 0.064 | 2.734 | 0.703 | 3.730 | 2.490 | 0.303 | 3.583 | 3.122 |
| nsp14 | aa503-517 | SAGFSLWVYKQFDTY | -0.148 | 0.380 | -0.189 | -0.636 | -0.304 | 0.094 | 0.048 | 3.240 | 0.056 | -0.052 | 2.412 | 1.810 |
| nsp14 | aa505-519 | GFSLWVYKQFDTYNL | -0.166 | -0.124 | -0.612 | -0.630 | -0.807 | -0.678 | -0.049 | 3.550 | 0.291 | -0.195 | 2.142 | -0.039 |
| nsp14 | aa509-523 | WVYKQFDTYNLWNTF | -0.533 | -0.176 | -0.603 | -0.769 | -0.780 | 0.160 | 0.708 | 2.510 | 0.783 | -0.352 | 3.253 | 0.806 |
| nsp15 | aa15-29 | HFDGQQGEVPVSIIN | 0.127 | -0.050 | -0.540 | -0.437 | -0.844 | -0.416 | -0.335 | 3.100 | 0.310 | 1.078 | -0.011 | 0.266 |
| nsp15 | aa29-43 | NNTVYTKVDGVDVEL | -0.384 | 0.120 | -0.041 | 0.448 | -0.465 | 0.811 | 0.644 | 3.000 | 1.320 | -0.198 | -0.103 | 1.840 |
| nsp15 | aa31-45 | TVYTKVDGVDVELFE | -0.041 | 1.327 | 0.945 | 0.950 | 1.007 | 2.370 | 1.332 | 3.740 | 2.170 | 1.786 | 1.504 | 2.880 |
| nsp15 | aa45-59 | ENKTTLPVNVAFELW | 0.031 | 0.966 | 0.986 | 0.329 | 0.351 | 2.596 | 1.142 | 3.770 | 2.350 | 0.470 | 3.549 | 3.065 |
| nsp15 | aa75-89 | NLGVDIAANTVIWDY | 0.942 | 1.804 | 1.600 | 0.691 | 0.796 | 1.619 | 1.539 | 3.770 | 2.140 | 1.639 | 3.106 | 3.115 |
| nsp15 | aa111-125 | KPTETICAPLTVFFD | 0.416 | 1.207 | 1.170 | 0.079 | -0.240 | -0.124 | 1.534 | 3.500 | 1.420 | 1.318 | 2.166 | -0.352 |
| nsp15 | aa121-135 | TVFFDGRVDGQVDLF | 0.458 | 1.260 | 1.110 | -0.005 | -0.126 | 1.626 | 1.857 | 4.300 | 2.760 | 0.371 | 3.737 | 3.143 |
| nsp15 | aa123-137 | FFDGRVDGQVDLFRN | -0.439 | 0.574 | 0.072 | -0.300 | -0.749 | 1.183 | 0.822 | 3.480 | 2.120 | -0.049 | 3.537 | 1.918 |
| nsp15 | aa127-141 | RVDGQVDLFRNARNG | -0.748 | -0.737 | -0.209 | -0.662 | -0.679 | 0.181 | -0.725 | 3.170 | 0.567 | -0.827 | 0.396 | -0.064 |
| nsp15 | aa137-151 | NARNGVLITEGSVKG | -0.062 | -0.792 | -0.447 | -0.215 | -0.588 | 3.925 | -0.758 | 1.210 | -0.094 | -0.812 | -0.386 | 0.043 |
| nsp15 | aa201-215 | LQEFKPRSQMEIDFL | -0.260 | 0.378 | 0.264 | -0.603 | -0.770 | 0.124 | -0.166 | 2.960 | 0.543 | 3.030 | 1.053 | 1.796 |
| nsp15 | aa209-223 | QMEIDFLELAMDEFI | 0.838 | 2.149 | 1.300 | 0.813 | 0.366 | 2.061 | 1.752 | 3.740 | 2.100 | 1.961 | 2.185 | 3.222 |
| nsp15 | aa211-225 | EIDFLELAMDEFIER | 1.343 | 1.446 | 1.450 | 0.689 | 0.511 | 2.376 | 1.592 | 4.060 | 2.000 | 1.773 | 3.003 | 3.289 |
| nsp15 | aa213-227 | DFLELAMDEFIERYK | -0.158 | 0.348 | 0.577 | -0.282 | -0.637 | 2.062 | 0.149 | 3.420 | 0.990 | -0.302 | 1.995 | 0.049 |
| nsp15 | aa217-231 | LAMDEFIERYKLEGY | 0.341 | 0.849 | 1.120 | 0.081 | -0.192 | 1.670 | 0.935 | 3.810 | 2.010 | 1.405 | 2.791 | 2.310 |
| nsp15 | aa219-233 | MDEFIERYKLEGYAF | -0.230 | 1.072 | 0.920 | 0.046 | 0.006 | 1.322 | 1.401 | 3.920 | 1.980 | 0.584 | 3.316 | 2.644 |
| nsp15 | aa221-235 | EFIERYKLEGYAFEH | 0.165 | 0.416 | 1.250 | -0.391 | -0.595 | 0.258 | 1.148 | 3.880 | 1.320 | 1.020 | 2.826 | 2.857 |
| nsp15 | aa227-241 | KLEGYAFEHIVYGDF | 0.413 | 1.826 | 1.810 | 0.124 | 0.556 | 0.632 | 1.558 | 4.540 | 2.910 | 1.656 | 3.606 | 2.316 |
| nsp15 | aa229-243 | EGYAFEHIVYGDFSH | -0.299 | 0.349 | 1.220 | -0.596 | -0.679 | -0.080 | 1.477 | 3.340 | 1.660 | 1.564 | 3.823 | 1.432 |
| nsp15 | aa233-247 | FEHIVYGDFSHSQLG | -0.817 | -0.614 | -0.716 | -0.857 | -0.850 | -0.325 | -0.046 | 3.310 | 0.164 | 0.640 | 1.851 | 0.087 |
| nsp15 | aa235-249 | HIVYGDFSHSQLGGL | -0.657 | 0.024 | -0.285 | -0.816 | -0.767 | -0.184 | -0.109 | 3.750 | 1.640 | -0.266 | 2.545 | 0.196 |
| nsp15 | aa237-251 | VYGDFSHSQLGGLHL | -0.776 | 0.147 | -0.449 | -0.847 | -0.877 | -0.389 | 0.126 | 3.420 | 1.470 | -0.426 | 2.780 | 0.678 |
| nsp15 | aa255-269 | LAKRFKESPFELEDF | 1.167 | 1.488 | 1.530 | 0.958 | 0.958 | 2.027 | 1.814 | 3.590 | 2.160 | 1.843 | 3.249 | 2.377 |
| nsp15 | aa263-277 | PFELEDFIPMDSTVK | 2.146 | 1.760 | 0.526 | -0.042 | -0.034 | 4.157 | 0.744 | 2.450 | 0.616 | 0.250 | 2.391 | 1.050 |
| nsp15 | aa265-279 | ELEDFIPMDSTVKNY | 0.646 | 0.108 | 0.065 | -0.560 | -0.574 | 1.278 | -0.372 | 2.020 | 1.310 | -0.417 | 3.054 | 1.969 |
| nsp15 | aa287-301 | GSSKCVCSVIDLLLD | 0.439 | 1.082 | 0.496 | 0.828 | 0.142 | 0.206 | 0.971 | 4.070 | 1.820 | 0.886 | 0.925 | 1.908 |
| nsp15 | aa289-303 | SKCVCSVIDLLLDDF | 0.995 | 1.853 | 1.650 | 1.846 | 1.113 | 1.314 | 2.274 | 5.140 | 2.350 | 1.387 | 2.771 | 3.416 |
| nsp15 | aa291-305 | CVCSVIDLLLDDFVE | 1.562 | 1.983 | 1.960 | 2.080 | 1.316 | 2.661 | 2.308 | 5.220 | 2.960 | 2.030 | 2.342 | 3.486 |
| nsp15 | aa293-307 | CSVIDLLLDDFVEII | 2.314 | 3.294 | 2.660 | 2.608 | 1.978 | 2.747 | 2.443 | 5.690 | 3.880 | 2.783 | 3.302 | 3.827 |
| nsp15 | aa295-309 | VIDLLLDDFVEIIKS | 1.198 | 1.075 | -0.175 | 1.768 | -0.288 | 0.761 | 0.983 | 3.770 | 1.330 | 0.318 | 1.317 | 1.476 |
| nsp15 | aa301-315 | DDFVEIIKSQDLSVV | -0.299 | -0.673 | -0.477 | -0.687 | -0.528 | -0.090 | -0.493 | 3.090 | -0.017 | -0.711 | 0.335 | 1.003 |
| nsp15 | aa319-333 | VKVTIDYTEISFMLW | 0.735 | 1.476 | 1.020 | 0.829 | 0.175 | 2.265 | 1.175 | 4.100 | 2.560 | 1.212 | 3.412 | 2.664 |
| nsp15 | aa329-343 | SFMLWCKDGHVETFY | -0.101 | 1.140 | 0.918 | -0.616 | -0.486 | 1.056 | 1.424 | 3.790 | 1.750 | 0.809 | 3.445 | 1.521 |
| nsp16 | aa57-71 | TLTLAVPYNMRVIHF | -0.680 | -0.688 | 0.080 | -0.735 | -0.609 | 0.002 | -0.363 | 3.140 | 1.430 | -0.624 | 1.916 | 0.464 |
| nsp16 | aa85-99 | VLRQWLPTGTLLVDS | -0.466 | -0.491 | -0.638 | -0.789 | -0.690 | -0.076 | -0.695 | 3.130 | 0.020 | -0.696 | 0.063 | -0.397 |
| nsp16 | aa91-105 | PTGTLLVDSDLNDFV | 0.429 | 0.513 | 0.146 | 0.619 | -0.374 | -0.518 | 1.733 | 3.370 | 0.835 | 1.023 | 2.483 | 2.844 |
| nsp16 | aa119-133 | VHTANKWDLIISDMY | 0.093 | 0.822 | 0.802 | -0.111 | -0.568 | 0.851 | 0.748 | 3.460 | 1.890 | 0.435 | 2.861 | 1.610 |
| nsp16 | aa121-135 | TANKWDLIISDMYDP | 0.410 | 0.926 | 1.010 | -0.162 | -0.310 | 1.868 | 0.320 | 3.300 | 1.460 | 0.808 | 1.722 | 1.668 |
| nsp16 | aa131-145 | DMYDPKTKNVTKEND | -0.649 | -0.780 | -0.531 | -0.797 | -0.702 | -0.531 | -0.845 | 0.529 | -0.400 | -0.614 | -0.410 | 3.056 |
| nsp16 | aa173-187 | TEHSWNADLYKLMGH | -0.126 | 1.433 | -0.109 | -0.329 | -0.837 | 1.257 | 0.023 | 3.470 | 1.270 | -0.331 | 2.221 | 1.926 |
| nsp16 | aa175-189 | HSWNADLYKLMGHFA | -0.557 | -0.610 | -0.286 | -0.317 | -0.803 | 0.820 | -0.194 | 3.420 | 1.220 | 0.308 | 1.868 | 1.744 |
| nsp16 | aa177-191 | WNADLYKLMGHFAWW | 0.219 | 0.712 | 0.877 | 0.345 | 0.037 | 2.875 | 2.106 | 4.720 | 2.930 | 0.954 | 4.235 | 3.562 |
| nsp16 | aa209-223 | GCNYLGKPREQIDGY | 0.076 | 0.781 | 0.447 | -0.008 | -0.267 | 0.778 | 0.959 | 3.480 | 1.470 | 0.647 | 2.640 | 2.857 |
| nsp16 | aa215-229 | KPREQIDGYVMHANY | -0.403 | 0.378 | 0.077 | -0.793 | -0.817 | 0.461 | 0.335 | 3.170 | 1.890 | 0.011 | 2.802 | 2.316 |
| nsp16 | aa217-231 | REQIDGYVMHANYIF | -0.246 | 0.548 | 0.177 | -0.372 | -0.774 | -0.084 | 0.500 | 3.280 | 1.330 | 0.825 | 3.545 | 1.543 |
| nsp16 | aa219-233 | QIDGYVMHANYIFWR | -0.256 | 1.145 | 0.476 | -0.151 | -0.103 | 1.492 | 0.840 | 3.650 | 1.960 | 0.772 | 4.164 | 1.743 |
| nsp16 | aa237-251 | PIQLSSYSLFDMSKF | -0.517 | 0.919 | -0.128 | -0.214 | -0.226 | 0.860 | 1.229 | 3.220 | 1.350 | 0.139 | 2.058 | 0.991 |
| Spike Protein | aa5-19 | SGMFVFLVLLPLVSS | -0.719 | 1.519 | 0.064 | -0.791 | -0.760 | -0.237 | -0.846 | 0.826 | 1.240 | 0.920 | 3.142 | 1.514 |
| Spike Protein | aa47-61 | KVFRSSVLHSTQDLF | -0.742 | 0.460 | -0.684 | -0.747 | -0.822 | -0.689 | 0.330 | 3.060 | -0.372 | -0.739 | 1.530 | 1.436 |
| Spike Protein | aa51-65 | SSVLHSTQDLFLPFF | -0.593 | 1.001 | 0.761 | 0.033 | -0.717 | 0.455 | 2.468 | 3.870 | 1.870 | 1.043 | 4.114 | 2.058 |
| Spike Protein | aa57-71 | TQDLFLPFFSNVTWF | -0.140 | 1.085 | 0.531 | 0.162 | -0.419 | 1.368 | 1.539 | 3.250 | 2.390 | 0.519 | 3.714 | 1.588 |
| Spike Protein | aa75-89 | HVSGTNGTKRFDNPV | -0.589 | -0.711 | -0.674 | -0.817 | -0.852 | 0.178 | 1.022 | 3.320 | -0.381 | -0.137 | 0.391 | -0.293 |
| Spike Protein | aa83-97 | KRFDNPVLPFNDGVY | -0.077 | 1.509 | 0.805 | -0.191 | -0.566 | 1.125 | 1.352 | 3.520 | 1.110 | 0.863 | 3.371 | 1.912 |
| Spike Protein | aa85-99 | FDNPVLPFNDGVYFA | -0.616 | 0.556 | 0.218 | -0.272 | -0.628 | 0.234 | 0.987 | 3.170 | 0.430 | 0.059 | 2.184 | 1.038 |
| Spike Protein | aa125-139 | IVNNATNVVIKVCEF | -0.312 | 0.180 | 0.125 | 0.119 | -0.475 | -0.594 | 0.747 | 3.440 | 0.182 | 0.322 | 0.621 | 0.667 |
| Spike Protein | aa127-141 | NNATNVVIKVCEFQF | -0.575 | 0.834 | -0.135 | -0.635 | -0.593 | -0.026 | 0.428 | 3.430 | 0.665 | -0.244 | 2.296 | 1.120 |
| Spike Protein | aa133-147 | VIKVCEFQFCNDPFL | 0.061 | 1.545 | 1.170 | 0.133 | -0.458 | -0.043 | 0.819 | 4.130 | 1.970 | 1.476 | 3.617 | 0.794 |
| Spike Protein | aa137-151 | CEFQFCNDPFLGVYY | 1.793 | 2.662 | 2.590 | 1.320 | 0.885 | 1.288 | 2.782 | 5.320 | 4.120 | 2.752 | 4.616 | 3.656 |
| Spike Protein | aa149-163 | VYYHKNNKSWMESEF | 0.373 | 0.384 | 0.745 | 0.161 | -0.095 | 1.813 | 0.852 | 3.500 | 2.230 | 0.694 | 3.488 | 3.256 |
| Spike Protein | aa151-165 | YHKNNKSWMESEFRV | 0.174 | -0.530 | 0.016 | 0.152 | -0.417 | 1.696 | 0.399 | 3.150 | 1.390 | 0.172 | 2.645 | 2.265 |
| Spike Protein | aa153-167 | KNNKSWMESEFRVYS | 0.100 | -0.358 | 0.233 | -0.241 | -0.266 | 1.174 | -0.517 | 3.600 | 2.080 | -0.057 | 2.067 | 1.269 |
| Spike Protein | aa163-177 | FRVYSSANNCTFEYV | -0.101 | 1.465 | 0.556 | 0.983 | -0.231 | 0.076 | 1.870 | 3.420 | 1.300 | 1.133 | 1.879 | 1.137 |
| Spike Protein | aa171-185 | NCTFEYVSQPFLMDL | -0.078 | 0.513 | 0.065 | 0.499 | -0.389 | 1.088 | 1.154 | 3.010 | 0.510 | 0.441 | 1.008 | 2.165 |
| Spike Protein | aa177-191 | VSQPFLMDLEGKQGN | -0.574 | -0.567 | -0.639 | -0.710 | -0.839 | -0.453 | 0.168 | 5.230 | -0.282 | -0.593 | -0.292 | 0.846 |
| Spike Protein | aa193-207 | KNLREFVFKNIDGYF | 0.239 | 1.604 | 0.594 | 0.680 | -0.416 | 1.406 | 2.014 | 4.280 | 2.070 | 1.092 | 4.109 | 2.393 |
| Spike Protein | aa195-209 | LREFVFKNIDGYFKI | 0.428 | -0.142 | -0.480 | 0.413 | -0.674 | 0.604 | 0.925 | 3.570 | 1.240 | -0.483 | 2.502 | 0.932 |
| Spike Protein | aa251-265 | HRSYLTPGDSSSGWT | -0.337 | -0.081 | 0.017 | 0.023 | -0.395 | 2.142 | -0.033 | 3.900 | 2.070 | 0.192 | 1.937 | 0.752 |
| Spike Protein | aa261-275 | SSGWTAGAAAYYVGY | -0.406 | 0.430 | 0.114 | -0.677 | -0.445 | 0.587 | 0.319 | 3.200 | 0.475 | 0.401 | 1.520 | 0.956 |
| Spike Protein | aa335-349 | FPNITNLCPFGEVFN | -0.620 | 0.661 | 0.314 | -0.595 | -0.796 | -0.311 | 0.703 | 3.090 | 0.908 | 0.067 | 2.108 | 0.376 |
| Spike Protein | aa339-353 | TNLCPFGEVFNATRF | -0.556 | -0.060 | -0.112 | -0.572 | -0.780 | 0.335 | 1.234 | 3.160 | 1.030 | -0.084 | 3.554 | 0.711 |
| Spike Protein | aa345-359 | GEVFNATRFASVYAW | -0.358 | -0.042 | 0.079 | -0.424 | -0.286 | 1.715 | 0.552 | 2.840 | 1.290 | -0.114 | 3.171 | 2.139 |
| Spike Protein | aa361-375 | RKRISNCVADYSVLY | -0.279 | -0.058 | -0.287 | -0.598 | -0.453 | -0.127 | 0.187 | 3.250 | 0.870 | -0.299 | 2.951 | 0.816 |
| Spike Protein | aa393-407 | LNDLCFTNVYADSFV | -0.317 | 0.799 | 0.824 | -0.305 | -0.612 | 0.200 | 1.171 | 3.690 | 0.448 | 1.283 | 1.536 | 0.343 |
| Spike Protein | aa397-411 | CFTNVYADSFVIRGD | 0.236 | -0.297 | 0.007 | -0.489 | -0.512 | 0.457 | 0.343 | 1.480 | 4.500 | 0.070 | 0.491 | 0.005 |
| Spike Protein | aa415-429 | QIAPGQTGKIADYNY | -0.139 | 0.326 | 0.446 | -0.599 | -0.631 | -0.237 | 0.373 | 3.710 | 2.350 | 0.135 | 2.623 | 1.293 |
| Spike Protein | aa417-431 | APGQTGKIADYNYKL | -0.657 | -0.709 | -0.580 | -0.806 | -0.767 | -0.466 | -0.576 | 3.160 | 0.603 | -0.784 | 0.088 | -0.109 |
| Spike Protein | aa421-435 | TGKIADYNYKLPDDF | -0.429 | 0.512 | 0.281 | -0.318 | 0.057 | 0.376 | 0.739 | 3.520 | 0.687 | -0.147 | 2.145 | 2.404 |
| Spike Protein | aa441-455 | AWNSNNLDSKVGGNY | -0.661 | -0.399 | -0.724 | -0.382 | -0.676 | -0.194 | 1.057 | 3.230 | 0.428 | -0.580 | 1.894 | 1.403 |
| Spike Protein | aa443-457 | NSNNLDSKVGGNYNY | 0.016 | 0.660 | -0.215 | -0.329 | -0.715 | -0.106 | 2.875 | 3.870 | 0.980 | 0.019 | 2.580 | 2.307 |
| Spike Protein | aa445-459 | NNLDSKVGGNYNYLY | 0.031 | 1.043 | 0.340 | -0.169 | -0.278 | 0.171 | 1.071 | 3.970 | 1.740 | 0.623 | 3.252 | 2.248 |
| Spike Protein | aa459-473 | YRLFRKSNLKPFERD | -0.520 | -0.371 | -0.327 | -0.289 | -0.287 | 1.794 | -0.250 | 3.040 | 0.949 | -0.043 | 2.595 | 2.281 |
| Spike Protein | aa465-479 | SNLKPFERDISTEIY | 0.679 | 1.631 | 0.469 | 0.154 | 0.179 | 1.181 | 1.565 | 4.350 | 1.880 | 1.173 | 2.817 | 2.347 |
| Spike Protein | aa481-495 | AGSTPCNGVEGFNCY | 0.341 | 0.974 | 1.460 | 0.067 | -0.261 | 0.488 | 1.314 | 3.290 | 2.160 | 1.667 | 3.151 | 1.839 |
| Spike Protein | aa483-497 | STPCNGVEGFNCYFP | 0.023 | 0.107 | 0.588 | -0.359 | -0.689 | 0.105 | 0.742 | 2.870 | 0.896 | 0.782 | 3.374 | 0.663 |
| Spike Protein | aa487-501 | NGVEGFNCYFPLQSY | 0.481 | 0.462 | 0.409 | -0.468 | -0.548 | -0.255 | 0.680 | 3.440 | 1.300 | 0.730 | 2.547 | 2.124 |
| Spike Protein | aa501-515 | YGFQPTNGVGYQPYR | -0.303 | 0.252 | 0.267 | -0.555 | -0.372 | 1.132 | -0.009 | 2.540 | 1.390 | -0.037 | 3.396 | 2.118 |
| Spike Protein | aa509-523 | VGYQPYRVVVLSFEL | 0.158 | 1.144 | 1.150 | 0.258 | 0.129 | 0.815 | 1.411 | 3.480 | 1.420 | 1.213 | 1.082 | 1.101 |
| Spike Protein | aa511-525 | YQPYRVVVLSFELLH | -0.417 | 0.840 | 0.696 | -0.249 | -0.841 | 0.140 | 0.802 | 3.560 | 1.030 | 0.610 | 2.405 | 0.467 |
| Spike Protein | aa577-591 | DTTDAVRDPQTLEIL | 0.784 | 0.151 | -0.025 | -0.781 | 0.020 | -0.350 | 0.855 | 3.020 | 1.160 | -0.300 | 0.193 | 0.585 |
| Spike Protein | aa579-593 | TDAVRDPQTLEILDI | 1.165 | 2.021 | 1.850 | 0.772 | -0.230 | -0.376 | 1.585 | 3.530 | 2.290 | 0.370 | 1.465 | 1.659 |
| Spike Protein | aa653-667 | AGCLIGAEHVNNSYE | 0.149 | 0.294 | 0.285 | -0.578 | -0.759 | -0.454 | 1.010 | 3.170 | 2.350 | 1.237 | 0.077 | 1.335 |
| Spike Protein | aa655-669 | CLIGAEHVNNSYECD | 1.285 | 1.878 | 1.810 | 0.724 | 0.260 | 1.182 | 1.839 | 5.330 | 2.790 | 3.521 | 1.549 | 3.045 |
| Spike Protein | aa657-671 | IGAEHVNNSYECDIP | -0.089 | 0.442 | 0.751 | -0.436 | -0.540 | 0.043 | 0.722 | 3.520 | 1.900 | 1.071 | 1.185 | 2.792 |
| Spike Protein | aa661-675 | HVNNSYECDIPIGAG | -0.615 | -0.758 | -0.544 | -0.546 | -0.771 | 0.446 | -0.680 | 3.060 | 1.810 | -0.499 | 0.293 | 1.480 |
| Spike Protein | aa733-747 | LPVSMTKTSVDCTMY | -0.262 | -0.131 | -0.275 | -0.595 | -0.697 | 1.063 | -0.407 | 3.390 | 0.988 | -0.081 | 2.544 | 1.622 |
| Spike Protein | aa741-755 | SVDCTMYICGDSTEC | 1.269 | 0.719 | 1.250 | 0.879 | 0.448 | 1.359 | 1.222 | 3.030 | 1.400 | 2.628 | 1.527 | 2.064 |
| Spike Protein | aa781-795 | DKNTQEVFAQVKQIY | -0.697 | -0.346 | -0.625 | -0.808 | -0.789 | 0.157 | -0.764 | 2.000 | 0.307 | -0.701 | 4.070 | -0.026 |
| Spike Protein | aa791-805 | VKQIYKTPPIKDFGG | -0.501 | -0.805 | -0.785 | -0.289 | -0.577 | 0.757 | -0.777 | 3.500 | 0.586 | -0.679 | 1.812 | -0.359 |
| Spike Protein | aa805-819 | GFNFSQILPDPSKPS | -0.766 | -0.791 | -0.376 | -0.792 | -0.843 | 0.462 | 3.193 | 1.610 | 0.405 | -0.845 | 1.132 | 0.151 |
| Spike Protein | aa811-825 | ILPDPSKPSKRSFIE | -0.124 | -0.639 | 0.539 | -0.458 | -0.664 | 1.475 | 2.769 | 3.170 | 1.520 | 0.157 | 3.699 | 1.367 |
| Spike Protein | aa815-829 | PSKPSKRSFIEDLLF | -0.017 | 0.194 | 1.100 | 0.544 | -0.456 | 2.940 | 1.737 | 4.160 | 3.020 | 0.781 | 3.324 | 2.404 |
| Spike Protein | aa833-847 | TLADAGFIKQYGDCL | 0.050 | 0.674 | -0.082 | 0.228 | -0.102 | 1.705 | 0.880 | 3.180 | 1.880 | 4.093 | 2.074 | 1.285 |
| Spike Protein | aa835-849 | ADAGFIKQYGDCLGD | 0.222 | 0.697 | 0.449 | 0.103 | -0.017 | 1.423 | 1.432 | 3.450 | 2.430 | 1.492 | 1.565 | 0.980 |
| Spike Protein | aa843-857 | YGDCLGDIAARDLIC | 0.186 | 1.081 | 1.840 | 0.743 | 0.240 | 1.737 | 1.437 | 3.560 | 2.450 | 3.065 | 2.703 | 2.400 |
| Spike Protein | aa863-877 | GLTVLPPLLTDEMIA | 0.597 | 0.713 | 0.217 | -0.088 | -0.498 | 1.594 | 0.827 | 3.140 | 1.340 | -0.655 | 0.603 | 2.158 |
| Spike Protein | aa865-879 | TVLPPLLTDEMIAQY | -0.116 | 0.755 | 0.469 | -0.127 | -0.357 | 0.598 | 0.777 | 1.930 | 0.538 | -0.147 | 3.086 | 1.709 |
| Spike Protein | aa899-913 | ALQIPFAMQMAYRFN | -0.775 | -0.321 | -0.612 | -0.221 | -0.812 | 1.034 | -0.141 | 3.910 | 1.660 | -0.476 | 2.437 | 1.298 |
| Spike Protein | aa909-923 | AYRFNGIGVTQNVLY | -0.510 | 0.294 | -0.412 | -0.832 | -0.858 | -0.515 | -0.171 | 1.950 | 0.101 | 0.052 | 3.883 | 0.742 |
| Spike Protein | aa919-933 | QNVLYENQKLIANQF | -0.743 | 0.024 | -0.668 | -0.763 | -0.809 | -0.209 | -0.546 | 2.010 | 1.470 | -0.202 | 3.022 | 0.262 |
| Spike Protein | aa987-1001 | LSRLDKVEAEVQIDR | 0.105 | -0.134 | 0.496 | -0.299 | -0.442 | 0.420 | -0.636 | 3.080 | 2.070 | -0.691 | 2.301 | 1.809 |
| Spike Protein | aa989-1003 | RLDKVEAEVQIDRLI | 1.046 | 0.869 | 0.032 | -0.214 | -0.659 | 0.294 | -0.177 | 3.410 | 1.340 | -0.273 | 2.365 | 1.031 |
| Spike Protein | aa1087-1101 | ICHDGKAHFPREGVF | 0.076 | 0.950 | -0.354 | 0.070 | -0.758 | 1.717 | 1.464 | 4.020 | 2.400 | 0.330 | 3.701 | 2.603 |
| Spike Protein | aa1095-1109 | FPREGVFVSNGTHWF | -0.438 | 1.001 | -0.116 | -0.091 | -0.783 | 1.221 | 1.969 | 3.860 | 1.850 | 0.452 | 4.170 | 2.188 |
| Spike Protein | aa1103-1117 | SNGTHWFVTQRNFYE | -0.571 | 0.526 | 0.007 | -0.207 | -0.654 | 0.443 | 1.894 | 3.320 | 1.190 | 0.972 | 1.973 | 1.401 |
| Spike Protein | aa1107-1121 | HWFVTQRNFYEPQII | 0.650 | 0.659 | -0.487 | -0.761 | -0.856 | -0.389 | 0.961 | 3.050 | 0.151 | 0.203 | 1.737 | 0.042 |
| Spike Protein | aa1117-1131 | EPQIITTDNTFVSGN | -0.764 | -0.699 | -0.779 | -0.778 | -0.868 | -0.152 | -0.733 | 3.920 | -0.505 | -0.667 | -0.503 | -0.532 |
| Spike Protein | aa1119-1133 | QIITTDNTFVSGNCD | -0.504 | -0.111 | -0.161 | -0.548 | -0.698 | -0.370 | -0.395 | 2.580 | 0.370 | 3.316 | 0.170 | 0.571 |
| Spike Protein | aa1145-1159 | DPLQPELDSFKEELD | 1.517 | 1.377 | 0.558 | 0.513 | 0.233 | 1.660 | 1.063 | 4.250 | 2.240 | 1.387 | 2.111 | 2.210 |
| Spike Protein | aa1147-1161 | LQPELDSFKEELDKY | 1.573 | 1.783 | 0.160 | 0.618 | 0.167 | 2.034 | 1.686 | 3.830 | 2.340 | 1.179 | 2.944 | 1.890 |
| Spike Protein | aa1149-1163 | PELDSFKEELDKYFK | 3.307 | 1.496 | -0.417 | -0.142 | -0.695 | 2.014 | 1.503 | 3.440 | 0.702 | 0.842 | 2.667 | 0.254 |
| Spike Protein | aa1159-1173 | DKYFKNHTSPDVDLG | -0.719 | -0.236 | -0.677 | -0.781 | -0.759 | -0.436 | 0.143 | 3.010 | 1.820 | 0.211 | 1.818 | 1.359 |
| Spike Protein | aa1161-1175 | YFKNHTSPDVDLGDI | -0.410 | 0.602 | 0.023 | -0.742 | -0.649 | -0.460 | 0.186 | 3.640 | 2.090 | 0.161 | 0.889 | 1.595 |
| Spike Protein | aa1167-1181 | SPDVDLGDISGINAS | -0.546 | 2.204 | -0.807 | -0.777 | -0.154 | -0.451 | -0.593 | 2.580 | -0.164 | -0.508 | 3.195 | 0.338 |
| Spike Protein | aa1169-1183 | DVDLGDISGINASVV | -0.705 | 2.643 | -0.550 | -0.828 | -0.808 | -0.722 | -0.716 | 0.616 | -0.113 | -0.645 | 4.979 | 0.139 |
| Spike Protein | aa1175-1189 | ISGINASVVNIQKEI | -0.398 | -0.737 | -0.659 | -0.829 | -0.841 | -0.450 | 0.755 | 1.240 | 5.110 | -0.817 | -0.626 | -0.780 |
| Spike Protein | aa1177-1191 | GINASVVNIQKEIDR | -0.426 | -0.318 | -0.740 | -0.486 | -0.307 | 0.123 | -0.106 | 1.050 | 4.740 | -0.454 | 0.276 | 0.814 |
| Spike Protein | aa1199-1213 | LNESLIDLQELGKYE | 1.333 | 2.072 | 1.060 | 0.618 | 0.091 | 2.213 | 1.007 | 3.780 | 3.250 | 1.654 | 2.354 | 2.397 |
| Spike Protein | aa1201-1215 | ESLIDLQELGKYEQY | 1.182 | 1.870 | 1.330 | 0.408 | 0.168 | 2.203 | 1.689 | 4.130 | 3.160 | 1.352 | 3.473 | 2.546 |
| Spike Protein | aa1205-1219 | DLQELGKYEQYIKWP | 0.419 | 0.408 | 0.932 | 0.210 | -0.695 | 1.918 | 0.969 | 2.820 | 3.110 | 0.003 | 2.964 | 1.651 |
| Spike Protein | aa1207-1221 | QELGKYEQYIKWPWY | 0.991 | 1.205 | 1.290 | 0.501 | -0.156 | 2.404 | 1.986 | 4.190 | 3.410 | 1.320 | 3.884 | 2.581 |
| Spike Protein | aa1209-1223 | LGKYEQYIKWPWYIW | 0.382 | 0.227 | 0.481 | 0.102 | -0.092 | 2.529 | 1.240 | 3.430 | 2.950 | 0.271 | 3.219 | 2.470 |
| Spike Protein | aa1213-1227 | EQYIKWPWYIWLGFI | -0.592 | 0.665 | -0.122 | -0.227 | -0.385 | 2.184 | 0.493 | 3.490 | 1.240 | 0.681 | 2.309 | 0.365 |
| Spike Protein | aa1233-1247 | IVMVTIMLCCMTSCC | -0.774 | -0.672 | 0.802 | -0.706 | -0.643 | 1.417 | -0.104 | 1.700 | 0.615 | 3.084 | -0.003 | 1.403 |
| Spike Protein | aa1251-1265 | KGCCSCGSCCKFDED | 0.993 | 1.093 | 0.509 | 0.779 | 0.441 | 1.893 | 0.424 | 4.100 | 2.110 | 1.242 | 2.330 | 3.335 |
| Spike Protein | aa1253-1267 | CCSCGSCCKFDEDDS | 0.638 | 1.102 | 0.752 | 1.033 | 0.301 | 1.877 | 1.620 | 4.000 | 1.730 | 1.078 | 2.814 | 3.565 |
| Spike Protein | aa1255-1269 | SCGSCCKFDEDDSEP | 0.556 | 0.718 | 0.132 | 0.674 | -0.430 | 1.839 | 1.019 | 3.290 | 3.190 | 1.050 | 1.971 | 2.739 |
| Spike Protein | aa1259-1273 | CCKFDEDDSEPVLKG | 0.302 | -0.590 | -0.650 | -0.079 | -0.670 | 1.477 | 0.438 | 3.180 | 0.929 | -0.727 | -0.315 | 0.594 |
| GS Linker | aa13-27 | HYTGSGSGSGMDLFM | -0.308 | 0.040 | 0.269 | -0.002 | -0.412 | 2.071 | 0.012 | 3.440 | 2.530 | 0.569 | 3.641 | 2.959 |
| Orf3a Protein | aa97-111 | YSHLLLVAAGLEAPF | -0.321 | -0.127 | -0.510 | -0.694 | -0.762 | 0.776 | 0.122 | 2.530 | 1.230 | -0.474 | 3.477 | 2.083 |
| Orf3a Protein | aa99-113 | HLLLVAAGLEAPFLY | -0.296 | 1.300 | 0.656 | 0.111 | -0.455 | 0.233 | 1.487 | 3.870 | 1.660 | 0.645 | 4.369 | 2.102 |
| Orf3a Protein | aa101-115 | LLVAAGLEAPFLYLY | 0.265 | 1.804 | 1.180 | 0.704 | 0.006 | 0.453 | 1.582 | 3.770 | 2.900 | 1.149 | 3.822 | 2.275 |
| Orf3a Protein | aa107-121 | LEAPFLYLYALVYFL | -0.624 | 0.511 | -0.617 | -0.609 | -0.805 | 0.633 | -0.539 | 2.950 | -0.061 | 0.355 | 3.096 | 0.368 |
| Orf3a Protein | aa137-151 | WKCRSKNPLLYDANY | -0.401 | 1.293 | 0.163 | -0.807 | -0.762 | -0.481 | 0.809 | 3.020 | 1.110 | 0.401 | 3.126 | 0.546 |
| Orf3a Protein | aa139-153 | CRSKNPLLYDANYFL | -0.560 | 0.661 | 0.031 | -0.609 | -0.726 | -0.594 | 0.673 | 3.040 | 4.510 | -0.123 | 3.106 | 0.710 |
| Orf3a Protein | aa141-155 | SKNPLLYDANYFLCW | -0.087 | 1.426 | 1.010 | 0.380 | 0.026 | 1.530 | 1.609 | 4.260 | 3.270 | 0.994 | 4.390 | 2.319 |
| Orf3a Protein | aa147-161 | YDANYFLCWHTNCYD | 1.235 | 2.344 | 1.700 | 1.075 | 0.967 | 0.033 | 2.837 | 4.470 | 3.090 | 2.744 | 3.311 | 3.285 |
| Orf3a Protein | aa149-163 | ANYFLCWHTNCYDYC | 1.096 | 2.496 | 2.450 | 1.427 | 1.621 | 0.967 | 2.468 | 4.520 | 3.420 | 3.192 | 3.770 | 3.703 |
| Orf3a Protein | aa151-165 | YFLCWHTNCYDYCIP | 0.271 | 1.628 | 1.490 | 0.691 | -0.510 | -0.427 | 1.990 | 3.830 | 2.250 | 2.212 | 3.364 | 1.447 |
| Orf3a Protein | aa153-167 | LCWHTNCYDYCIPYN | -0.460 | 0.796 | 0.128 | -0.703 | -0.737 | -0.324 | 1.372 | 3.700 | 1.110 | 0.988 | 3.952 | 2.131 |
| Orf3a Protein | aa171-185 | SSIVITSGDGTTSPI | -0.672 | -0.793 | -0.811 | -0.771 | -0.473 | -0.274 | 0.268 | 1.520 | 2.600 | 3.425 | -0.323 | -0.438 |
| Orf3a Protein | aa185-199 | ISEHDYQIGGYTEKW | 0.570 | 0.985 | 0.074 | 0.719 | -0.074 | 2.455 | 1.227 | 4.300 | 3.640 | 0.327 | 3.528 | 2.713 |
| Orf3a Protein | aa187-201 | EHDYQIGGYTEKWES | -0.091 | 0.562 | 0.838 | 0.663 | -0.305 | 2.220 | 0.615 | 1.090 | 3.540 | -0.200 | 1.825 | 2.362 |
| Orf3a Protein | aa189-203 | DYQIGGYTEKWESGV | -0.205 | 0.319 | 0.156 | 0.795 | -0.605 | 1.209 | -0.090 | 0.986 | 5.490 | 0.166 | 1.515 | 1.170 |
| Orf3a Protein | aa191-205 | QIGGYTEKWESGVKD | -0.305 | -0.249 | -0.428 | -0.027 | -0.640 | 1.540 | -0.090 | 0.393 | 3.070 | -0.449 | 0.805 | 0.563 |
| Orf3a Protein | aa199-213 | WESGVKDCVVLHSYF | -0.617 | 1.182 | -0.103 | 0.529 | -0.709 | 0.130 | 1.555 | 3.800 | 1.850 | 0.183 | 3.247 | 2.430 |
| Orf3a Protein | aa203-217 | VKDCVVLHSYFTSDY | 0.433 | 1.801 | 0.547 | 0.439 | -0.263 | 0.313 | 1.239 | 4.530 | 1.780 | 0.873 | 1.818 | 2.865 |
| Orf3a Protein | aa205-219 | DCVVLHSYFTSDYYQ | 0.186 | 2.111 | 0.934 | 0.912 | -0.552 | 0.354 | 2.382 | 4.200 | 2.210 | 1.826 | 2.977 | 2.776 |
| Orf3a Protein | aa207-221 | VVLHSYFTSDYYQLY | 0.091 | 2.281 | 0.944 | 0.692 | -0.614 | 0.149 | 2.731 | 4.420 | 1.710 | 1.730 | 3.946 | 2.731 |
| Orf3a Protein | aa225-239 | LSTDTGVEHVTFFIY | -0.126 | 1.309 | 0.491 | 0.531 | -0.382 | -0.229 | 1.093 | 2.550 | 2.560 | 1.656 | 3.582 | 1.768 |
| Orf3a Protein | aa239-253 | YNKIVDEPEEHVQIH | -0.410 | -0.370 | -0.600 | -0.844 | -0.832 | -0.606 | -0.549 | 1.190 | 0.351 | -0.359 | 3.076 | 0.336 |
| Orf3a Protein | aa257-271 | GSSGVVNPVMEPIYD | 0.432 | 1.550 | 0.087 | -0.382 | -0.301 | 0.064 | 3.431 | 1.970 | 1.050 | 1.926 | 1.338 | 1.249 |
| Orf3a Protein | aa259-273 | SGVVNPVMEPIYDEP | 0.063 | 1.441 | -0.133 | -0.539 | -0.374 | 0.078 | 3.361 | 0.357 | 0.349 | 1.135 | 1.891 | 0.958 |
| Orf3a Protein | aa261-275 | VVNPVMEPIYDEPTT | 0.040 | 1.534 | -0.666 | -0.795 | -0.810 | -0.676 | 3.295 | 2.010 | 0.205 | 0.757 | 2.676 | -0.162 |
| Orf3a Protein | aa263-277 | NPVMEPIYDEPTTTT | 0.059 | 0.529 | -0.812 | -0.729 | -0.550 | 0.225 | 3.214 | -0.116 | -0.315 | -0.078 | 2.641 | 0.295 |
| Orf3a Protein | aa265-279 | VMEPIYDEPTTTTSV | -0.511 | 0.187 | -0.655 | -0.764 | -0.848 | -0.228 | 3.080 | 0.247 | -0.185 | -0.639 | 2.147 | 1.130 |
| Envelope Protein | aa9-23 | SFVSEETGTLIVNSV | -0.446 | -0.533 | -0.657 | -0.760 | -0.823 | -0.687 | -0.333 | 0.288 | 0.077 | 3.597 | -0.614 | -0.387 |
| Membrane Glycoprotein | aa1-15 | SGSGSGMADSNGTIT | -0.736 | -0.565 | -0.836 | -0.803 | -0.820 | -0.538 | -0.687 | 5.060 | -0.549 | -0.790 | 2.759 | 1.087 |
| Membrane Glycoprotein | aa3-17 | SGSGMADSNGTITVE | 0.245 | -0.649 | -0.861 | -0.733 | -0.731 | -0.478 | -0.793 | 5.210 | 0.392 | -0.618 | 3.126 | 1.546 |
| Membrane Glycoprotein | aa5-19 | SGMADSNGTITVEEL | 1.225 | 0.315 | -0.796 | -0.075 | -0.375 | -0.106 | -0.559 | 5.340 | 0.445 | -0.601 | 2.849 | 2.120 |
| Membrane Glycoprotein | aa7-21 | MADSNGTITVEELKK | -0.277 | -0.761 | 0.330 | -0.333 | -0.611 | 0.187 | -0.741 | 3.560 | 0.447 | -0.845 | 2.071 | 1.370 |
| Membrane Glycoprotein | aa9-23 | DSNGTITVEELKKLL | -0.067 | -0.438 | 2.390 | -0.410 | -0.715 | 0.149 | 0.296 | 4.270 | 1.140 | -0.817 | 0.498 | -0.136 |
| Membrane Glycoprotein | aa13-27 | TITVEELKKLLEQWN | -0.159 | 0.455 | 0.790 | -0.089 | -0.622 | 0.304 | 0.012 | 3.310 | 1.630 | 0.054 | 2.866 | 0.560 |
| Membrane Glycoprotein | aa23-37 | LEQWNLVIGFLFLTW | -0.559 | 0.413 | -0.198 | -0.519 | -0.439 | 0.740 | 0.780 | 3.100 | 1.150 | 1.184 | 2.497 | 1.827 |
| Membrane Glycoprotein | aa37-51 | WICLLQFAYANRNRF | -0.489 | -0.010 | -0.799 | -0.750 | -0.623 | -0.658 | 0.785 | 1.860 | -0.397 | -0.675 | 4.141 | 1.755 |
| Membrane Glycoprotein | aa39-53 | CLLQFAYANRNRFLY | -0.611 | 0.387 | -0.284 | -0.777 | -0.620 | -0.491 | 0.530 | 2.770 | 6.030 | -0.496 | 4.485 | 1.826 |
| Membrane Glycoprotein | aa95-109 | GLMWLSYFIASFRLF | -0.279 | -0.211 | -0.153 | -0.441 | -0.389 | 0.568 | -0.146 | 2.380 | 2.300 | -0.049 | 3.179 | 2.019 |
| Membrane Glycoprotein | aa131-145 | HGTILTRPLLESELV | -0.535 | 0.147 | -0.441 | -0.001 | -0.747 | 0.017 | -0.235 | 3.070 | 0.552 | -0.385 | 0.472 | 0.204 |
| Membrane Glycoprotein | aa171-185 | PKEITVATSRTLSYY | 0.005 | 1.599 | -0.277 | -0.723 | -0.051 | 0.900 | 0.127 | 2.050 | 3.130 | 0.124 | 1.742 | 1.172 |
| Membrane Glycoprotein | aa191-205 | QRVAGDSGFAAYSRY | -0.275 | 0.883 | 0.271 | -0.540 | -0.464 | 0.552 | 0.914 | 2.370 | 1.340 | 0.351 | 3.853 | 1.686 |
| Membrane Glycoprotein | aa195-209 | GDSGFAAYSRYRIGN | 0.144 | -0.005 | -0.285 | -0.578 | -0.758 | 0.426 | 0.097 | 3.020 | 1.750 | -0.208 | -0.215 | 0.903 |
| Membrane Glycoprotein | aa205-219 | YRIGNYKLNTDHSSS | -0.199 | -0.722 | -0.673 | -0.819 | -0.819 | -0.401 | 3.152 | -0.517 | 0.167 | -0.657 | -0.026 | -0.573 |
| Membrane Glycoprotein | aa207-221 | IGNYKLNTDHSSSSD | -0.504 | -0.820 | -0.765 | -0.824 | -0.828 | -0.311 | 3.532 | 1.590 | -0.138 | -0.564 | -0.190 | -0.384 |
| Membrane Glycoprotein | aa209-223 | NYKLNTDHSSSSDNI | -0.333 | -0.511 | -0.664 | -0.280 | -0.829 | -0.632 | 3.576 | 1.410 | 0.575 | -0.278 | -0.003 | -0.111 |
| Orf6 Protein | aa7-21 | GMFHLVDFQVTIAEI | 0.709 | 1.218 | 0.138 | 0.395 | -0.702 | 0.153 | 0.707 | 3.030 | 2.340 | 0.198 | -0.133 | 0.454 |
| Orf6 Protein | aa9-23 | FHLVDFQVTIAEILL | -0.155 | 1.755 | 0.662 | 1.225 | -0.757 | 0.044 | 1.183 | 3.690 | 1.710 | 1.108 | 1.054 | 0.187 |
| Orf6 Protein | aa11-25 | LVDFQVTIAEILLII | 0.519 | 1.295 | 0.928 | 0.681 | 0.000 | -0.103 | 1.569 | 3.220 | 1.030 | 1.343 | 0.135 | -0.348 |
| Orf6 Protein | aa25-39 | IMRTFKVSIWNLDYI | -0.408 | 1.181 | 0.251 | 0.773 | -0.642 | -0.098 | 0.841 | 3.670 | 2.410 | 0.815 | 2.791 | 1.760 |
| Orf6 Protein | aa55-69 | KYSQLDEEQPMEIDG | 0.126 | -0.484 | -0.425 | -0.075 | -0.370 | -0.121 | 1.401 | -0.040 | 3.590 | 0.023 | 1.435 | 1.446 |
| Orf7a Protein | aa11-25 | ILFLALITLATCELY | 0.863 | 2.012 | 1.350 | 0.433 | 0.782 | 0.856 | 1.533 | 4.020 | 1.350 | 1.679 | 4.787 | 2.182 |
| Orf7a Protein | aa13-27 | FLALITLATCELYHY | -0.005 | 1.911 | 1.010 | 0.131 | -0.188 | -0.024 | 1.403 | 4.270 | 1.650 | 1.919 | 5.490 | 2.119 |
| Orf7a Protein | aa15-29 | ALITLATCELYHYQE | 0.025 | 0.104 | 0.285 | -0.326 | -0.260 | -0.342 | 0.740 | 2.570 | 0.460 | 1.281 | 4.307 | 1.315 |
| Orf7a Protein | aa17-31 | ITLATCELYHYQECV | 0.427 | 1.026 | 1.390 | 0.031 | -0.220 | 0.530 | 1.201 | 3.440 | 1.010 | 2.044 | 4.206 | 1.899 |
| Orf7a Protein | aa19-33 | LATCELYHYQECVRG | 1.189 | -0.430 | -0.232 | -0.316 | -0.342 | 1.406 | -0.025 | 1.950 | 1.360 | 0.477 | 3.642 | 1.528 |
| Orf7a Protein | aa31-45 | VRGTTVLLKEPCSSG | -0.493 | -0.396 | -0.219 | -0.200 | -0.390 | 1.621 | -0.721 | 0.897 | -0.287 | -0.732 | 3.153 | 0.510 |
| Orf7a Protein | aa35-49 | TVLLKEPCSSGTYEG | 0.526 | 0.678 | 0.372 | -0.452 | -0.500 | 1.491 | 0.037 | 1.790 | 1.070 | 0.594 | 3.947 | 0.710 |
| Orf7a Protein | aa39-53 | KEPCSSGTYEGNSPF | 2.429 | -0.202 | -0.129 | -0.685 | -0.744 | 0.326 | 0.082 | 2.250 | 1.110 | -0.209 | 3.942 | 0.835 |
| Orf7a Protein | aa41-55 | PCSSGTYEGNSPFHP | -0.188 | -0.677 | -0.515 | -0.814 | -0.823 | -0.625 | -0.566 | 2.240 | -0.346 | -0.606 | 3.311 | 0.065 |
| Orf7a Protein | aa47-61 | YEGNSPFHPLADNKF | -0.278 | -0.405 | -0.448 | -0.667 | -0.772 | 0.344 | -0.629 | 3.200 | 0.207 | -0.609 | 3.505 | -0.079 |
| Orf7a Protein | aa71-85 | AFACPDGVKHVYQLR | -0.814 | -0.334 | -0.829 | -0.819 | -0.612 | -0.171 | -0.627 | 3.080 | 1.050 | -0.730 | 1.825 | 1.167 |
| Orf7a Protein | aa73-87 | ACPDGVKHVYQLRAR | -0.776 | -0.779 | -0.828 | -0.295 | -0.145 | 0.831 | -0.485 | 3.110 | 1.300 | -0.748 | 1.727 | 1.346 |
| Orf8 Protein | aa33-47 | TQHQPYVVDDPCPIH | -0.444 | 0.337 | 0.109 | -0.665 | -0.540 | 0.038 | 0.210 | 3.070 | 0.363 | -0.108 | 3.775 | 1.792 |
| Orf8 Protein | aa35-49 | HQPYVVDDPCPIHFY | -0.152 | 1.458 | 1.440 | -0.319 | -0.629 | -0.154 | 1.924 | 4.540 | 1.850 | 1.369 | 4.705 | 2.554 |
| Orf8 Protein | aa39-53 | VVDDPCPIHFYSKWY | 0.469 | 1.053 | 1.080 | -0.091 | -0.550 | 1.005 | 1.232 | 3.680 | 2.230 | 0.693 | 3.628 | 1.916 |
| Orf8 Protein | aa69-83 | VDEAGSKSPIQYIDI | -0.379 | 1.268 | -0.155 | -0.488 | -0.245 | -0.108 | 0.356 | 3.680 | 0.285 | 1.659 | 4.433 | 1.437 |
| Orf8 Protein | aa71-85 | EAGSKSPIQYIDIGN | -0.754 | -0.096 | -0.350 | -0.637 | -0.822 | -0.045 | -0.339 | 4.700 | 0.473 | 1.151 | 2.724 | 0.253 |
| Orf8 Protein | aa75-89 | KSPIQYIDIGNYTVS | -0.852 | -0.460 | -0.859 | -0.536 | -0.846 | -0.515 | -0.081 | 2.300 | -0.619 | 1.104 | 3.123 | -0.380 |
| Orf8 Protein | aa77-91 | PIQYIDIGNYTVSCL | -0.685 | 1.567 | -0.296 | 0.031 | -0.798 | -0.289 | 1.222 | 2.790 | 0.333 | 2.505 | 3.256 | 0.151 |
| Orf8 Protein | aa101-115 | KLGSLVVRCSFYEDF | 0.200 | 1.552 | 1.310 | 1.238 | 1.104 | 0.501 | 2.288 | 4.510 | 2.570 | 0.659 | 3.835 | 3.371 |
| Orf8 Protein | aa103-117 | GSLVVRCSFYEDFLE | 1.424 | 2.713 | 1.880 | 1.626 | 1.267 | 1.737 | 1.852 | 4.370 | 3.440 | 2.374 | 3.143 | 3.636 |
| Orf8 Protein | aa105-119 | LVVRCSFYEDFLEYH | 1.665 | 2.803 | 2.140 | 2.041 | 0.601 | 1.115 | 2.489 | 5.440 | 4.310 | 2.426 | 4.450 | 4.110 |
| Orf8 Protein | aa107-121 | VRCSFYEDFLEYHDV | 1.628 | 2.379 | 2.110 | 1.605 | 0.126 | 0.449 | 1.844 | 5.260 | 4.270 | 2.364 | 3.766 | 3.217 |
| Orf8 Protein | aa109-123 | CSFYEDFLEYHDVRV | 0.306 | 1.479 | 0.680 | 0.294 | -0.478 | 0.720 | 1.619 | 4.350 | 3.000 | 1.355 | 3.720 | 2.913 |
| Orf8 Protein | aa111-125 | FYEDFLEYHDVRVVL | 0.210 | 1.715 | 1.040 | 1.038 | -0.042 | 1.163 | 1.448 | 4.620 | 3.060 | 0.964 | 4.032 | 3.059 |
| Orf8 Protein | aa113-127 | EDFLEYHDVRVVLDF | 1.222 | 1.945 | 1.510 | 1.854 | 0.852 | 1.195 | 1.232 | 3.900 | 2.760 | 1.405 | 3.044 | 2.460 |
| Nucleocapsid Phosphoprotein | aa9-23 | SDNGPQNQRNAPRIT | -0.308 | -0.498 | -0.869 | -0.851 | -0.845 | 0.062 | -0.818 | -0.700 | 4.200 | -0.395 | 0.972 | 0.934 |
| Nucleocapsid Phosphoprotein | aa13-27 | PQNQRNAPRITFGGP | -0.359 | -0.329 | -0.697 | -0.745 | -0.800 | 0.618 | -0.444 | 0.319 | 0.419 | 0.060 | 5.267 | -0.275 |
| Nucleocapsid Phosphoprotein | aa59-73 | WFTALTQHGKEDLKF | -0.459 | -0.205 | 3.040 | -0.683 | -0.679 | 0.540 | 0.118 | 2.850 | 1.520 | -0.713 | 1.851 | 0.503 |
| Nucleocapsid Phosphoprotein | aa79-93 | VPINTNSSPDDQIGY | -0.193 | 0.530 | 0.089 | -0.488 | -0.794 | -0.279 | 0.638 | 3.530 | 0.790 | 0.385 | 0.597 | 0.877 |
| Nucleocapsid Phosphoprotein | aa81-95 | INTNSSPDDQIGYYR | -0.345 | 0.404 | -0.125 | -0.414 | -0.610 | 1.408 | 0.856 | 3.290 | 1.080 | -0.260 | 2.547 | 0.929 |
| Nucleocapsid Phosphoprotein | aa101-115 | IRGGDGKMKDLSPRW | 0.124 | 0.039 | 0.116 | 0.320 | 0.332 | 2.650 | 0.257 | 3.550 | 2.900 | -0.090 | 4.090 | 3.106 |
| Nucleocapsid Phosphoprotein | aa103-117 | GGDGKMKDLSPRWYF | -0.031 | 0.689 | 0.106 | 0.045 | 0.136 | 2.160 | 0.537 | 4.110 | 2.820 | 0.423 | 4.395 | 3.026 |
| Nucleocapsid Phosphoprotein | aa105-119 | DGKMKDLSPRWYFYY | 0.055 | 0.389 | 0.316 | -0.296 | -0.046 | 1.248 | 0.266 | 4.000 | 2.590 | 0.127 | 4.347 | 2.396 |
| Nucleocapsid Phosphoprotein | aa125-139 | EAGLPYGANKDGIIW | 0.157 | -0.117 | 0.357 | 0.008 | -0.189 | 1.571 | 0.978 | 2.730 | 2.260 | 0.051 | 3.086 | 2.202 |
| Nucleocapsid Phosphoprotein | aa135-149 | DGIIWVATEGALNTP | -0.829 | -0.400 | -0.718 | -0.817 | -0.862 | -0.081 | -0.784 | 0.081 | -0.636 | -0.692 | 3.346 | -0.523 |
| Nucleocapsid Phosphoprotein | aa163-177 | AIVLQLPQGTTLPKG | -0.827 | -0.644 | -0.728 | -0.805 | -0.777 | 0.899 | 5.451 | 0.274 | 2.020 | -0.789 | 3.309 | 0.299 |
| Nucleocapsid Phosphoprotein | aa165-179 | VLQLPQGTTLPKGFY | -0.782 | -0.485 | -0.674 | -0.746 | -0.688 | 1.298 | 5.469 | 1.290 | 3.160 | -0.127 | 4.987 | 0.731 |
| Nucleocapsid Phosphoprotein | aa167-181 | QLPQGTTLPKGFYAE | -0.426 | 0.136 | -0.544 | -0.391 | -0.539 | 1.569 | 5.510 | 0.359 | 3.070 | -0.117 | 4.071 | 0.628 |
| Nucleocapsid Phosphoprotein | aa169-183 | PQGTTLPKGFYAEGS | -0.348 | -0.814 | -0.540 | -0.581 | -0.517 | 1.476 | 5.192 | 0.776 | 3.530 | -0.255 | 3.276 | 0.132 |
| Nucleocapsid Phosphoprotein | aa207-221 | GSSRGTSPARMAGNG | -0.643 | -0.795 | -0.670 | -0.632 | -0.556 | 0.944 | -0.650 | 4.980 | 1.080 | -0.039 | 1.031 | 0.006 |
| Nucleocapsid Phosphoprotein | aa209-223 | SRGTSPARMAGNGGD | -0.711 | -0.563 | -0.348 | -0.224 | -0.640 | 1.710 | 0.073 | 6.140 | 1.170 | 1.141 | -0.075 | -0.110 |
| Nucleocapsid Phosphoprotein | aa211-225 | GTSPARMAGNGGDAA | -0.845 | -0.665 | -0.798 | -0.794 | -0.867 | 0.883 | -0.682 | 4.080 | -0.494 | -0.362 | -0.346 | -0.239 |
| Nucleocapsid Phosphoprotein | aa213-227 | SPARMAGNGGDAALA | -0.808 | -0.320 | -0.678 | -0.828 | -0.150 | 0.926 | -0.693 | 5.000 | 0.130 | -0.293 | -0.545 | -0.141 |
| Nucleocapsid Phosphoprotein | aa215-229 | ARMAGNGGDAALALL | -0.795 | -0.653 | -0.687 | -0.824 | -0.788 | 0.640 | -0.749 | 4.910 | -0.060 | -0.418 | 0.237 | -0.176 |
| Nucleocapsid Phosphoprotein | aa217-231 | MAGNGGDAALALLLL | -0.315 | -0.115 | -0.623 | -0.845 | -0.825 | 0.621 | -0.490 | 3.710 | 0.611 | -0.135 | 0.152 | 1.533 |
| Nucleocapsid Phosphoprotein | aa219-233 | GNGGDAALALLLLDR | -0.298 | 0.977 | 0.068 | 0.275 | -0.339 | 1.618 | 1.180 | 4.480 | 1.640 | 1.256 | 2.525 | 2.316 |
| Nucleocapsid Phosphoprotein | aa243-257 | GKGQQQQGQTVTKKS | -0.779 | -0.812 | -0.857 | -0.749 | -0.619 | 0.101 | -0.628 | 3.760 | -0.199 | -0.733 | -0.254 | 1.209 |
| Nucleocapsid Phosphoprotein | aa245-259 | GQQQQGQTVTKKSAA | -0.817 | -0.815 | -0.782 | -0.776 | -0.830 | -0.147 | -0.333 | 3.410 | -0.089 | -0.798 | 1.208 | -0.211 |
| Nucleocapsid Phosphoprotein | aa273-287 | KAYNVTQAFGRRGPE | -0.078 | -0.385 | -0.201 | -0.335 | -0.152 | 1.216 | -0.670 | -0.136 | 1.940 | 0.332 | 3.691 | 1.205 |
| Nucleocapsid Phosphoprotein | aa285-299 | GPEQTQGNFGDQELI | -0.312 | 1.031 | 0.951 | -0.244 | -0.698 | 0.371 | 0.696 | 3.430 | 2.280 | 0.839 | 1.894 | 1.603 |
| Nucleocapsid Phosphoprotein | aa291-305 | GNFGDQELIRQGTDY | 0.242 | 1.089 | 1.140 | -0.432 | -0.438 | 1.262 | 0.930 | 3.770 | 2.200 | 1.143 | 2.961 | 1.766 |
| Nucleocapsid Phosphoprotein | aa307-321 | HWPQIAQFAPSASAF | -0.818 | -0.462 | -0.781 | -0.804 | -0.870 | -0.689 | -0.425 | 3.110 | -0.254 | -0.722 | 2.096 | 0.387 |
| Nucleocapsid Phosphoprotein | aa317-331 | SASAFFGMSRIGMEV | -0.102 | 0.299 | -0.273 | -0.542 | -0.537 | 1.923 | -0.290 | 1.680 | 6.230 | 0.430 | 1.824 | 1.882 |
| Nucleocapsid Phosphoprotein | aa323-337 | GMSRIGMEVTPSGTW | 0.108 | 0.805 | 0.536 | 0.051 | 0.080 | 2.505 | 0.624 | 3.230 | 2.300 | 0.401 | 2.667 | 2.921 |
| Nucleocapsid Phosphoprotein | aa397-411 | QTVTLLPAADLDDFS | 0.273 | 1.077 | 1.070 | -0.184 | -0.433 | 0.522 | 1.311 | 3.080 | 1.060 | 0.607 | 1.520 | 0.580 |
| Nucleocapsid Phosphoprotein | aa399-413 | VTLLPAADLDDFSKQ | -0.337 | 1.689 | 0.114 | -0.571 | -0.742 | 0.674 | 1.305 | 3.140 | 0.220 | -0.504 | 1.467 | 1.897 |
