# Supplementary material for: Humoral Immune Response Profile of COVID-19 Reveals Severity and Variant-Specific Epitopes: Lessons from SARS-CoV-2 Peptide Microarray
Source: Viruses. 2023 Jan 15;15(1):248. doi: 10.3390/v15010248 (PMC9866125; doi:10.3390/v15010248)
Supplement: Supplementary file 1 [file viruses-15-00248-s001.zip › Table S4.docx]

Table S4. Epitopes identified based on IgA response against SARS-CoV-2 proteins

| Protein | Immunogenic Epitope | No. of Peptides |
| --- | --- | --- |
| nsp1 | IPVAYRKVLLRKNGN | 1 |
| nsp1 | LKSFDLGDELGTDPYEDFQ | 3 |
| nsp2 | YTRYVDNNFCGPDGY | 1 |
| nsp2 | NIVGDFKLNEEIAII | 1 |
| nsp2 | EEKFKEGVEFLRDGWEI | 2 |
| nsp3 | DTVIEVQGYKSVNIT | 1 |
| nsp3 | VSELLTPLGIDLDEW | 1 |
| nsp3 | PLGIDLDEWSMATYYLFDE | 3 |
| nsp3 | WSMATYYLFDESGEF | 1 |
| nsp3 | CSFYPPDEDEEEGDCEEEEFE | 4 |
| nsp3 | GDCEEEEFEPSTQYEYGTE | 3 |
| nsp3 | SAALQPEEEQEEDWLDDDS | 3 |
| nsp3 | RTNVYLAVFDKNLYD | 1 |
| nsp3 | TLEETKFLTENLLLYID | 2 |
| nsp3 | FLTENLLLYIDINGN | 1 |
| nsp3 | KIQEGVVDYGARFYF | 1 |
| nsp3 | VLPNDDTLRVEAFEYYH | 2 |
| nsp3 | FEYYHTTDPSFLGRY | 1 |
| nsp3 | IELKFNPPALQDAYY | 1 |
| nsp3 | AGEAANFCALILAYC | 1 |
| nsp3 | LKHGTFTCASEYTGN | 1 |
| nsp3 | PITDVFYKENSYTTT | 1 |
| nsp3 | GVVCTEIDPKLDNYY | 1 |
| nsp3 | TFFPDLNGDVVAIDY | 1 |
| nsp3 | ITEEVGHTDLMAAYV | 1 |
| nsp3 | FKWDLTAFGLVAEWFLAYILF | 4 |
| nsp3 | VLGLAAIMQLFFSYF | 1 |
| nsp3 | SYFAVHFISNSWLMWLI | 2 |
| nsp3 | SAMVRMYIFFASFYYVW | 2 |
| nsp3 | PINPTDQSSYIVDSV | 1 |
| nsp4 | DTCFANKHADFDTWF | 1 |
| nsp4 | FATSACVLAAECTIF | 1 |
| nsp4 | EGSVRVVTTFDSEYC | 1 |
| nsp4 | PGVYSVIYLYLTFYL | 1 |
| nsp4 | IAYIICISTKHFYWF | 1 |
| nsp4 | VSFSTFEEAALCTFL | 1 |
| nsp5 | GTTTLNGLWLDDVVYCP | 2 |
| nsp5 | LNGSCGSVGFNIDYDCVSFCY | 4 |
| nsp5 | LAWLYAAVINGDRWF | 1 |
| nsp5 | NGRTILGSALLEDEF | 1 |
| nsp5 | ILGSALLEDEFTPFD | 1 |
| nsp6 | LVQSTQWSLFFFLYE | 1 |
| nsp6 | MFLARGIVFMCVEYC | 1 |
| nsp6 | RGIVFMCVEYCPIFF | 1 |
| nsp6 | MLVYCFLGYFCTCYF | 1 |
| nsp6 | CFLGYFCTCYFGLFC | 1 |
| nsp6 | CLLNRYFRLTLGVYDYL | 2 |
| nsp6 | LGVYDYLVSTQEFRY | 1 |
| nsp8 | CDGTTFTYASALWEI | 1 |
| nsp9 | TTQTACTDDNALAYY | 1 |
| nsp9 | VLGSLAATVRLQAGN | 1 |
| nsp10 | MDQESFGGASCCLYC | 1 |
| nsp12 | TGTSTDVVYRAFDIYND | 2 |
| nsp12 | FQEKDEDDNLIDSYFVV | 2 |
| nsp12 | MADLVYALRHFDEGN | 1 |
| nsp12 | DTLKEILVTYNCCDDDYFN | 3 |
| nsp12 | TYNCCDDDYFNKKDWYDFVEN | 4 |
| nsp12 | VLTLDNQDLNGNWYDFGDF | 3 |
| nsp12 | QTTPGSGVPVVDSYYSL | 2 |
| nsp12 | PVVDSYYSLLMPILT | 1 |
| nsp12 | VPFVVSTGYHFRELG | 1 |
| nsp12 | QTVKPGNFNKDFYDF | 1 |
| nsp12 | FFFAQDGNAAISDYDYYRY | 3 |
| nsp12 | LPTMCDIRQLLFVVEVV | 2 |
| nsp12 | IRQLLFVVEVVDKYFDCYD | 3 |
| nsp12 | EVVDKYFDCYDGGCI | 1 |
| nsp12 | LYYDSMSYEDQDALFAY | 2 |
| nsp12 | KYVRNLQHRLYECLY | 1 |
| nsp12 | YRNRDVDTDFVNEFYAY | 2 |
| nsp12 | HFSMMILSDDAVVCF | 1 |
| nsp12 | MLVKQGDDYVYLPYP | 1 |
| nsp12 | PYPDPSRILGAGCFV | 1 |
| nsp12 | PLTKHPNQEYADVFH | 1 |
| nsp12 | EYADVFHLYLQYIRK | 1 |
| nsp12 | MLTNDNTSRYWEPEFYEAMYT | 4 |
| nsp13 | ACIRRPFLCCKCCYD | 1 |
| nsp13 | TDVTQLYLGGMSYYC | 1 |
| nsp13 | FNAIATCDWTNAGDYIL | 2 |
| nsp13 | QIGEYTFEKGDYGDA | 1 |
| nsp13 | ALPETTADIVVFDEI | 1 |
| nsp13 | CPAEIVDTVSALVYD | 1 |
| nsp13 | TQTVDSSQGSEYDYVIF | 2 |
| nsp14 | PTGYVDTPNNTDFSR | 1 |
| nsp14 | GDQFKHLIPLMYKGL | 1 |
| nsp14 | SDTYACWHHSIGFDYVY | 2 |
| nsp14 | WGFTGNLQSNHDLYC | 1 |
| nsp14 | CFVKRVDWTIEYPII | 1 |
| nsp14 | EYPIIGDELKINAAC | 1 |
| nsp14 | PCSDKAYKIEELFYS | 1 |
| nsp14 | ELFYSYATHSDKFTD | 1 |
| nsp14 | ATHSDKFTDGVCLFW | 1 |
| nsp14 | AYNMMISAGFSLWVY | 1 |
| nsp15 | NLGVDIAANTVIWDY | 1 |
| nsp15 | TVFFDGRVDGQVDLF | 1 |
| nsp15 | QMEIDFLELAMDEFIER | 2 |
| nsp15 | MDEFIERYKLEGYAF | 1 |
| nsp15 | KLEGYAFEHIVYGDF | 1 |
| nsp15 | LAKRFKESPFELEDF | 1 |
| nsp15 | SKCVCSVIDLLLDDFVEII | 3 |
| nsp15 | VKVTIDYTEISFMLW | 1 |
| nsp16 | PTGTLLVDSDLNDFV | 1 |
| nsp16 | DCATVHTANKWDLIISDMYDPKT | 5 |
| nsp16 | ENDSKEGFFTYICGF | 1 |
| nsp16 | WNADLYKLMGHFAWW | 1 |
| nsp16 | PIQLSSYSLFDMSKF | 1 |
| Spike Protein | TQDLFLPFFSNVTWF | 1 |
| Spike Protein | CEFQFCNDPFLGVYY | 1 |
| Spike Protein | FRVYSSANNCTFEYV | 1 |
| Spike Protein | KNLREFVFKNIDGYF | 1 |
| Spike Protein | DSSSGWTAGAAAYYVGY | 2 |
| Spike Protein | NATRFASVYAWNRKR | 1 |
| Spike Protein | TDAVRDPQTLEILDI | 1 |
| Spike Protein | CLIGAEHVNNSYECD | 1 |
| Spike Protein | CASYQTQTNSPRRAR | 1 |
| Spike Protein | PLLTDEMIAQYTSAL | 1 |
| Spike Protein | DPLQPELDSFKEELD | 1 |
| Spike Protein | LNESLIDLQELGKYEQY | 2 |
| Spike Protein | QELGKYEQYIKWPWYIW | 2 |
| Spike Protein | CCSCGSCCKFDEDDS | 1 |
| Orf3a Protein | HLLLVAAGLEAPFLYLY | 2 |
| Orf3a Protein | AGLEAPFLYLYALVY | 1 |
| Orf3a Protein | SKNPLLYDANYFLCW | 1 |
| Orf3a Protein | YDANYFLCWHTNCYDYCIP | 3 |
| Orf3a Protein | DCVVLHSYFTSDYYQLY | 2 |
| Membrane Glycoprotein | LEQWNLVIGFLFLTW | 1 |
| Orf6 Protein | LVDFQVTIAEILLII | 1 |
| Orf6 Protein | IMRTFKVSIWNLDYI | 1 |
| Orf7a Protein | ILFLALITLATCELYHY | 2 |
| Orf7a Protein | QEEVQELYSPIFLIV | 1 |
| Orf8 Protein | PIQYIDIGNYTVSCLPF | 2 |
| Orf8 Protein | KLGSLVVRCSFYEDFLEYHDV | 4 |
| Orf8 Protein | FYEDFLEYHDVRVVLDF | 2 |
| Nucleocapsid Phosphoprotein | WFTALTQHGKEDLKF | 1 |
| Nucleocapsid Phosphoprotein | DGKMKDLSPRWYFYY | 1 |
| Nucleocapsid Phosphoprotein | SRGTSPARMAGNGGD | 1 |
| Nucleocapsid Phosphoprotein | TYTGAIKLDDKDPNF | 1 |
| Nucleocapsid Phosphoprotein | TFPPTEPKKDKKKKA | 1 |
