# Supplementary material for: Humoral Immune Response Profile of COVID-19 Reveals Severity and Variant-Specific Epitopes: Lessons from SARS-CoV-2 Peptide Microarray
Source: Viruses. 2023 Jan 15;15(1):248. doi: 10.3390/v15010248 (PMC9866125; doi:10.3390/v15010248)
Supplement: Supplementary file 1 [file viruses-15-00248-s001.zip › Table S5.docx]

Table S5. Epitopes identified based on IgG response against SARS-CoV-2 proteins

| Protein | Immunogenic Epitope | No. of Peptides |
| --- | --- | --- |
| nsp1 | DGTCGLVEVEKGVLPQL | 2 |
| nsp1 | VEVEKGVLPQLEQPYVF | 2 |
| nsp1 | GEIPVAYRKVLLRKNGN | 2 |
| nsp1 | GAGGHSYGADLKSFD | 1 |
| nsp1 | HSYGADLKSFDLGDELG | 2 |
| nsp1 | LKSFDLGDELGTDPYEDFQENWN | 5 |
| nsp2 | ELMRELNGGAYTRYV | 1 |
| nsp2 | ELNGGAYTRYVDNNF | 1 |
| nsp2 | GAYTRYVDNNFCGPDGYPLEC | 4 |
| nsp2 | RGVYCCREHEHEIAWYTER | 3 |
| nsp2 | HEIAWYTERSEKSYE | 1 |
| nsp2 | EIKLAKKFDTFNGEC | 1 |
| nsp2 | KCDHCGETSWQTGDF | 1 |
| nsp2 | HNSEVGPEHSLAEYH | 1 |
| nsp2 | PEHSLAEYHNESGLK | 1 |
| nsp2 | RTIAFGGCVFSYVGC | 1 |
| nsp2 | NIVGDFKLNEEIAII | 1 |
| nsp2 | ASTSAFVETVKGLDY | 1 |
| nsp2 | LDYKAFKQIVESCGN | 1 |
| nsp2 | IFGTVYEKLKPVLDW | 1 |
| nsp2 | LDWLEEKFKEGVEFLRDGWEIVKFI | 6 |
| nsp2 | EIIFLEGETLPTEVLTE | 2 |
| nsp2 | GETLPTEVLTEEVVL | 1 |
| nsp3 | KVTFGDDTVIEVQGY | 1 |
| nsp3 | YTVELGTEVNEFACV | 1 |
| nsp3 | QPVSELLTPLGIDLDEWSMATYYLFDESGEFKL | 10 |
| nsp3 | FDESGEFKLASHMYC | 1 |
| nsp3 | GEFKLASHMYCSFYP | 1 |
| nsp3 | MYCSFYPPDEDEEEGDCEEEEFEPSTQYEYGTEDDYQ | 12 |
| nsp3 | EYGTEDDYQGKPLEF | 1 |
| nsp3 | SAALQPEEEQEEDWLDDDSQQ | 4 |
| nsp3 | NSFSGYLKLTDNVYI | 1 |
| nsp3 | NKATNNAMQVESDDY | 1 |
| nsp3 | NNAMQVESDDYIATN | 1 |
| nsp3 | KGEDIQLLKSAYENF | 1 |
| nsp3 | RTNVYLAVFDKNLYD | 1 |
| nsp3 | KQDDKKIKACVEEVT | 1 |
| nsp3 | TLEETKFLTENLLLYIDINGN | 4 |
| nsp3 | HPDSATLVSDIDITF | 1 |
| nsp3 | GIKIQEGVVDYGARFYFYT | 3 |
| nsp3 | LNDLNETLVTMPLGY | 1 |
| nsp3 | YVTHGLNLEEAARYM | 1 |
| nsp3 | HFIETISLAGSYKDWSY | 2 |
| nsp3 | WSYSGQSTQLGIEFL | 1 |
| nsp3 | QLGIEFLKRGDKSVY | 1 |
| nsp3 | NPTTFHLDGEVITFD | 1 |
| nsp3 | VDNINLHTQVVDMSM | 1 |
| nsp3 | HTQVVDMSMTYGQQF | 1 |
| nsp3 | FYVLPNDDTLRVEAFEYYH | 3 |
| nsp3 | TLRVEAFEYYHTTDPSFLGRY | 4 |
| nsp3 | HTTDPSFLGRYMSAL | 1 |
| nsp3 | SALNHTKKWKYPQVN | 1 |
| nsp3 | NGLTSIKWADNNCYL | 1 |
| nsp3 | IELKFNPPALQDAYY | 1 |
| nsp3 | AGEAANFCALILAYC | 1 |
| nsp3 | VGELGDVRETMSYLF | 1 |
| nsp3 | TTLKGVEAVMYMGTL | 1 |
| nsp3 | VMYMGTLSYEQFKKG | 1 |
| nsp3 | LKHGTFTCASEYTGN | 1 |
| nsp3 | TCASEYTGNYQCGHY | 1 |
| nsp3 | HITSKETLYCIDGAL | 1 |
| nsp3 | TKSSEYKGPITDVFY | 1 |
| nsp3 | GVVCTEIDPKLDNYY | 1 |
| nsp3 | TEIDPKLDNYYKKDNSY | 2 |
| nsp3 | YFTEQPIDLVPNQPY | 1 |
| nsp3 | LVPNQPYPNASFDNFKF | 2 |
| nsp3 | YPNASFDNFKFVCDN | 1 |
| nsp3 | FKFVCDNIKFADDLN | 1 |
| nsp3 | PASRELKVTFFPDLN | 1 |
| nsp3 | TFFPDLNGDVVAIDY | 1 |
| nsp3 | SFKKGAKLLHKPIVW | 1 |
| nsp3 | KATYKPNTWCIRCLW | 1 |
| nsp3 | LECNVKTTEVVGDII | 1 |
| nsp3 | ITEEVGHTDLMAAYV | 1 |
| nsp3 | LATHGLAAVNSVPWD | 1 |
| nsp3 | TRCLNRVCTNYMPYF | 1 |
| nsp3 | ASMPTTIAKNTVKSV | 1 |
| nsp3 | KSVGKFCLEASFNYL | 1 |
| nsp3 | VLMSNLGMPSYCTGY | 1 |
| nsp3 | NLGMPSYCTGYREGYLN | 2 |
| nsp3 | CSVCLSGLDSLDTYP | 1 |
| nsp3 | FKWDLTAFGLVAEWF | 1 |
| nsp3 | AFGLVAEWFLAYILF | 1 |
| nsp3 | VLGLAAIMQLFFSYF | 1 |
| nsp3 | SYFAVHFISNSWLMWLI | 2 |
| nsp3 | SAMVRMYIFFASFYYVW | 2 |
| nsp3 | FYYVWKSYVHVVDGC | 1 |
| nsp3 | VYANGGKGFCKLHNW | 1 |
| nsp3 | KGFCKLHNWNCVNCDTF | 2 |
| nsp3 | FCAGSTFISDEVARD | 1 |
| nsp3 | DSVTVKNGSIHLYFD | 1 |
| nsp3 | HLYFDKAGQKTYERHSL | 2 |
| nsp3 | ERHSLSHFVNLDNLRAN | 2 |
| nsp3 | VGDSAEVAVKMFDAY | 1 |
| nsp3 | GFVDSDVETKDVVEC | 1 |
| nsp3 | EVTGDSCNNYMLTYN | 1 |
| nsp3 | VENMTPRDLGACIDC | 1 |
| nsp4 | NWLKQLIKVTLVFLF | 1 |
| nsp4 | QLIKVTLVFLFVAAIFY | 2 |
| nsp4 | SEIIGYKAIDGGVTR | 1 |
| nsp4 | DTCFANKHADFDTWF | 1 |
| nsp4 | PGTILRTTNGDFLHF | 1 |
| nsp4 | DFLHFLPRVFSAVGN | 1 |
| nsp4 | NICYTPSKLIEYTDF | 1 |
| nsp4 | SKLIEYTDFATSACV | 1 |
| nsp4 | FATSACVLAAECTIF | 1 |
| nsp4 | TIFKDASGKPVPYCY | 1 |
| nsp4 | EGSVAYESLRPDTRY | 1 |
| nsp4 | EGSVRVVTTFDSEYCRH | 2 |
| nsp4 | ERSEAGVCVSTSGRW | 1 |
| nsp4 | VCVSTSGRWVLNNDYYR | 2 |
| nsp4 | WVLNNDYYRSLPGVF | 1 |
| nsp4 | LAYYFMRFRRAFGEYSH | 2 |
| nsp4 | GEYSHVVAFNTLLFL | 1 |
| nsp4 | FYLTNDVSFLAHIQW | 1 |
| nsp4 | NDVSFLAHIQWMVMF | 1 |
| nsp4 | AHIQWMVMFTPLVPFWI | 2 |
| nsp4 | HFYWFFSNYLKRRVV | 1 |
| nsp4 | VSFSTFEEAALCTFLLN | 2 |
| nsp4 | EEAALCTFLLNKEMY | 1 |
| nsp4 | SGAMDTTSYREAACC | 1 |
| nsp4 | KALNDFSNSGSDVLY | 1 |
| nsp5 | QVTCGTTTLNGLWLDDVVYCPRH | 5 |
| nsp5 | HVICTSEDMLNPNYEDLLI | 3 |
| nsp5 | RPNFTIKGSFLNGSC | 1 |
| nsp5 | LNGSCGSVGFNIDYDCVSFCYMHHMEL | 7 |
| nsp5 | LPTGVHAGTDLEGNFYGPF | 3 |
| nsp5 | AAGTDTTITVNVLAWLY | 2 |
| nsp5 | LAWLYAAVINGDRWF | 1 |
| nsp5 | YAAVINGDRWFLNRF | 1 |
| nsp5 | DMCASLKELLQNGMN | 1 |
| nsp5 | NGRTILGSALLEDEF | 1 |
| nsp5 | ILGSALLEDEFTPFDVVRQ | 3 |
| nsp5 | PFDVVRQCSGVTFQS | 1 |
| nsp6 | SLLVLVQSTQWSLFF | 1 |
| nsp6 | LVQSTQWSLFFFLYE | 1 |
| nsp6 | WSLFFFLYENAFLPF | 1 |
| nsp6 | FAMGIIAMSAFAMMF | 1 |
| nsp6 | FNMVYMPASWVMRIMTW | 2 |
| nsp6 | AVVLLILMTARTVYD | 1 |
| nsp6 | LILMTARTVYDDGAR | 1 |
| nsp6 | RTVYDDGARRVWTLM | 1 |
| nsp6 | KVYYGNALDQAISMW | 1 |
| nsp6 | GNALDQAISMWALII | 1 |
| nsp6 | MFLARGIVFMCVEYC | 1 |
| nsp6 | RGIVFMCVEYCPIFF | 1 |
| nsp6 | FMCVEYCPIFFITGN | 1 |
| nsp6 | LQCIMLVYCFLGYFC | 1 |
| nsp6 | MLVYCFLGYFCTCYF | 1 |
| nsp6 | CFLGYFCTCYFGLFC | 1 |
| nsp6 | CTCYFGLFCLLNRYF | 1 |
| nsp6 | CLLNRYFRLTLGVYDYL | 2 |
| nsp6 | LTLGVYDYLVSTQEFRY | 2 |
| nsp7 | SVLQQLRVESSSKLW | 1 |
| nsp7 | KLWAQCVQLHNDILL | 1 |
| nsp8 | AFATAQEAYEQAVANGDSEVV | 4 |
| nsp8 | RAKVTSAMQTMLFTM | 1 |
| nsp8 | NDALNNIINNARDGC | 1 |
| nsp8 | NTCDGTTFTYASALWEI | 2 |
| nsp8 | SKIVQLSEISMDNSP | 1 |
| nsp8 | QLSEISMDNSPNLAW | 1 |
| nsp9 | TTQTACTDDNALAYY | 1 |
| nsp9 | LALLSDLQDLKWARF | 1 |
| nsp9 | VLGSLAATVRLQAGN | 1 |
| nsp9 | LAATVRLQAGNATEV | 1 |
| nsp10 | CAFAVDAAKAYKDYL | 1 |
| nsp10 | PEANMDQESFGGASC | 1 |
| nsp10 | CRCHIDHPNPKGFCD | 1 |
| nsp10 | HPNPKGFCDLKGKYV | 1 |
| nsp10 | CTVCGMWKGYGCSCD | 1 |
| nsp12 | RLTPCGTGTSTDVVY | 1 |
| nsp12 | TGTSTDVVYRAFDIYND | 2 |
| nsp12 | FLKTNCCRFQEKDED | 1 |
| nsp12 | FQEKDEDDNLIDSYFVV | 2 |
| nsp12 | HEETIYNLLKDCPAV | 1 |
| nsp12 | NLLKDCPAVAKHDFF | 1 |
| nsp12 | TKYTMADLVYALRHFDEGNCD | 4 |
| nsp12 | NCDTLKEILVTYNCCDDDYFN | 4 |
| nsp12 | TYNCCDDDYFNKKDWYDFVEN | 4 |
| nsp12 | VGVLTLDNQDLNGNWYD | 2 |
| nsp12 | DNQDLNGNWYDFGDFIQ | 2 |
| nsp12 | QTTPGSGVPVVDSYY | 1 |
| nsp12 | HVDTDLTKPYIKWDL | 1 |
| nsp12 | DLTKPYIKWDLLKYD | 1 |
| nsp12 | PYIKWDLLKYDFTEE | 1 |
| nsp12 | KYDFTEERLKLFDRYFK | 2 |
| nsp12 | LKLFDRYFKYWDQTY | 1 |
| nsp12 | QTYHPNCVNCLDDRCIL | 2 |
| nsp12 | CANFNVLFSTVFPPT | 1 |
| nsp12 | FRELGVVHNQDVNLH | 1 |
| nsp12 | NQDVNLHSSRLSFKE | 1 |
| nsp12 | NLHSSRLSFKELLVY | 1 |
| nsp12 | LVYAADPAMHAASGN | 1 |
| nsp12 | ALTNNVAFQTVKPGN | 1 |
| nsp12 | QTVKPGNFNKDFYDF | 1 |
| nsp12 | FFKEGSSVELKHFFF | 1 |
| nsp12 | FFFAQDGNAAISDYDYYRYNL | 4 |
| nsp12 | ISDYDYYRYNLPTMCDI | 2 |
| nsp12 | TMCDIRQLLFVVEVV | 1 |
| nsp12 | QLLFVVEVVDKYFDCYD | 2 |
| nsp12 | EVVDKYFDCYDGGCI | 1 |
| nsp12 | QVIVNNLDKSAGFPF | 1 |
| nsp12 | KWGKARLYYDSMSYE | 1 |
| nsp12 | ARLYYDSMSYEDQDALFAY | 3 |
| nsp12 | GATVVIGTSKFYGGW | 1 |
| nsp12 | TVYSDVENPHLMGWDYP | 2 |
| nsp12 | AQVLSEMVMCGGSLY | 1 |
| nsp12 | ADKYVRNLQHRLYECLY | 2 |
| nsp12 | NLQHRLYECLYRNRD | 1 |
| nsp12 | RLYECLYRNRDVDTDFVNEFYAY | 5 |
| nsp12 | HFSMMILSDDAVVCF | 1 |
| nsp12 | SVLYYQNNVFMSEAKCW | 2 |
| nsp12 | FCSQHTMLVKQGDDYVYLPYP | 4 |
| nsp12 | QGDDYVYLPYPDPSR | 1 |
| nsp12 | YLPYPDPSRILGAGC | 1 |
| nsp12 | PSRILGAGCFVDDIV | 1 |
| nsp12 | LMIERFVSLAIDAYP | 1 |
| nsp12 | PLTKHPNQEYADVFHLYLQYI | 4 |
| nsp12 | IRKLHDELTGHMLDMYSVM | 3 |
| nsp12 | MYSVMLTNDNTSRYW | 1 |
| nsp12 | MLTNDNTSRYWEPEFYEAMYTPHTVLQ | 7 |
| nsp13 | ACIRRPFLCCKCCYD | 1 |
| nsp13 | VLSVNPYVCNAPGCD | 1 |
| nsp13 | CNAPGCDVTDVTQLY | 1 |
| nsp13 | DVTDVTQLYLGGMSYYC | 2 |
| nsp13 | SDNVTDFNAIATCDW | 1 |
| nsp13 | FNAIATCDWTNAGDYIL | 2 |
| nsp13 | VREVLSDRELHLSWE | 1 |
| nsp13 | HLSWEVGKPRPPLNRNY | 2 |
| nsp13 | GEYTFEKGDYGDAVVYR | 2 |
| nsp13 | LVPQEHYVRITGLYP | 1 |
| nsp13 | AAVDALCEKALKYLP | 1 |
| nsp13 | CSRIIPARARVECFD | 1 |
| nsp13 | VNALPETTADIVVFDEISM | 3 |
| nsp13 | ADIVVFDEISMATNYDL | 2 |
| nsp13 | PRTLLTKGTLEPEYF | 1 |
| nsp13 | FNSVCRLMKTIGPDM | 1 |
| nsp13 | CPAEIVDTVSALVYD | 1 |
| nsp13 | QIGVVREFLTRNPAW | 1 |
| nsp13 | TQTVDSSQGSEYDYVIF | 2 |
| nsp13 | AKVGILCIMSDRDLYDK | 2 |
| nsp13 | DRDLYDKLQFTSLEI | 1 |
| nsp14 | ISMMGFKMNYQVNGY | 1 |
| nsp14 | GFKMNYQVNGYPNMF | 1 |
| nsp14 | RHVRAWIGFDVEGCH | 1 |
| nsp14 | QFKHLIPLMYKGLPW | 1 |
| nsp14 | YFVKIGPERTCCLCD | 1 |
| nsp14 | RATCFSTASDTYACW | 1 |
| nsp14 | SDTYACWHHSIGFDYVYNPFMIDVQQWGF | 8 |
| nsp14 | WGFTGNLQSNHDLYC | 1 |
| nsp14 | DAIMTRCLAVHECFV | 1 |
| nsp14 | HECFVKRVDWTIEYPII | 2 |
| nsp14 | RVDWTIEYPIIGDELKI | 2 |
| nsp14 | EYPIIGDELKINAAC | 1 |
| nsp14 | AIKCVPQADVEWKFY | 1 |
| nsp14 | AQPCSDKAYKIEELFYS | 2 |
| nsp14 | IEELFYSYATHSDKFTD | 2 |
| nsp14 | ATHSDKFTDGVCLFWNC | 2 |
| nsp14 | FDTRVLSNLNLPGCD | 1 |
| nsp14 | VLSNLNLPGCDGGSLYV | 2 |
| nsp14 | FDKSAFVNLKQLPFFYYSDSPCESH | 6 |
| nsp14 | PCESHGKQVVSDIDY | 1 |
| nsp14 | LGGAVCRHHANEYRLYL | 2 |
| nsp14 | RHHANEYRLYLDAYNMM | 2 |
| nsp14 | LDAYNMMISAGFSLW | 1 |
| nsp14 | SAGFSLWVYKQFDTYNL | 2 |
| nsp14 | WVYKQFDTYNLWNTF | 1 |
| nsp15 | HFDGQQGEVPVSIIN | 1 |
| nsp15 | NNTVYTKVDGVDVELFE | 2 |
| nsp15 | ENKTTLPVNVAFELW | 1 |
| nsp15 | NLGVDIAANTVIWDY | 1 |
| nsp15 | KPTETICAPLTVFFD | 1 |
| nsp15 | TVFFDGRVDGQVDLFRN | 2 |
| nsp15 | RVDGQVDLFRNARNG | 1 |
| nsp15 | NARNGVLITEGSVKG | 1 |
| nsp15 | LQEFKPRSQMEIDFL | 1 |
| nsp15 | QMEIDFLELAMDEFIERYK | 3 |
| nsp15 | LAMDEFIERYKLEGYAFEH | 3 |
| nsp15 | KLEGYAFEHIVYGDFSH | 2 |
| nsp15 | FEHIVYGDFSHSQLGGLHL | 3 |
| nsp15 | LAKRFKESPFELEDF | 1 |
| nsp15 | PFELEDFIPMDSTVKNY | 2 |
| nsp15 | GSSKCVCSVIDLLLDDFVEIIKS | 5 |
| nsp15 | DDFVEIIKSQDLSVV | 1 |
| nsp15 | VKVTIDYTEISFMLW | 1 |
| nsp15 | SFMLWCKDGHVETFY | 1 |
| nsp16 | TLTLAVPYNMRVIHF | 1 |
| nsp16 | VLRQWLPTGTLLVDS | 1 |
| nsp16 | PTGTLLVDSDLNDFV | 1 |
| nsp16 | VHTANKWDLIISDMYDP | 2 |
| nsp16 | DMYDPKTKNVTKEND | 1 |
| nsp16 | TEHSWNADLYKLMGHFAWW | 3 |
| nsp16 | GCNYLGKPREQIDGY | 1 |
| nsp16 | KPREQIDGYVMHANYIFWR | 3 |
| nsp16 | PIQLSSYSLFDMSKF | 1 |
| Spike Protein | SGMFVFLVLLPLVSS | 1 |
| Spike Protein | KVFRSSVLHSTQDLF | 1 |
| Spike Protein | SSVLHSTQDLFLPFF | 1 |
| Spike Protein | TQDLFLPFFSNVTWF | 1 |
| Spike Protein | HVSGTNGTKRFDNPV | 1 |
| Spike Protein | KRFDNPVLPFNDGVYFA | 2 |
| Spike Protein | IVNNATNVVIKVCEFQF | 2 |
| Spike Protein | VIKVCEFQFCNDPFL | 1 |
| Spike Protein | CEFQFCNDPFLGVYY | 1 |
| Spike Protein | VYYHKNNKSWMESEFRVYS | 3 |
| Spike Protein | FRVYSSANNCTFEYV | 1 |
| Spike Protein | NCTFEYVSQPFLMDL | 1 |
| Spike Protein | VSQPFLMDLEGKQGN | 1 |
| Spike Protein | KNLREFVFKNIDGYFKI | 2 |
| Spike Protein | HRSYLTPGDSSSGWT | 1 |
| Spike Protein | SSGWTAGAAAYYVGY | 1 |
| Spike Protein | FPNITNLCPFGEVFN | 1 |
| Spike Protein | TNLCPFGEVFNATRF | 1 |
| Spike Protein | GEVFNATRFASVYAW | 1 |
| Spike Protein | RKRISNCVADYSVLY | 1 |
| Spike Protein | LNDLCFTNVYADSFV | 1 |
| Spike Protein | CFTNVYADSFVIRGD | 1 |
| Spike Protein | QIAPGQTGKIADYNYKL | 2 |
| Spike Protein | TGKIADYNYKLPDDF | 1 |
| Spike Protein | AWNSNNLDSKVGGNYNYLY | 3 |
| Spike Protein | YRLFRKSNLKPFERD | 1 |
| Spike Protein | SNLKPFERDISTEIY | 1 |
| Spike Protein | AGSTPCNGVEGFNCYFP | 2 |
| Spike Protein | NGVEGFNCYFPLQSY | 1 |
| Spike Protein | YGFQPTNGVGYQPYR | 1 |
| Spike Protein | VGYQPYRVVVLSFELLH | 2 |
| Spike Protein | DTTDAVRDPQTLEILDI | 2 |
| Spike Protein | AGCLIGAEHVNNSYECDIP | 3 |
| Spike Protein | HVNNSYECDIPIGAG | 1 |
| Spike Protein | LPVSMTKTSVDCTMY | 1 |
| Spike Protein | SVDCTMYICGDSTEC | 1 |
| Spike Protein | DKNTQEVFAQVKQIY | 1 |
| Spike Protein | VKQIYKTPPIKDFGG | 1 |
| Spike Protein | GFNFSQILPDPSKPS | 1 |
| Spike Protein | ILPDPSKPSKRSFIE | 1 |
| Spike Protein | PSKPSKRSFIEDLLF | 1 |
| Spike Protein | TLADAGFIKQYGDCLGD | 2 |
| Spike Protein | YGDCLGDIAARDLIC | 1 |
| Spike Protein | GLTVLPPLLTDEMIAQY | 2 |
| Spike Protein | ALQIPFAMQMAYRFN | 1 |
| Spike Protein | AYRFNGIGVTQNVLY | 1 |
| Spike Protein | QNVLYENQKLIANQF | 1 |
| Spike Protein | LSRLDKVEAEVQIDRLI | 2 |
| Spike Protein | ICHDGKAHFPREGVF | 1 |
| Spike Protein | FPREGVFVSNGTHWF | 1 |
| Spike Protein | SNGTHWFVTQRNFYE | 1 |
| Spike Protein | HWFVTQRNFYEPQII | 1 |
| Spike Protein | EPQIITTDNTFVSGNCD | 2 |
| Spike Protein | DPLQPELDSFKEELDKYFK | 3 |
| Spike Protein | DKYFKNHTSPDVDLGDI | 2 |
| Spike Protein | SPDVDLGDISGINASVV | 2 |
| Spike Protein | ISGINASVVNIQKEIDR | 2 |
| Spike Protein | LNESLIDLQELGKYEQY | 2 |
| Spike Protein | DLQELGKYEQYIKWPWYIW | 3 |
| Spike Protein | EQYIKWPWYIWLGFI | 1 |
| Spike Protein | IVMVTIMLCCMTSCC | 1 |
| Spike Protein | KGCCSCGSCCKFDEDDSEP | 3 |
| Spike Protein | CCKFDEDDSEPVLKG | 1 |
| GS Linker | HYTGSGSGSGMDLFM | 1 |
| Orf3a Protein | YSHLLLVAAGLEAPFLYLY | 3 |
| Orf3a Protein | LEAPFLYLYALVYFL | 1 |
| Orf3a Protein | WKCRSKNPLLYDANYFLCW | 3 |
| Orf3a Protein | YDANYFLCWHTNCYDYCIPYN | 4 |
| Orf3a Protein | SSIVITSGDGTTSPI | 1 |
| Orf3a Protein | ISEHDYQIGGYTEKWESGVKD | 4 |
| Orf3a Protein | WESGVKDCVVLHSYF | 1 |
| Orf3a Protein | VKDCVVLHSYFTSDYYQLY | 3 |
| Orf3a Protein | LSTDTGVEHVTFFIY | 1 |
| Orf3a Protein | YNKIVDEPEEHVQIH | 1 |
| Orf3a Protein | GSSGVVNPVMEPIYDEPTTTTSV | 5 |
| Envelope Protein | SFVSEETGTLIVNSV | 1 |
| Membrane Glycoprotein | SGSGSGMADSNGTITVEELKKLL | 5 |
| Membrane Glycoprotein | TITVEELKKLLEQWN | 1 |
| Membrane Glycoprotein | LEQWNLVIGFLFLTW | 1 |
| Membrane Glycoprotein | WICLLQFAYANRNRFLY | 2 |
| Membrane Glycoprotein | GLMWLSYFIASFRLF | 1 |
| Membrane Glycoprotein | HGTILTRPLLESELV | 1 |
| Membrane Glycoprotein | PKEITVATSRTLSYY | 1 |
| Membrane Glycoprotein | QRVAGDSGFAAYSRY | 1 |
| Membrane Glycoprotein | GDSGFAAYSRYRIGN | 1 |
| Membrane Glycoprotein | YRIGNYKLNTDHSSSSDNI | 3 |
| Orf6 Protein | GMFHLVDFQVTIAEILLII | 3 |
| Orf6 Protein | IMRTFKVSIWNLDYI | 1 |
| Orf6 Protein | KYSQLDEEQPMEIDG | 1 |
| Orf7a Protein | ILFLALITLATCELYHYQECVRG | 5 |
| Orf7a Protein | VRGTTVLLKEPCSSG | 1 |
| Orf7a Protein | TVLLKEPCSSGTYEG | 1 |
| Orf7a Protein | KEPCSSGTYEGNSPFHP | 2 |
| Orf7a Protein | YEGNSPFHPLADNKF | 1 |
| Orf7a Protein | AFACPDGVKHVYQLRAR | 2 |
| Orf8 Protein | TQHQPYVVDDPCPIHFY | 2 |
| Orf8 Protein | VVDDPCPIHFYSKWY | 1 |
| Orf8 Protein | VDEAGSKSPIQYIDIGN | 2 |
| Orf8 Protein | KSPIQYIDIGNYTVSCL | 2 |
| Orf8 Protein | KLGSLVVRCSFYEDFLEYHDVRVVLDF | 7 |
| Nucleocapsid Phosphoprotein | SDNGPQNQRNAPRIT | 1 |
| Nucleocapsid Phosphoprotein | PQNQRNAPRITFGGP | 1 |
| Nucleocapsid Phosphoprotein | WFTALTQHGKEDLKF | 1 |
| Nucleocapsid Phosphoprotein | VPINTNSSPDDQIGYYR | 2 |
| Nucleocapsid Phosphoprotein | IRGGDGKMKDLSPRWYFYY | 3 |
| Nucleocapsid Phosphoprotein | EAGLPYGANKDGIIW | 1 |
| Nucleocapsid Phosphoprotein | DGIIWVATEGALNTP | 1 |
| Nucleocapsid Phosphoprotein | AIVLQLPQGTTLPKGFYAEGS | 4 |
| Nucleocapsid Phosphoprotein | GSSRGTSPARMAGNGGDAALALLLLDR | 7 |
| Nucleocapsid Phosphoprotein | GKGQQQQGQTVTKKSAA | 2 |
| Nucleocapsid Phosphoprotein | KAYNVTQAFGRRGPE | 1 |
| Nucleocapsid Phosphoprotein | GPEQTQGNFGDQELI | 1 |
| Nucleocapsid Phosphoprotein | GNFGDQELIRQGTDY | 1 |
| Nucleocapsid Phosphoprotein | HWPQIAQFAPSASAF | 1 |
| Nucleocapsid Phosphoprotein | SASAFFGMSRIGMEV | 1 |
| Nucleocapsid Phosphoprotein | GMSRIGMEVTPSGTW | 1 |
| Nucleocapsid Phosphoprotein | QTVTLLPAADLDDFSKQ | 2 |
