# Supplementary material for: Humoral Immune Response Profile of COVID-19 Reveals Severity and Variant-Specific Epitopes: Lessons from SARS-CoV-2 Peptide Microarray
Source: Viruses. 2023 Jan 15;15(1):248. doi: 10.3390/v15010248 (PMC9866125; doi:10.3390/v15010248)
Supplement: Supplementary file 1 [file viruses-15-00248-s001.zip › Table S6.docx]

Table S6. Severity-based discrimination in IgA response against SARS-CoV-2 peptides

| Protein | Sequence | Peptides | NS1 | NS2 | NS3 | NS4 | NS5 | NS6 | SV1 | SV2 | SV3 | SV4 | SV5 | SV6 | P-value |
| --- | --- | --- | --- | --- | --- | --- | --- | --- | --- | --- | --- | --- | --- | --- | --- |
| nsp3 | aa443-457 | FLTENLLLYIDINGN | 1.22 | 0.48 | 0.27 | 0.02 | -0.84 | -0.20 | 1.49 | 3.46 | 0.99 | 0.55 | 0.75 | 0.49 | 0.03 |
| nsp3 | aa1011-1025 | LKHGTFTCASEYTGN | 0.84 | -0.10 | 0.30 | -1.17 | -1.16 | -0.37 | 0.63 | 3.31 | 1.20 | 0.66 | -0.16 | 1.04 | 0.05 |
| nsp3 | aa1535-1549 | VLGLAAIMQLFFSYF | 2.26 | 1.14 | 0.70 | 0.52 | 0.68 | 1.70 | 3.08 | 1.44 | 2.02 | 1.37 | 2.42 | 2.58 | 0.05 |
| nsp12 | aa225-239 | QTTPGSGVPVVDSYY | 1.70 | 1.39 | 1.55 | 0.99 | 0.80 | 1.89 | 3.18 | 1.59 | 2.51 | 1.64 | 1.94 | 1.90 | 0.03 |
| nsp12 | aa869-883 | PLTKHPNQEYADVFH | 0.68 | -0.26 | -0.48 | 0.13 | -0.32 | -0.99 | 3.08 | 1.00 | 0.67 | -0.15 | 0.85 | 0.56 | 0.03 |
| nsp14 | aa143-157 | GDQFKHLIPLMYKGL | 0.17 | -0.35 | -0.51 | -1.25 | -1.55 | -0.96 | 0.31 | -0.26 | -0.40 | -0.20 | 3.04 | -0.30 | 0.05 |
