# Supplementary material for: Humoral Immune Response Profile of COVID-19 Reveals Severity and Variant-Specific Epitopes: Lessons from SARS-CoV-2 Peptide Microarray
Source: Viruses. 2023 Jan 15;15(1):248. doi: 10.3390/v15010248 (PMC9866125; doi:10.3390/v15010248)
Supplement: Supplementary file 1 [file viruses-15-00248-s001.zip › Table S7.docx]

Table S7. Severity-based discrimination in IgG response against SARS-CoV-2 peptides

| Protein | Sequence | Peptides | NS1 | NS2 | NS3 | NS4 | NS5 | NS6 | SV1 | SV2 | SV3 | SV4 | SV5 | SV6 | P-val |
| --- | --- | --- | --- | --- | --- | --- | --- | --- | --- | --- | --- | --- | --- | --- | --- |
| nsp1 | aa57-71 | TCGLVEVEKGVLPQL | -0.81 | -0.79 | -0.78 | -0.82 | -0.86 | -0.55 | -0.62 | 1.43 | -0.65 | -0.78 | 3.21 | -0.17 | 0.03 |
| nsp1 | aa61-75 | VEVEKGVLPQLEQPY | -0.56 | 0.68 | 0.28 | -0.79 | -0.47 | -0.22 | -0.05 | 3.45 | 0.32 | 0.64 | 2.74 | 0.56 | 0.05 |
| nsp1 | aa63-77 | VEKGVLPQLEQPYVF | -0.61 | -0.44 | -0.46 | -0.81 | -0.74 | -0.43 | -0.22 | 3.10 | 0.03 | -0.62 | 2.67 | 0.31 | 0.03 |
| nsp1 | aa137-151 | GAGGHSYGADLKSFD | -0.29 | 1.23 | -0.51 | -0.72 | -0.70 | 0.89 | 1.47 | 3.51 | 1.14 | 0.36 | 2.02 | 1.51 | 0.02 |
| nsp1 | aa141-155 | HSYGADLKSFDLGDE | 0.23 | 0.88 | 0.05 | -0.17 | -0.02 | 0.61 | 0.77 | 4.05 | 1.93 | 1.27 | 2.22 | 2.54 | 0.01 |
| nsp1 | aa143-157 | YGADLKSFDLGDELG | 0.06 | 0.70 | 0.00 | -0.02 | 0.35 | 1.03 | 0.78 | 3.05 | 1.65 | 0.90 | 2.00 | 2.10 | 0.01 |
| nsp1 | aa147-161 | LKSFDLGDELGTDPY | 0.90 | 1.64 | 0.18 | 0.94 | 0.51 | 1.59 | 2.02 | 4.35 | 2.18 | 1.98 | 4.53 | 3.43 | 0.01 |
| nsp1 | aa149-163 | SFDLGDELGTDPYED | 2.04 | 2.48 | 1.19 | 1.65 | 1.52 | 3.03 | 2.57 | 4.54 | 3.03 | 2.73 | 3.90 | 4.57 | 0.02 |
| nsp1 | aa151-165 | DLGDELGTDPYEDFQ | 1.33 | 2.22 | 0.60 | 0.87 | 0.59 | 1.73 | 2.35 | 4.54 | 2.57 | 1.67 | 3.35 | 4.03 | 0.01 |
| nsp1 | aa153-167 | GDELGTDPYEDFQEN | 0.41 | 2.32 | 0.49 | 0.22 | 0.07 | 1.12 | 1.96 | 3.55 | 2.34 | 1.90 | 3.22 | 3.48 | 0.01 |
| nsp1 | aa155-169 | ELGTDPYEDFQENWN | -0.01 | 1.76 | 0.61 | 0.34 | -0.11 | 1.68 | 2.22 | 4.39 | 2.45 | 1.57 | 3.96 | 3.95 | 0.01 |
| nsp2 | aa9-23 | ELNGGAYTRYVDNNF | -0.77 | 0.58 | 0.14 | -0.82 | -0.76 | -0.65 | 1.10 | 3.27 | -0.41 | -0.09 | 2.54 | 0.54 | 0.05 |
| nsp2 | aa13-27 | GAYTRYVDNNFCGPD | -0.50 | -0.65 | -0.14 | -0.79 | -0.79 | -0.31 | 0.63 | 0.47 | 0.27 | 0.98 | 3.82 | 1.04 | 0.01 |
| nsp2 | aa15-29 | YTRYVDNNFCGPDGY | 0.26 | 1.11 | 1.14 | -0.01 | -0.18 | 0.21 | 1.71 | 4.49 | 2.27 | 1.76 | 4.30 | 2.68 | 0.01 |
| nsp2 | aa17-31 | RYVDNNFCGPDGYPL | -0.51 | -0.03 | 0.80 | -0.83 | -0.82 | -0.15 | 0.92 | 3.11 | 1.31 | 0.86 | 2.98 | 1.98 | 0.01 |
| nsp2 | aa19-33 | VDNNFCGPDGYPLEC | 0.88 | 1.51 | 1.48 | 0.23 | 1.16 | 1.64 | 1.53 | 4.04 | 2.55 | 4.60 | 1.94 | 2.48 | 0.01 |
| nsp2 | aa59-73 | RGVYCCREHEHEIAW | 0.54 | 1.48 | 0.90 | 0.27 | -0.32 | 1.26 | 1.72 | 4.12 | 1.97 | 1.28 | 3.77 | 3.08 | 0.01 |
| nsp2 | aa61-75 | VYCCREHEHEIAWYT | -0.01 | 0.88 | 0.70 | -0.06 | -0.36 | 0.50 | 1.14 | 3.69 | 0.90 | 0.43 | 3.25 | 2.69 | 0.02 |
| nsp2 | aa63-77 | CCREHEHEIAWYTER | -0.19 | 1.45 | 1.16 | 0.26 | -0.34 | 1.52 | 1.46 | 4.13 | 2.03 | 1.25 | 3.76 | 3.19 | 0.02 |
| nsp2 | aa89-103 | EIKLAKKFDTFNGEC | -0.44 | -0.02 | 0.18 | -0.23 | 0.24 | 0.83 | 0.15 | 1.97 | 1.52 | 3.18 | 1.34 | 1.11 | 0.02 |
| nsp2 | aa155-169 | KCDHCGETSWQTGDF | -0.56 | 0.54 | -0.22 | -0.59 | -0.40 | -0.15 | 0.66 | 3.51 | 1.97 | 0.36 | 2.57 | 1.72 | 0.01 |
| nsp2 | aa207-221 | HNSEVGPEHSLAEYH | 0.72 | 0.86 | 0.20 | -0.46 | -0.71 | -0.33 | 0.51 | 3.90 | 1.34 | 0.85 | 2.99 | 1.56 | 0.02 |
| nsp2 | aa235-249 | RTIAFGGCVFSYVGC | 0.03 | -0.13 | 1.09 | -0.19 | -0.44 | 0.67 | 0.32 | 1.53 | 1.49 | 4.13 | 0.85 | 0.85 | 0.03 |
| nsp2 | aa295-309 | NIVGDFKLNEEIAII | 0.40 | 0.68 | 0.10 | -0.73 | -0.71 | -0.24 | 0.78 | 3.55 | 2.13 | 0.52 | 1.62 | 0.42 | 0.01 |
| nsp2 | aa315-329 | ASTSAFVETVKGLDY | -0.33 | -0.01 | -0.09 | -0.53 | -0.45 | 0.22 | 0.29 | 3.47 | 2.61 | -0.08 | 1.78 | 1.06 | 0.01 |
| nsp2 | aa449-463 | IFGTVYEKLKPVLDW | -0.37 | 0.10 | -0.07 | 0.00 | 0.10 | 1.91 | 0.74 | 2.89 | 1.63 | 0.03 | 3.45 | 2.37 | 0.05 |
| nsp2 | aa461-475 | LDWLEEKFKEGVEFL | -0.04 | 1.78 | 0.93 | 0.46 | 0.74 | 2.46 | 1.74 | 4.65 | 2.92 | 1.21 | 3.90 | 3.50 | 0.03 |
| nsp2 | aa463-477 | WLEEKFKEGVEFLRD | 1.21 | 1.14 | 0.60 | 0.22 | 0.47 | 2.49 | 1.26 | 4.05 | 2.91 | 1.70 | 3.40 | 3.51 | 0.01 |
| nsp2 | aa465-479 | EEKFKEGVEFLRDGW | 0.18 | 1.30 | 1.00 | 0.66 | 0.75 | 3.01 | 1.95 | 4.70 | 3.29 | 0.95 | 4.71 | 3.61 | 0.03 |
| nsp2 | aa467-481 | KFKEGVEFLRDGWEI | -0.04 | 1.52 | 1.29 | 0.63 | 0.59 | 2.63 | 1.61 | 4.43 | 3.46 | 1.53 | 3.96 | 3.69 | 0.01 |
| nsp3 | aa17-31 | KVTFGDDTVIEVQGY | 0.28 | 0.55 | 0.24 | -0.01 | -0.52 | -0.11 | 0.29 | 3.35 | 1.61 | 0.41 | 2.03 | 1.85 | 0.01 |
| nsp3 | aa55-69 | YTVELGTEVNEFACV | 0.18 | -0.36 | 0.54 | 0.21 | -0.20 | -0.26 | 0.59 | 3.20 | 1.95 | 1.97 | 0.17 | 1.15 | 0.02 |
| nsp3 | aa81-95 | VSELLTPLGIDLDEW | 1.52 | 2.94 | 2.07 | 1.34 | 1.28 | 2.95 | 2.16 | 5.64 | 3.09 | 2.43 | 4.35 | 3.85 | 0.03 |
| nsp3 | aa83-97 | ELLTPLGIDLDEWSM | 0.79 | 1.40 | 0.98 | 0.42 | 1.21 | 2.69 | 1.02 | 4.51 | 1.68 | 1.42 | 3.24 | 3.01 | 0.05 |
| nsp3 | aa87-101 | PLGIDLDEWSMATYY | 1.16 | 3.01 | 1.48 | 0.94 | 0.38 | 2.38 | 2.66 | 5.31 | 3.38 | 2.78 | 4.96 | 3.94 | 0.01 |
| nsp3 | aa89-103 | GIDLDEWSMATYYLF | 0.75 | 3.15 | 1.34 | 0.75 | 0.64 | 2.32 | 2.28 | 5.07 | 3.24 | 2.15 | 4.52 | 3.41 | 0.03 |
| nsp3 | aa91-105 | DLDEWSMATYYLFDE | 1.72 | 2.83 | 2.34 | 1.48 | 1.23 | 2.92 | 2.32 | 5.32 | 3.53 | 2.36 | 4.04 | 3.41 | 0.05 |
| nsp3 | aa93-107 | DEWSMATYYLFDESG | 0.04 | 0.71 | 0.02 | 0.33 | -0.04 | 1.66 | 1.00 | 3.25 | 2.70 | 1.10 | 3.02 | 2.74 | 0.01 |
| nsp3 | aa95-109 | WSMATYYLFDESGEF | 1.58 | 2.32 | 1.75 | 1.14 | 0.52 | 2.16 | 2.11 | 4.92 | 3.99 | 2.39 | 4.08 | 3.69 | 0.01 |
| nsp3 | aa103-117 | FDESGEFKLASHMYC | 0.11 | 0.10 | 1.51 | -0.35 | -0.81 | 1.45 | 0.42 | 3.66 | 3.08 | 0.75 | 2.98 | 2.95 | 0.03 |
| nsp3 | aa107-121 | GEFKLASHMYCSFYP | -0.36 | -0.31 | -0.13 | -0.72 | -0.84 | 0.35 | -0.18 | 3.26 | 2.48 | 0.08 | 3.36 | 0.93 | 0.02 |
| nsp3 | aa117-131 | CSFYPPDEDEEEGDC | 2.10 | 2.44 | 1.86 | 1.89 | 1.31 | 3.19 | 1.96 | 4.67 | 3.79 | 3.78 | 2.33 | 3.87 | 0.05 |
| nsp3 | aa119-133 | FYPPDEDEEEGDCEE | 2.44 | 2.80 | 1.76 | 2.03 | 1.76 | 3.38 | 1.99 | 4.89 | 3.81 | 3.88 | 3.65 | 3.82 | 0.03 |
| nsp3 | aa129-143 | GDCEEEEFEPSTQYE | 1.57 | 2.65 | 1.58 | 1.49 | 1.33 | 2.64 | 1.72 | 4.22 | 3.17 | 2.48 | 3.61 | 2.69 | 0.03 |
| nsp3 | aa131-145 | CEEEEFEPSTQYEYG | 1.89 | 2.48 | 1.43 | 1.62 | 1.45 | 2.24 | 1.72 | 4.68 | 3.14 | 2.56 | 4.03 | 3.26 | 0.02 |
| nsp3 | aa133-147 | EEEFEPSTQYEYGTE | 1.16 | 1.84 | 1.21 | 0.80 | 0.92 | 2.55 | 1.52 | 1.96 | 1.91 | 2.18 | 3.99 | 3.31 | 0.05 |
| nsp3 | aa135-149 | EFEPSTQYEYGTEDD | 1.36 | 2.03 | 1.24 | 1.13 | 1.04 | 2.27 | 1.37 | 3.64 | 2.30 | 2.47 | 2.79 | 3.28 | 0.01 |
| nsp3 | aa137-151 | EPSTQYEYGTEDDYQ | 0.71 | 1.77 | 1.08 | 1.10 | 0.30 | 1.40 | 1.86 | 4.09 | 2.04 | 1.90 | 3.10 | 3.30 | 0.01 |
| nsp3 | aa143-157 | EYGTEDDYQGKPLEF | 0.05 | 0.48 | 0.35 | 0.35 | -0.02 | 1.42 | 1.06 | 3.27 | 1.79 | 0.36 | 3.89 | 2.22 | 0.02 |
| nsp3 | aa221-235 | NSFSGYLKLTDNVYI | -0.72 | -0.47 | -0.62 | -0.69 | -0.83 | -0.54 | 1.80 | 3.07 | 0.91 | -0.73 | 2.15 | 0.14 | 0.05 |
| nsp3 | aa319-333 | KGEDIQLLKSAYENF | -0.61 | 0.57 | 0.09 | -0.40 | -0.47 | 1.73 | 0.73 | 3.68 | 1.33 | -0.03 | 2.87 | 2.68 | 0.05 |
| nsp3 | aa365-379 | RTNVYLAVFDKNLYD | 0.69 | 1.35 | 1.01 | 0.05 | -0.32 | 0.62 | 2.12 | 4.60 | 3.22 | 0.91 | 3.38 | 1.68 | 0.01 |
| nsp3 | aa437-451 | TLEETKFLTENLLLY | 0.66 | 1.97 | 1.17 | 0.57 | 0.57 | 0.72 | 1.31 | 4.05 | 2.20 | 1.01 | 3.22 | 1.28 | 0.03 |
| nsp3 | aa441-455 | TKFLTENLLLYIDIN | -0.53 | -0.45 | -0.28 | -0.59 | -0.73 | -0.62 | -0.12 | 3.04 | 0.78 | -0.67 | 1.11 | 1.01 | 0.05 |
| nsp3 | aa443-457 | FLTENLLLYIDINGN | -0.61 | -0.49 | -0.58 | -0.76 | -0.83 | -0.35 | -0.48 | 5.82 | -0.09 | -0.04 | 1.03 | 0.19 | 0.01 |
| nsp3 | aa459-473 | HPDSATLVSDIDITF | -0.50 | 0.70 | -0.08 | -0.25 | -0.76 | -0.30 | 0.03 | 4.11 | 1.47 | 0.43 | 2.66 | 1.59 | 0.01 |
| nsp3 | aa603-617 | GIKIQEGVVDYGARF | -0.47 | 0.63 | 0.21 | 0.09 | -0.08 | 1.75 | 0.76 | 3.33 | 2.44 | 0.08 | 3.89 | 2.32 | 0.05 |
| nsp3 | aa605-619 | KIQEGVVDYGARFYF | -0.64 | 1.19 | 0.09 | 0.07 | -0.26 | 1.09 | 1.34 | 3.86 | 2.13 | 0.46 | 4.35 | 2.23 | 0.01 |
| nsp3 | aa607-621 | QEGVVDYGARFYFYT | -0.24 | 0.57 | 0.32 | -0.42 | -0.67 | 0.11 | -0.03 | 3.16 | 1.33 | 0.38 | 3.19 | 0.34 | 0.05 |
| nsp3 | aa633-647 | LNDLNETLVTMPLGY | -0.06 | 0.42 | 0.69 | -0.35 | -0.49 | 0.97 | 0.05 | 3.43 | 2.42 | 0.53 | 2.91 | 2.01 | 0.05 |
| nsp3 | aa647-661 | YVTHGLNLEEAARYM | -0.34 | -0.04 | 0.79 | -0.13 | -0.11 | 1.68 | 0.98 | 3.14 | 1.52 | 0.63 | 2.13 | 2.18 | 0.03 |
| nsp3 | aa697-711 | IETISLAGSYKDWSY | 0.23 | -0.31 | -0.14 | -0.35 | -0.08 | 1.86 | 0.40 | 3.92 | 1.90 | 1.14 | 3.33 | 1.45 | 0.02 |
| nsp3 | aa709-723 | WSYSGQSTQLGIEFL | -0.29 | 1.55 | 0.32 | -0.12 | -0.44 | -0.03 | 0.97 | 3.11 | 2.50 | 0.26 | 1.72 | 1.72 | 0.02 |
| nsp3 | aa717-731 | QLGIEFLKRGDKSVY | -0.48 | 1.03 | -0.05 | -0.46 | -0.79 | 0.45 | 1.04 | 3.68 | 1.83 | 0.21 | 2.87 | 0.78 | 0.02 |
| nsp3 | aa735-749 | NPTTFHLDGEVITFD | 0.10 | 1.75 | 0.87 | -0.09 | -0.28 | 1.23 | 1.27 | 4.00 | 2.85 | 1.87 | 2.25 | 1.33 | 0.01 |
| nsp3 | aa775-789 | HTQVVDMSMTYGQQF | -0.83 | -0.04 | -0.69 | -0.40 | -0.78 | -0.11 | 0.13 | 3.61 | -0.18 | -0.47 | 1.02 | 1.14 | 0.05 |
| nsp3 | aa813-827 | FYVLPNDDTLRVEAF | -0.59 | 0.36 | -0.34 | -0.24 | -0.42 | -0.39 | 0.76 | 2.90 | 1.94 | -0.49 | 3.28 | 1.73 | 0.05 |
| nsp3 | aa815-829 | VLPNDDTLRVEAFEY | 1.23 | 2.80 | 1.97 | 1.10 | 0.98 | 2.03 | 2.05 | 4.87 | 3.18 | 2.51 | 4.36 | 3.32 | 0.01 |
| nsp3 | aa817-831 | PNDDTLRVEAFEYYH | 0.83 | 2.74 | 1.68 | 0.76 | 0.34 | 1.52 | 2.71 | 4.80 | 3.43 | 2.27 | 4.35 | 2.44 | 0.02 |
| nsp3 | aa821-835 | TLRVEAFEYYHTTDP | -0.72 | -0.44 | -0.69 | -0.63 | -0.57 | -0.56 | 0.17 | 0.96 | -0.43 | -0.52 | 7.60 | -0.05 | 0.01 |
| nsp3 | aa823-837 | RVEAFEYYHTTDPSF | -0.41 | 0.20 | 0.12 | -0.16 | -0.71 | -0.19 | 1.07 | 2.25 | 0.28 | 0.04 | 6.22 | 0.27 | 0.01 |
| nsp3 | aa827-841 | FEYYHTTDPSFLGRY | 0.18 | 1.35 | 1.30 | 0.04 | -0.13 | 1.02 | 2.42 | 4.67 | 2.72 | 1.88 | 5.38 | 3.31 | 0.01 |
| nsp3 | aa857-871 | NGLTSIKWADNNCYL | -0.36 | 0.49 | -0.27 | -0.53 | -0.62 | -0.04 | 0.48 | 3.37 | 1.98 | -0.17 | 2.68 | 0.39 | 0.03 |
| nsp3 | aa881-895 | IELKFNPPALQDAYY | 0.76 | 2.23 | 1.61 | 0.39 | -0.21 | 0.62 | 2.37 | 5.00 | 3.58 | 2.57 | 4.13 | 2.84 | 0.01 |
| nsp3 | aa899-913 | AGEAANFCALILAYC | 0.02 | 0.39 | 1.17 | 0.24 | -0.07 | 0.83 | 0.78 | 3.31 | 2.08 | 1.54 | 1.91 | 1.46 | 0.01 |
| nsp3 | aa917-931 | VGELGDVRETMSYLF | -0.18 | 1.51 | 0.39 | 0.55 | 0.16 | 1.74 | 1.08 | 3.75 | 1.56 | 0.62 | 2.76 | 2.41 | 0.05 |
| nsp3 | aa1011-1025 | LKHGTFTCASEYTGN | -0.85 | -0.61 | -0.24 | -0.83 | -0.86 | -0.38 | -0.72 | 5.66 | 1.31 | 0.25 | 0.03 | 0.00 | 0.02 |
| nsp3 | aa1017-1031 | TCASEYTGNYQCGHY | -0.75 | 0.78 | 0.07 | -0.67 | -0.78 | -0.36 | 0.92 | 2.71 | 1.49 | 0.15 | 3.18 | 2.04 | 0.01 |
| nsp3 | aa1033-1047 | HITSKETLYCIDGAL | -0.70 | -0.52 | -0.47 | -0.58 | -0.81 | -0.13 | -0.43 | 3.41 | 0.99 | -0.03 | 1.23 | 0.68 | 0.01 |
| nsp3 | aa1049-1063 | TKSSEYKGPITDVFY | -0.62 | 0.94 | 0.05 | -0.08 | -0.47 | 0.13 | 0.61 | 4.25 | 0.94 | 0.34 | 2.37 | 0.79 | 0.03 |
| nsp3 | aa1081-1095 | GVVCTEIDPKLDNYY | 1.09 | 2.62 | 1.13 | 0.31 | -0.15 | -0.29 | 1.84 | 4.99 | 2.61 | 1.79 | 4.30 | 3.53 | 0.02 |
| nsp3 | aa1085-1099 | TEIDPKLDNYYKKDN | -0.51 | 0.66 | -0.73 | -0.69 | -0.83 | -0.20 | 0.95 | 2.41 | -0.14 | 0.05 | 3.11 | 0.61 | 0.02 |
| nsp3 | aa1087-1101 | IDPKLDNYYKKDNSY | -0.58 | -0.51 | -0.70 | -0.78 | -0.83 | -0.70 | -0.36 | 3.03 | -0.24 | -0.30 | 2.84 | 1.72 | 0.01 |
| nsp3 | aa1101-1115 | YFTEQPIDLVPNQPY | -0.77 | 0.93 | 0.14 | -0.73 | -0.78 | -0.23 | 0.66 | 2.75 | 0.53 | -0.18 | 3.10 | 1.04 | 0.03 |
| nsp3 | aa1109-1123 | LVPNQPYPNASFDNF | -0.79 | 1.26 | 0.05 | -0.55 | -0.83 | -0.45 | 1.37 | 3.73 | 1.44 | 0.54 | 3.69 | 2.52 | 0.01 |
| nsp3 | aa1123-1137 | FKFVCDNIKFADDLN | -0.75 | 0.85 | 1.24 | -0.76 | -0.40 | -0.28 | 0.15 | 2.71 | 1.52 | 0.05 | 3.07 | 2.98 | 0.03 |
| nsp3 | aa1145-1159 | PASRELKVTFFPDLN | -0.63 | -0.64 | -0.19 | -0.68 | -0.44 | 0.02 | 0.11 | 3.35 | 1.33 | -0.27 | 0.59 | 2.38 | 0.01 |
| nsp3 | aa1153-1167 | TFFPDLNGDVVAIDY | 0.37 | 1.21 | 1.92 | 0.34 | 0.44 | 1.64 | 1.79 | 4.62 | 2.95 | 1.67 | 3.12 | 2.67 | 0.01 |
| nsp3 | aa1173-1187 | SFKKGAKLLHKPIVW | -0.50 | -0.72 | -0.62 | -0.58 | -0.47 | 0.98 | -0.42 | 3.20 | 1.01 | -0.53 | 1.99 | 0.71 | 0.05 |
| nsp3 | aa1195-1209 | KATYKPNTWCIRCLW | 0.08 | 0.38 | -0.07 | 0.00 | 0.03 | 1.66 | 0.44 | 3.25 | 1.95 | 0.22 | 3.66 | 2.41 | 0.02 |
| nsp3 | aa1257-1271 | LECNVKTTEVVGDII | 0.45 | 0.87 | 0.32 | 0.68 | -0.48 | 0.24 | 1.08 | 3.08 | 2.46 | 1.61 | 0.90 | -0.20 | 0.05 |
| nsp3 | aa1281-1295 | ITEEVGHTDLMAAYV | -0.74 | 0.12 | 0.19 | -0.63 | -0.85 | 0.99 | 0.20 | 2.44 | 1.23 | 0.18 | 2.26 | 3.56 | 0.02 |
| nsp3 | aa1317-1331 | LATHGLAAVNSVPWD | -0.01 | 0.71 | 0.00 | -0.03 | -0.69 | 0.83 | 1.62 | 3.98 | 1.81 | 1.41 | 3.24 | 1.52 | 0.01 |
| nsp3 | aa1353-1367 | TRCLNRVCTNYMPYF | -0.39 | 0.70 | -0.24 | -0.37 | -0.62 | 0.26 | 1.92 | 3.73 | 0.90 | 0.41 | 3.23 | 1.31 | 0.01 |
| nsp3 | aa1399-1413 | KSVGKFCLEASFNYL | -0.42 | 0.82 | -0.10 | -0.66 | -0.76 | -0.31 | 0.47 | 3.27 | 1.23 | 0.47 | 2.24 | 0.38 | 0.02 |
| nsp3 | aa1451-1465 | NLGMPSYCTGYREGY | 0.49 | 1.39 | 0.80 | 0.79 | 0.89 | 2.17 | 1.71 | 4.61 | 2.25 | 1.19 | 4.45 | 3.29 | 0.02 |
| nsp3 | aa1453-1467 | GMPSYCTGYREGYLN | -0.21 | 0.87 | -0.53 | 0.22 | -0.53 | 1.30 | 0.68 | 3.99 | 1.02 | 0.43 | 3.78 | 1.95 | 0.05 |
| nsp3 | aa1483-1497 | CSVCLSGLDSLDTYP | -0.17 | 0.24 | 0.16 | -0.02 | -0.78 | 1.44 | 0.31 | 3.72 | 0.51 | 0.91 | 1.25 | 0.75 | 0.05 |
| nsp3 | aa1509-1523 | FKWDLTAFGLVAEWF | 0.67 | 2.30 | 1.27 | 0.70 | 0.56 | 2.63 | 2.34 | 4.97 | 3.25 | 1.38 | 4.28 | 3.49 | 0.02 |
| nsp3 | aa1515-1529 | AFGLVAEWFLAYILF | -0.57 | 0.87 | 0.08 | -0.68 | -0.20 | 0.41 | 1.06 | 3.57 | 2.35 | 0.16 | 3.16 | 1.98 | 0.01 |
| nsp3 | aa1535-1549 | VLGLAAIMQLFFSYF | -0.57 | 0.93 | 0.57 | -0.63 | -0.74 | 0.32 | 0.93 | 3.51 | 2.23 | 0.56 | 3.26 | 2.16 | 0.01 |
| nsp3 | aa1547-1561 | SYFAVHFISNSWLMW | -0.40 | 1.06 | 0.74 | 0.50 | -0.11 | 3.20 | 1.41 | 3.48 | 3.23 | 0.59 | 3.96 | 3.17 | 0.05 |
| nsp3 | aa1549-1563 | FAVHFISNSWLMWLI | 0.07 | 0.53 | 0.45 | -0.06 | -0.45 | 2.38 | 0.50 | 3.23 | 1.95 | 1.08 | 3.50 | 2.42 | 0.03 |
| nsp3 | aa1573-1587 | SAMVRMYIFFASFYY | -0.15 | 0.97 | 0.05 | -0.32 | -0.61 | 0.18 | 0.95 | 4.02 | 1.46 | 0.09 | 3.07 | 1.45 | 0.02 |
| nsp3 | aa1585-1599 | FYYVWKSYVHVVDGC | -0.29 | -0.11 | 0.55 | -0.16 | -0.55 | 0.43 | 0.41 | 2.33 | -0.01 | 3.88 | 0.73 | 1.69 | 0.03 |
| nsp3 | aa1631-1645 | VYANGGKGFCKLHNW | -0.39 | -0.17 | -0.35 | -0.28 | -0.72 | 1.21 | 0.38 | 3.42 | 0.96 | -0.30 | 3.73 | 1.39 | 0.05 |
| nsp3 | aa1637-1651 | KGFCKLHNWNCVNCD | -0.59 | -0.15 | 0.01 | -0.59 | -0.56 | -0.23 | 0.27 | 3.00 | 0.81 | 4.14 | 0.22 | 0.87 | 0.01 |
| nsp3 | aa1639-1653 | FCKLHNWNCVNCDTF | -0.76 | 0.39 | -0.50 | -0.58 | -0.57 | -0.40 | 0.58 | 3.16 | 0.31 | 1.69 | 2.11 | 2.54 | 0.01 |
| nsp3 | aa1653-1667 | FCAGSTFISDEVARD | 0.38 | -0.28 | -0.11 | -0.33 | -0.45 | 0.73 | 0.00 | 1.49 | 1.47 | 1.00 | 0.80 | 3.01 | 0.01 |
| nsp3 | aa1687-1701 | DSVTVKNGSIHLYFD | 0.16 | 0.76 | 0.83 | 0.27 | -0.72 | 0.37 | 3.16 | 4.31 | 2.87 | 0.54 | 3.12 | 0.88 | 0.01 |
| nsp3 | aa1697-1711 | HLYFDKAGQKTYERH | -0.77 | -0.35 | -0.09 | -0.70 | -0.78 | -0.21 | 0.05 | 4.29 | 1.55 | -0.46 | 2.60 | 2.36 | 0.02 |
| nsp3 | aa1699-1713 | YFDKAGQKTYERHSL | -0.68 | -0.63 | -0.64 | -0.50 | -0.85 | -0.33 | -0.33 | 3.47 | 0.84 | -0.74 | 1.84 | 1.29 | 0.05 |
| nsp3 | aa1709-1723 | ERHSLSHFVNLDNLR | -0.66 | 0.24 | -0.12 | -0.38 | -0.39 | -0.31 | 0.69 | 4.00 | 0.43 | -0.59 | 3.25 | 1.34 | 0.05 |
| nsp3 | aa1711-1725 | HSLSHFVNLDNLRAN | -0.84 | -0.70 | -0.77 | -0.79 | -0.38 | -0.45 | 0.04 | 3.37 | -0.28 | -0.83 | 1.51 | 1.39 | 0.05 |
| nsp3 | aa1859-1873 | EVTGDSCNNYMLTYN | -0.70 | -0.04 | 0.06 | -0.43 | -0.81 | 0.58 | 0.23 | 2.18 | 0.61 | -0.05 | 3.28 | 1.70 | 0.03 |
| nsp3 | aa1875-1889 | VENMTPRDLGACIDC | 0.89 | 1.26 | 1.45 | 0.75 | 0.40 | 1.53 | 1.50 | 3.29 | 1.98 | 3.74 | 1.54 | 2.49 | 0.01 |
| nsp4 | aa5-19 | NWLKQLIKVTLVFLF | -0.81 | -0.74 | -0.54 | -0.72 | -0.80 | -0.05 | -0.47 | 3.17 | 0.36 | -0.24 | 0.22 | 0.11 | 0.01 |
| nsp4 | aa9-23 | QLIKVTLVFLFVAAI | -0.68 | -0.80 | -0.77 | -0.85 | -0.86 | -0.41 | -0.63 | 1.54 | 0.05 | -0.61 | 3.66 | -0.67 | 0.02 |
| nsp4 | aa11-25 | IKVTLVFLFVAAIFY | -0.45 | -0.54 | -0.29 | -0.70 | -0.73 | 0.01 | -0.20 | 3.11 | 1.31 | -0.29 | 1.21 | 0.31 | 0.01 |
| nsp4 | aa61-75 | DTCFANKHADFDTWF | 0.60 | 2.36 | 1.50 | 0.82 | -0.39 | 1.63 | 2.50 | 5.11 | 3.03 | 1.28 | 4.86 | 3.27 | 0.02 |
| nsp4 | aa107-121 | PGTILRTTNGDFLHF | -0.70 | 0.79 | 0.09 | -0.49 | -0.75 | -0.14 | 1.49 | 3.84 | 1.88 | 0.82 | 4.13 | 1.35 | 0.01 |
| nsp4 | aa117-131 | DFLHFLPRVFSAVGN | -0.77 | -0.73 | -0.52 | -0.86 | -0.86 | 0.07 | 0.66 | 5.60 | 0.46 | -0.33 | 0.45 | -0.05 | 0.01 |
| nsp4 | aa131-145 | NICYTPSKLIEYTDF | 0.38 | 0.39 | 1.46 | 0.12 | -0.32 | 0.96 | 1.05 | 3.61 | 1.60 | 0.56 | 2.61 | 1.46 | 0.03 |
| nsp4 | aa137-151 | SKLIEYTDFATSACV | -0.56 | -0.11 | 0.87 | 0.61 | -0.45 | -0.43 | 1.06 | 6.74 | 1.39 | 0.70 | 0.00 | -0.02 | 0.05 |
| nsp4 | aa145-159 | FATSACVLAAECTIF | 0.26 | 0.87 | 0.23 | 1.28 | -0.38 | -0.15 | 1.53 | 3.53 | 1.78 | 0.64 | 1.24 | 0.72 | 0.05 |
| nsp4 | aa157-171 | TIFKDASGKPVPYCY | -0.17 | -0.08 | 0.17 | -0.56 | -0.40 | 0.45 | -0.01 | 3.14 | 1.77 | 0.64 | 2.10 | 1.47 | 0.01 |
| nsp4 | aa177-191 | EGSVAYESLRPDTRY | -0.19 | 0.45 | 0.98 | 0.28 | -0.29 | 1.58 | 1.70 | 3.58 | 1.51 | 1.25 | 3.52 | 2.49 | 0.01 |
| nsp4 | aa207-221 | EGSVRVVTTFDSEYC | 1.99 | 2.60 | 1.87 | 1.37 | 1.09 | 1.77 | 2.02 | 4.71 | 3.72 | 3.20 | 3.10 | 2.80 | 0.01 |
| nsp4 | aa209-223 | SVRVVTTFDSEYCRH | -0.20 | 0.11 | 0.21 | -0.39 | -0.77 | -0.41 | 1.25 | 3.32 | 1.32 | 2.12 | 3.57 | 0.57 | 0.01 |
| nsp4 | aa227-241 | ERSEAGVCVSTSGRW | 0.10 | 0.34 | 0.50 | 0.09 | 0.36 | 2.40 | 0.59 | 3.19 | 2.57 | 0.28 | 3.32 | 2.45 | 0.05 |
| nsp4 | aa233-247 | VCVSTSGRWVLNNDY | 0.15 | 1.01 | 0.67 | -0.46 | -0.38 | -0.28 | 0.90 | 3.88 | 1.05 | 0.50 | 2.57 | 2.16 | 0.02 |
| nsp4 | aa235-249 | VSTSGRWVLNNDYYR | 0.41 | 1.29 | 0.46 | -0.04 | -0.05 | 0.88 | 1.21 | 4.10 | 2.25 | 0.81 | 4.23 | 2.16 | 0.02 |
| nsp4 | aa241-255 | WVLNNDYYRSLPGVF | -0.41 | -0.15 | -0.15 | -0.34 | -0.39 | 1.38 | 0.75 | 3.33 | 1.04 | -0.03 | 3.30 | 1.54 | 0.02 |
| nsp4 | aa297-311 | LAYYFMRFRRAFGEY | 0.65 | 1.44 | 1.06 | 0.40 | 0.32 | 1.57 | 1.69 | 3.97 | 2.20 | 1.62 | 3.77 | 3.19 | 0.01 |
| nsp4 | aa299-313 | YYFMRFRRAFGEYSH | 0.21 | 1.13 | 0.37 | -0.28 | -0.71 | 0.26 | 1.21 | 3.90 | 1.34 | 0.19 | 4.11 | 1.84 | 0.03 |
| nsp4 | aa309-323 | GEYSHVVAFNTLLFL | -0.75 | 1.03 | -0.52 | -0.81 | -0.77 | -0.23 | 0.34 | 3.02 | 1.10 | -0.32 | 0.86 | 0.56 | 0.05 |
| nsp4 | aa351-365 | FYLTNDVSFLAHIQW | -0.32 | 0.72 | 0.62 | -0.49 | -0.73 | 0.13 | 0.99 | 4.12 | 2.20 | 0.64 | 4.40 | 1.99 | 0.01 |
| nsp4 | aa355-369 | NDVSFLAHIQWMVMF | -0.25 | 0.47 | 0.50 | -0.12 | -0.42 | 1.44 | 0.78 | 3.62 | 1.58 | 0.87 | 3.16 | 2.94 | 0.01 |
| nsp4 | aa361-375 | AHIQWMVMFTPLVPF | -0.72 | 0.29 | -0.28 | -0.78 | -0.80 | 1.28 | 0.94 | 3.19 | 1.06 | 0.04 | 2.77 | 1.85 | 0.03 |
| nsp4 | aa363-377 | IQWMVMFTPLVPFWI | -0.46 | 0.69 | 0.55 | -0.46 | -0.57 | 1.76 | 0.93 | 3.33 | 1.62 | 0.31 | 2.41 | 2.10 | 0.05 |
| nsp4 | aa409-423 | FSTFEEAALCTFLLN | -0.55 | -0.37 | -0.65 | -0.44 | -0.52 | 0.85 | -0.41 | 3.24 | 1.28 | 0.18 | 1.09 | 0.15 | 0.03 |
| nsp4 | aa473-487 | KALNDFSNSGSDVLY | 0.06 | 0.99 | 0.46 | -0.39 | -0.43 | 0.80 | 1.27 | 3.07 | 2.12 | 0.79 | 3.37 | 1.24 | 0.01 |
| nsp5 | aa19-33 | QVTCGTTTLNGLWLD | 0.46 | 0.46 | 0.44 | 0.15 | 0.18 | 2.20 | 1.09 | 3.18 | 2.43 | 0.75 | 2.61 | 0.79 | 0.02 |
| nsp5 | aa23-37 | GTTTLNGLWLDDVVY | 1.31 | 1.59 | 2.20 | 0.89 | 0.33 | 2.39 | 1.97 | 4.31 | 3.15 | 1.67 | 3.85 | 2.29 | 0.05 |
| nsp5 | aa27-41 | LNGLWLDDVVYCPRH | -0.46 | -0.57 | -0.20 | -0.72 | -0.79 | 0.17 | -0.04 | 3.65 | 2.08 | -0.56 | 3.59 | 0.70 | 0.03 |
| nsp5 | aa41-55 | HVICTSEDMLNPNYE | -0.46 | 0.18 | -0.31 | -0.65 | -0.72 | 0.32 | 0.13 | 3.46 | 1.76 | 0.96 | 0.20 | 1.10 | 0.02 |
| nsp5 | aa45-59 | TSEDMLNPNYEDLLI | 0.54 | 1.39 | 0.96 | 0.92 | 0.19 | 1.63 | 1.51 | 3.89 | 2.82 | 1.76 | 3.79 | 3.61 | 0.01 |
| nsp5 | aa141-155 | LNGSCGSVGFNIDYD | 1.56 | 1.64 | 1.49 | -0.13 | 0.24 | 0.79 | 2.24 | 4.67 | 2.39 | 1.85 | 2.94 | 3.42 | 0.01 |
| nsp5 | aa143-157 | GSCGSVGFNIDYDCV | 0.93 | 1.46 | 1.74 | 0.07 | -0.06 | 1.83 | 1.27 | 4.20 | 2.38 | 2.55 | 2.62 | 2.54 | 0.02 |
| nsp5 | aa145-159 | CGSVGFNIDYDCVSF | 1.11 | 1.65 | 1.64 | 0.73 | -0.06 | 0.73 | 2.12 | 4.64 | 2.98 | 1.49 | 3.77 | 2.65 | 0.01 |
| nsp5 | aa147-161 | SVGFNIDYDCVSFCY | 2.13 | 2.60 | 2.65 | 1.66 | 0.95 | 1.30 | 2.32 | 5.02 | 4.02 | 2.90 | 4.09 | 2.94 | 0.01 |
| nsp5 | aa167-181 | LPTGVHAGTDLEGNF | -0.75 | -0.32 | -0.19 | -0.73 | -0.79 | 0.90 | 0.20 | 3.26 | 2.23 | 0.00 | 1.06 | 2.13 | 0.01 |
| nsp5 | aa169-183 | TGVHAGTDLEGNFYG | -0.71 | -0.32 | 0.94 | -0.73 | -0.73 | 0.53 | 1.10 | 3.27 | 2.90 | 0.53 | 1.85 | 1.35 | 0.01 |
| nsp5 | aa171-185 | VHAGTDLEGNFYGPF | -0.71 | 1.11 | 0.06 | -0.01 | -0.77 | 0.71 | 1.74 | 3.89 | 2.95 | 1.27 | 3.43 | 2.85 | 0.01 |
| nsp5 | aa205-219 | LAWLYAAVINGDRWF | 0.07 | 1.74 | 1.04 | 0.46 | -0.02 | 2.25 | 2.10 | 4.73 | 3.03 | 1.32 | 4.81 | 2.52 | 0.02 |
| nsp5 | aa209-223 | YAAVINGDRWFLNRF | -0.61 | 0.82 | 0.04 | -0.20 | -0.52 | 1.12 | 1.68 | 3.38 | 2.00 | 0.59 | 4.01 | 2.13 | 0.01 |
| nsp5 | aa277-291 | NGRTILGSALLEDEF | 0.62 | 1.02 | 1.02 | 0.30 | 0.51 | 1.77 | 1.20 | 3.96 | 1.85 | 1.63 | 2.35 | 2.70 | 0.01 |
| nsp5 | aa283-297 | GSALLEDEFTPFDVV | 0.39 | 0.27 | 0.70 | -0.63 | -0.27 | 1.30 | -0.14 | 3.33 | 1.97 | 1.14 | 1.36 | 2.46 | 0.05 |
| nsp6 | aa21-35 | SLLVLVQSTQWSLFF | -0.57 | -0.18 | 0.08 | -0.45 | -0.69 | 0.96 | 0.72 | 3.51 | 1.20 | 0.17 | 2.31 | 0.89 | 0.02 |
| nsp6 | aa25-39 | LVQSTQWSLFFFLYE | 1.39 | 1.73 | 1.63 | 1.61 | 0.53 | 1.90 | 1.89 | 4.07 | 2.55 | 2.05 | 2.79 | 1.53 | 0.05 |
| nsp6 | aa31-45 | WSLFFFLYENAFLPF | -0.53 | 1.35 | 0.69 | 0.05 | -0.50 | -0.23 | 1.88 | 3.90 | 0.74 | 0.89 | 3.57 | 1.73 | 0.01 |
| nsp6 | aa45-59 | FAMGIIAMSAFAMMF | -0.18 | -0.11 | 0.09 | 0.13 | -0.05 | 2.56 | 0.37 | 2.23 | 1.56 | 0.54 | 1.71 | 3.80 | 0.05 |
| nsp6 | aa123-137 | LILMTARTVYDDGAR | -0.59 | 0.68 | 0.31 | -0.08 | -0.36 | 1.92 | 0.47 | 3.01 | 2.39 | 0.26 | 2.04 | 2.52 | 0.05 |
| nsp6 | aa151-165 | KVYYGNALDQAISMW | 0.27 | 0.73 | 0.70 | 0.34 | -0.10 | 1.80 | 0.85 | 3.59 | 2.84 | 0.29 | 3.38 | 2.66 | 0.05 |
| nsp6 | aa155-169 | GNALDQAISMWALII | -0.65 | 0.20 | -0.36 | -0.37 | -0.74 | 0.58 | 0.51 | 3.09 | 1.35 | 0.24 | 0.26 | 1.08 | 0.02 |
| nsp6 | aa183-197 | MFLARGIVFMCVEYC | 1.50 | 2.55 | 2.01 | 1.43 | 0.63 | 1.63 | 2.09 | 4.50 | 3.21 | 3.01 | 3.17 | 2.46 | 0.01 |
| nsp6 | aa187-201 | RGIVFMCVEYCPIFF | -0.39 | 1.38 | 0.68 | 0.60 | -0.24 | 1.30 | 2.13 | 4.41 | 2.03 | 1.50 | 3.55 | 1.08 | 0.01 |
| nsp6 | aa191-205 | FMCVEYCPIFFITGN | -0.40 | -0.62 | -0.41 | -0.74 | -0.78 | -0.27 | -0.64 | 4.91 | 0.04 | -0.35 | 0.21 | -0.22 | 0.05 |
| nsp6 | aa207-221 | LQCIMLVYCFLGYFC | 0.17 | 0.47 | 0.70 | -0.12 | -0.35 | 1.16 | 0.42 | 3.18 | 1.49 | 1.68 | 2.64 | 1.24 | 0.02 |
| nsp6 | aa211-225 | MLVYCFLGYFCTCYF | 0.27 | 0.89 | 0.86 | -0.22 | -0.40 | -0.39 | 0.97 | 3.82 | 0.71 | 0.67 | 2.31 | 1.62 | 0.03 |
| nsp6 | aa215-229 | CFLGYFCTCYFGLFC | -0.05 | 0.63 | 0.74 | -0.13 | -0.10 | 0.87 | 0.68 | 3.13 | 0.48 | 1.39 | 2.02 | 2.00 | 0.05 |
| nsp6 | aa221-235 | CTCYFGLFCLLNRYF | -0.71 | 0.34 | -0.23 | -0.29 | -0.52 | -0.43 | 0.59 | 3.28 | 0.48 | -0.34 | 2.74 | 1.46 | 0.02 |
| nsp6 | aa229-243 | CLLNRYFRLTLGVYD | 0.85 | 1.43 | 0.91 | -0.22 | -0.47 | 0.60 | 2.16 | 4.04 | 2.22 | 0.90 | 1.70 | 0.76 | 0.05 |
| nsp6 | aa237-251 | LTLGVYDYLVSTQEF | 0.58 | 1.12 | 1.20 | -0.47 | -0.36 | 0.65 | 1.28 | 3.56 | 2.19 | 0.89 | 2.70 | 2.34 | 0.01 |
| nsp6 | aa239-253 | LGVYDYLVSTQEFRY | 0.01 | 1.64 | 1.50 | 0.09 | 0.07 | 1.58 | 2.18 | 4.68 | 2.24 | 1.38 | 4.15 | 3.30 | 0.02 |
| nsp7 | aa27-41 | KLWAQCVQLHNDILL | -0.79 | -0.07 | -0.01 | -0.75 | -0.79 | -0.51 | -0.08 | 3.06 | 0.06 | -0.35 | 1.26 | 0.44 | 0.03 |
| nsp8 | aa15-29 | ATAQEAYEQAVANGD | -0.57 | 0.26 | -0.37 | -0.54 | -0.83 | -0.10 | 0.68 | 3.30 | 0.58 | -0.20 | 0.67 | 0.30 | 0.01 |
| nsp8 | aa99-113 | NDALNNIINNARDGC | -0.45 | -0.19 | 0.70 | -0.22 | -0.46 | 0.32 | -0.30 | 0.92 | 1.24 | 3.15 | 1.61 | 1.69 | 0.03 |
| nsp8 | aa139-153 | NTCDGTTFTYASALW | -0.32 | 0.27 | 0.32 | -0.28 | -0.21 | 2.01 | 0.70 | 3.38 | 2.64 | 0.29 | 2.58 | 1.59 | 0.03 |
| nsp8 | aa141-155 | CDGTTFTYASALWEI | 0.27 | 0.94 | 1.59 | 0.58 | 0.30 | 1.56 | 1.40 | 4.04 | 2.98 | 0.97 | 2.79 | 2.25 | 0.03 |
| nsp8 | aa163-177 | SKIVQLSEISMDNSP | -0.74 | -0.64 | -0.75 | -0.81 | -0.80 | -0.26 | -0.60 | 5.37 | -0.53 | -0.44 | -0.41 | -0.57 | 0.05 |
| nsp9 | aa17-31 | TTQTACTDDNALAYY | 0.20 | 1.46 | 0.75 | -0.11 | -0.57 | -0.28 | 1.49 | 4.23 | 2.62 | 1.50 | 0.99 | 1.12 | 0.01 |
| nsp9 | aa41-55 | LALLSDLQDLKWARF | -0.79 | 0.25 | -0.57 | -0.67 | -0.45 | 1.60 | 0.52 | 3.40 | 1.22 | -0.27 | 3.29 | 2.33 | 0.03 |
| nsp10 | aa17-31 | CAFAVDAAKAYKDYL | -0.28 | -0.50 | -0.72 | -0.80 | -0.70 | -0.37 | -0.47 | 3.10 | -0.10 | -0.67 | -0.01 | 1.21 | 0.05 |
| nsp10 | aa77-91 | CRCHIDHPNPKGFCD | -0.34 | 0.07 | 0.37 | 0.03 | -0.51 | 1.27 | 1.14 | 3.60 | 1.49 | 2.18 | 2.09 | 2.11 | 0.01 |
| nsp12 | aa25-39 | TGTSTDVVYRAFDIY | 0.73 | 0.30 | 0.96 | -0.46 | 0.10 | 0.43 | 0.66 | 3.23 | 2.10 | 0.86 | 3.12 | 2.54 | 0.02 |
| nsp12 | aa27-41 | TSTDVVYRAFDIYND | 0.95 | 1.72 | 0.88 | 0.13 | 0.04 | 0.14 | 1.33 | 3.87 | 2.68 | 1.87 | 2.85 | 1.96 | 0.01 |
| nsp12 | aa57-71 | FQEKDEDDNLIDSYF | 1.54 | 2.51 | 1.97 | 1.05 | 0.58 | 1.75 | 2.34 | 5.09 | 3.76 | 2.23 | 3.32 | 2.51 | 0.02 |
| nsp12 | aa89-103 | NLLKDCPAVAKHDFF | -0.52 | -0.10 | -0.15 | -0.13 | 0.13 | -0.53 | 1.09 | 3.57 | 1.36 | -0.45 | 2.47 | 1.58 | 0.03 |
| nsp12 | aa121-135 | TKYTMADLVYALRHF | -0.84 | -0.25 | -0.62 | -0.83 | -0.85 | -0.48 | 0.27 | 2.79 | -0.02 | -0.11 | 3.17 | 0.85 | 0.01 |
| nsp12 | aa123-137 | YTMADLVYALRHFDE | 0.04 | 0.30 | 1.16 | 0.11 | -0.71 | -0.13 | 0.85 | 3.13 | 1.22 | 1.01 | 1.64 | 2.16 | 0.01 |
| nsp12 | aa125-139 | MADLVYALRHFDEGN | -0.57 | -0.53 | -0.26 | -0.27 | -0.74 | -0.36 | 0.07 | 5.88 | -0.28 | 0.75 | 0.23 | 2.53 | 0.01 |
| nsp12 | aa127-141 | DLVYALRHFDEGNCD | 0.45 | 0.26 | 1.36 | 0.52 | -0.17 | 0.67 | 1.21 | 4.83 | 1.77 | 3.73 | 0.47 | 2.90 | 0.03 |
| nsp12 | aa145-159 | EILVTYNCCDDDYFN | 2.11 | 2.56 | 2.27 | 2.07 | 0.86 | 1.38 | 2.06 | 4.77 | 3.69 | 3.13 | 3.03 | 3.29 | 0.03 |
| nsp12 | aa149-163 | TYNCCDDDYFNKKDW | 1.25 | 1.14 | 0.47 | 1.03 | 0.02 | 2.76 | 1.81 | 4.37 | 2.42 | 1.11 | 3.09 | 2.83 | 0.05 |
| nsp12 | aa151-165 | NCCDDDYFNKKDWYD | 2.52 | 2.51 | 2.18 | 1.79 | 1.39 | 3.14 | 3.00 | 5.47 | 3.45 | 2.88 | 3.95 | 4.23 | 0.01 |
| nsp12 | aa153-167 | CDDDYFNKKDWYDFV | 1.18 | 1.70 | 1.51 | 1.00 | 1.34 | 2.50 | 2.02 | 4.87 | 2.54 | 1.85 | 4.10 | 4.34 | 0.01 |
| nsp12 | aa155-169 | DDYFNKKDWYDFVEN | 1.11 | 1.47 | 0.81 | 0.64 | 0.02 | 1.95 | 1.38 | 3.02 | 3.36 | 1.02 | 3.17 | 2.44 | 0.05 |
| nsp12 | aa203-217 | VGVLTLDNQDLNGNW | 0.34 | 1.07 | 0.31 | -0.12 | -0.08 | 1.85 | 1.29 | 3.85 | 2.49 | 0.22 | 2.98 | 2.88 | 0.05 |
| nsp12 | aa205-219 | VLTLDNQDLNGNWYD | 2.03 | 2.59 | 2.26 | 0.82 | 0.91 | 2.72 | 3.05 | 4.89 | 3.48 | 2.27 | 3.82 | 3.08 | 0.01 |
| nsp12 | aa209-223 | DNQDLNGNWYDFGDF | 1.92 | 2.92 | 2.50 | 1.57 | 1.00 | 2.37 | 2.95 | 5.08 | 3.57 | 2.10 | 4.51 | 3.53 | 0.02 |
| nsp12 | aa211-225 | QDLNGNWYDFGDFIQ | 0.10 | 1.35 | 0.90 | -0.02 | -0.20 | 0.78 | 1.39 | 3.63 | 3.21 | 0.57 | 2.99 | 1.59 | 0.02 |
| nsp12 | aa225-239 | QTTPGSGVPVVDSYY | -0.10 | 1.67 | 1.16 | -0.17 | -0.31 | -0.24 | 1.75 | 3.25 | 2.95 | 1.83 | 2.23 | 1.37 | 0.01 |
| nsp12 | aa273-287 | KYDFTEERLKLFDRY | -0.27 | 0.74 | 0.72 | -0.05 | 0.02 | 0.95 | 1.11 | 3.79 | 1.10 | -0.02 | 3.96 | 3.20 | 0.03 |
| nsp12 | aa275-289 | DFTEERLKLFDRYFK | -0.33 | 0.12 | -0.44 | 0.01 | -0.70 | 0.19 | 0.39 | 3.32 | 1.17 | -0.03 | 1.65 | 0.39 | 0.02 |
| nsp12 | aa293-307 | QTYHPNCVNCLDDRC | -0.28 | 0.03 | 0.40 | -0.34 | -0.09 | 0.56 | 0.49 | 3.33 | 2.37 | 2.86 | 2.31 | 1.92 | 0.01 |
| nsp12 | aa295-309 | YHPNCVNCLDDRCIL | -0.29 | 0.27 | 0.28 | -0.28 | -0.67 | 0.42 | 0.98 | 3.72 | 2.45 | 0.83 | 2.82 | 1.59 | 0.01 |
| nsp12 | aa357-371 | NQDVNLHSSRLSFKE | -0.63 | -0.66 | -0.72 | -0.77 | -0.83 | -0.36 | -0.33 | 0.11 | 0.05 | -0.66 | -0.15 | 3.73 | 0.02 |
| nsp12 | aa361-375 | NLHSSRLSFKELLVY | -0.56 | 0.55 | -0.38 | -0.56 | 0.01 | 0.10 | 0.96 | 3.49 | 1.40 | -0.13 | 1.78 | 1.04 | 0.02 |
| nsp12 | aa401-415 | ALTNNVAFQTVKPGN | -0.79 | -0.74 | -0.79 | -0.86 | -0.82 | -0.43 | -0.78 | 4.97 | -0.56 | -0.67 | 0.79 | -0.46 | 0.05 |
| nsp12 | aa409-423 | QTVKPGNFNKDFYDF | 0.72 | 1.81 | 2.31 | 0.16 | -0.14 | 0.36 | 1.60 | 4.51 | 2.17 | 0.76 | 3.74 | 2.75 | 0.05 |
| nsp12 | aa429-443 | FFKEGSSVELKHFFF | -0.80 | -0.05 | -0.71 | -0.73 | -0.63 | -0.33 | -0.25 | 3.50 | -0.22 | 0.00 | 3.47 | 1.51 | 0.01 |
| nsp12 | aa441-455 | FFFAQDGNAAISDYD | 1.61 | 1.92 | 1.64 | 0.63 | 0.59 | -0.08 | 2.47 | 4.52 | 3.26 | 2.05 | 2.19 | 3.53 | 0.01 |
| nsp12 | aa443-457 | FAQDGNAAISDYDYY | 2.53 | 3.74 | 3.19 | 2.18 | 1.65 | 2.05 | 3.00 | 6.12 | 4.35 | 3.59 | 5.04 | 3.90 | 0.02 |
| nsp12 | aa445-459 | QDGNAAISDYDYYRY | 1.82 | 2.99 | 2.28 | 1.40 | 0.17 | 1.65 | 2.73 | 5.53 | 3.65 | 2.35 | 5.15 | 2.51 | 0.02 |
| nsp12 | aa447-461 | GNAAISDYDYYRYNL | -0.39 | 0.59 | 0.50 | -0.13 | -0.58 | 0.04 | 0.81 | 3.38 | 1.39 | 0.29 | 3.82 | 1.34 | 0.01 |
| nsp12 | aa453-467 | DYDYYRYNLPTMCDI | 0.10 | 0.77 | 0.81 | -0.29 | 0.25 | 0.26 | 0.46 | 3.17 | 1.48 | 1.48 | 1.59 | 2.53 | 0.01 |
| nsp12 | aa471-485 | LFVVEVVDKYFDCYD | 3.07 | 3.53 | 3.17 | 2.19 | 1.69 | 2.51 | 2.96 | 6.15 | 4.59 | 3.36 | 4.15 | 3.25 | 0.05 |
| nsp12 | aa475-489 | EVVDKYFDCYDGGCI | 1.56 | 1.76 | 2.38 | 0.85 | 0.25 | 1.58 | 1.63 | 4.21 | 3.41 | 3.04 | 3.01 | 1.70 | 0.03 |
| nsp12 | aa493-507 | QVIVNNLDKSAGFPF | -0.27 | 0.26 | -0.42 | -0.68 | -0.77 | 0.20 | 1.08 | 3.05 | 0.84 | -0.19 | 2.15 | 1.53 | 0.01 |
| nsp12 | aa509-523 | KWGKARLYYDSMSYE | 0.67 | 1.23 | 1.26 | 0.60 | -0.03 | 2.51 | 1.63 | 3.95 | 2.42 | 1.70 | 2.69 | 2.49 | 0.03 |
| nsp12 | aa513-527 | ARLYYDSMSYEDQDA | 0.40 | 0.68 | 0.82 | 0.14 | -0.60 | 1.22 | 1.87 | 2.11 | 1.42 | 1.05 | 0.23 | 3.67 | 0.05 |
| nsp12 | aa515-529 | LYYDSMSYEDQDALF | 0.97 | 1.80 | 1.80 | 1.24 | 0.20 | 1.97 | 2.40 | 4.88 | 2.73 | 1.46 | 3.59 | 3.75 | 0.02 |
| nsp12 | aa517-531 | YDSMSYEDQDALFAY | 0.83 | 1.54 | 1.85 | 1.35 | 0.24 | 1.57 | 2.42 | 4.16 | 2.75 | 1.90 | 3.60 | 4.13 | 0.01 |
| nsp12 | aa605-619 | TVYSDVENPHLMGWD | 0.48 | 0.51 | 0.58 | -0.12 | -0.01 | 2.08 | 1.99 | 3.69 | 3.05 | 1.13 | 2.98 | 2.55 | 0.01 |
| nsp12 | aa607-621 | YSDVENPHLMGWDYP | -0.10 | 1.05 | 1.17 | 0.04 | 0.48 | 2.05 | 1.94 | 4.04 | 2.76 | 1.28 | 4.58 | 3.46 | 0.01 |
| nsp12 | aa661-675 | AQVLSEMVMCGGSLY | -0.15 | 0.50 | 0.32 | -0.36 | -0.36 | 1.34 | 1.15 | 3.61 | 1.73 | 0.96 | 2.38 | 1.68 | 0.01 |
| nsp12 | aa719-733 | KYVRNLQHRLYECLY | 0.38 | 2.01 | 1.48 | 0.44 | -0.05 | 0.48 | 1.83 | 4.46 | 1.82 | 1.47 | 3.97 | 2.82 | 0.03 |
| nsp12 | aa723-737 | NLQHRLYECLYRNRD | -0.44 | 0.46 | -0.01 | -0.65 | -0.23 | 0.37 | 0.62 | 3.57 | 0.23 | 0.73 | 3.47 | 1.24 | 0.01 |
| nsp12 | aa727-741 | RLYECLYRNRDVDTD | -0.29 | -0.66 | -0.55 | -0.76 | -0.63 | 0.09 | -0.22 | 0.76 | -0.40 | -0.51 | 0.57 | 3.34 | 0.05 |
| nsp12 | aa729-743 | YECLYRNRDVDTDFV | 0.20 | 0.77 | 0.19 | -0.38 | 0.32 | 0.32 | 0.78 | 3.47 | 0.58 | 0.35 | 2.52 | 2.76 | 0.01 |
| nsp12 | aa731-745 | CLYRNRDVDTDFVNE | -0.10 | -0.29 | -0.55 | -0.65 | -0.69 | -0.33 | 0.54 | 0.86 | 0.84 | 0.41 | 0.23 | 3.07 | 0.01 |
| nsp12 | aa733-747 | YRNRDVDTDFVNEFY | 1.56 | 2.24 | 2.37 | 1.02 | 0.83 | 1.85 | 2.67 | 4.52 | 3.55 | 2.62 | 3.66 | 4.02 | 0.01 |
| nsp12 | aa735-749 | NRDVDTDFVNEFYAY | 1.52 | 2.12 | 1.90 | 0.99 | 0.81 | 1.50 | 2.37 | 4.23 | 2.67 | 1.67 | 3.54 | 3.26 | 0.01 |
| nsp12 | aa753-767 | HFSMMILSDDAVVCF | 0.31 | 1.23 | 0.54 | 1.05 | -0.66 | 0.17 | 1.58 | 3.93 | 2.13 | 0.55 | 2.46 | 1.61 | 0.01 |
| nsp12 | aa817-831 | HTMLVKQGDDYVYLP | -0.43 | 0.10 | -0.79 | -0.58 | -0.82 | 0.00 | 0.29 | 4.05 | 0.63 | -0.38 | 2.02 | 0.23 | 0.01 |
| nsp12 | aa819-833 | MLVKQGDDYVYLPYP | 0.04 | 1.08 | 0.31 | -0.43 | -0.37 | 0.29 | 1.42 | 3.54 | 1.75 | 0.68 | 3.14 | 1.43 | 0.01 |
| nsp12 | aa823-837 | QGDDYVYLPYPDPSR | -0.08 | 0.82 | 0.33 | -0.23 | -0.09 | 1.73 | 1.12 | 3.54 | 2.31 | 0.24 | 4.07 | 2.40 | 0.03 |
| nsp12 | aa835-849 | PSRILGAGCFVDDIV | 0.19 | 1.32 | 0.98 | -0.20 | 0.27 | -0.15 | 0.48 | 3.04 | 2.55 | 1.11 | 1.68 | 1.77 | 0.02 |
| nsp12 | aa869-883 | PLTKHPNQEYADVFH | -0.54 | 0.53 | -0.54 | -0.60 | -0.75 | -0.03 | 1.37 | 3.13 | 0.62 | -0.58 | 2.57 | 1.47 | 0.03 |
| nsp12 | aa871-885 | TKHPNQEYADVFHLY | -0.61 | 0.43 | -0.44 | -0.40 | -0.80 | 0.01 | 1.32 | 4.03 | 1.37 | -0.34 | 3.63 | 2.01 | 0.01 |
| nsp12 | aa873-887 | HPNQEYADVFHLYLQ | -0.24 | -0.61 | -0.59 | -0.79 | -0.81 | -0.39 | -0.07 | 3.75 | 0.68 | -0.63 | 2.41 | 1.41 | 0.03 |
| nsp12 | aa875-889 | NQEYADVFHLYLQYI | 0.22 | 0.74 | 1.26 | -0.38 | -0.60 | -0.36 | 0.29 | 3.08 | 1.59 | 0.68 | 2.26 | 0.91 | 0.05 |
| nsp12 | aa911-925 | DNTSRYWEPEFYEAM | 0.61 | 1.45 | 1.32 | 0.69 | 0.63 | 2.83 | 1.81 | 3.94 | 2.59 | 1.51 | 3.19 | 3.26 | 0.02 |
| nsp12 | aa915-929 | RYWEPEFYEAMYTPH | 1.93 | 1.47 | 1.04 | -0.06 | -0.67 | 1.89 | 2.07 | 5.02 | 2.39 | -0.04 | 4.66 | 3.45 | 0.03 |
| nsp13 | aa19-33 | ACIRRPFLCCKCCYD | 0.93 | 0.64 | 0.65 | 0.65 | 0.21 | 0.70 | 1.43 | 3.80 | 1.62 | 1.76 | 2.35 | 1.36 | 0.01 |
| nsp13 | aa51-65 | CNAPGCDVTDVTQLY | -0.66 | 0.06 | 0.62 | -0.76 | -0.71 | -0.41 | -0.04 | 3.11 | 0.04 | 0.70 | 3.00 | 0.97 | 0.03 |
| nsp13 | aa57-71 | DVTDVTQLYLGGMSY | -0.63 | 0.32 | 0.09 | -0.58 | -0.44 | 1.39 | -0.43 | 3.52 | 1.53 | 0.26 | 2.45 | 2.18 | 0.05 |
| nsp13 | aa101-115 | SDNVTDFNAIATCDW | 0.36 | 1.17 | 1.10 | 0.92 | 0.42 | 1.74 | 1.25 | 4.26 | 2.04 | 1.11 | 2.30 | 2.80 | 0.02 |
| nsp13 | aa107-121 | FNAIATCDWTNAGDY | -0.38 | 0.84 | 0.45 | 0.39 | 0.04 | 0.68 | 0.82 | 3.70 | 1.66 | 0.14 | 1.50 | 1.95 | 0.05 |
| nsp13 | aa109-123 | AIATCDWTNAGDYIL | -0.53 | 1.16 | 0.10 | 0.07 | -0.66 | 0.11 | 0.74 | 4.06 | 2.32 | 0.06 | 1.61 | 1.38 | 0.05 |
| nsp13 | aa197-211 | GEYTFEKGDYGDAVV | 0.72 | 0.88 | 0.74 | -0.31 | -0.60 | 1.27 | 0.44 | 3.14 | 3.30 | 1.08 | 1.32 | 2.23 | 0.05 |
| nsp13 | aa241-255 | LVPQEHYVRITGLYP | -0.75 | -0.05 | -0.49 | -0.58 | -0.84 | 0.17 | 0.20 | 2.28 | 1.23 | -0.54 | 3.18 | 0.23 | 0.02 |
| nsp13 | aa313-327 | AAVDALCEKALKYLP | 0.07 | 0.92 | -0.54 | 0.09 | -0.79 | -0.33 | 0.55 | 3.13 | 0.91 | 0.34 | 1.70 | 0.09 | 0.03 |
| nsp13 | aa409-423 | PRTLLTKGTLEPEYF | 1.25 | 1.49 | 1.04 | 0.10 | 0.09 | 1.03 | 1.34 | 4.15 | 1.88 | 1.34 | 3.11 | 2.88 | 0.01 |
| nsp13 | aa493-507 | QIGVVREFLTRNPAW | -0.40 | 0.16 | -0.06 | -0.10 | -0.31 | 1.34 | 0.89 | 3.70 | 1.84 | -0.04 | 3.02 | 2.04 | 0.02 |
| nsp13 | aa531-545 | TQTVDSSQGSEYDYV | 0.96 | 1.94 | 1.39 | 0.80 | 0.06 | 0.53 | 1.92 | 4.40 | 2.05 | 1.71 | 2.21 | 2.30 | 0.01 |
| nsp13 | aa569-583 | AKVGILCIMSDRDLY | 0.05 | 1.48 | 0.78 | 0.39 | 0.00 | 1.37 | 1.28 | 4.15 | 2.86 | 0.53 | 3.75 | 3.15 | 0.05 |
| nsp13 | aa579-593 | DRDLYDKLQFTSLEI | 0.35 | 0.60 | 0.47 | -0.26 | -0.46 | 0.33 | 1.12 | 4.63 | 1.56 | 1.11 | 2.17 | 1.59 | 0.01 |
| nsp14 | aa59-73 | GFKMNYQVNGYPNMF | -0.60 | 0.31 | 0.04 | -0.70 | -0.74 | 0.30 | 0.49 | 3.05 | 0.50 | -0.15 | 3.27 | 1.89 | 0.02 |
| nsp14 | aa81-95 | RHVRAWIGFDVEGCH | -0.36 | 0.03 | 0.21 | -0.54 | -0.83 | -0.35 | 0.14 | 3.37 | 1.99 | 2.02 | 2.72 | 1.53 | 0.01 |
| nsp14 | aa145-159 | QFKHLIPLMYKGLPW | -0.26 | 0.66 | -0.06 | 0.08 | -0.17 | 1.93 | 1.19 | 3.82 | 2.19 | 0.62 | 3.89 | 1.73 | 0.03 |
| nsp14 | aa213-227 | RATCFSTASDTYACW | 0.13 | 0.26 | 0.35 | -0.16 | -0.02 | 1.38 | 0.21 | 3.39 | 1.61 | 1.04 | 1.78 | 2.00 | 0.03 |
| nsp14 | aa221-235 | SDTYACWHHSIGFDY | 0.11 | 1.59 | 1.66 | 0.19 | 0.74 | 1.07 | 1.70 | 4.59 | 2.29 | 1.64 | 4.24 | 3.13 | 0.01 |
| nsp14 | aa223-237 | TYACWHHSIGFDYVY | 0.48 | 1.96 | 1.60 | 0.59 | 0.03 | 1.34 | 2.05 | 4.94 | 2.59 | 1.91 | 4.66 | 2.14 | 0.01 |
| nsp14 | aa225-239 | ACWHHSIGFDYVYNP | -0.64 | 0.27 | -0.31 | -0.83 | -0.85 | -0.63 | -0.32 | 2.08 | 0.54 | -0.18 | 4.15 | 0.16 | 0.03 |
| nsp14 | aa227-241 | WHHSIGFDYVYNPFM | -0.65 | 0.08 | -0.28 | -0.51 | -0.74 | 0.47 | 0.35 | 3.49 | 1.45 | 0.06 | 4.43 | 2.00 | 0.02 |
| nsp14 | aa229-243 | HSIGFDYVYNPFMID | 0.01 | 0.34 | -0.03 | -0.29 | -0.62 | 0.31 | 0.77 | 3.30 | 1.86 | 0.32 | 1.49 | 2.68 | 0.01 |
| nsp14 | aa233-247 | FDYVYNPFMIDVQQW | 0.10 | 1.50 | 0.90 | 0.32 | -0.11 | 2.16 | 1.49 | 4.52 | 3.00 | 0.96 | 4.22 | 3.46 | 0.03 |
| nsp14 | aa235-249 | YVYNPFMIDVQQWGF | -0.28 | 1.38 | 0.16 | 0.16 | -0.25 | 2.17 | 1.21 | 3.65 | 2.55 | 0.69 | 3.55 | 2.58 | 0.03 |
| nsp14 | aa247-261 | WGFTGNLQSNHDLYC | 1.00 | 1.23 | 1.12 | 0.52 | -0.29 | 1.24 | 1.46 | 3.75 | 2.63 | 2.15 | 1.81 | 1.56 | 0.01 |
| nsp14 | aa273-287 | DAIMTRCLAVHECFV | -0.59 | -0.07 | -0.03 | 0.69 | -0.69 | -0.44 | 1.27 | 3.43 | 1.37 | 1.35 | 1.81 | 0.34 | 0.01 |
| nsp14 | aa283-297 | HECFVKRVDWTIEYP | 0.61 | 0.82 | 0.49 | 0.76 | -0.39 | 0.42 | 1.41 | 4.62 | 1.85 | 0.67 | 1.83 | 2.02 | 0.01 |
| nsp14 | aa355-369 | PCSDKAYKIEELFYS | -0.01 | 0.89 | 0.42 | 0.16 | 0.07 | 1.06 | 0.92 | 3.09 | 0.91 | 0.87 | 2.04 | 1.32 | 0.03 |
| nsp14 | aa373-387 | HSDKFTDGVCLFWNC | 0.14 | 0.15 | -0.03 | -0.09 | -0.32 | 0.45 | 0.24 | 4.23 | 1.06 | 1.55 | 2.80 | 1.81 | 0.01 |
| nsp14 | aa405-419 | VLSNLNLPGCDGGSL | -0.50 | -0.34 | -0.06 | -0.22 | -0.29 | 1.53 | 0.59 | 3.05 | 2.17 | 1.36 | 1.34 | 0.38 | 0.03 |
| nsp14 | aa407-421 | SNLNLPGCDGGSLYV | -0.49 | 0.31 | 0.31 | 0.60 | -0.32 | 1.86 | 1.09 | 3.67 | 2.02 | 1.82 | 1.73 | 0.88 | 0.03 |
| nsp14 | aa431-445 | FDKSAFVNLKQLPFF | -0.73 | -0.50 | -0.33 | -0.72 | -0.79 | -0.72 | 0.90 | 3.49 | -0.10 | -0.41 | 3.44 | 2.33 | 0.01 |
| nsp14 | aa433-447 | KSAFVNLKQLPFFYY | -0.16 | 1.11 | -0.07 | -0.32 | -0.68 | -0.48 | 1.02 | 4.20 | 1.51 | 0.68 | 3.36 | 2.02 | 0.01 |
| nsp14 | aa451-465 | PCESHGKQVVSDIDY | 0.84 | 0.58 | 0.81 | -0.23 | -0.27 | 0.11 | 0.95 | 3.89 | 1.89 | 0.94 | 1.78 | 2.61 | 0.01 |
| nsp14 | aa479-493 | LGGAVCRHHANEYRL | -0.83 | 0.24 | -0.68 | -0.79 | -0.77 | -0.15 | 0.40 | 3.08 | -0.29 | 0.19 | 3.01 | 1.88 | 0.02 |
| nsp14 | aa481-495 | GAVCRHHANEYRLYL | -0.81 | 0.45 | -0.01 | -0.74 | -0.67 | 0.07 | 0.94 | 3.62 | 0.88 | 0.34 | 3.83 | 1.70 | 0.01 |
| nsp14 | aa485-499 | RHHANEYRLYLDAYN | -0.56 | 0.62 | 0.19 | -0.80 | -0.84 | -0.44 | 0.06 | 3.78 | 0.47 | 0.65 | 4.55 | 2.65 | 0.02 |
| nsp14 | aa495-509 | LDAYNMMISAGFSLW | -0.01 | 0.71 | 0.20 | 0.02 | 0.06 | 2.73 | 0.70 | 3.73 | 2.49 | 0.30 | 3.58 | 3.12 | 0.05 |
| nsp14 | aa505-519 | GFSLWVYKQFDTYNL | -0.17 | -0.12 | -0.61 | -0.63 | -0.81 | -0.68 | -0.05 | 3.55 | 0.29 | -0.19 | 2.14 | -0.04 | 0.01 |
| nsp14 | aa509-523 | WVYKQFDTYNLWNTF | -0.53 | -0.18 | -0.60 | -0.77 | -0.78 | 0.16 | 0.71 | 2.51 | 0.78 | -0.35 | 3.25 | 0.81 | 0.01 |
| nsp15 | aa15-29 | HFDGQQGEVPVSIIN | 0.13 | -0.05 | -0.54 | -0.44 | -0.84 | -0.42 | -0.33 | 3.10 | 0.31 | 1.08 | -0.01 | 0.27 | 0.02 |
| nsp15 | aa31-45 | TVYTKVDGVDVELFE | -0.04 | 1.33 | 0.94 | 0.95 | 1.01 | 2.37 | 1.33 | 3.74 | 2.17 | 1.79 | 1.50 | 2.88 | 0.03 |
| nsp15 | aa45-59 | ENKTTLPVNVAFELW | 0.03 | 0.97 | 0.99 | 0.33 | 0.35 | 2.60 | 1.14 | 3.77 | 2.35 | 0.47 | 3.55 | 3.06 | 0.05 |
| nsp15 | aa75-89 | NLGVDIAANTVIWDY | 0.94 | 1.80 | 1.60 | 0.69 | 0.80 | 1.62 | 1.54 | 3.77 | 2.14 | 1.64 | 3.11 | 3.12 | 0.03 |
| nsp15 | aa121-135 | TVFFDGRVDGQVDLF | 0.46 | 1.26 | 1.11 | -0.01 | -0.13 | 1.63 | 1.86 | 4.30 | 2.76 | 0.37 | 3.74 | 3.14 | 0.03 |
| nsp15 | aa123-137 | FFDGRVDGQVDLFRN | -0.44 | 0.57 | 0.07 | -0.30 | -0.75 | 1.18 | 0.82 | 3.48 | 2.12 | -0.05 | 3.54 | 1.92 | 0.03 |
| nsp15 | aa201-215 | LQEFKPRSQMEIDFL | -0.26 | 0.38 | 0.26 | -0.60 | -0.77 | 0.12 | -0.17 | 2.96 | 0.54 | 3.03 | 1.05 | 1.80 | 0.02 |
| nsp15 | aa209-223 | QMEIDFLELAMDEFI | 0.84 | 2.15 | 1.30 | 0.81 | 0.37 | 2.06 | 1.75 | 3.74 | 2.10 | 1.96 | 2.19 | 3.22 | 0.05 |
| nsp15 | aa211-225 | EIDFLELAMDEFIER | 1.34 | 1.45 | 1.45 | 0.69 | 0.51 | 2.38 | 1.59 | 4.06 | 2.00 | 1.77 | 3.00 | 3.29 | 0.02 |
| nsp15 | aa217-231 | LAMDEFIERYKLEGY | 0.34 | 0.85 | 1.12 | 0.08 | -0.19 | 1.67 | 0.93 | 3.81 | 2.01 | 1.40 | 2.79 | 2.31 | 0.02 |
| nsp15 | aa219-233 | MDEFIERYKLEGYAF | -0.23 | 1.07 | 0.92 | 0.05 | 0.01 | 1.32 | 1.40 | 3.92 | 1.98 | 0.58 | 3.32 | 2.64 | 0.02 |
| nsp15 | aa221-235 | EFIERYKLEGYAFEH | 0.17 | 0.42 | 1.25 | -0.39 | -0.59 | 0.26 | 1.15 | 3.88 | 1.32 | 1.02 | 2.83 | 2.86 | 0.01 |
| nsp15 | aa227-241 | KLEGYAFEHIVYGDF | 0.41 | 1.83 | 1.81 | 0.12 | 0.56 | 0.63 | 1.56 | 4.54 | 2.91 | 1.66 | 3.61 | 2.32 | 0.03 |
| nsp15 | aa229-243 | EGYAFEHIVYGDFSH | -0.30 | 0.35 | 1.22 | -0.60 | -0.68 | -0.08 | 1.48 | 3.34 | 1.66 | 1.56 | 3.82 | 1.43 | 0.01 |
| nsp15 | aa233-247 | FEHIVYGDFSHSQLG | -0.82 | -0.61 | -0.72 | -0.86 | -0.85 | -0.33 | -0.05 | 3.31 | 0.16 | 0.64 | 1.85 | 0.09 | 0.01 |
| nsp15 | aa235-249 | HIVYGDFSHSQLGGL | -0.66 | 0.02 | -0.28 | -0.82 | -0.77 | -0.18 | -0.11 | 3.75 | 1.64 | -0.27 | 2.55 | 0.20 | 0.02 |
| nsp15 | aa237-251 | VYGDFSHSQLGGLHL | -0.78 | 0.15 | -0.45 | -0.85 | -0.88 | -0.39 | 0.13 | 3.42 | 1.47 | -0.43 | 2.78 | 0.68 | 0.02 |
| nsp15 | aa255-269 | LAKRFKESPFELEDF | 1.17 | 1.49 | 1.53 | 0.96 | 0.96 | 2.03 | 1.81 | 3.59 | 2.16 | 1.84 | 3.25 | 2.38 | 0.01 |
| nsp15 | aa287-301 | GSSKCVCSVIDLLLD | 0.44 | 1.08 | 0.50 | 0.83 | 0.14 | 0.21 | 0.97 | 4.07 | 1.82 | 0.89 | 0.93 | 1.91 | 0.02 |
| nsp15 | aa289-303 | SKCVCSVIDLLLDDF | 0.99 | 1.85 | 1.65 | 1.85 | 1.11 | 1.31 | 2.27 | 5.14 | 2.35 | 1.39 | 2.77 | 3.42 | 0.02 |
| nsp15 | aa291-305 | CVCSVIDLLLDDFVE | 1.56 | 1.98 | 1.96 | 2.08 | 1.32 | 2.66 | 2.31 | 5.22 | 2.96 | 2.03 | 2.34 | 3.49 | 0.03 |
| nsp15 | aa293-307 | CSVIDLLLDDFVEII | 2.31 | 3.29 | 2.66 | 2.61 | 1.98 | 2.75 | 2.44 | 5.69 | 3.88 | 2.78 | 3.30 | 3.83 | 0.05 |
| nsp15 | aa319-333 | VKVTIDYTEISFMLW | 0.73 | 1.48 | 1.02 | 0.83 | 0.17 | 2.26 | 1.17 | 4.10 | 2.56 | 1.21 | 3.41 | 2.66 | 0.03 |
| nsp15 | aa329-343 | SFMLWCKDGHVETFY | -0.10 | 1.14 | 0.92 | -0.62 | -0.49 | 1.06 | 1.42 | 3.79 | 1.75 | 0.81 | 3.45 | 1.52 | 0.02 |
| nsp16 | aa57-71 | TLTLAVPYNMRVIHF | -0.68 | -0.69 | 0.08 | -0.74 | -0.61 | 0.00 | -0.36 | 3.14 | 1.43 | -0.62 | 1.92 | 0.46 | 0.05 |
| nsp16 | aa91-105 | PTGTLLVDSDLNDFV | 0.43 | 0.51 | 0.15 | 0.62 | -0.37 | -0.52 | 1.73 | 3.37 | 0.83 | 1.02 | 2.48 | 2.84 | 0.01 |
| nsp16 | aa175-189 | HSWNADLYKLMGHFA | -0.56 | -0.61 | -0.29 | -0.32 | -0.80 | 0.82 | -0.19 | 3.42 | 1.22 | 0.31 | 1.87 | 1.74 | 0.01 |
| nsp16 | aa177-191 | WNADLYKLMGHFAWW | 0.22 | 0.71 | 0.88 | 0.35 | 0.04 | 2.88 | 2.11 | 4.72 | 2.93 | 0.95 | 4.24 | 3.56 | 0.01 |
| nsp16 | aa209-223 | GCNYLGKPREQIDGY | 0.08 | 0.78 | 0.45 | -0.01 | -0.27 | 0.78 | 0.96 | 3.48 | 1.47 | 0.65 | 2.64 | 2.86 | 0.01 |
| nsp16 | aa215-229 | KPREQIDGYVMHANY | -0.40 | 0.38 | 0.08 | -0.79 | -0.82 | 0.46 | 0.34 | 3.17 | 1.89 | 0.01 | 2.80 | 2.32 | 0.05 |
| nsp16 | aa217-231 | REQIDGYVMHANYIF | -0.25 | 0.55 | 0.18 | -0.37 | -0.77 | -0.08 | 0.50 | 3.28 | 1.33 | 0.83 | 3.54 | 1.54 | 0.01 |
| nsp16 | aa219-233 | QIDGYVMHANYIFWR | -0.26 | 1.15 | 0.48 | -0.15 | -0.10 | 1.49 | 0.84 | 3.65 | 1.96 | 0.77 | 4.16 | 1.74 | 0.03 |
| nsp16 | aa237-251 | PIQLSSYSLFDMSKF | -0.52 | 0.92 | -0.13 | -0.21 | -0.23 | 0.86 | 1.23 | 3.22 | 1.35 | 0.14 | 2.06 | 0.99 | 0.01 |
| Spike Protein | aa47-61 | KVFRSSVLHSTQDLF | -0.74 | 0.46 | -0.68 | -0.75 | -0.82 | -0.69 | 0.33 | 3.06 | -0.37 | -0.74 | 1.53 | 1.44 | 0.05 |
| Spike Protein | aa51-65 | SSVLHSTQDLFLPFF | -0.59 | 1.00 | 0.76 | 0.03 | -0.72 | 0.45 | 2.47 | 3.87 | 1.87 | 1.04 | 4.11 | 2.06 | 0.01 |
| Spike Protein | aa57-71 | TQDLFLPFFSNVTWF | -0.14 | 1.08 | 0.53 | 0.16 | -0.42 | 1.37 | 1.54 | 3.25 | 2.39 | 0.52 | 3.71 | 1.59 | 0.02 |
| Spike Protein | aa75-89 | HVSGTNGTKRFDNPV | -0.59 | -0.71 | -0.67 | -0.82 | -0.85 | 0.18 | 1.02 | 3.32 | -0.38 | -0.14 | 0.39 | -0.29 | 0.02 |
| Spike Protein | aa83-97 | KRFDNPVLPFNDGVY | -0.08 | 1.51 | 0.81 | -0.19 | -0.57 | 1.13 | 1.35 | 3.52 | 1.11 | 0.86 | 3.37 | 1.91 | 0.05 |
| Spike Protein | aa85-99 | FDNPVLPFNDGVYFA | -0.62 | 0.56 | 0.22 | -0.27 | -0.63 | 0.23 | 0.99 | 3.17 | 0.43 | 0.06 | 2.18 | 1.04 | 0.03 |
| Spike Protein | aa125-139 | IVNNATNVVIKVCEF | -0.31 | 0.18 | 0.13 | 0.12 | -0.47 | -0.59 | 0.75 | 3.44 | 0.18 | 0.32 | 0.62 | 0.67 | 0.01 |
| Spike Protein | aa127-141 | NNATNVVIKVCEFQF | -0.58 | 0.83 | -0.13 | -0.63 | -0.59 | -0.03 | 0.43 | 3.43 | 0.66 | -0.24 | 2.30 | 1.12 | 0.05 |
| Spike Protein | aa133-147 | VIKVCEFQFCNDPFL | 0.06 | 1.54 | 1.17 | 0.13 | -0.46 | -0.04 | 0.82 | 4.13 | 1.97 | 1.48 | 3.62 | 0.79 | 0.05 |
| Spike Protein | aa137-151 | CEFQFCNDPFLGVYY | 1.79 | 2.66 | 2.59 | 1.32 | 0.88 | 1.29 | 2.78 | 5.32 | 4.12 | 2.75 | 4.62 | 3.66 | 0.01 |
| Spike Protein | aa149-163 | VYYHKNNKSWMESEF | 0.37 | 0.38 | 0.75 | 0.16 | -0.10 | 1.81 | 0.85 | 3.50 | 2.23 | 0.69 | 3.49 | 3.26 | 0.02 |
| Spike Protein | aa151-165 | YHKNNKSWMESEFRV | 0.17 | -0.53 | 0.02 | 0.15 | -0.42 | 1.70 | 0.40 | 3.15 | 1.39 | 0.17 | 2.65 | 2.27 | 0.03 |
| Spike Protein | aa163-177 | FRVYSSANNCTFEYV | -0.10 | 1.47 | 0.56 | 0.98 | -0.23 | 0.08 | 1.87 | 3.42 | 1.30 | 1.13 | 1.88 | 1.14 | 0.02 |
| Spike Protein | aa177-191 | VSQPFLMDLEGKQGN | -0.57 | -0.57 | -0.64 | -0.71 | -0.84 | -0.45 | 0.17 | 5.23 | -0.28 | -0.59 | -0.29 | 0.85 | 0.02 |
| Spike Protein | aa193-207 | KNLREFVFKNIDGYF | 0.24 | 1.60 | 0.59 | 0.68 | -0.42 | 1.41 | 2.01 | 4.28 | 2.07 | 1.09 | 4.11 | 2.39 | 0.01 |
| Spike Protein | aa195-209 | LREFVFKNIDGYFKI | 0.43 | -0.14 | -0.48 | 0.41 | -0.67 | 0.60 | 0.92 | 3.57 | 1.24 | -0.48 | 2.50 | 0.93 | 0.05 |
| Spike Protein | aa261-275 | SSGWTAGAAAYYVGY | -0.41 | 0.43 | 0.11 | -0.68 | -0.44 | 0.59 | 0.32 | 3.20 | 0.47 | 0.40 | 1.52 | 0.96 | 0.05 |
| Spike Protein | aa335-349 | FPNITNLCPFGEVFN | -0.62 | 0.66 | 0.31 | -0.60 | -0.80 | -0.31 | 0.70 | 3.09 | 0.91 | 0.07 | 2.11 | 0.38 | 0.02 |
| Spike Protein | aa339-353 | TNLCPFGEVFNATRF | -0.56 | -0.06 | -0.11 | -0.57 | -0.78 | 0.34 | 1.23 | 3.16 | 1.03 | -0.08 | 3.55 | 0.71 | 0.01 |
| Spike Protein | aa345-359 | GEVFNATRFASVYAW | -0.36 | -0.04 | 0.08 | -0.42 | -0.29 | 1.71 | 0.55 | 2.84 | 1.29 | -0.11 | 3.17 | 2.14 | 0.05 |
| Spike Protein | aa361-375 | RKRISNCVADYSVLY | -0.28 | -0.06 | -0.29 | -0.60 | -0.45 | -0.13 | 0.19 | 3.25 | 0.87 | -0.30 | 2.95 | 0.82 | 0.03 |
| Spike Protein | aa393-407 | LNDLCFTNVYADSFV | -0.32 | 0.80 | 0.82 | -0.30 | -0.61 | 0.20 | 1.17 | 3.69 | 0.45 | 1.28 | 1.54 | 0.34 | 0.03 |
| Spike Protein | aa415-429 | QIAPGQTGKIADYNY | -0.14 | 0.33 | 0.45 | -0.60 | -0.63 | -0.24 | 0.37 | 3.71 | 2.35 | 0.14 | 2.62 | 1.29 | 0.02 |
| Spike Protein | aa421-435 | TGKIADYNYKLPDDF | -0.43 | 0.51 | 0.28 | -0.32 | 0.06 | 0.38 | 0.74 | 3.52 | 0.69 | -0.15 | 2.14 | 2.40 | 0.03 |
| Spike Protein | aa441-455 | AWNSNNLDSKVGGNY | -0.66 | -0.40 | -0.72 | -0.38 | -0.68 | -0.19 | 1.06 | 3.23 | 0.43 | -0.58 | 1.89 | 1.40 | 0.02 |
| Spike Protein | aa443-457 | NSNNLDSKVGGNYNY | 0.02 | 0.66 | -0.21 | -0.33 | -0.71 | -0.11 | 2.88 | 3.87 | 0.98 | 0.02 | 2.58 | 2.31 | 0.01 |
| Spike Protein | aa445-459 | NNLDSKVGGNYNYLY | 0.03 | 1.04 | 0.34 | -0.17 | -0.28 | 0.17 | 1.07 | 3.97 | 1.74 | 0.62 | 3.25 | 2.25 | 0.01 |
| Spike Protein | aa459-473 | YRLFRKSNLKPFERD | -0.52 | -0.37 | -0.33 | -0.29 | -0.29 | 1.79 | -0.25 | 3.04 | 0.95 | -0.04 | 2.60 | 2.28 | 0.02 |
| Spike Protein | aa465-479 | SNLKPFERDISTEIY | 0.68 | 1.63 | 0.47 | 0.15 | 0.18 | 1.18 | 1.57 | 4.35 | 1.88 | 1.17 | 2.82 | 2.35 | 0.02 |
| Spike Protein | aa481-495 | AGSTPCNGVEGFNCY | 0.34 | 0.97 | 1.46 | 0.07 | -0.26 | 0.49 | 1.31 | 3.29 | 2.16 | 1.67 | 3.15 | 1.84 | 0.01 |
| Spike Protein | aa483-497 | STPCNGVEGFNCYFP | 0.02 | 0.11 | 0.59 | -0.36 | -0.69 | 0.11 | 0.74 | 2.87 | 0.90 | 0.78 | 3.37 | 0.66 | 0.01 |
| Spike Protein | aa487-501 | NGVEGFNCYFPLQSY | 0.48 | 0.46 | 0.41 | -0.47 | -0.55 | -0.26 | 0.68 | 3.44 | 1.30 | 0.73 | 2.55 | 2.12 | 0.01 |
| Spike Protein | aa509-523 | VGYQPYRVVVLSFEL | 0.16 | 1.14 | 1.15 | 0.26 | 0.13 | 0.82 | 1.41 | 3.48 | 1.42 | 1.21 | 1.08 | 1.10 | 0.03 |
| Spike Protein | aa511-525 | YQPYRVVVLSFELLH | -0.42 | 0.84 | 0.70 | -0.25 | -0.84 | 0.14 | 0.80 | 3.56 | 1.03 | 0.61 | 2.40 | 0.47 | 0.05 |
| Spike Protein | aa653-667 | AGCLIGAEHVNNSYE | 0.15 | 0.29 | 0.28 | -0.58 | -0.76 | -0.45 | 1.01 | 3.17 | 2.35 | 1.24 | 0.08 | 1.34 | 0.02 |
| Spike Protein | aa655-669 | CLIGAEHVNNSYECD | 1.29 | 1.88 | 1.81 | 0.72 | 0.26 | 1.18 | 1.84 | 5.33 | 2.79 | 3.52 | 1.55 | 3.05 | 0.02 |
| Spike Protein | aa657-671 | IGAEHVNNSYECDIP | -0.09 | 0.44 | 0.75 | -0.44 | -0.54 | 0.04 | 0.72 | 3.52 | 1.90 | 1.07 | 1.19 | 2.79 | 0.01 |
| Spike Protein | aa741-755 | SVDCTMYICGDSTEC | 1.27 | 0.72 | 1.25 | 0.88 | 0.45 | 1.36 | 1.22 | 3.03 | 1.40 | 2.63 | 1.53 | 2.06 | 0.02 |
| Spike Protein | aa811-825 | ILPDPSKPSKRSFIE | -0.12 | -0.64 | 0.54 | -0.46 | -0.66 | 1.47 | 2.77 | 3.17 | 1.52 | 0.16 | 3.70 | 1.37 | 0.02 |
| Spike Protein | aa815-829 | PSKPSKRSFIEDLLF | -0.02 | 0.19 | 1.10 | 0.54 | -0.46 | 2.94 | 1.74 | 4.16 | 3.02 | 0.78 | 3.32 | 2.40 | 0.03 |
| Spike Protein | aa833-847 | TLADAGFIKQYGDCL | 0.05 | 0.67 | -0.08 | 0.23 | -0.10 | 1.71 | 0.88 | 3.18 | 1.88 | 4.09 | 2.07 | 1.29 | 0.01 |
| Spike Protein | aa835-849 | ADAGFIKQYGDCLGD | 0.22 | 0.70 | 0.45 | 0.10 | -0.02 | 1.42 | 1.43 | 3.45 | 2.43 | 1.49 | 1.56 | 0.98 | 0.01 |
| Spike Protein | aa843-857 | YGDCLGDIAARDLIC | 0.19 | 1.08 | 1.84 | 0.74 | 0.24 | 1.74 | 1.44 | 3.56 | 2.45 | 3.06 | 2.70 | 2.40 | 0.01 |
| Spike Protein | aa899-913 | ALQIPFAMQMAYRFN | -0.78 | -0.32 | -0.61 | -0.22 | -0.81 | 1.03 | -0.14 | 3.91 | 1.66 | -0.48 | 2.44 | 1.30 | 0.03 |
| Spike Protein | aa909-923 | AYRFNGIGVTQNVLY | -0.51 | 0.29 | -0.41 | -0.83 | -0.86 | -0.52 | -0.17 | 1.95 | 0.10 | 0.05 | 3.88 | 0.74 | 0.02 |
| Spike Protein | aa919-933 | QNVLYENQKLIANQF | -0.74 | 0.02 | -0.67 | -0.76 | -0.81 | -0.21 | -0.55 | 2.01 | 1.47 | -0.20 | 3.02 | 0.26 | 0.02 |
| Spike Protein | aa1087-1101 | ICHDGKAHFPREGVF | 0.08 | 0.95 | -0.35 | 0.07 | -0.76 | 1.72 | 1.46 | 4.02 | 2.40 | 0.33 | 3.70 | 2.60 | 0.02 |
| Spike Protein | aa1095-1109 | FPREGVFVSNGTHWF | -0.44 | 1.00 | -0.12 | -0.09 | -0.78 | 1.22 | 1.97 | 3.86 | 1.85 | 0.45 | 4.17 | 2.19 | 0.01 |
| Spike Protein | aa1103-1117 | SNGTHWFVTQRNFYE | -0.57 | 0.53 | 0.01 | -0.21 | -0.65 | 0.44 | 1.89 | 3.32 | 1.19 | 0.97 | 1.97 | 1.40 | 0.01 |
| Spike Protein | aa1119-1133 | QIITTDNTFVSGNCD | -0.50 | -0.11 | -0.16 | -0.55 | -0.70 | -0.37 | -0.39 | 2.58 | 0.37 | 3.32 | 0.17 | 0.57 | 0.02 |
| Spike Protein | aa1145-1159 | DPLQPELDSFKEELD | 1.52 | 1.38 | 0.56 | 0.51 | 0.23 | 1.66 | 1.06 | 4.25 | 2.24 | 1.39 | 2.11 | 2.21 | 0.05 |
| Spike Protein | aa1159-1173 | DKYFKNHTSPDVDLG | -0.72 | -0.24 | -0.68 | -0.78 | -0.76 | -0.44 | 0.14 | 3.01 | 1.82 | 0.21 | 1.82 | 1.36 | 0.01 |
| Spike Protein | aa1161-1175 | YFKNHTSPDVDLGDI | -0.41 | 0.60 | 0.02 | -0.74 | -0.65 | -0.46 | 0.19 | 3.64 | 2.09 | 0.16 | 0.89 | 1.60 | 0.01 |
| Spike Protein | aa1177-1191 | GINASVVNIQKEIDR | -0.43 | -0.32 | -0.74 | -0.49 | -0.31 | 0.12 | -0.11 | 1.05 | 4.74 | -0.45 | 0.28 | 0.81 | 0.05 |
| Spike Protein | aa1201-1215 | ESLIDLQELGKYEQY | 1.18 | 1.87 | 1.33 | 0.41 | 0.17 | 2.20 | 1.69 | 4.13 | 3.16 | 1.35 | 3.47 | 2.55 | 0.03 |
| Spike Protein | aa1207-1221 | QELGKYEQYIKWPWY | 0.99 | 1.21 | 1.29 | 0.50 | -0.16 | 2.40 | 1.99 | 4.19 | 3.41 | 1.32 | 3.88 | 2.58 | 0.01 |
| Spike Protein | aa1209-1223 | LGKYEQYIKWPWYIW | 0.38 | 0.23 | 0.48 | 0.10 | -0.09 | 2.53 | 1.24 | 3.43 | 2.95 | 0.27 | 3.22 | 2.47 | 0.05 |
| Spike Protein | aa1253-1267 | CCSCGSCCKFDEDDS | 0.64 | 1.10 | 0.75 | 1.03 | 0.30 | 1.88 | 1.62 | 4.00 | 1.73 | 1.08 | 2.81 | 3.56 | 0.03 |
| Spike Protein | aa1255-1269 | SCGSCCKFDEDDSEP | 0.56 | 0.72 | 0.13 | 0.67 | -0.43 | 1.84 | 1.02 | 3.29 | 3.19 | 1.05 | 1.97 | 2.74 | 0.01 |
| GS Linker | aa13-27 | HYTGSGSGSGMDLFM | -0.31 | 0.04 | 0.27 | 0.00 | -0.41 | 2.07 | 0.01 | 3.44 | 2.53 | 0.57 | 3.64 | 2.96 | 0.03 |
| Orf3a Protein | aa97-111 | YSHLLLVAAGLEAPF | -0.32 | -0.13 | -0.51 | -0.69 | -0.76 | 0.78 | 0.12 | 2.53 | 1.23 | -0.47 | 3.48 | 2.08 | 0.03 |
| Orf3a Protein | aa99-113 | HLLLVAAGLEAPFLY | -0.30 | 1.30 | 0.66 | 0.11 | -0.45 | 0.23 | 1.49 | 3.87 | 1.66 | 0.65 | 4.37 | 2.10 | 0.01 |
| Orf3a Protein | aa101-115 | LLVAAGLEAPFLYLY | 0.26 | 1.80 | 1.18 | 0.70 | 0.01 | 0.45 | 1.58 | 3.77 | 2.90 | 1.15 | 3.82 | 2.27 | 0.02 |
| Orf3a Protein | aa137-151 | WKCRSKNPLLYDANY | -0.40 | 1.29 | 0.16 | -0.81 | -0.76 | -0.48 | 0.81 | 3.02 | 1.11 | 0.40 | 3.13 | 0.55 | 0.03 |
| Orf3a Protein | aa139-153 | CRSKNPLLYDANYFL | -0.56 | 0.66 | 0.03 | -0.61 | -0.73 | -0.59 | 0.67 | 3.04 | 4.51 | -0.12 | 3.11 | 0.71 | 0.01 |
| Orf3a Protein | aa141-155 | SKNPLLYDANYFLCW | -0.09 | 1.43 | 1.01 | 0.38 | 0.03 | 1.53 | 1.61 | 4.26 | 3.27 | 0.99 | 4.39 | 2.32 | 0.02 |
| Orf3a Protein | aa147-161 | YDANYFLCWHTNCYD | 1.23 | 2.34 | 1.70 | 1.07 | 0.97 | 0.03 | 2.84 | 4.47 | 3.09 | 2.74 | 3.31 | 3.28 | 0.01 |
| Orf3a Protein | aa149-163 | ANYFLCWHTNCYDYC | 1.10 | 2.50 | 2.45 | 1.43 | 1.62 | 0.97 | 2.47 | 4.52 | 3.42 | 3.19 | 3.77 | 3.70 | 0.01 |
| Orf3a Protein | aa151-165 | YFLCWHTNCYDYCIP | 0.27 | 1.63 | 1.49 | 0.69 | -0.51 | -0.43 | 1.99 | 3.83 | 2.25 | 2.21 | 3.36 | 1.45 | 0.01 |
| Orf3a Protein | aa153-167 | LCWHTNCYDYCIPYN | -0.46 | 0.80 | 0.13 | -0.70 | -0.74 | -0.32 | 1.37 | 3.70 | 1.11 | 0.99 | 3.95 | 2.13 | 0.01 |
| Orf3a Protein | aa171-185 | SSIVITSGDGTTSPI | -0.67 | -0.79 | -0.81 | -0.77 | -0.47 | -0.27 | 0.27 | 1.52 | 2.60 | 3.42 | -0.32 | -0.44 | 0.01 |
| Orf3a Protein | aa185-199 | ISEHDYQIGGYTEKW | 0.57 | 0.99 | 0.07 | 0.72 | -0.07 | 2.46 | 1.23 | 4.30 | 3.64 | 0.33 | 3.53 | 2.71 | 0.05 |
| Orf3a Protein | aa199-213 | WESGVKDCVVLHSYF | -0.62 | 1.18 | -0.10 | 0.53 | -0.71 | 0.13 | 1.55 | 3.80 | 1.85 | 0.18 | 3.25 | 2.43 | 0.01 |
| Orf3a Protein | aa203-217 | VKDCVVLHSYFTSDY | 0.43 | 1.80 | 0.55 | 0.44 | -0.26 | 0.31 | 1.24 | 4.53 | 1.78 | 0.87 | 1.82 | 2.87 | 0.02 |
| Orf3a Protein | aa205-219 | DCVVLHSYFTSDYYQ | 0.19 | 2.11 | 0.93 | 0.91 | -0.55 | 0.35 | 2.38 | 4.20 | 2.21 | 1.83 | 2.98 | 2.78 | 0.01 |
| Orf3a Protein | aa207-221 | VVLHSYFTSDYYQLY | 0.09 | 2.28 | 0.94 | 0.69 | -0.61 | 0.15 | 2.73 | 4.42 | 1.71 | 1.73 | 3.95 | 2.73 | 0.01 |
| Orf3a Protein | aa225-239 | LSTDTGVEHVTFFIY | -0.13 | 1.31 | 0.49 | 0.53 | -0.38 | -0.23 | 1.09 | 2.55 | 2.56 | 1.66 | 3.58 | 1.77 | 0.01 |
| Orf3a Protein | aa239-253 | YNKIVDEPEEHVQIH | -0.41 | -0.37 | -0.60 | -0.84 | -0.83 | -0.61 | -0.55 | 1.19 | 0.35 | -0.36 | 3.08 | 0.34 | 0.01 |
| Orf3a Protein | aa257-271 | GSSGVVNPVMEPIYD | 0.43 | 1.55 | 0.09 | -0.38 | -0.30 | 0.06 | 3.43 | 1.97 | 1.05 | 1.93 | 1.34 | 1.25 | 0.02 |
| Orf3a Protein | aa259-273 | SGVVNPVMEPIYDEP | 0.06 | 1.44 | -0.13 | -0.54 | -0.37 | 0.08 | 3.36 | 0.36 | 0.35 | 1.13 | 1.89 | 0.96 | 0.03 |
| Orf3a Protein | aa261-275 | VVNPVMEPIYDEPTT | 0.04 | 1.53 | -0.67 | -0.79 | -0.81 | -0.68 | 3.29 | 2.01 | 0.20 | 0.76 | 2.68 | -0.16 | 0.03 |
| Orf3a Protein | aa265-279 | VMEPIYDEPTTTTSV | -0.51 | 0.19 | -0.65 | -0.76 | -0.85 | -0.23 | 3.08 | 0.25 | -0.19 | -0.64 | 2.15 | 1.13 | 0.03 |
| Envelope Protein | aa9-23 | SFVSEETGTLIVNSV | -0.45 | -0.53 | -0.66 | -0.76 | -0.82 | -0.69 | -0.33 | 0.29 | 0.08 | 3.60 | -0.61 | -0.39 | 0.01 |
| Membrane Glycoprotein | aa23-37 | LEQWNLVIGFLFLTW | -0.56 | 0.41 | -0.20 | -0.52 | -0.44 | 0.74 | 0.78 | 3.10 | 1.15 | 1.18 | 2.50 | 1.83 | 0.01 |
| Membrane Glycoprotein | aa37-51 | WICLLQFAYANRNRF | -0.49 | -0.01 | -0.80 | -0.75 | -0.62 | -0.66 | 0.79 | 1.86 | -0.40 | -0.68 | 4.14 | 1.75 | 0.05 |
| Membrane Glycoprotein | aa39-53 | CLLQFAYANRNRFLY | -0.61 | 0.39 | -0.28 | -0.78 | -0.62 | -0.49 | 0.53 | 2.77 | 6.03 | -0.50 | 4.49 | 1.83 | 0.02 |
| Membrane Glycoprotein | aa95-109 | GLMWLSYFIASFRLF | -0.28 | -0.21 | -0.15 | -0.44 | -0.39 | 0.57 | -0.15 | 2.38 | 2.30 | -0.05 | 3.18 | 2.02 | 0.01 |
| Membrane Glycoprotein | aa171-185 | PKEITVATSRTLSYY | 0.00 | 1.60 | -0.28 | -0.72 | -0.05 | 0.90 | 0.13 | 2.05 | 3.13 | 0.12 | 1.74 | 1.17 | 0.05 |
| Membrane Glycoprotein | aa191-205 | QRVAGDSGFAAYSRY | -0.27 | 0.88 | 0.27 | -0.54 | -0.46 | 0.55 | 0.91 | 2.37 | 1.34 | 0.35 | 3.85 | 1.69 | 0.01 |
| Membrane Glycoprotein | aa207-221 | IGNYKLNTDHSSSSD | -0.50 | -0.82 | -0.77 | -0.82 | -0.83 | -0.31 | 3.53 | 1.59 | -0.14 | -0.56 | -0.19 | -0.38 | 0.02 |
| Membrane Glycoprotein | aa209-223 | NYKLNTDHSSSSDNI | -0.33 | -0.51 | -0.66 | -0.28 | -0.83 | -0.63 | 3.58 | 1.41 | 0.57 | -0.28 | 0.00 | -0.11 | 0.01 |
| Orf6 Protein | aa25-39 | IMRTFKVSIWNLDYI | -0.41 | 1.18 | 0.25 | 0.77 | -0.64 | -0.10 | 0.84 | 3.67 | 2.41 | 0.82 | 2.79 | 1.76 | 0.01 |
| Orf6 Protein | aa55-69 | KYSQLDEEQPMEIDG | 0.13 | -0.48 | -0.43 | -0.08 | -0.37 | -0.12 | 1.40 | -0.04 | 3.59 | 0.02 | 1.44 | 1.45 | 0.01 |
| Orf7a Protein | aa11-25 | ILFLALITLATCELY | 0.86 | 2.01 | 1.35 | 0.43 | 0.78 | 0.86 | 1.53 | 4.02 | 1.35 | 1.68 | 4.79 | 2.18 | 0.03 |
| Orf7a Protein | aa13-27 | FLALITLATCELYHY | 0.00 | 1.91 | 1.01 | 0.13 | -0.19 | -0.02 | 1.40 | 4.27 | 1.65 | 1.92 | 5.49 | 2.12 | 0.01 |
| Orf7a Protein | aa15-29 | ALITLATCELYHYQE | 0.02 | 0.10 | 0.29 | -0.33 | -0.26 | -0.34 | 0.74 | 2.57 | 0.46 | 1.28 | 4.31 | 1.31 | 0.01 |
| Orf7a Protein | aa17-31 | ITLATCELYHYQECV | 0.43 | 1.03 | 1.39 | 0.03 | -0.22 | 0.53 | 1.20 | 3.44 | 1.01 | 2.04 | 4.21 | 1.90 | 0.02 |
| Orf7a Protein | aa19-33 | LATCELYHYQECVRG | 1.19 | -0.43 | -0.23 | -0.32 | -0.34 | 1.41 | -0.03 | 1.95 | 1.36 | 0.48 | 3.64 | 1.53 | 0.05 |
| Orf7a Protein | aa41-55 | PCSSGTYEGNSPFHP | -0.19 | -0.68 | -0.52 | -0.81 | -0.82 | -0.63 | -0.57 | 2.24 | -0.35 | -0.61 | 3.31 | 0.06 | 0.05 |
| Orf8 Protein | aa33-47 | TQHQPYVVDDPCPIH | -0.44 | 0.34 | 0.11 | -0.66 | -0.54 | 0.04 | 0.21 | 3.07 | 0.36 | -0.11 | 3.78 | 1.79 | 0.03 |
| Orf8 Protein | aa35-49 | HQPYVVDDPCPIHFY | -0.15 | 1.46 | 1.44 | -0.32 | -0.63 | -0.15 | 1.92 | 4.54 | 1.85 | 1.37 | 4.71 | 2.55 | 0.01 |
| Orf8 Protein | aa39-53 | VVDDPCPIHFYSKWY | 0.47 | 1.05 | 1.08 | -0.09 | -0.55 | 1.01 | 1.23 | 3.68 | 2.23 | 0.69 | 3.63 | 1.92 | 0.02 |
| Orf8 Protein | aa69-83 | VDEAGSKSPIQYIDI | -0.38 | 1.27 | -0.16 | -0.49 | -0.25 | -0.11 | 0.36 | 3.68 | 0.28 | 1.66 | 4.43 | 1.44 | 0.01 |
| Orf8 Protein | aa71-85 | EAGSKSPIQYIDIGN | -0.75 | -0.10 | -0.35 | -0.64 | -0.82 | -0.04 | -0.34 | 4.70 | 0.47 | 1.15 | 2.72 | 0.25 | 0.01 |
| Orf8 Protein | aa75-89 | KSPIQYIDIGNYTVS | -0.85 | -0.46 | -0.86 | -0.54 | -0.85 | -0.52 | -0.08 | 2.30 | -0.62 | 1.10 | 3.12 | -0.38 | 0.02 |
| Orf8 Protein | aa77-91 | PIQYIDIGNYTVSCL | -0.69 | 1.57 | -0.30 | 0.03 | -0.80 | -0.29 | 1.22 | 2.79 | 0.33 | 2.51 | 3.26 | 0.15 | 0.02 |
| Orf8 Protein | aa101-115 | KLGSLVVRCSFYEDF | 0.20 | 1.55 | 1.31 | 1.24 | 1.10 | 0.50 | 2.29 | 4.51 | 2.57 | 0.66 | 3.84 | 3.37 | 0.03 |
| Orf8 Protein | aa103-117 | GSLVVRCSFYEDFLE | 1.42 | 2.71 | 1.88 | 1.63 | 1.27 | 1.74 | 1.85 | 4.37 | 3.44 | 2.37 | 3.14 | 3.64 | 0.02 |
| Orf8 Protein | aa105-119 | LVVRCSFYEDFLEYH | 1.67 | 2.80 | 2.14 | 2.04 | 0.60 | 1.12 | 2.49 | 5.44 | 4.31 | 2.43 | 4.45 | 4.11 | 0.01 |
| Orf8 Protein | aa107-121 | VRCSFYEDFLEYHDV | 1.63 | 2.38 | 2.11 | 1.61 | 0.13 | 0.45 | 1.84 | 5.26 | 4.27 | 2.36 | 3.77 | 3.22 | 0.02 |
| Orf8 Protein | aa109-123 | CSFYEDFLEYHDVRV | 0.31 | 1.48 | 0.68 | 0.29 | -0.48 | 0.72 | 1.62 | 4.35 | 3.00 | 1.36 | 3.72 | 2.91 | 0.01 |
| Orf8 Protein | aa111-125 | FYEDFLEYHDVRVVL | 0.21 | 1.71 | 1.04 | 1.04 | -0.04 | 1.16 | 1.45 | 4.62 | 3.06 | 0.96 | 4.03 | 3.06 | 0.05 |
| Nucleocapsid Phosphoprotein | aa79-93 | VPINTNSSPDDQIGY | -0.19 | 0.53 | 0.09 | -0.49 | -0.79 | -0.28 | 0.64 | 3.53 | 0.79 | 0.38 | 0.60 | 0.88 | 0.01 |
| Nucleocapsid Phosphoprotein | aa103-117 | GGDGKMKDLSPRWYF | -0.03 | 0.69 | 0.11 | 0.04 | 0.14 | 2.16 | 0.54 | 4.11 | 2.82 | 0.42 | 4.39 | 3.03 | 0.03 |
| Nucleocapsid Phosphoprotein | aa125-139 | EAGLPYGANKDGIIW | 0.16 | -0.12 | 0.36 | 0.01 | -0.19 | 1.57 | 0.98 | 2.73 | 2.26 | 0.05 | 3.09 | 2.20 | 0.03 |
| Nucleocapsid Phosphoprotein | aa165-179 | VLQLPQGTTLPKGFY | -0.78 | -0.48 | -0.67 | -0.75 | -0.69 | 1.30 | 5.47 | 1.29 | 3.16 | -0.13 | 4.99 | 0.73 | 0.02 |
| Nucleocapsid Phosphoprotein | aa167-181 | QLPQGTTLPKGFYAE | -0.43 | 0.14 | -0.54 | -0.39 | -0.54 | 1.57 | 5.51 | 0.36 | 3.07 | -0.12 | 4.07 | 0.63 | 0.03 |
| Nucleocapsid Phosphoprotein | aa169-183 | PQGTTLPKGFYAEGS | -0.35 | -0.81 | -0.54 | -0.58 | -0.52 | 1.48 | 5.19 | 0.78 | 3.53 | -0.26 | 3.28 | 0.13 | 0.02 |
| Nucleocapsid Phosphoprotein | aa209-223 | SRGTSPARMAGNGGD | -0.71 | -0.56 | -0.35 | -0.22 | -0.64 | 1.71 | 0.07 | 6.14 | 1.17 | 1.14 | -0.08 | -0.11 | 0.05 |
| Nucleocapsid Phosphoprotein | aa219-233 | GNGGDAALALLLLDR | -0.30 | 0.98 | 0.07 | 0.28 | -0.34 | 1.62 | 1.18 | 4.48 | 1.64 | 1.26 | 2.53 | 2.32 | 0.01 |
| Nucleocapsid Phosphoprotein | aa245-259 | GQQQQGQTVTKKSAA | -0.82 | -0.82 | -0.78 | -0.78 | -0.83 | -0.15 | -0.33 | 3.41 | -0.09 | -0.80 | 1.21 | -0.21 | 0.05 |
| Nucleocapsid Phosphoprotein | aa285-299 | GPEQTQGNFGDQELI | -0.31 | 1.03 | 0.95 | -0.24 | -0.70 | 0.37 | 0.70 | 3.43 | 2.28 | 0.84 | 1.89 | 1.60 | 0.03 |
| Nucleocapsid Phosphoprotein | aa291-305 | GNFGDQELIRQGTDY | 0.24 | 1.09 | 1.14 | -0.43 | -0.44 | 1.26 | 0.93 | 3.77 | 2.20 | 1.14 | 2.96 | 1.77 | 0.03 |
| Nucleocapsid Phosphoprotein | aa307-321 | HWPQIAQFAPSASAF | -0.82 | -0.46 | -0.78 | -0.80 | -0.87 | -0.69 | -0.43 | 3.11 | -0.25 | -0.72 | 2.10 | 0.39 | 0.01 |
