# Supplementary material for: Humoral Immune Response Profile of COVID-19 Reveals Severity and Variant-Specific Epitopes: Lessons from SARS-CoV-2 Peptide Microarray
Source: Viruses. 2023 Jan 15;15(1):248. doi: 10.3390/v15010248 (PMC9866125; doi:10.3390/v15010248)
Supplement: Supplementary file 1 [file viruses-15-00248-s001.zip › Table S8.docx]

Table S8: Discriminatory epitope for IgA

| Protein | Immunogenic Epitope | No. of Peptides |
| --- | --- | --- |
| nsp3 | FLTENLLLYIDINGN | 1 |
| nsp3 | LKHGTFTCASEYTGN | 1 |
| nsp3 | VLGLAAIMQLFFSYF | 1 |
| nsp12 | QTTPGSGVPVVDSYY | 1 |
| nsp12 | PLTKHPNQEYADVFH | 1 |
| nsp14 | GDQFKHLIPLMYKGL | 1 |
