# Supplementary material for: Humoral Immune Response Profile of COVID-19 Reveals Severity and Variant-Specific Epitopes: Lessons from SARS-CoV-2 Peptide Microarray
Source: Viruses. 2023 Jan 15;15(1):248. doi: 10.3390/v15010248 (PMC9866125; doi:10.3390/v15010248)
Supplement: Supplementary file 1 [file viruses-15-00248-s001.zip › Table S9.docx]

Table S9: Discriminatory epitopes for IgG

| Protein | Immunogenic Epitope | No. of Peptides |
| --- | --- | --- |
| nsp1 | TCGLVEVEKGVLPQL | 1 |
| nsp1 | VEVEKGVLPQLEQPYVF | 2 |
| nsp1 | GAGGHSYGADLKSFD | 1 |
| nsp1 | HSYGADLKSFDLGDELG | 2 |
| nsp1 | LKSFDLGDELGTDPYEDFQENWN | 5 |
| nsp2 | ELNGGAYTRYVDNNF | 1 |
| nsp2 | GAYTRYVDNNFCGPDGYPLEC | 4 |
| nsp2 | RGVYCCREHEHEIAWYTER | 3 |
| nsp2 | EIKLAKKFDTFNGEC | 1 |
| nsp2 | KCDHCGETSWQTGDF | 1 |
| nsp2 | HNSEVGPEHSLAEYH | 1 |
| nsp2 | RTIAFGGCVFSYVGC | 1 |
| nsp2 | NIVGDFKLNEEIAII | 1 |
| nsp2 | ASTSAFVETVKGLDY | 1 |
| nsp2 | IFGTVYEKLKPVLDW | 1 |
| nsp2 | LDWLEEKFKEGVEFLRDGWEI | 4 |
| nsp3 | KVTFGDDTVIEVQGY | 1 |
| nsp3 | YTVELGTEVNEFACV | 1 |
| nsp3 | VSELLTPLGIDLDEWSM | 2 |
| nsp3 | PLGIDLDEWSMATYYLFDESGEF | 5 |
| nsp3 | FDESGEFKLASHMYC | 1 |
| nsp3 | GEFKLASHMYCSFYP | 1 |
| nsp3 | CSFYPPDEDEEEGDCEE | 2 |
| nsp3 | GDCEEEEFEPSTQYEYGTEDDYQ | 5 |
| nsp3 | EYGTEDDYQGKPLEF | 1 |
| nsp3 | NSFSGYLKLTDNVYI | 1 |
| nsp3 | KGEDIQLLKSAYENF | 1 |
| nsp3 | RTNVYLAVFDKNLYD | 1 |
| nsp3 | TLEETKFLTENLLLY | 1 |
| nsp3 | TKFLTENLLLYIDINGN | 2 |
| nsp3 | HPDSATLVSDIDITF | 1 |
| nsp3 | GIKIQEGVVDYGARFYFYT | 3 |
| nsp3 | LNDLNETLVTMPLGY | 1 |
| nsp3 | YVTHGLNLEEAARYM | 1 |
| nsp3 | IETISLAGSYKDWSY | 1 |
| nsp3 | WSYSGQSTQLGIEFL | 1 |
| nsp3 | QLGIEFLKRGDKSVY | 1 |
| nsp3 | NPTTFHLDGEVITFD | 1 |
| nsp3 | HTQVVDMSMTYGQQF | 1 |
| nsp3 | FYVLPNDDTLRVEAFEYYH | 3 |
| nsp3 | TLRVEAFEYYHTTDPSF | 2 |
| nsp3 | FEYYHTTDPSFLGRY | 1 |
| nsp3 | NGLTSIKWADNNCYL | 1 |
| nsp3 | IELKFNPPALQDAYY | 1 |
| nsp3 | AGEAANFCALILAYC | 1 |
| nsp3 | VGELGDVRETMSYLF | 1 |
| nsp3 | LKHGTFTCASEYTGN | 1 |
| nsp3 | TCASEYTGNYQCGHY | 1 |
| nsp3 | HITSKETLYCIDGAL | 1 |
| nsp3 | TKSSEYKGPITDVFY | 1 |
| nsp3 | GVVCTEIDPKLDNYY | 1 |
| nsp3 | TEIDPKLDNYYKKDNSY | 2 |
| nsp3 | YFTEQPIDLVPNQPY | 1 |
| nsp3 | LVPNQPYPNASFDNF | 1 |
| nsp3 | FKFVCDNIKFADDLN | 1 |
| nsp3 | PASRELKVTFFPDLN | 1 |
| nsp3 | TFFPDLNGDVVAIDY | 1 |
| nsp3 | SFKKGAKLLHKPIVW | 1 |
| nsp3 | KATYKPNTWCIRCLW | 1 |
| nsp3 | LECNVKTTEVVGDII | 1 |
| nsp3 | ITEEVGHTDLMAAYV | 1 |
| nsp3 | LATHGLAAVNSVPWD | 1 |
| nsp3 | TRCLNRVCTNYMPYF | 1 |
| nsp3 | KSVGKFCLEASFNYL | 1 |
| nsp3 | NLGMPSYCTGYREGYLN | 2 |
| nsp3 | CSVCLSGLDSLDTYP | 1 |
| nsp3 | FKWDLTAFGLVAEWF | 1 |
| nsp3 | AFGLVAEWFLAYILF | 1 |
| nsp3 | VLGLAAIMQLFFSYF | 1 |
| nsp3 | SYFAVHFISNSWLMWLI | 2 |
| nsp3 | SAMVRMYIFFASFYY | 1 |
| nsp3 | FYYVWKSYVHVVDGC | 1 |
| nsp3 | VYANGGKGFCKLHNW | 1 |
| nsp3 | KGFCKLHNWNCVNCDTF | 2 |
| nsp3 | FCAGSTFISDEVARD | 1 |
| nsp3 | DSVTVKNGSIHLYFD | 1 |
| nsp3 | HLYFDKAGQKTYERHSL | 2 |
| nsp3 | ERHSLSHFVNLDNLRAN | 2 |
| nsp3 | EVTGDSCNNYMLTYN | 1 |
| nsp3 | VENMTPRDLGACIDC | 1 |
| nsp4 | NWLKQLIKVTLVFLF | 1 |
| nsp4 | QLIKVTLVFLFVAAIFY | 2 |
| nsp4 | DTCFANKHADFDTWF | 1 |
| nsp4 | PGTILRTTNGDFLHF | 1 |
| nsp4 | DFLHFLPRVFSAVGN | 1 |
| nsp4 | NICYTPSKLIEYTDF | 1 |
| nsp4 | SKLIEYTDFATSACV | 1 |
| nsp4 | FATSACVLAAECTIF | 1 |
| nsp4 | TIFKDASGKPVPYCY | 1 |
| nsp4 | EGSVAYESLRPDTRY | 1 |
| nsp4 | EGSVRVVTTFDSEYCRH | 2 |
| nsp4 | ERSEAGVCVSTSGRW | 1 |
| nsp4 | VCVSTSGRWVLNNDYYR | 2 |
| nsp4 | WVLNNDYYRSLPGVF | 1 |
| nsp4 | LAYYFMRFRRAFGEYSH | 2 |
| nsp4 | GEYSHVVAFNTLLFL | 1 |
| nsp4 | FYLTNDVSFLAHIQW | 1 |
| nsp4 | NDVSFLAHIQWMVMF | 1 |
| nsp4 | AHIQWMVMFTPLVPFWI | 2 |
| nsp4 | FSTFEEAALCTFLLN | 1 |
| nsp4 | KALNDFSNSGSDVLY | 1 |
| nsp5 | QVTCGTTTLNGLWLD | 1 |
| nsp5 | GTTTLNGLWLDDVVY | 1 |
| nsp5 | LNGLWLDDVVYCPRH | 1 |
| nsp5 | HVICTSEDMLNPNYE | 1 |
| nsp5 | TSEDMLNPNYEDLLI | 1 |
| nsp5 | LNGSCGSVGFNIDYDCVSFCY | 4 |
| nsp5 | LPTGVHAGTDLEGNFYGPF | 3 |
| nsp5 | LAWLYAAVINGDRWF | 1 |
| nsp5 | YAAVINGDRWFLNRF | 1 |
| nsp5 | NGRTILGSALLEDEF | 1 |
| nsp5 | GSALLEDEFTPFDVV | 1 |
| nsp6 | SLLVLVQSTQWSLFF | 1 |
| nsp6 | LVQSTQWSLFFFLYE | 1 |
| nsp6 | WSLFFFLYENAFLPF | 1 |
| nsp6 | FAMGIIAMSAFAMMF | 1 |
| nsp6 | LILMTARTVYDDGAR | 1 |
| nsp6 | KVYYGNALDQAISMW | 1 |
| nsp6 | GNALDQAISMWALII | 1 |
| nsp6 | MFLARGIVFMCVEYC | 1 |
| nsp6 | RGIVFMCVEYCPIFF | 1 |
| nsp6 | FMCVEYCPIFFITGN | 1 |
| nsp6 | LQCIMLVYCFLGYFC | 1 |
| nsp6 | MLVYCFLGYFCTCYF | 1 |
| nsp6 | CFLGYFCTCYFGLFC | 1 |
| nsp6 | CTCYFGLFCLLNRYF | 1 |
| nsp6 | CLLNRYFRLTLGVYD | 1 |
| nsp6 | LTLGVYDYLVSTQEFRY | 2 |
| nsp7 | KLWAQCVQLHNDILL | 1 |
| nsp8 | ATAQEAYEQAVANGD | 1 |
| nsp8 | NDALNNIINNARDGC | 1 |
| nsp8 | NTCDGTTFTYASALWEI | 2 |
| nsp8 | SKIVQLSEISMDNSP | 1 |
| nsp9 | TTQTACTDDNALAYY | 1 |
| nsp9 | LALLSDLQDLKWARF | 1 |
| nsp10 | CAFAVDAAKAYKDYL | 1 |
| nsp10 | CRCHIDHPNPKGFCD | 1 |
| nsp12 | TGTSTDVVYRAFDIYND | 2 |
| nsp12 | FQEKDEDDNLIDSYF | 1 |
| nsp12 | NLLKDCPAVAKHDFF | 1 |
| nsp12 | TKYTMADLVYALRHFDEGNCD | 4 |
| nsp12 | EILVTYNCCDDDYFN | 1 |
| nsp12 | TYNCCDDDYFNKKDWYDFVEN | 4 |
| nsp12 | VGVLTLDNQDLNGNWYD | 2 |
| nsp12 | DNQDLNGNWYDFGDFIQ | 2 |
| nsp12 | QTTPGSGVPVVDSYY | 1 |
| nsp12 | KYDFTEERLKLFDRYFK | 2 |
| nsp12 | QTYHPNCVNCLDDRCIL | 2 |
| nsp12 | NQDVNLHSSRLSFKE | 1 |
| nsp12 | NLHSSRLSFKELLVY | 1 |
| nsp12 | ALTNNVAFQTVKPGN | 1 |
| nsp12 | QTVKPGNFNKDFYDF | 1 |
| nsp12 | FFKEGSSVELKHFFF | 1 |
| nsp12 | FFFAQDGNAAISDYDYYRYNL | 4 |
| nsp12 | DYDYYRYNLPTMCDI | 1 |
| nsp12 | LFVVEVVDKYFDCYD | 1 |
| nsp12 | EVVDKYFDCYDGGCI | 1 |
| nsp12 | QVIVNNLDKSAGFPF | 1 |
| nsp12 | KWGKARLYYDSMSYE | 1 |
| nsp12 | ARLYYDSMSYEDQDALFAY | 3 |
| nsp12 | TVYSDVENPHLMGWDYP | 2 |
| nsp12 | AQVLSEMVMCGGSLY | 1 |
| nsp12 | KYVRNLQHRLYECLY | 1 |
| nsp12 | NLQHRLYECLYRNRD | 1 |
| nsp12 | RLYECLYRNRDVDTDFVNEFYAY | 5 |
| nsp12 | HFSMMILSDDAVVCF | 1 |
| nsp12 | HTMLVKQGDDYVYLPYP | 2 |
| nsp12 | QGDDYVYLPYPDPSR | 1 |
| nsp12 | PSRILGAGCFVDDIV | 1 |
| nsp12 | PLTKHPNQEYADVFHLYLQYI | 4 |
| nsp12 | DNTSRYWEPEFYEAM | 1 |
| nsp12 | RYWEPEFYEAMYTPH | 1 |
| nsp13 | ACIRRPFLCCKCCYD | 1 |
| nsp13 | CNAPGCDVTDVTQLY | 1 |
| nsp13 | DVTDVTQLYLGGMSY | 1 |
| nsp13 | SDNVTDFNAIATCDW | 1 |
| nsp13 | FNAIATCDWTNAGDYIL | 2 |
| nsp13 | GEYTFEKGDYGDAVV | 1 |
| nsp13 | LVPQEHYVRITGLYP | 1 |
| nsp13 | AAVDALCEKALKYLP | 1 |
| nsp13 | PRTLLTKGTLEPEYF | 1 |
| nsp13 | QIGVVREFLTRNPAW | 1 |
| nsp13 | TQTVDSSQGSEYDYV | 1 |
| nsp13 | AKVGILCIMSDRDLY | 1 |
| nsp13 | DRDLYDKLQFTSLEI | 1 |
| nsp14 | GFKMNYQVNGYPNMF | 1 |
| nsp14 | RHVRAWIGFDVEGCH | 1 |
| nsp14 | QFKHLIPLMYKGLPW | 1 |
| nsp14 | RATCFSTASDTYACW | 1 |
| nsp14 | SDTYACWHHSIGFDYVYNPFMID | 5 |
| nsp14 | FDYVYNPFMIDVQQWGF | 2 |
| nsp14 | WGFTGNLQSNHDLYC | 1 |
| nsp14 | DAIMTRCLAVHECFV | 1 |
| nsp14 | HECFVKRVDWTIEYP | 1 |
| nsp14 | PCSDKAYKIEELFYS | 1 |
| nsp14 | HSDKFTDGVCLFWNC | 1 |
| nsp14 | VLSNLNLPGCDGGSLYV | 2 |
| nsp14 | FDKSAFVNLKQLPFFYY | 2 |
| nsp14 | PCESHGKQVVSDIDY | 1 |
| nsp14 | LGGAVCRHHANEYRLYL | 2 |
| nsp14 | RHHANEYRLYLDAYN | 1 |
| nsp14 | LDAYNMMISAGFSLW | 1 |
| nsp14 | GFSLWVYKQFDTYNL | 1 |
| nsp14 | WVYKQFDTYNLWNTF | 1 |
| nsp15 | HFDGQQGEVPVSIIN | 1 |
| nsp15 | TVYTKVDGVDVELFE | 1 |
| nsp15 | ENKTTLPVNVAFELW | 1 |
| nsp15 | NLGVDIAANTVIWDY | 1 |
| nsp15 | TVFFDGRVDGQVDLFRN | 2 |
| nsp15 | LQEFKPRSQMEIDFL | 1 |
| nsp15 | QMEIDFLELAMDEFIER | 2 |
| nsp15 | LAMDEFIERYKLEGYAFEH | 3 |
| nsp15 | KLEGYAFEHIVYGDFSH | 2 |
| nsp15 | FEHIVYGDFSHSQLGGLHL | 3 |
| nsp15 | LAKRFKESPFELEDF | 1 |
| nsp15 | GSSKCVCSVIDLLLDDFVEII | 4 |
| nsp15 | VKVTIDYTEISFMLW | 1 |
| nsp15 | SFMLWCKDGHVETFY | 1 |
| nsp16 | TLTLAVPYNMRVIHF | 1 |
| nsp16 | PTGTLLVDSDLNDFV | 1 |
| nsp16 | HSWNADLYKLMGHFAWW | 2 |
| nsp16 | GCNYLGKPREQIDGY | 1 |
| nsp16 | KPREQIDGYVMHANYIFWR | 3 |
| nsp16 | PIQLSSYSLFDMSKF | 1 |
| GS Linker | KVFRSSVLHSTQDLF | 1 |
| Spike Protein | SSVLHSTQDLFLPFF | 1 |
| Spike Protein | TQDLFLPFFSNVTWF | 1 |
| Spike Protein | HVSGTNGTKRFDNPV | 1 |
| Spike Protein | KRFDNPVLPFNDGVYFA | 2 |
| Spike Protein | IVNNATNVVIKVCEFQF | 2 |
| Spike Protein | VIKVCEFQFCNDPFL | 1 |
| Spike Protein | CEFQFCNDPFLGVYY | 1 |
| Spike Protein | VYYHKNNKSWMESEFRV | 2 |
| Spike Protein | FRVYSSANNCTFEYV | 1 |
| Spike Protein | VSQPFLMDLEGKQGN | 1 |
| Spike Protein | KNLREFVFKNIDGYFKI | 2 |
| Spike Protein | SSGWTAGAAAYYVGY | 1 |
| Spike Protein | FPNITNLCPFGEVFN | 1 |
| Spike Protein | TNLCPFGEVFNATRF | 1 |
| Spike Protein | GEVFNATRFASVYAW | 1 |
| Spike Protein | RKRISNCVADYSVLY | 1 |
| Spike Protein | LNDLCFTNVYADSFV | 1 |
| Spike Protein | QIAPGQTGKIADYNY | 1 |
| Spike Protein | TGKIADYNYKLPDDF | 1 |
| Spike Protein | AWNSNNLDSKVGGNYNYLY | 3 |
| Spike Protein | YRLFRKSNLKPFERD | 1 |
| Spike Protein | SNLKPFERDISTEIY | 1 |
| Spike Protein | AGSTPCNGVEGFNCYFP | 2 |
| Spike Protein | NGVEGFNCYFPLQSY | 1 |
| Spike Protein | VGYQPYRVVVLSFELLH | 2 |
| Spike Protein | AGCLIGAEHVNNSYECDIP | 3 |
| Spike Protein | SVDCTMYICGDSTEC | 1 |
| Spike Protein | ILPDPSKPSKRSFIE | 1 |
| Spike Protein | PSKPSKRSFIEDLLF | 1 |
| Spike Protein | TLADAGFIKQYGDCLGD | 2 |
| Spike Protein | YGDCLGDIAARDLIC | 1 |
| Spike Protein | ALQIPFAMQMAYRFN | 1 |
| Spike Protein | AYRFNGIGVTQNVLY | 1 |
| Spike Protein | QNVLYENQKLIANQF | 1 |
| Spike Protein | ICHDGKAHFPREGVF | 1 |
| Spike Protein | FPREGVFVSNGTHWF | 1 |
| Spike Protein | SNGTHWFVTQRNFYE | 1 |
| Spike Protein | QIITTDNTFVSGNCD | 1 |
| Spike Protein | DPLQPELDSFKEELD | 1 |
| Spike Protein | DKYFKNHTSPDVDLGDI | 2 |
| Spike Protein | GINASVVNIQKEIDR | 1 |
| Spike Protein | ESLIDLQELGKYEQY | 1 |
| Spike Protein | QELGKYEQYIKWPWYIW | 2 |
| Spike Protein | CCSCGSCCKFDEDDSEP | 2 |
| Spike Protein | HYTGSGSGSGMDLFM | 1 |
| Orf3a Protein | YSHLLLVAAGLEAPFLYLY | 3 |
| Orf3a Protein | WKCRSKNPLLYDANYFLCW | 3 |
| Orf3a Protein | YDANYFLCWHTNCYDYCIPYN | 4 |
| Orf3a Protein | SSIVITSGDGTTSPI | 1 |
| Orf3a Protein | ISEHDYQIGGYTEKW | 1 |
| Orf3a Protein | WESGVKDCVVLHSYF | 1 |
| Orf3a Protein | VKDCVVLHSYFTSDYYQLY | 3 |
| Orf3a Protein | LSTDTGVEHVTFFIY | 1 |
| Orf3a Protein | YNKIVDEPEEHVQIH | 1 |
| Orf3a Protein | GSSGVVNPVMEPIYDEPTT | 3 |
| Orf3a Protein | VMEPIYDEPTTTTSV | 1 |
| Orf3a Protein | SFVSEETGTLIVNSV | 1 |
| Envelope Protein | LEQWNLVIGFLFLTW | 1 |
| Membrane Glycoprotein | WICLLQFAYANRNRFLY | 2 |
| Membrane Glycoprotein | GLMWLSYFIASFRLF | 1 |
| Membrane Glycoprotein | PKEITVATSRTLSYY | 1 |
| Membrane Glycoprotein | QRVAGDSGFAAYSRY | 1 |
| Membrane Glycoprotein | IGNYKLNTDHSSSSDNI | 2 |
| Orf6 Protein | IMRTFKVSIWNLDYI | 1 |
| Orf6 Protein | KYSQLDEEQPMEIDG | 1 |
| Orf6 Protein | ILFLALITLATCELYHYQECVRG | 5 |
| Orf7a Protein | PCSSGTYEGNSPFHP | 1 |
| Orf8 Protein | TQHQPYVVDDPCPIHFY | 2 |
| Orf8 Protein | VVDDPCPIHFYSKWY | 1 |
| Orf8 Protein | VDEAGSKSPIQYIDIGN | 2 |
| Orf8 Protein | KSPIQYIDIGNYTVSCL | 2 |
| Orf8 Protein | KLGSLVVRCSFYEDFLEYHDVRVVL | 6 |
| Nucleocapsid Phosphoprotein | VPINTNSSPDDQIGY | 1 |
| Nucleocapsid Phosphoprotein | GGDGKMKDLSPRWYF | 1 |
| Nucleocapsid Phosphoprotein | EAGLPYGANKDGIIW | 1 |
| Nucleocapsid Phosphoprotein | VLQLPQGTTLPKGFYAEGS | 3 |
| Nucleocapsid Phosphoprotein | SRGTSPARMAGNGGD | 1 |
| Nucleocapsid Phosphoprotein | GNGGDAALALLLLDR | 1 |
| Nucleocapsid Phosphoprotein | GQQQQGQTVTKKSAA | 1 |
| Nucleocapsid Phosphoprotein | GPEQTQGNFGDQELI | 1 |
| Nucleocapsid Phosphoprotein | GNFGDQELIRQGTDY | 1 |
| Nucleocapsid Phosphoprotein | HWPQIAQFAPSASAF | 1 |
